# Well-Defined Heparin Mimetics Can Inhibit Binding of the Trimeric Spike of SARS-CoV-2 in a Length-Dependent Manner

**DOI:** 10.1021/jacsau.3c00042

**Published:** 2023-04-06

**Authors:** Lifeng Sun, Pradeep Chopra, Ilhan Tomris, Roosmarijn van der Woude, Lin Liu, Robert P. de Vries, Geert-Jan Boons

**Affiliations:** † Department of Chemical Biology and Drug Discovery, Utrecht Institute for Pharmaceutical Sciences, Utrecht University, 3584 CG Utrecht, The Netherlands; ‡ Complex Carbohydrate Research Center, The University of Georgia, Athens, Georgia 30602, United States; § Bijvoet Center for Biomolecular Research, Utrecht University, 3584 CG Utrecht, The Netherlands; ∥ Chemistry Department, The University of Georgia, Athens, Georgia 30602, United States

**Keywords:** chemoenzymatic synthesis, multivalent, glycosyl transferases, glyco-mimetics, coronavirus

## Abstract

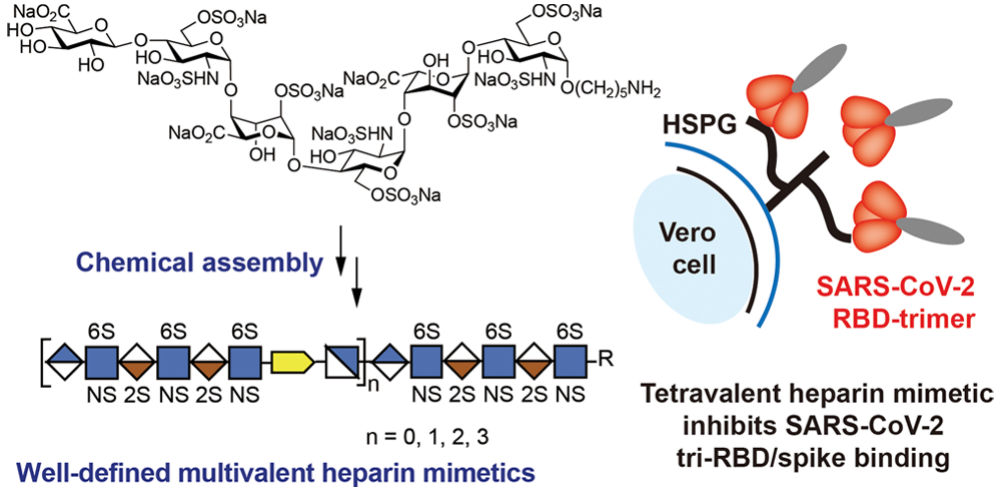

The emergence of new SARS-CoV-2 variants and the dangers of long-covid necessitate the development of broad-acting therapeutics that can reduce viral burden. SARS-CoV-2 employs heparan sulfate (HS) as an initial cellular attachment factor, and therefore, there is interest in developing heparin as a therapeutic for SARS-CoV-2. Its use is, however, complicated by structural heterogeneity and the risk of causing bleeding and thrombocytopenia. Here, we describe the preparation of well-defined heparin mimetics by a controlled head-to-tail assembly of HS oligosaccharides having an alkyne or azide moiety by copper-catalyzed azide-alkyne cycloaddition (CuAAC). Alkyne- and azide-containing sulfated oligosaccharides were prepared from a common precursor by modifying an anomeric linker with 4-pentynoic acid and by enzymatic extension with an *N*-acetyl-glucosamine having an azide moiety at C-6 (GlcNAc6N_3_), respectively, followed by CuAAC. The process of enzymatic extension with GlcNAc6N_3_ followed by CuAAC with the desired alkyne-containing oligosaccharides could be repeated to give compounds composed of 20 and 27 monosaccharides, respectively. The heparin mimetics could inhibit the binding of the SARS-CoV-2 spike or RBD to immobilized heparin or to Vero E6 cells. The inhibitory potency increased with increasing chain length, and a compound composed of four sulfated hexasaccharides linked by triazoles had a similar potency as unfractionated heparin. Sequence analysis and HS microarray binding studies with a wide range of RBDs of variants of concern indicate that they have maintained HS-binding capabilities and selectivities. The heparin mimetics exhibit no or reduced binding to antithrombin-III and platelet factor 4, respectively, which are associated with side effects.

## Introduction

Severe acute respiratory syndrome-related coronavirus 2 (SARS-CoV-2) is causing an unprecedented worldwide pandemic that is greatly impacting global health systems and disrupting many aspects of society.^1^ The spike glycoprotein of SARS-CoV-2, which is a homotrimer composed of S1 and S2 subunits, mediates viral entry and is the main determinant of cell tropism and pathogenesis.^2,3^ The S1 subunits harbor a receptor-binding domain (RBD) that can bind with high affinity to angiotensin-converting enzyme 2 (ACE2) on the surface of host cells. Fusion with host cell membranes requires proteolytic cleavage at the S1/S2 boundary by TMPRSS2 or lysosomal cathepsins critical for infection.

Heparan sulfate (HS) serves as the initial point of attachment of SARS-CoV-2, allowing the virus to migrate through the glycocalyx.^4−6^ Treatment of cells with heparin lyases substantially reduces infectivity, highlighting the importance of HS for infectivity. The RBD harbors an HS-binding site adjacent to the ACE2-binding site and can simultaneously engage with ACE2 and HS. Computation studies have identified additional HS-binding sites in the spike glycoprotein.^4,5,7−13^

Heparin, which is structurally related to HS, can inhibit the binding of the RBD and spike of SARS-CoV-2 to relevant human cell lines and tissues.^7,10,14^ Furthermore, it can inhibit the entry of pseudoviruses carrying the SARS-CoV-2 spike or various SARS-CoV-2 strains into human cells.^4,5,7,14^ These observations have generated interest in developing heparin as a therapeutic for SARS-CoV-2 infections. Heparin is, however, structurally poorly defined and can cause bleeding, limiting the dose regime. Therefore, heparin analogues that can inhibit cell binding without causing bleeding have been examined; however, these compounds still suffer from structural heterogeneity, are less potent, and can interact with platelet factor 4 (PF4), thereby causing thrombocytopenia.^5,15^ Recently, it was shown that Pixatimod (PG545), which is a clinical-stage highly sulfated oligosaccharide having a steroidal aglycone moiety with immunomodulatory activity, can inhibit infection of Vero E6 and human cells by various strains of SARS-CoV-2.^16^

We have developed chemical and enzymatic methodologies that made it possible to prepare a large library of well-defined HS oligosaccharides differing in chain length, backbone composition, and sulfation pattern.^17−20^ The oligosaccharides were printed as a microarray that was used to probe the ligand requirements of the RBD and the spike protein of SARS-CoV-2.^6^ It was found that HS oligosaccharides were recognized in a length- and sequence-dependent manner and a hexasaccharide composed of IdoA2S-GlcNS6S repeating units was identified as the minimal binding epitope. Surface plasma resonance (SPR) binding studies showed that the hexasaccharide could inhibit the binding of RBD or the spike glycoprotein of SARS-CoV-2 to immobilized heparin. Similar inhibition studies showed, however, that unfractionated heparin (UFH) is a much more potent inhibitor.

Computation studies have indicated that the RBD of the spike of SARS-CoV-2 harbors an electropositive surface that can accommodate HS chains composed of as many as 20 monosaccharide units.^4^ Furthermore, the spike protein occurs as a homotrimer and thus exhibits three HS-binding sites, making it possible for polymeric compounds such as heparin to engage in a multivalent manner with the spike, resulting in high avidity of binding.^5,9,11−13^ Polymeric molecules can also exhibit higher affinities through rebinding events in which a single ligand–receptor complex dissociates and the presence of another close-by ligand will increase the probability of another binding event.^21^

**Figure 1:**
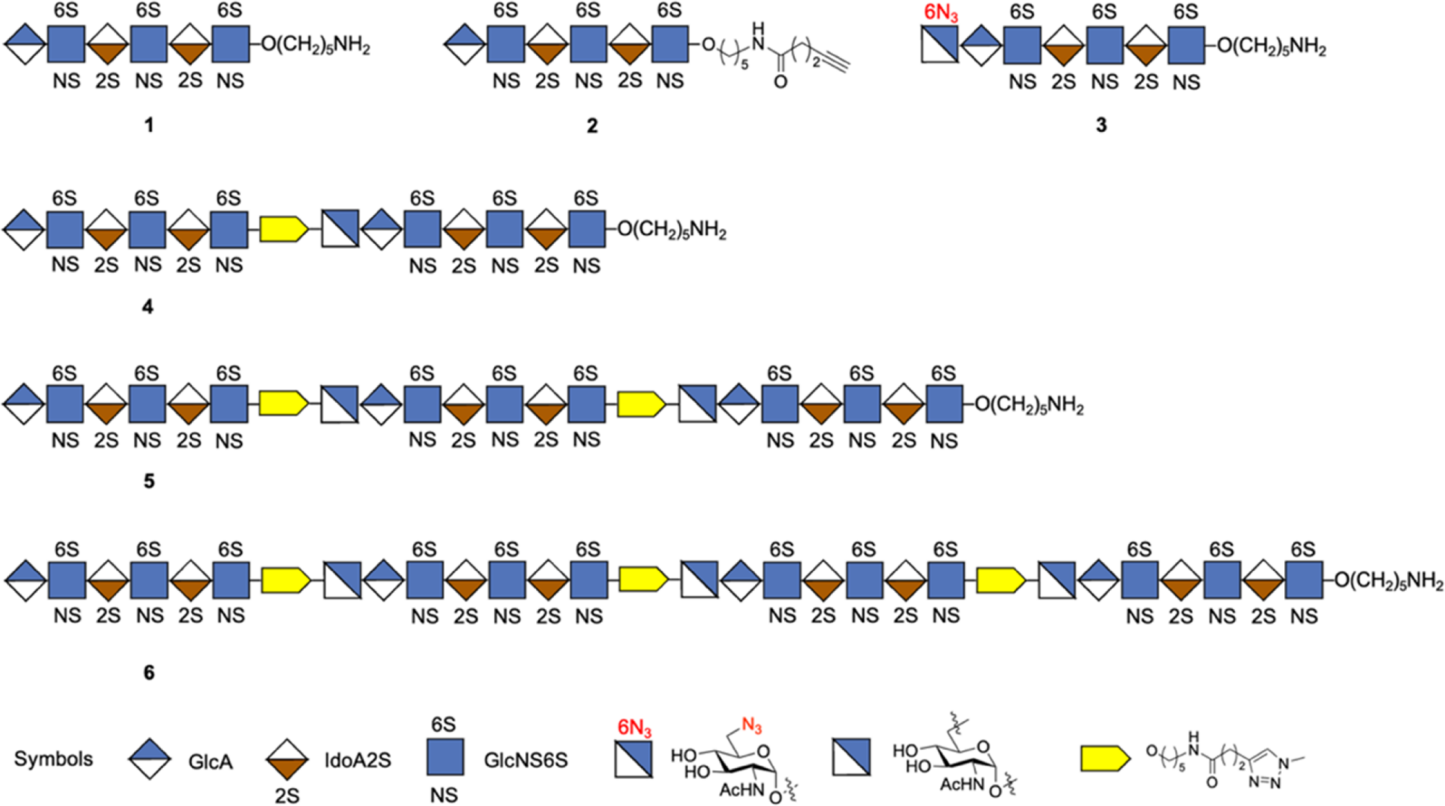
Fully synthetic and well-defined multivalent heparin mimetics assembled by CuAAC (**4**–**6**). Hexasaccharide **1** was prepared based on modular chemical synthesis, in which the anomeric linker can be modified with 4-pentynoic acid to give **2** or the nonreducing end can be extended with GlcNAc6N_3_ by the enzyme to give **3**. Symbol structures are also provided.

Here, we describe the preparation of well-defined heparin mimetics of different chain lengths (**1**, **4**, **5**, **6**, Figure 1) by the controlled copper-catalyzed azide-alkyne cycloaddition (CuAAC)^22^-mediated assembly of HS oligosaccharides having an alkyne (**2**) or azide (**3**) moiety.^23^ The heparin mimetics were examined for their ability to inhibit the binding of the SARS-CoV-2 spike to heparin immobilized on an SPR sensor chip. It was found that the inhibitory potency of the mimetics increased with increasing chain length and a compound composed of four sulfated hexasaccharides linked by triazoles (**6**) had a similar potency as UFH. Furthermore, the binding of trimeric RBDs from various SARS-CoV-2 strains to Vero E6 cells could be inhibited with these heparin mimetics in a length-dependent manner, and derivative **6** exhibited a similar potency as UFH. The alkyne- (**2**) and azido-containing (**3**) building blocks could readily be prepared from the common precursor **1** by modifying the anomeric linker at the reducing end with 4-pentynoic acid or by enzymatic extension of the nonreducing end by an *N*-acetyl-glucosamine having an azide moiety at C-6 (GlcNAc6N_3_), respectively, and then linked by CuAAC to give **4**. The process of enzymatic extension with GlcNAc6N_3_ followed by the CuAAC reaction with the desired alkyne-containing derivatives could be repeated to give compounds **5** and **6** composed of 20 and 27 monosaccharide units, respectively.

## Results and Discussion

### Chemoenzymatic Synthesis

Hexasaccharide **1** (GlcA-GlcNS6S-IdoA2S-GlcNS6S-IdoA2S-GlcNS6S) was selected as the precursor for the installation of an alkyne (**2**) and azide (**3**) moiety for subsequent CuAAC-mediated assembly of oligomers. Although the terminal glucuronic acid moiety (GlcA) of **1** reduces the affinity for the RBD of SARS-CoV-2, it makes it in an appropriate substrate for the recombinant glycosyl transferase *Pasteurella multocida* heparosan synthase (PmHS2).^24^ This bifunctional enzyme has α 1,4-*N*-acetyl-glucosaminyltransferase and β1,4-glucuronyltransferase activity by utilizing UDP-GlcNAc and UDP-GlcA, respectively, and can biosynthesize heparosan, which is the initial polymeric precursor for the biosynthesis of heparin and HS.^24,25^ Furthermore, PmHS2 exhibits some promiscuity for the donor substrates and can for example utilize UDP-GlcNAc6N_3_.^26^ It has an obligatory requirement for a terminal GlcA moiety as an acceptor, and thus, it was expected that treatment of **1** with UDP-GlcNAc-6N_3_ in the presence of PmHS2 will result in the incorporation of a GlcNAcN_3_ moiety. Furthermore, it was expected that the aminopentyl moiety of **1** can easily be reacted with *N*-hydroxysuccinimide (NHS)-activated 4-pentynoic acid to install an alkyne moiety. The azido and alkyne moieties were installed at a late stage of synthesis because it would allow the use of benzyl ethers as permanent protecting groups.

**Scheme 1:**
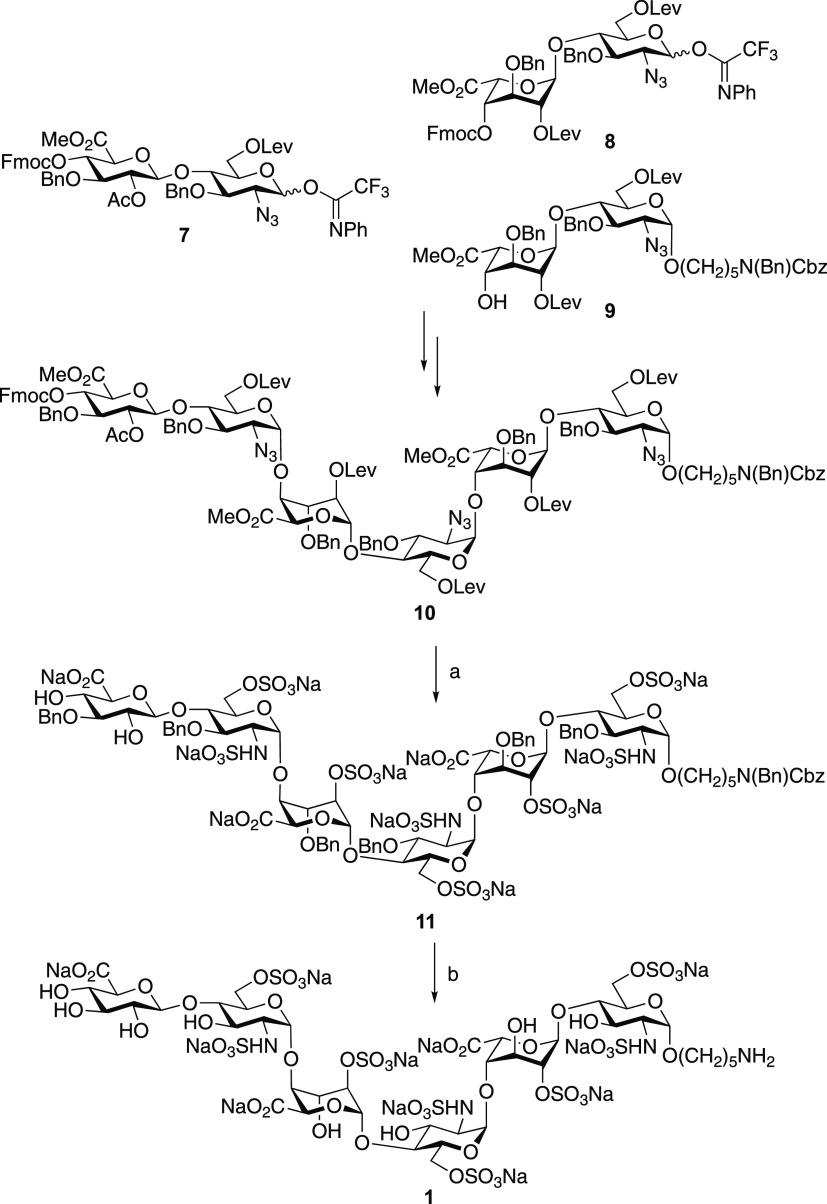
Synthesis of Hexasaccharide **1** Reagents and conditions: (a) (i) DCM/Et_3_N (4/1), 2 h; (ii) Ac_2_O, Py, 3 h; (iii) NH_2_NH_2_·AcOH, toluene/EtOH, 2 h; (iv) SO_3_·Py, DMF, 2 h; (v) 1 M LiOH, 30% H_2_O_2_, THF/H_2_O; (vi) PMe_3_/NaOH, THF/H_2_O, 2 h; (vii) SO_3_·Py, MeOH/Et_3_N, NaOH (pH∼11); **11**: 22% over seven steps; (b) Pd(OH)_2_/C, H_2_ (atm.), *t*-BuOH/H_2_O (1/1), 48 h; 85%.

Hexasaccharide **1** was prepared using modular disaccharides **7**, **8**, and **9** (Schemes 1 and S1).^17^ Triflic acid (TfOH)-catalyzed glycosylation of **8** with **9** gave a tetrasaccharide (**S1**) as only the α-anomer, which was treated with triethylamine (Et_3_N) in CH_2_Cl_2_ to remove 9-fluorenylmethyl carbonate (Fmoc) to give an acceptor (**S2**) that was further glycosylated with **7** to provide hexasaccharide **10** in an overall yield of 36%. The Fmoc protecting group of **10** was replaced by an acetyl ester, which was followed by selective cleavage of the levulinoyl (Lev) esters by treatment with hydrazine acetate, and the resulting hydroxyls were sulfated with the sulfur trioxide-pyridine complex (SO_3_·Py) in *N*,*N*-dimethylformamide (DMF). Next, the acetyl and methyl esters were cleaved with lithium hydroxide and hydrogen peroxide (LiOH/H_2_O_2_), which was followed by reduction of the azido groups using trimethyl phosphine in THF/H_2_O to give the free amines that were subjected to selective *N*-sulfation employing the SO_3_·Py complex in MeOH/Et_3_N in the presence of sodium hydroxide (NaOH) to provide **11**. The target hexasaccharide **1** was obtained by hydrogenolysis of compound **11** over palladium hydroxide (Pd(OH)_2_/C) in a mixture of *tert*-butanol/H_2_O (1/1, v/v).

**Scheme 2:**
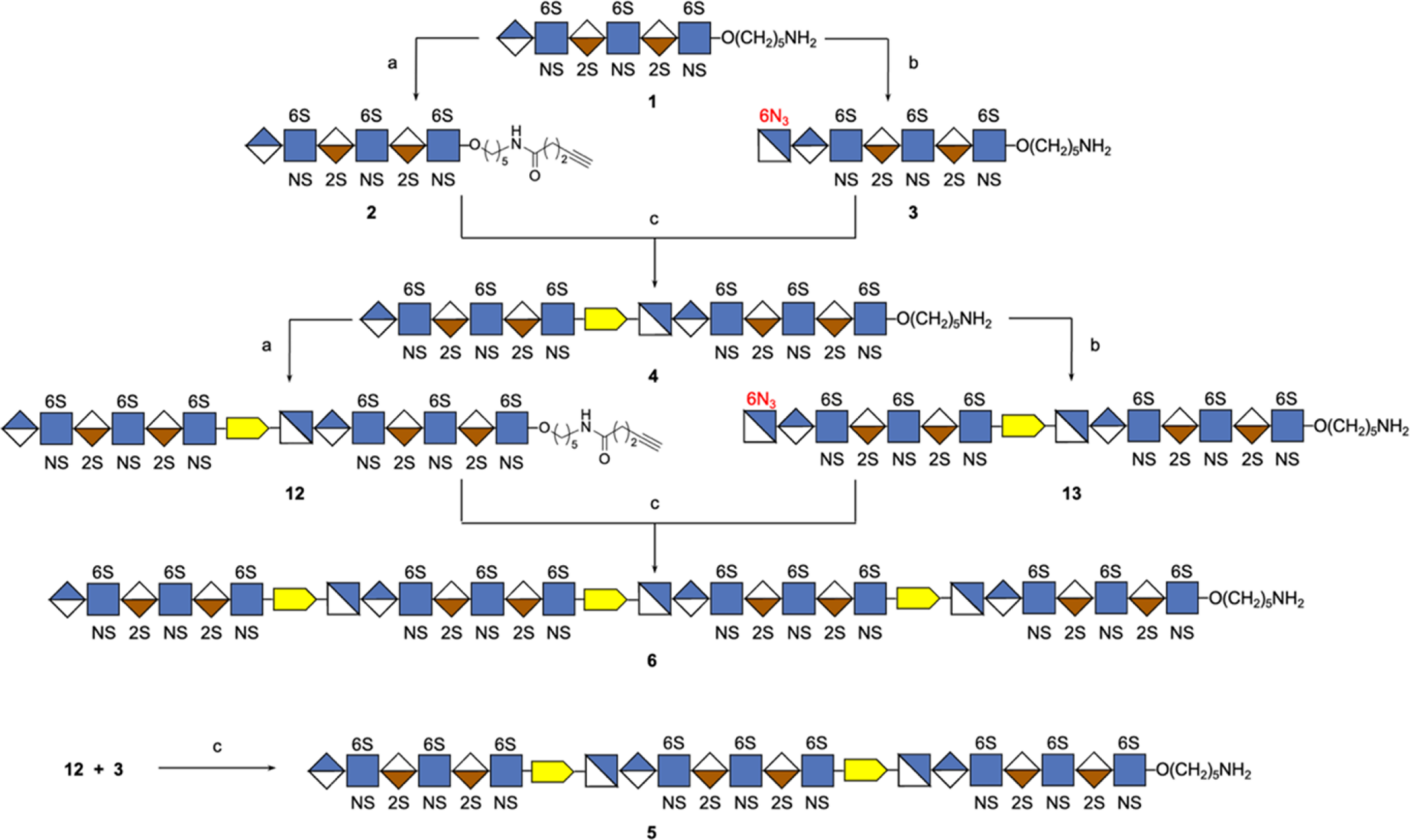
Assembly of Di-, Tri-, and Tetravalent Heparin Mimetics from Monovalent Hexasaccharide **1** Reagents and conditions: (a) NHS-4-pentynoic acid, 0.3 N NaHCO_3_ /ACN/MeOH, 2 h; **2**: 86%; **12**: 81%; (b) UDP-GlcNAc6N_3_, PmHS2, Tris-HCl (pH = 7.5), MnCl_2_, 37 °C, 24 h; **3**: 85%; **13**: 58%; (c) CuSO_4_, THPTA, aminoguanidine, sodium ascorbate, 37 °C, 24 h; **4**: 69%; **5**: 51%; **6**: 48%.

Next, attention was focused on the chemoenzymatic assembly of heparin mimetics **4**–**6** (Scheme 2). Thus, treatment of hexasaccharide **1** with UDP-GlcNAc6N_3_ in the presence of the recombinant glycosyl transferase PmHS2 gave, after purification by size exclusion chromatography (SEC) over a P6 Biogel and sodium exchange using Dowex 50 × 8 Na^+^ resin, heptasaccharide **3** in a yield of 85%. The reaction could be driven to completion by employing 1.5 equivalent of UDP-GlcNAc6N_3_ in the presence of a relatively high concentration of PmHS2 (200 μg/mL) for a period of 12 h. The use of a natural UDP-GlcNAc requires a somewhat smaller amount of enzyme (100 μg/mL) to drive the reaction to completion, indicating that the catalytic efficiency for the modified donor is somewhat lower. In parallel, hexasaccharide **1** was treated with NHS-activated 4-pentynoic acid in a mixture of 0.3 M NaHCO_3_/acetonitrile/MeOH (5/5/1, v/v/v), resulting in the formation of linker modified **2**. Alkyne- and azide-containing building blocks **2** and **3**, respectively, were subjected to CuAAC using CuSO_4_, sodium ascorbate, tris(3-hydroxypropyltriazolylmethyl)amine (THPTA), and aminoguanidine^22^ at 37 °C for 24 h to yield the dimeric heparin mimetic **4** in 69% yield. The aminopentyl moiety of the latter compound could readily be functionalized with an alkyne moiety by reaction with NHS-activated 4-pentynoic acid to give **12**. Furthermore, the nonreducing GlcA moiety of **4** could be extended by a GlcNAc6N_3_ moiety by treatment with UDP-GlcNAc6N_3_ in the presence of recombinant PmHS2 to give **13**. CuAAC of **12** with **3** resulted in the formation of trimeric **5**, whereas a 2 + 2 CuAAC-mediated coupling of **12** with **13** led to tetrameric **6**. Although trimer and tetramer formation had almost proceeded to competing without byproduct formation, the isolated yields were moderate (51 and 48%, respectively) most likely due to loss of product during purification, which was challenging because of the relatively small scales of the reactions.

The heparin mimetics were transformed into the sodium salt by treatment with the Dowex 50 × 8Na^+^ resin, and their structural integrity was confirmed by nuclear magnetic resonance (NMR) spectroscopy and electrospray ionization mass (ESI-MS) spectrometry. ^1^H NMR spectra of the compounds were fully assigned by one-dimensional (1D) and 2D NMR spectroscopies. The anomeric configuration was confirmed by ^1^*J*_C1,H1_ coupling constants (^1^*J*_C1,H1_ ∼171 Hz for α linkage) and ^13^C chemical shifts of C1 (<100 ppm for α linkage). The formation of a 1,2,3-triazole ring was confirmed by the presence of an aromatic proton at 7.85 ppm (Figures S2A, S2B and S2D) and chemical shift (δ) analysis of protons corresponding to GlcNAc6N_3_ (Figures S2A, S2C and S2D) and the alkyne moiety (Figures S2A, S2B and S2D).

### SPR and Cell-Based Inhibition Studies

**Figure 2:**
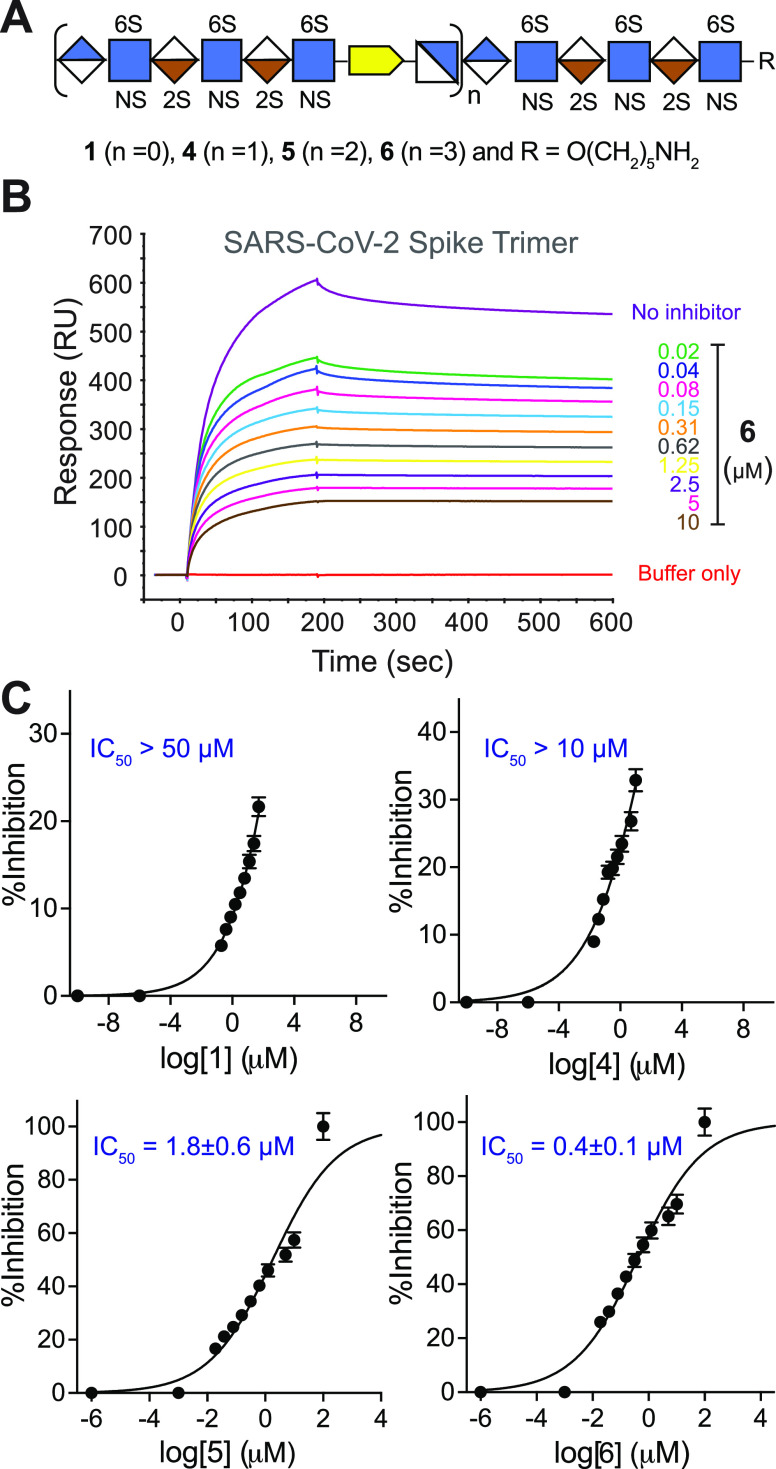
Surface plasmon resonance (SPR)-based competition assays on the heparin-immobilized surface. (A) Symbol structures of heparin mimetics employed in the SPR assay. (B) Representative SPR sensorgram of the spike trimer glycoprotein in the presence of tetravalent heparin mimetic **6**. (C) Inhibition curves of mimetics **1**, **4**–**6**; respective half-maximal inhibitory concentration (IC_50_) values are provided in each panel. See Figures S3–S6 for SPR sensorgrams. All experiments were performed at least two times. All assays were performed at 100 nM concentration of the spike trimer protein. Data were analyzed using Biacore T100 evaluation software, and the IC_50_ values were calculated using dose–response equations [nonlinear regression, log(inhibitor) vs response-variable slope (four parameters)] in Prism software 9 (GraphPad Software, Inc.).

The ability of the mono- (**1**), di- (**4**), tri- (**5**), and tetravalent (**6**) heparin mimetics to inhibit the binding of the trimeric spike glycoprotein of SARS-CoV-2 to heparin was evaluated. Various concentrations of **1**, **4**, **5**, and **6** (ranging from 10 to 0.02 μM, 2-fold dilutions) were premixed with the recombinant trimeric SARS-CoV-2 spike having a His-tag^27^ (100 nM) and then passed over an SPR sensor chip modified by heparin.^6,15^ It resulted in concentration-dependent reductions in SPR response units (RUs), and nonlinear fitting of log(inhibitor) *vs.* response with a variable slope was used to calculate half-maximal inhibitory (IC_50_) concentrations (50% reduction in RUs) (Figure 2A–C and Figures S3–S6). Monovalent **1** and divalent **4** exhibited only a modest inhibitory potential, and IC_50_ values were estimated to be larger than 50 and 10 μM, respectively. Trivalent **5** (20-mer) gave an IC_50_ value of 1.8 ± 0.6 μM, whereas tetravalent **6** (27-mer) gave an IC_50_ value of 0.4 ± 0.1 μM. Similar inhibition studies with UFH gave IC_50_ values of 0.1 ± 0.01 μg/mL (Figure S7), which, on a weight basis, is similar as for mimic **6** (3.6 ± 0.9 μg/mL).

**Figure 3:**
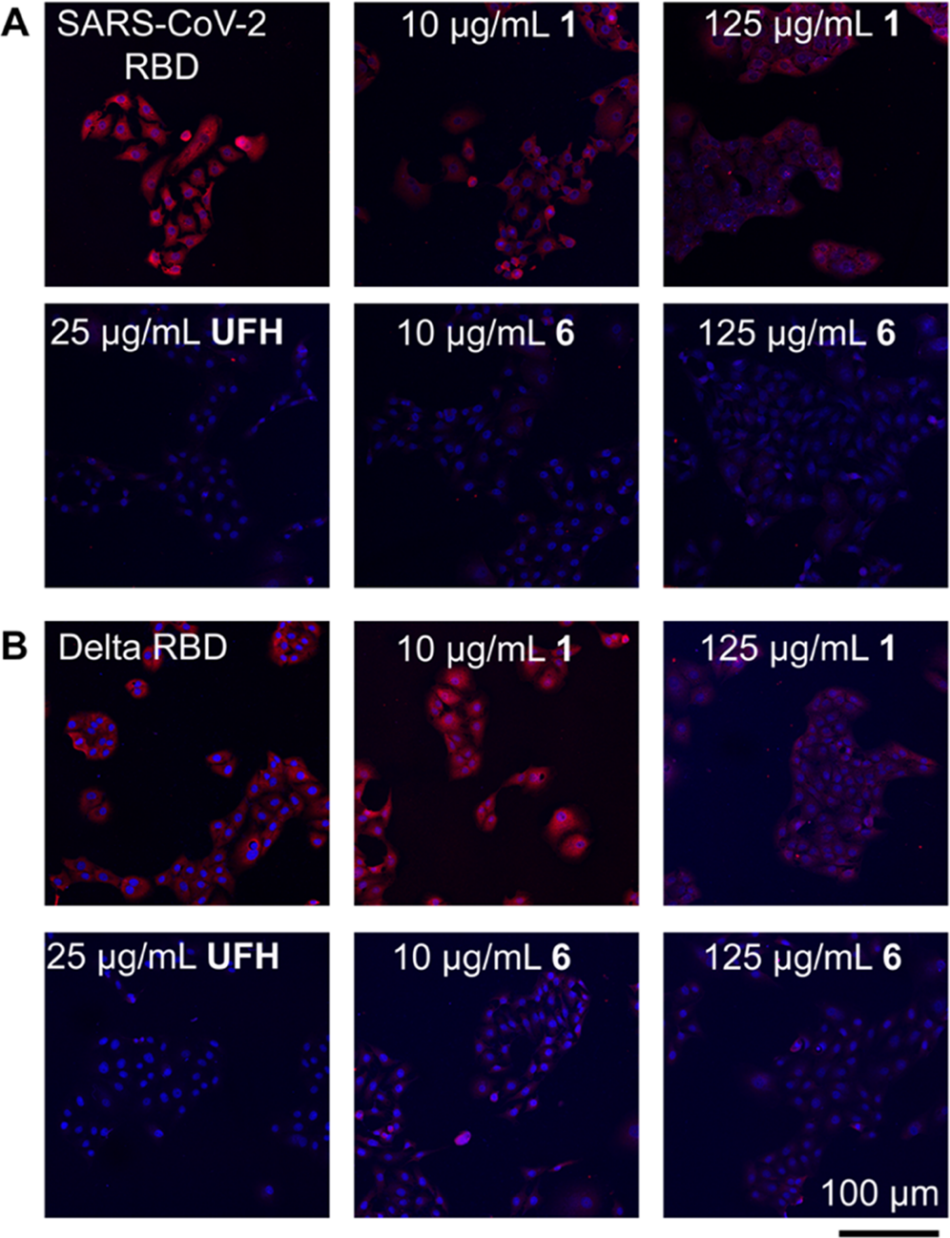
Binding of SARS-CoV-2 Wuhan and Delta RBD pretreated with heparin mimetics to Vero E6 cells. (A) Untreated SARS-CoV-2 Wuhan RBD and also pretreated with compound **1** (10 and 125 μg/mL, which equal to 5.0 and 62.6 μM, respectively), UFH (25 μg/mL), and compound **6** (10 and 125 μg/mL, which equal to 1.1 and 14.0 μM, respectively) were visualized by using overlay of DAPI (nuclear stain) and goat antimouse antibody labeled with AlexaFluor555 (RBD stain). Inhibition was observed for UFH and compound **6**, but no inhibition was detected with compound **1**. (B) Untreated delta RBD and pretreated with compound **1** (10 and 125 μg/mL, which equal to 5.0 and 62.6 μM, respectively), UFH (25 μg/mL), and compound **6** (10 and 125 μg/mL, which equal to 1.1 and 14.0 μM, respectively) show similar inhibitory effects as SARS-CoV-2 Wuhan RBD. UFH and compound **6** inhibit RBD-binding, while compound **1** does not display inhibition.

Compounds **1** and heparin mimetic **6** were further examined for their ability to inhibit the binding of trimeric RBDs from the original Wuhan and variant of concern (VoC) delta to Vero E6 cells. Previously, we demonstrated that recombinant RBDs (amino acid residues 319-541), C-terminal GCN4 trimerization domains fused to mOrange2, can readily be expressed in HEK293T cells and are ideally suited for receptor specificity studies.^28^ The two fluorescent RBDs were pretreated with compounds **1**, **6**, and UFH and then exposed to Vero E6 cells followed by imaging using fluorescent microscopy. The compounds were employed at concentrations of 10 and 125 μg/mL because earlier studies had shown that UFH fully inhibits cell binding at a concentration of 10–20 μg/mL.^6^ Compound **1** failed to invoke substantial inhibition of cell binding even at a high concentration of 125 μg/mL (62.6 μM) (Figure 3). On the other hand, compound **6** displayed inhibitory potency similar to UFH, eliciting complete inhibition at 10 μg/mL (1.1 μM). It was observed that the RBD derived from the delta variant was equally well inhibited when compared to that from the Wuhan strain.

### SARS-CoV-2 Variants of Concern Have Maintained HS-Binding Selectivities

Previously, we employed a microarray with over a hundred well-defined HS tetra-, hexa-, and octasaccharides to examine the ligand requirements of the RBD of the original SARS-CoV-2 Wuhan virus.^6^ It was found that a hexasaccharide composed of IdoA2S-GlcNS6S repeating units is the minimal epitope for binding. Perturbations in the HS backbone or sulfation pattern resulted in a substantial reduction or loss of binding.

**Figure 4:**
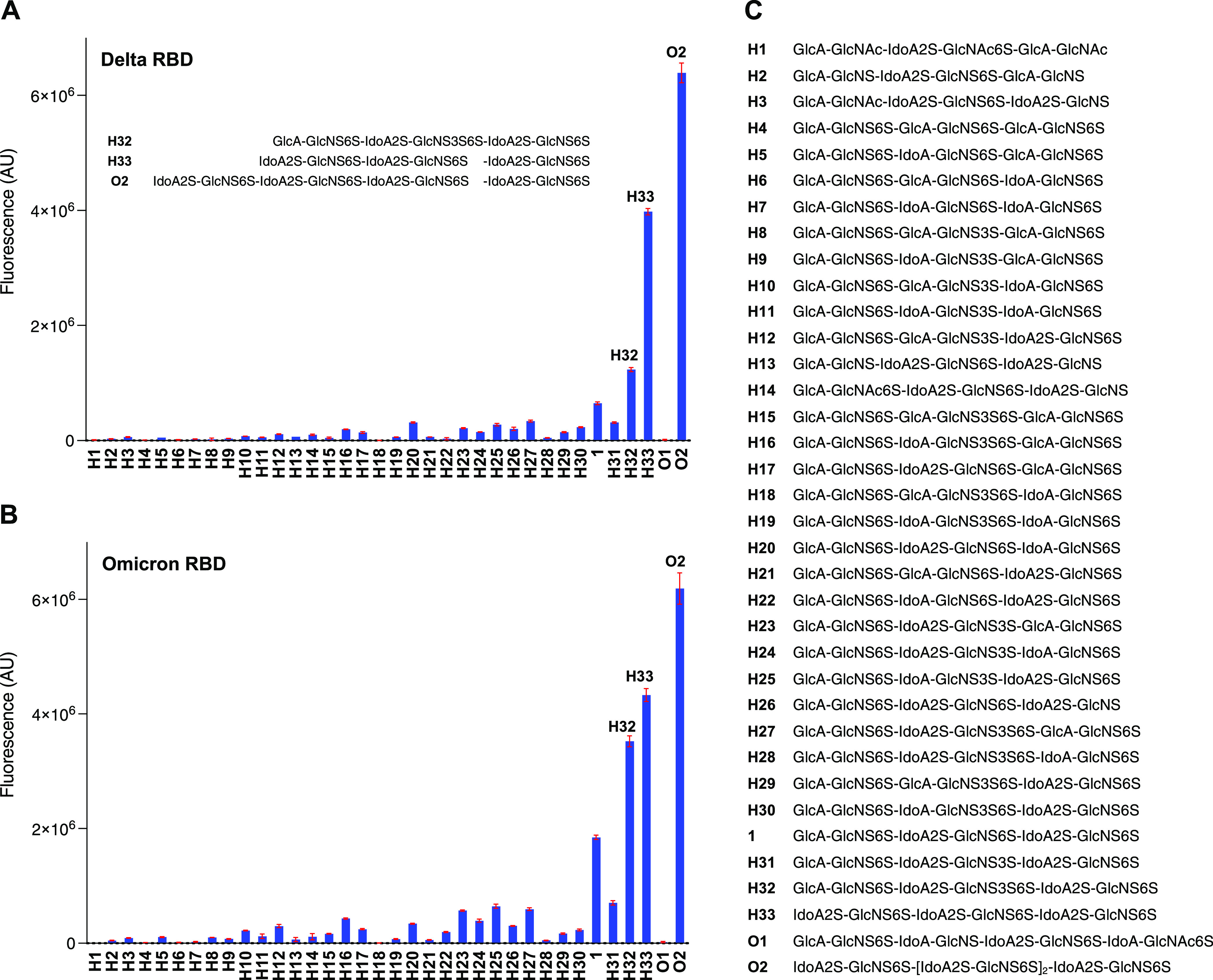
Microarray analysis of RBD of various SARS-CoVs shows similar binding selectivities to heparan sulfate. (A) Trimeric RBD protein of the Delta variant (20 μg/mL); structures of selected compounds are shown. (B) Trimeric RBD protein of the Omicron variant (20 μg/mL). (C) Compound numbering and structures (H, hexasaccharide; O, octasaccharide). All compounds have an anomeric linker; R = O(CH_2_)_5_NH_2_. Data are presented as mean ± SD (*n* = 4).

**Figure 5:**
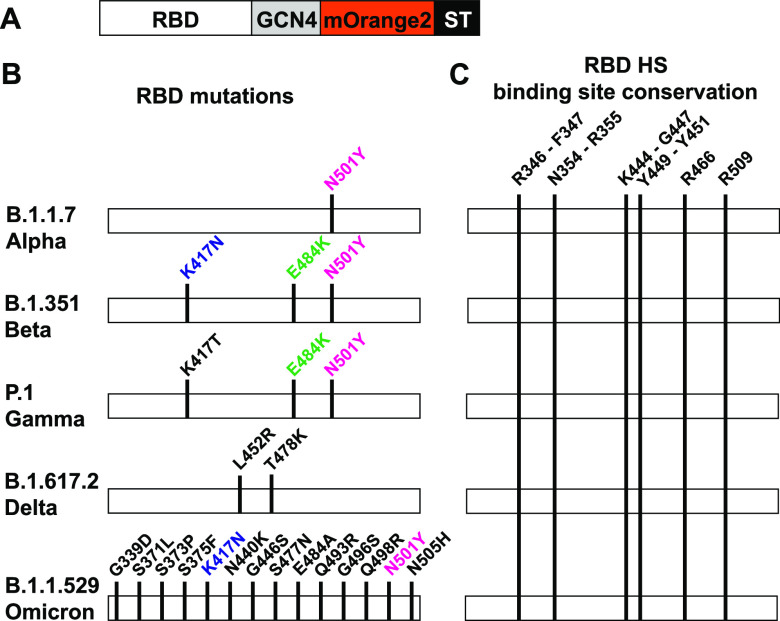
Sequence analysis of the putative HS-binding site of various SARS-CoV RBDs. (A) Schematic overview of the trimeric RBDs, which contain a C-terminal trimerization domain (GCN4) and is fused to mOrange2 (fluorescent reporter). The Strep-tag (ST) is used for purification purposes and detection. (B) Mutations that define each VoC, Alpha, Beta, Gamma, Delta, and Omicron. Common mutations in other VoC are indicated in blue, green, and pink. (C) Amino acids R346, F347, S349, N354, R355, K444, G447, Y449, Y451, R466, and R509 in the RBD, which are proposed to compose the HS-binding site, are conserved in SARS-CoV-2 Wuhan, Alpha, Beta, Gamma, Delta, and B.1.1.529 Omicron.

To examine whether SARS-CoVs have maintained their HS-binding selectivities during antigenic evolution, recombinant trimeric RBDs from SARS-CoV-1, SARS-CoV-2 Wuhan, Alpha, Beta, Gamma, Delta, and Omicron were exposed to subarrays, and after washing and drying, binding was visualized using an anti-His-tag antibody labeled with AlexaFluor647 (original SARS-CoV-2 RBD) or an anti-Strep-tag antibody labeled with StrepMAB-Classic Oyster 645 (Figures 4, 5A and S8). In each case, the same structure-binding relationship was observed, indicating that the VoCs have maintained similar HS-binding selectivities. The array contains a series of hexasaccharides modified by a 3-*O*-sulfate,^20^ which in each case was found not important for binding.

We performed an amino acid sequence alignment analysis of RBDs of a wide range of SARS-CoVs to examine whether the putative HS-binding site has been preserved during antigenic drift or shift. SARS-CoVs engages with human ACE2 in open conformation of the RBD. Structural and molecular modeling experiments have indicated that an exposed bind cleft composed of positively charged and hydrophobic amino acid (R346, F347, S349, N354, R355, K444, G447, Y449, Y451, R466, and R509) is positioned adjacent to the ACE2-binding site where HS can putatively bind.^4^ Although the RBDs of SARS-CoV-1, SARS-CoV-2 Wuhan, Alpha, Beta, Gamma, Delta, and Omicron have undergone mutational changes (Figure 5B), especially the Omicron variant, amino acids of the putative HS-binding site have been preserved,^29^ indicating they are important for infection (Figure 5C).

### Heparin Mimetics Exhibit Reduced Unfavorable Properties

The pleiotropic nature and promiscuous binding properties of heparin can result in adverse effects. In this respect, antithrombin-III (AT-III) is a key serine protease inhibitor (Serpin) for multiple proteases of the coagulation cascade including Factor-Xa and thrombin (Factor-IIa) and prevents blood clot formation.^30^ The conformationally active form of AT-III is generated only after binding to a specific 3-*O*-sulfation bearing pentasaccharide in heparin.^31^ Therefore, administration of heparin can be associated with bleeding. Furthermore, a complex of heparin with PF4 (also known as CXCL4) can result in heparin-induced thrombocytopenia (HIT), which is a life-threatening condition. PF4 is a chemokine that belongs to the CXC chemokine family that is released by activated platelets, and its binding to heparin induces coagulation by preventing interactions with blood coagulation factors. The heparin:PF4 complex can induce autoantibodies that, upon re-exposure to heparin, can cause HIT.

**Figure 6:**
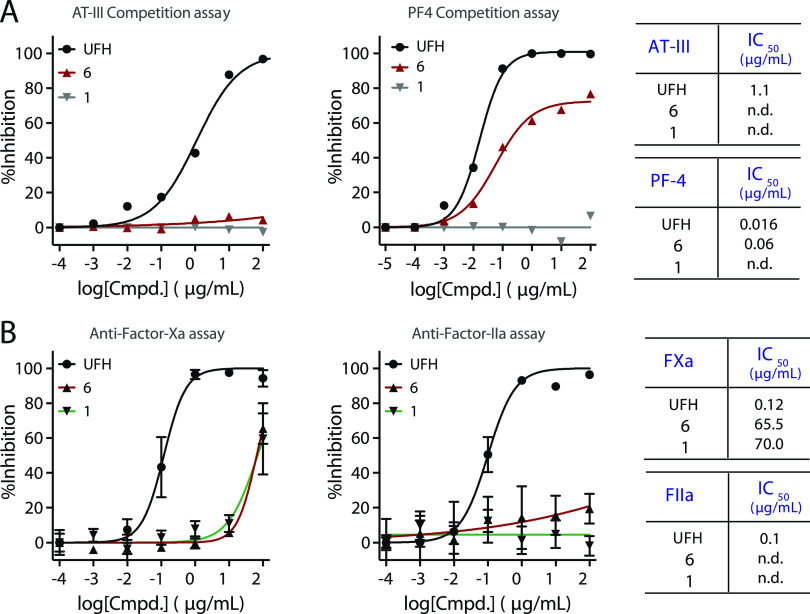
Evaluation of mimetics for their anticoagulation effects. (A) Inhibition curves for an SPR-based competition assay, binding of antithrombin-III (AT-III) and platelet factor 4 (PF4) to the heparin surface in the presence of heparin mimetics (**1** and **6**) or unfractionated heparin (UFH); half-maximal inhibitory concentration (IC_50_) values are presented in the summary table. For the individual inhibition curve and the SPR sensorgram, see Figures S10–S15. (B) Effects of **1**, **6**, and UFH on AT-III-mediated inhibition of Factor-Xa and Factor-IIa; overlay of inhibition curves and table of IC_50_ values are provided. Individual inhibition curves are presented in Supporting Figures S16 and S17.

A competition SPR method^32−34^ was employed to examine the binding of compounds **1** and **6** to AT-III and PF4, and the results were compared to similar properties of UFH. First, biotinylated heparin was immobilized on a streptavidin-coated sensor chip, which was employed to establish equilibrium dissociation constants (*K*_D_) of AT-III and PF4 by employing different concentrations of these proteins as analytes (Figure S9). In the case of AT-III, the resulting binding curves were fitted to a two-state binding model to obtain a *K*_D_ of 47.3 nM (Χ^2^ = 1.7). PF4 exhibited fast binding kinetics, and steady-state affinity analysis gave a *K*_D_ of 88.5 nM. The determined *K*_D_ values are in close agreement with literature reports.^34−36^ Next, inhibition experiments were performed by premixing different concentrations of monovalent **1**, tetravalent **6**, and UFH with AT-III or PF4 followed by exposure to the sensor chip modified by heparin. Both **1** and **6** exhibited no inhibitory potential toward AT-III, which was expected due to the absence of a 3-*O*-sulfate moiety (Figure 6A). On the other hand, UFH inhibited the binding of AT-III with an IC_50_ value of 1.1 μg/mL. Similar experiments with PF4 gave an IC_50_ value of 0.06 μg/mL for **6**, whereas no inhibition was observed for **1** (Figures 6A and S10–S15). UFH is, however, a more potent binder for PF4, and in this case, an IC_50_ value of 0.016 μg/mL was measured. Previous studies^37^ have indicated that a dodecasaccharide is required for the binding of PF4, which agrees with the observation that monovalent **1** does not exhibit inhibitory activity. The fact that tetravalent **6** does bind to PF4 supports the notion that it is a mimetic of heparin. It exhibits, however, a higher IC_50_ value, indicating it may be less prone to inducing HIT.

To validate the SPR results, AT-III-mediated anti-Factor-Xa and anti-Factor-IIa activities of the mimetics and UFH were measured using a colorimetric assay (Biophen Anti-Xa and Anti-IIa kits, two-stage chromogenic assays) (Figures 6B and S16–S17). As anticipated, UFH gave an IC_50_ value of 0.12 μg/mL for Factor-Xa and 0.1 μg/mL for Factor-IIa (Figure 6B). Compounds **6** and **1** only inhibited Factor-Xa with high IC_50_ values of 65.5 and 70 μg/mL, respectively (Figure 6B), and failed to invoke any inhibition of Factor-IIa. Collectively, these results show that multivalent mimetic **6** has more favorable properties compared to UFH.

The cytotoxicity of heparin mimetics **1** and **6** was evaluated on Vero E6 cells using the CellTox green cytotoxicity assay (G8741, Promega). It was discovered that compounds **1** and **6** at a concentration of 100 μg/mL exhibit no toxicity, and their properties are comparable to those of UFH at 25 μg/mL (see Figure S18).

## Conclusions

New variants of SARS-CoV-2 keep emerging, which necessitates the development of broad-acting therapeutics. Therapeutics may also reduce the prevalence of long-covid, which is associated with both hospitalized patients and those with mild or moderate disease. Earlier studies have demonstrated that SARS-CoV-2 employs HS for initial cell attachment and enzymatic removal of HS resulted in a very substantial reduction in cell binding and infectivity.^4,6^ These observations have generated interest in developing heparin as a therapeutic for SARS-CoV-2 infections.^5,15,16,38,39^ Its use is, however, complicated by the structural heterogeneity and the risk of causing bleeding and thrombocytopenia. Therefore, there is an urgent need to develop well-defined heparin mimetics that can potently inhibit viral cell binding with reduced side effects. In this study, we developed a heparin mimetic by the CuAAC-mediated assembly of an appropriately sulfated hexa- or heptasaccharide modified by an alkyne or azide moiety, respectively. It readily provided mono-, di-, tri-, and tetravalent heparin mimetics that were evaluated for their ability to inhibit the binding of spike or RBD of SARS-CoV-2 to immobilized heparin or Vero E6 cells. It was found that increasing the number of hexasaccharide repeating units resulted in large increases in the inhibitory potential and a tetrameric compound (**6**) had similar potency compared to UFH. The data indicate that multivalent interactions govern the binding between spike and HS and support a model in which the spike engages with the glycocalyx by low-affinity high-avidity interactions to travel through the glycocalyx to reach the cell membrane to engage with ACE2 for cell entry. Furthermore, it was found that a wide range of variants of concern have maintained HS-binding properties, indicating it is critical for infection and may function as an inhibitor for emerging viruses. The heparin mimetics are devoid of activation of AT-III and exhibit reduced binding of PF4, indicating they cause fewer side effects.

Multiple viruses employ HS as a receptor or coreceptor for cell attachment.^40−42^ It is the expectation that glycan microarray screening to identify monovalent binding partners combined with the strategy described here to prepare multivalent heparin mimetics will provide potent inhibitors. Furthermore, next-generation heparins have the potential to act as therapeutics for multiple diseases including inflammatory and neurological diseases, cancer, and wound healing.^43,44^ It is the expectation that the mimetics described here will facilitate harnessing the biomedical potential of heparin.

Synthetic oligosaccharides have been attached to polymers in a pendant manner to mimic the properties of heparin.^45,46^ Well-defined heparin and HS mimetics in which HS oligosaccharides are linked in a head-to-tail manner^23,47^ are expected to mimic more closely the structure of natural counterparts. It provided well-defined compounds including a compound composed of as many as 27 monosaccharides. The methodology makes it possible to examine in a systemic manner the optimal length for binding. Very recently, another approach was reported for head-to-tail coupling of HS-like saccharide building blocks^47^ by amide coupling of disaccharides that have amino- and carboxylic acid-containing linkers at the anomeric center and C-4 of the nonreducing end, respectively. The goal was to mimic compounds that resemble relatively short HS fragments, and it was found that a pseudo-hexasaccharide mimetic can bind to FGF2 with a similar affinity as a natural hexasaccharide. The chemoenzymatic approach employed here is particularly well suited to prepare polysaccharide mimetics that engage with their targets in a multivalent manner. One of its attractive features is that it employs HS oligosaccharides that can readily be prepared by previously reported methodologies^17,19,20^ and then modified by an azido-containing monosaccharide by an easy-to-perform enzymatic transformation. The resulting compounds can then be assembled into larger structures by robust CuAAC.

## Supporting Information Available

Synthetic protocols, compound characterization, detailed SPR data, cell experimental, and NMR spectra (PDF)
