# Supplementary material for: Well-Defined Heparin Mimetics Can Inhibit Binding of the Trimeric Spike of SARS-CoV-2 in a Length-Dependent Manner
Source: JACS Au. 2023 Apr 6;3(4):1185–95. doi: 10.1021/jacsau.3c00042 (PMC10089289; doi:10.1021/jacsau.3c00042)

Supporting Information

Well-Defined Heparin Mimetics Can Inhibit Binding of the Trimeric Spike of SARS-CoV-2 in a Length-Dependent Manner

Lifeng Sun,[1] Pradeep Chopra,[2] Ilhan Tomris,[1] Roosmarijn van der Woude,[1] Lin Liu,[2] Robert P. de Vries,[1] and Geert-Jan Boons[1,2,3,4] *

[1] Department of Chemical Biology and Drug Discovery, Utrecht Institute for Pharmaceutical Sciences, Utrecht University, 3584 CG Utrecht, The Netherlands

[2] Complex Carbohydrate Research Center, The University of Georgia, Athens, Georgia 30602, United States

[3] Bijvoet Center for Biomolecular Research, Utrecht University, 3584 CG Utrecht, The Netherlands

[4] Chemistry Department, The University of Georgia, Athens, Georgia 30602, United States

Corresponding author email: g.j.p.h.boons@uu.nl and gjboons@ccrc.uga.edu

Table of Contents

1. Experimental procedures S4

General experimental procedures. S4

Figure S1. Monosaccharide nomenclature system for NMR assignments. S5

Scheme S1. Synthesis of hexasaccharide precursor.[a] S6

Figure S2. Analysis of synthetic compounds by NMR spectroscopy. S7

General procedure for *O*-sulfation. S8

General procedure for saponification of methyl esters and de-*O*-acetylation. S8

General procedure for the reduction of azide group. S9

General procedure for selective *N*-sulfation reaction. S9

General procedure for global deprotection. S9

General procedure for copper (I)-catalyzed azide-alkyne cycloaddition (CuAAC) reaction. S11

2. Experimental procedures and analytical data of synthetic compounds S11

3. Materials for SPR and Vero E6 inhibition experiments S37

4. Surface plasma resonance (SPR) experiments S37

Preparation of heparin chip S37

SARS-CoV-2 spike protein competition assays S37

Figure S3. SARS-CoV-2 spike protein competition SPR data of compound 1. ..... S38

Figure S4. SARS-CoV-2 spike protein competition SPR data of compound 4. ..... S39

Figure S5. SARS-CoV-2 spike protein competition SPR data of compound 5. ..... S39

Figure S6. SARS-CoV-2 spike protein competition SPR data of compound 6. ..... S40

Figure S7. SARS-CoV-2 spike protein competition SPR data of UFH. S40

5. Microarray printing and screening S41

Figure S8. Binding analysis of synthetic HS oligosaccharides to SARS-CoV-2 related proteins by microarray. S43

6. Coagulation-related proteins SPR binding and competition studies S44

Coagulation-related proteins SPR binding studies S44

Coagulation-related proteins SPR competition assays S44

Figure S9. Surface Plasmon Resonance (SPR) binding analysis of Antithrombin-III (AT-III) and Platelet factor-4 (PF-4) to heparin chip. S45

Figure S10. AT-III competition SPR data of compound 1. .... S46

Figure S11. AT-III competition SPR data of compound 6. .... S46

Figure S12. AT-III competition SPR data of unfractionated heparin (UFH)................ S47

Figure S13. PF-4 competition SPR data of compound 1... S47

Figure S14. PF-4 competition SPR data of compound 6... S48

Figure S15. PF-4 competition SPR data of unfractionated heparin (UFH).................. S48

7. Anticoagulant assays S49

AT-III mediated Factor-IIa inhibition assays.. S49

AT-III mediated Factor-Xa inhibition assays S49

Figure S16. Anticoagulant activity of heparin mimetics was determined by utilizing AT-III mediated Factor-IIa inhibition assays.. S50

Figure S17. Anticoagulant activity of heparin mimetics was determined by utilizing AT-III mediated Factor-Xa inhibition assays... S51

8. Vero E6 cell inhibition experiments... S51

Figure S18. Cytotoxicity assay on Vero E6 cells... S51

9. References S54

10. NMR and ESI-MS spectra S56

1. Experimental procedures

General experimental procedures. All chemical reagents were purchased from commercial sources and used without further purification unless otherwise noted. Molecular sieves (4 Å) were flame-dried prior to use. All moisture-sensitive reactions were carried out under an argon atmosphere. All reactions ate elevated temperatures were performed in silicon oil bath unless otherwise specified. Reactions were monitored by thin-layer chromatography (TLC) on silica gel-coated aluminum or glass plates (EMD Chemicals Inc.). Spots were visualized by UV light (254 nm) when applicable and charring with 10 % sulfuric acid in ethanol or a solution of $(NH_4)_6Mo_7O_{24}\cdot 4H_2O$ (24.0 g, 19.4 mmol) and $Ce(NH_4)_2(NO_3)_6$ (0.50 g, 0.9 mmol) in sulfuric acid (5%, 500 mL). Column chromatography was performed on silica gel G60 (Silicycle 60 – 200 μm, 60 Å). Fractions or reaction mixture containing sulfated compound were concentrated under reduced pressure with water bath temperature no more than 25 °C. NMR spectra (^{1}H, ^{13}C, COSY, HSQC, TOCSY, HMBC, NOESY) were recorded on an Agilent 400-MR DD2 or Bruker AVANCE-600 MHz spectrometer at 25 °C. Chemical shifts are reported in parts per million (ppm) relative to tetramethylsilane (TMS δ = 0 ppm) or deuterium oxide (D_2O δ = 4.79 ppm) as the internal standard. NMR data is presented as follows: chemical shift, multiplicity (s = singlet, bs = broad singlet, d = doublet, bd = broad doublet, t = triplet, dd = doublet of doublet, m = multiplet and/or multiple resonances), q = quartet, integration, coupling constant in Hertz (Hz). NMR signals were assigned on the basis of ^{1}H NMR, ^{13}C NMR, COSY, HSQC, HMBC, TOCSY or NOESY experiments. Due to the small sample size, carbon chemical shifts for compounds **1-6**, **12** and **13** were collected from the F1 dimension in the HSQC spectra. Mass spectra were obtained on a Bruker micrOTOF-Q II (ESI-MS). High resolution masses were measured on an Agilent 6560 Ion Mobility Q-TOF LC-MS system. ^{1}H NMR spectroscopy was used to determine the yield of the final compounds, with acetone as an internal reference. *Pasteurella multocida* heparosan synthase 2 (PmHS2) were expressed in E. coli as previously described.[1]

Abbreviations: Ac, acetyl; AcOH, acetic acid; BAIB, (Diacetoxyiodo)benzene; Bn, benzyl; Bu, butyl; Cbz, benzyloxycabonyl; Bz, benzoyl; $CDCl_3$, deuterated chloroform; CMPI, 2-Chloro-1-methylpyridinium iodide; CSA, Camphorsulfonic acid; CuAAC, copper(I)-catalyzed alkyne-azide cycloaddition; DABCO, 1,4-Diazabicyclo[2.2.2]octane; DCC, *N*,*N*'-Dicyclohexylcarbodiimide; DCM, dichloromethane; DMF, dimethyl formamide; D_2O, deuterated water; Et, ethyl; Et_3N, triethylamine; EtOAc, ethyl acetate; EtOH, ethanol; ESI-

TOF, electrospray ionizaiton – time of flight; Fmoc, fluorenylmethoxycarbonyl; GlcNAc, *N*-acetyl-D-glucosamine; H_2O, water; H_2O_2, hydrogen peroxide; HRMS, high resolution mass spectrometry; Lev, levulinoyl; LiOH, lithium hydroxide; Me, methyl; MeCN, acetonitrile; MeOH, methanol; $MgSO_4$, magnesium sulfate; $MnCl_2$, manganese dichloride; N_3, azide; $NaHCO_3$, sodium bicarbonate; NaOH, sodium hydroxide; NIS, *N*-Iodosuccinimide; NHS, *N*-Hydroxysuccinimide; $Pd(OH)_2/C$, palladium hydroxide over carbon; Piv, pivaloyl; PmHS2, *Pasteurella multocida* heparosan synthase; TEMPO, 2,2,6,6-Tetramethylpiperidine 1-oxyl; $SO_3{\cdot}Py$, sulfur trioxide/pyridine complex; SPR, surface plasma resonance; TBAF, tetrabutylammonium fluoride; TDS, dimethyl thexylsilyl; TFA, trifluoroacid; TfOH, trifluoromethanesulfonic acid; THF, tetrahydrofuran; THPTA, Tris(3-hydroxypropyltriazolylmethyl)amine; TLC, thin layer chromatography; $TMSCHN_2$, (Trimethylsilyl)diazomethane; TMSOTf, Trimethylsilyl trifluoromethanesulfonate; Tol, toluene; Tris, Tris(hydroxymethyl)aminomethane; UDP, Uridine diphosphate; v/v, volume/volume.

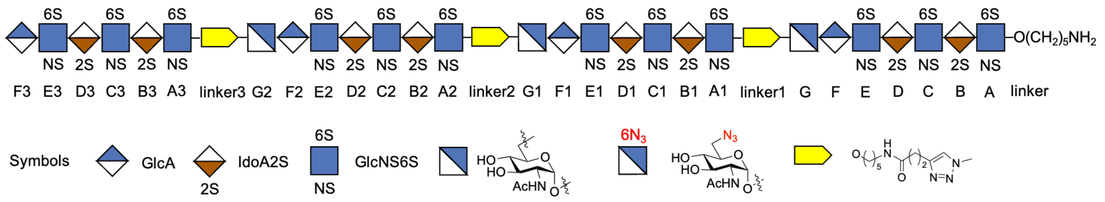

Figure S1. Monosaccharide nomenclature system for NMR assignments. For oligosaccharide nomenclature for NMR spectroscopy, the monosaccharide residues of the HS oligosaccharides have been labelled from the reducing end to non-reducing end in an alphabetical order (A, B, C, D, E, F or G) for each domain and labelled together with 1, 2, 3 for the repeated domains (*e.g.* compound **6** labelled as above).

Scheme S1. Synthesis of hexasaccharide precursor.[a]

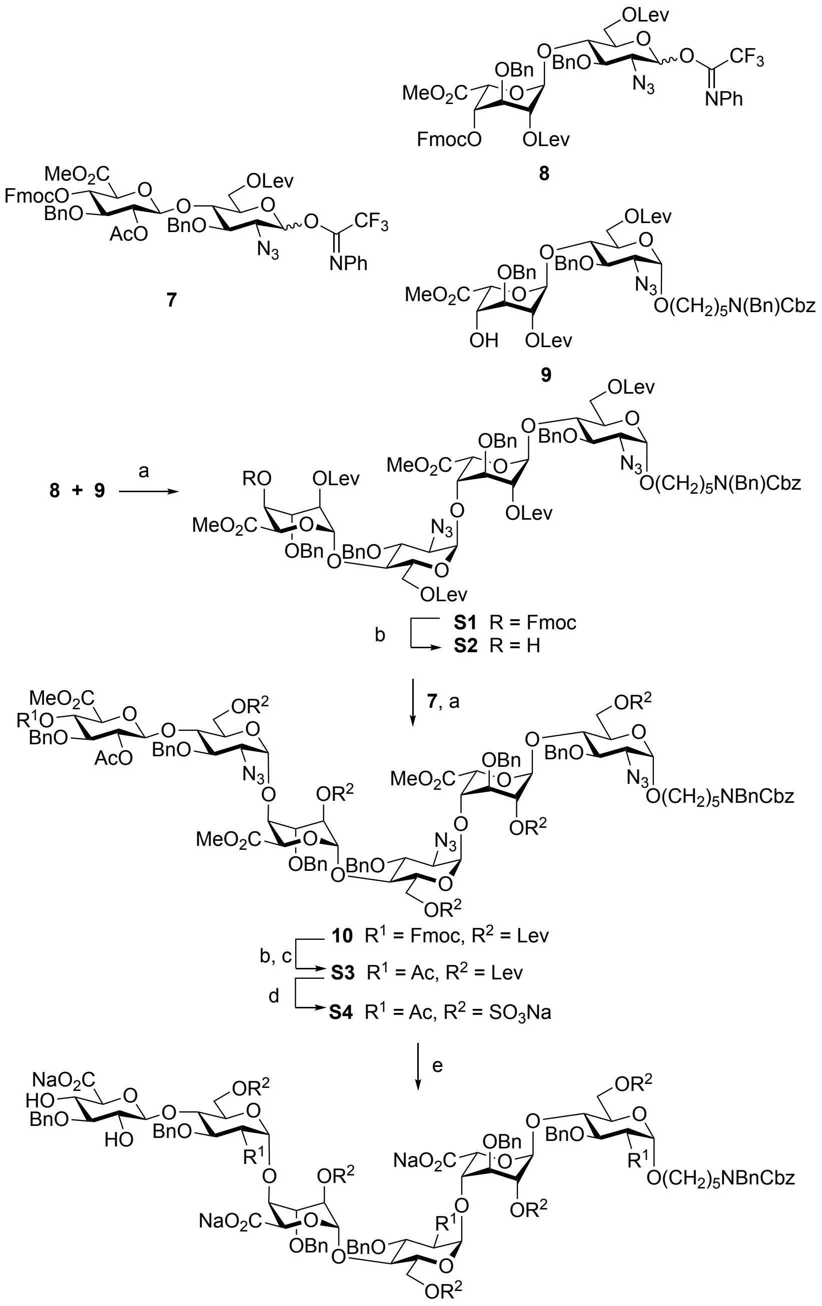

[a]Reagents and conditions: (a) TfOH, DCM, 4Å MS, -40 °C, 1 h, **S1**: 56%; **10**: 71%; (b) DCM/Et_3N (4/1), 2 h; **S2**: 90%; (c) Ac_2O, Py., 3h; (d) NH_2NH_2·AcOH, Toluene/EtOH (1/2), 2 h; SO_3·Py, DMF, 2 h; (e) 1.0 M LiOH, 30% H_2O_2, THF/H_2O (1/1); (f) 1.0 M PMe_3 in THF, 0.1 N NaOH, 2 h; (g) SO_3·Py, MeOH, Et_3N, 0.1 N NaOH (pH = 11); **11**: 22% over seven steps.

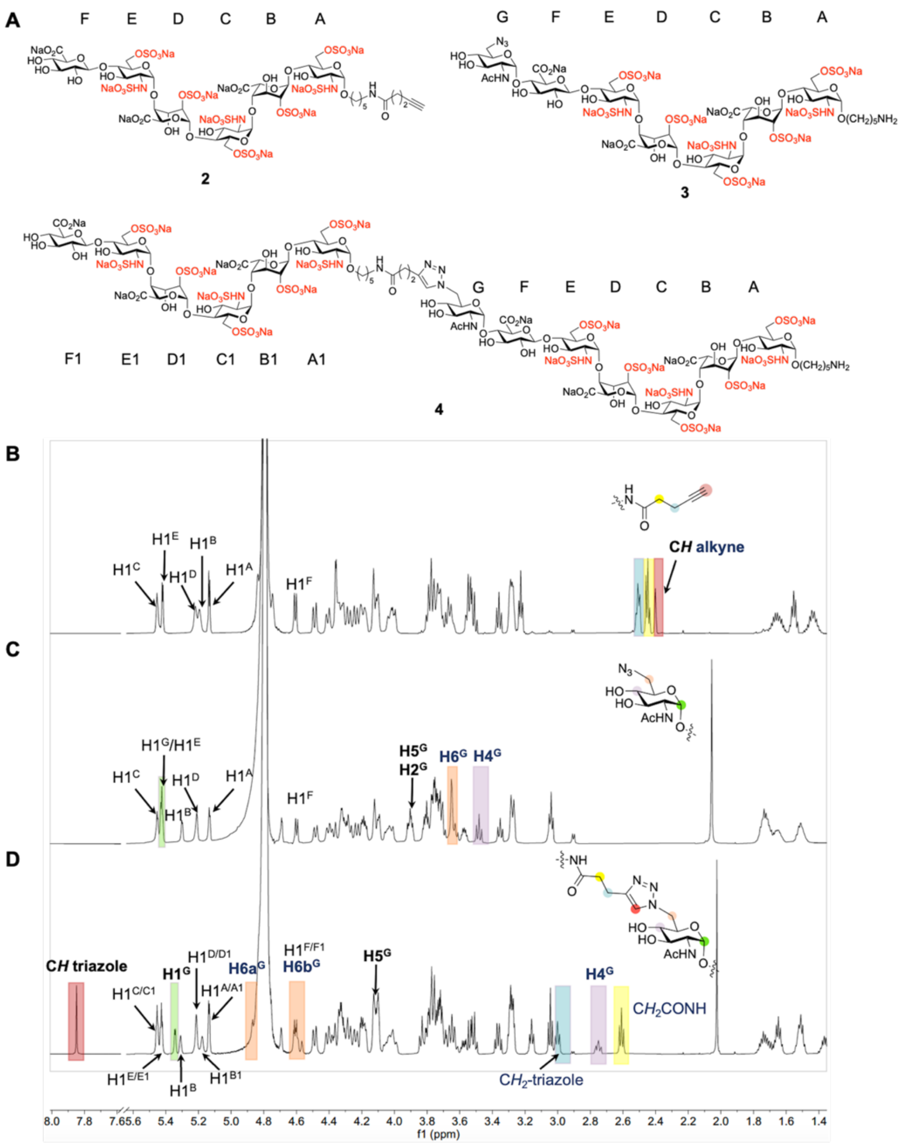

Figure S2. Analysis of synthetic compounds by NMR spectroscopy. Stacked plots showing ^{1}H NMR and structures of compounds **2**, **3**, and CuAAC product **4**. (A) Structures of compounds **2**, **3** and **4**, sugar rings are labelled in an alphabetical order starting from reducing end to non-reducing end. (B) ^{1}H NMR of hexasaccharide **2** where anomeric linker is extended with alkyne functionality; characteristic protons are annotated. (C) ^{1}H NMR of compound **3** with terminal GlcNAc6N$_3$; characteristic protons are annotated. (D) ^{1}H NMR of CuAAC product of **2** and **3**, heparin mimetic **4**. Blue highlighted area, presence of CH_2 that is attached to alkyne of compound 2 and presence of CH_2 that is attached to triazole of compound **4**. Yellow highlighted area, presence of CH_2 that is highlighted in the structure of compound **2** and **4**. Red highlighted area, presence of CH alkyne of compound **2** and presence of CH triazole of compound **4**. Orange highlighted area, presence of H6 of GlcNAc6N$_3$ of compound **3** and presence of H6 of sugar with triazole of compound **4**. Purple highlighted area, presence of H4 of GlcNAc6N$_3$ of compound **3** and presence of H4 of sugar attached to triazole of compound **4**. Green highlighted area, presence of H1 of GlcNAc6N$_3$ of compound **3** and presence of H1 of sugar attached triazole of compound **4**.

General procedure for Lev esters removal. Hydrazine acetate (5 equiv. per Lev group) was added to a solution of the starting material in a mixture of DCM and MeOH (1/1, v/v, 0.02 M). The reaction mixture was stirred at room temperature for 2 to 4 h until TLC analysis (petroleum ether/EtOAc, 1/1 to 1/2, v/v) indicated completion of the reaction. The reaction mixture was diluted with DCM (30 mL), washed with water (25 mL), saturated bicarbonate solution (2 × 25 mL) and brine (25 mL), dried (Na_2SO_4) and filtered. The filtrate was concentrated under reduced pressure, and the residue was purified by silica gel column chromatography using petroleum ether and EtOAc as eluent (1/1 to 1/2, v/v), to give pure product.

General procedure for *O*-sulfation. To a solution of the starting material in DMF (0.15 M) was added SO_3·Py (10 equiv. per OH). The reaction mixture was stirred at room temperature for 2 to 4 h until TLC (DCM/MeOH, 90/10, v/v) indicated completion of the reaction. A mixture of triethylamine and MeOH (1/1, v/v, 1 mL) was added to the reaction mixture and stirring was continued for another 30 min. The reaction mixture was concentrated under reduced pressure, and the residue was applied to a column of Iatrobeads (5 g), which was eluted with a gradient of DCM and MeOH (from 95/5 to 85/15, v/v). Fractions containing product were concentrated under reduced pressure and the residue was passed through a column of Dowex® 50 × 8 Na^+ resin (0.6 cm × 5 cm) using MeOH as eluent, to give pure product.

General procedure for saponification of methyl esters and de-*O*-acetylation. A premixed solution of 30% solution of H_2O_2 in H_2O (100 equiv. per CO_2Me) and 1.0 M LiOH (50 equiv. per CO_2Me) was added to a solution of the starting material in THF (0.02 M). The reaction mixture was stirred at room temperature for 12 h. Then, a 4.0 M solution of NaOH was added until pH ~ 14. Stirring was continued until LC-MS indicated completion of the reaction. After adjusting the pH to 8-9 by careful addition of AcOH, the solvents were removed under reduced pressure. The residue was dissolved in water and applied to a Reverse Phase C18 column (1.0 cm × 5 cm), which was eluted with a gradient of H_2O and MeOH (from 90/10 to 40/60, v/v). Fractions containing product were concentrated under reduced pressure and the residue was

passed through a column of Dowex® 50 × 8 Na^+ resin (0.6 cm × 5 cm) using H_2O as eluent to give pure product.

General procedure for the reduction of azide group. To a solution of the starting material in THF (1.0 mL for 0.013 mmol) was added 0.1 M NaOH (10 equiv. per azido group). Then, 1.0 M solution of PMe_3 in THF (8 equiv. per azido group) was added to the solution. The reaction mixture was stirred at room temperature for 1 h until LC-MS indicated completion of the reaction. After adjusting the pH to 8-9 by careful addition of AcOH, the solvents were removed under reduced pressure. The residue was dissolved in water and applied to a Reverse Phase C18 column (1.0 cm × 5 cm), which was eluted with a gradient of H_2O and MeOH (from 90/10 to 40/60, v/v). Fractions containing product were concentrated under reduced pressure and the residue was passed through a column of Dowex® 50 × 8 Na^+ resin (0.6 cm × 5 cm) using H_2O as eluent, to give the desired product.

General procedure for selective *N*-sulfation reaction. SO_3·Py (5 equiv. per NH_2) was added to the solution of starting material in a mixture of MeOH (1 mL for 0.006 mmol) and Et_3N (0.3 mL). 1 M NaOH was added to adjust the pH to 11. Three additional portions of SO_3·Py (5 equiv. per NH_2) was added to the solution after 30 min, 1 h, 2 h followed by adjusting the pH to 11 by careful addition of 1 M NaOH respectively. The progress of the reaction was monitored by TLC (silica gel TLC, EtOAc/Pyridine/H_2O/AcOH, 8/5/3/1, v/v/v/v). After stirring for an additional 12 h, the reaction mixture was co-evaporated with water and the residue passed through a short column of Dowex® 50 × 8Na^+ resin (1.0 cm × 5 cm) with H_2O as eluent. Fractions containing product were lyophilized, and the residue was dissolved in water and applied to Reverse Phase C18 silica gel column (1.0 cm × 5 cm), which was eluted with a gradient of H_2O and acetonitrile (from 98/2 to 85/15, v/v). Appropriate fractions were lyophilized to give the desired product.

General procedure for global deprotection. Palladium hydroxide on carbon (Degussa type, 20%, 1.5 times the weight of starting material) was added to a solution of the starting material in *tert*-butanol and H_2O (1/1, v/v, 1 mL for 1 mg). The mixture was placed under an atmosphere

of hydrogen until completion of the reaction as indicated by ESI-MS. The mixture was filtered through a PTFE syringe filter (Acrodisc®, 0.2 μm), and the residue was washed with *tert*-butanol and H_2O mixture (1/1, v/v, 2 mL). The filtrate was lyophilized to give the final product. Biogel P2 (1.5 cm × 50 cm) was used for purification with 0.1 M ammonium bicarbonate as eluent, the fractions containing compound were lyophilized and passed through a short column of Dowex® 50 × 8Na^+ resin (1.0 cm × 5 cm) with H_2O as eluent. Appropriate fractions were lyophilized to give the desired product.

General procedure for installation of alkyne moiety. Compounds containing linker free amine (1.0 equiv.) were dissolved in aqueous 0.3 M $NaHCO_3$ (29 μL/μmol compound) and MeOH (6 μL/μmol compound), followed by addition of a solution of *N*-succinimido 4-pentynonate (3.0 equiv) in acetonitrile (29 μL/μmol compound). The reaction mixture was stirred at room temperature for 3 h. Bio-Gel P-6 (1.5 cm × 100 cm) was used for purification with 100 mM ammonium bicarbonate as eluent. The fractions containing compound were lyophilized and passed through a short column of Dowex® 50 × 8Na^+ resin (1.0 cm × 2 cm) with H_2O as eluent. Appropriate fractions were lyophilized to give the desired product.

General procedures for installation of unnatural α (1→4) 6-azido-GlcNAc. HS glycan (1.0 equiv.) and UDP 6-azido-GlcNAc (1.5 equiv.) were dissolved in Tris buffer (25 mM, pH = 7.5) containing $MnCl_2$ (5 mM). *Pasteurella multocida* heparosan synthase (PmHS2) (final concentration of enzyme 200 μg/mL) was added to form a final concentration of 3.2 mM. The reaction mixture was incubated at 37 °C for 12 h. The progress of the reaction was monitored by ESI-LC-MS, on completion the reaction mixture was lyophilized and the residue was passed through Biogel P6 (1.5 cm × 50 cm or 1.5 cm × 120 cm) with 0.1 M ammonium bicarbonate as eluent. Appropriate fractions as indicated by ESI-MS were lyophilized and passed through a short column of Dowex® 50 × 8Na^+ resin (1.0 cm × 2 cm) with H_2O as eluent. Appropriate fractions were lyophilized to give the desired product.

General procedure for copper (I)-catalyzed azide-alkyne cycloaddition (CuAAC) reaction. Compounds containing azide group (1.0 equiv.) and compounds containing alkyne group (1.2 equiv.) were dissolved in 0.1 M ammonium bicarbonate solution (0.4 mM for compounds containing azido) in a 0.5 mL or 1.5 mL centrifugal tube to form solution **A**. A premixed solution of $CuSO_4$ (1.25 equiv.) and THPTA (6.25 equiv.) were added to the solution **A** followed by the addition of aminoguanidine solution (final concentration 10 mM). After adding sodium ascorbate solution (freshly made 20 mg/mL, final concentration 10 mM), the reaction mixture was mixed well, securely closed to prevent more oxygen from diffusing in. The reaction mixture was in 37 °C incubator for 24 h. After lyophilization, the residue was dissolved in 0.1 M ammonium bicarbonate and applied to Biogel P6 (1.5 cm × 120 cm), which was eluted with 0.1 M ammonium bicarbonate. Appropriate fractions as indicated by ESI-MS were lyophilized and passed through a short column of Dowex® 50 × $8Na^+$ resin (1.0 cm × 2 cm) with H_2O as eluent. Appropriate fractions were lyophilized to give the desired product. All the reactions were performed at a small scale (no more than 1.5 mL scale).

2. Experimental procedures and analytical data of synthetic compounds

***N*-(Benzyl)-benzyloxycarbonyl-5-aminopentyl *O*-[methyl-2-*O*-levulinoyl-3-*O*-benzyl-4-*O*-(9-fluorenylmethyloxycarbonyl)-α-L-idopyranosyluronate]-(1→4)-*O*-(2-azido-3-*O*-benzyl-6-*O*-levulinoyl-2-deoxy-α-D-glucopyranosyl)-(1→4)-*O*-(methyl-2-*O*-levulinoyl-3-*O*-benzyl-α-L-idopyranosyluronate)-(1→4)-*O*-(2-azido-3-*O*-benzyl-6-*O*-levulinoyl-2-deoxy-α-D-glucopyranoside (S1).** Compound **S1** was prepared as illustrated in **Scheme S1**.

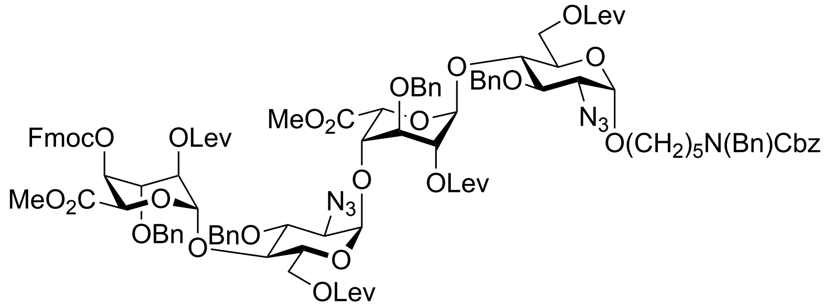

Freshly activated 4 Å molecular sieves was added to a solution of glycosyl donor **8**[2,3] (541 mg, 0.464 mmol) and glycosyl acceptor **9**[2,3] (418 mg, 0.387 mmol) in anhydrous DCM (9.3 mL). After stirring for 30 min at room temperature, the solution was cooled to -40 °C followed by the addition of TfOH (17 μL,

0.193 mmol). The reaction mixture was stirred for 1 h followed by quenching with pyridine. The reaction mixture was filtered, and the filtrate was concentrated *in vacuo*. The residue was purified by silica gel column chromatography using a stepwise gradient of petroleum ether and EtOAc (2/1 to 1/2, v/v) as the eluent to give compound **S1** (445 mg, 56%). R_f = 0.50 (petroleum ether/EtOAc, 1/1.5, v/v). ^{1}H NMR (600 MHz, $CDCl_3$) δ 7.80 – 7.12 (m, 38H, CH Aromatic), 5.21 (d, *J* = 3.7 Hz, 1H, $H1^B$), 5.17 (bd, *J* = 20.2 Hz, 2H, $C\mathit{H}_2$Cbz), 5.06 (d, *J* = 2.7 Hz, 1H, $H1^D$), 5.05 (d, *J* = 3.6 Hz, 1H, $H1^C$), 4.97 – 4.93 (m, 2H, $H4^D$, $H2^B$), 4.87 (d, *J* = 3.3 Hz, 1H, $H5^D$), 4.85 – 4.79 (m, 3H, $H2^D$, $H1^A$, C*H*HBn), 4.78 – 4.67 (m, 6H, $H5^B$, 2 × $C\mathit{H}_2$Bn, C*H*HBn), 4.64 (d, *J* = 10.8 Hz, 1H, C*H*HBn), 4.60 (d, *J* = 10.7 Hz, 1H, C*H*HBn), 4.53 – 4.36 (m, 6H, $NC\mathit{H}_2$Bn, $H6a^C$, $H6a^A$, $C\mathit{H}_2$ Fmoc), 4.26 (d, *J* = 12.0 Hz, 1H, $H6b^A$), 4.21 (t, *J* = 7.2 Hz, 1H, C*H* Fmoc), 4.16 (dd, *J* = 12.1, 2.8 Hz, 1H, $H6a^C$), 4.01 (t, *J* = 4.5 Hz, 1H, $H4^B$), 3.96 – 3.79 (m, 7H, $H3^B$, $H4^C$, $H5^A$, $H5^C$, $H4^A$, $H3^D$, $H3^A$), 3.68 – 3.59 (m, 2H, $H3^C$, OCH*H* linker), 3.47 (s, 3H, $CO_2C\mathit{H}_3$), 3.44 (s, 3H, $CO_2C\mathit{H}_3$), 3.44 – 3.16 (m, 5H, OCH*H* linker, $H2^A$, $H2^C$, $C\mathit{H}_2N$ linker), 2.86 – 2.46 (m, 16H, 8 × $C\mathit{H}_2$ Lev), 2.17 (s, 6H, 2 × $C\mathit{H}_3$ Lev), 2.11 (s, 3H, $C\mathit{H}_3$ Lev), 2.04 (s, 3H, $C\mathit{H}_3$ Lev), 1.70 – 1.20 (m, 6H, 3 × $C\mathit{H}_2$ linker). ^{13}C NMR (151 MHz, $CDCl_3$) δ 207.0 ($CH_3\underline{C}O$ Lev), 206.7 ($CH_3\underline{C}O$ Lev), 206.4 ($CH_3\underline{C}O$ Lev), 206.3 ($CH_3\underline{C}O$ Lev), 172.5 ($CH_2\underline{C}O$ Lev), 172.3 ($CH_2\underline{C}O$ Lev), 172.3 ($CH_2\underline{C}O$ Lev), 171.8 ($CH_2\underline{C}O$ Lev), 169.5 ($\underline{C}O_2CH_3$), 168.6 ($\underline{C}O_2CH_3$), 154.4, 143.3, 141.4, 138.2, 138.1, 137.7, 137.6, 137.3, 128.7, 128.6, 128.4, 128.3, 128.1, 128.0, 127.6, 127.4, 127.3, 125.2, 125.1, 120.2, 98.0 ($C1^B$), 97.7 ($C1^A$), 97.4 ($C1^D$), 96.8 ($C1^C$), 78.4 ($C3^A$), 78.2 ($C3^C$), 75.7 ($C4^A$), 74.9 ($O\underline{C}H_2$Bn), 74.8 ($O\underline{C}H_2$Bn), 74.3 ($C4^C$), 73.5 ($C3^B$, $C3^D$), 73.4 (2 × $O\underline{C}H_2$Bn), 71.9 ($C4^B$), 71.5 ($C4^D$), 70.3 ($\underline{C}H_2$ Fmoc), 69.7 ($C5^C$), 69.5 ($C5^B$), 69.4 ($C2^B$), 69.1 ($C5^A$), 68.2 ($O\underline{C}H_2$ linker, $C2^D$), 67.3 ($\underline{C}H_2$Cbz), 67.2 ($C5^D$), 63.4 ($C2^A$, $C2^C$), 62.6 ($C6^A$), 62.1 ($C6^C$), 52.3 ($CO_2\underline{C}H_3$), 51.9 ($CO_2\underline{C}H_3$), 50.7 ($N\underline{C}H_2$Bn), 50.4 ($N\underline{C}H_2$Bn), 47.2 ($\underline{C}H_2N$ linker), 46.8 ($\underline{C}$H Fmoc), 46.3 ($\underline{C}H_2N$ linker), 38.1 ($\underline{C}H_2$ Lev), 38.1 ($\underline{C}H_2$ Lev), 37.9 ($\underline{C}H_2$ Lev), 37.8 ($\underline{C}H_2$ Lev), 30.0 (3 × $\underline{C}H_3$ Lev), 29.8 ($\underline{C}H_3$ Lev), 29.6 ($\underline{C}H_3$ Lev), 29.2 ($\underline{C}H_2$ linker), 28.3 ($\underline{C}H_2$ Lev), 28.1 ($\underline{C}H_2$ Lev, $\underline{C}H_2$ linker), 28.0 ($\underline{C}H_2$ Lev), 27.8 ($\underline{C}H_2$ Lev), 23.5 ($\underline{C}H_2$ linker). HRMS (ESI-MS) (pos): m/z calculated for $C_{109}H_{125}N_8O_{33}$ $[M+NH_4]^+$: 2074.8378; found: 2074.8336.

***N*-(Benzyl)-benzyloxycarbonyl-5-aminopentyl *O*-[methyl-2-*O*-levulinoyl-3-*O*-benzyl-α-L-idopyranosyluronate]-(1→4)-*O*-(2-azido-3-*O*-benzyl-6-*O*-levulinoyl-2-deoxy-α-D-glucopyranosyl)-(1→4)-*O*-(methyl-2-*O*-levulinoyl-3-*O*-benzyl-α-L-idopyranosyluronate)-(1→4)-*O*-(2-azido-3-*O*-benzyl-6-*O*-levulinoyl-2-deoxy-α-D-glucopyranoside (S2).** Compound **S2** was prepared as illustrated in **Scheme S1**. Compound

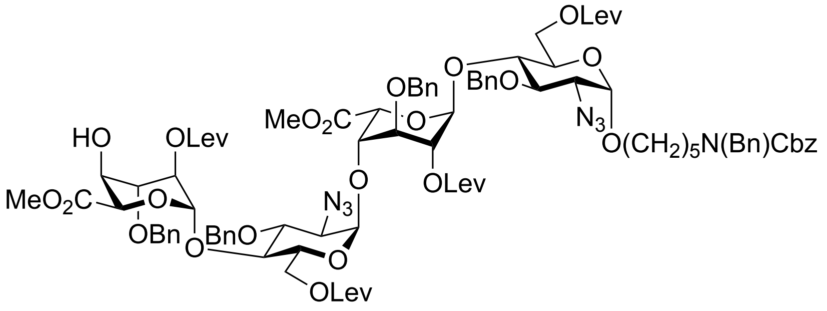

S1 (436 mg, 0.212 mmol) was dissolved in a mixture of DCM/Et_3N (18.7 mL, v/v, 4/1). The reaction mixture was stirred at room temperature for 2 h. After TLC analysis indicating completion of the reaction, the reaction mixture was concentrated under reduced pressure. The residue was purified by silica gel column chromatography using a stepwise gradient of petroleum ether and EtOAc (1/4 to 1/9, v/v) as the eluent to give compound **S2** (350 mg, 90%). R_f = 0.32 (petroleum ether/EtOAc, 1/4, v/v). ^{1}H NMR (600 MHz, $CDCl_3$) δ 7.42 – 7.07 (m, 30H, CH Aromatic), 5.24 – 5.12 (m, 3H, CH_2Cbz, $H1^B$), 5.04 (d, *J* = 3.6 Hz, 1H, $H1^C$), 4.99 (bs, 1H, $H1^D$), 4.96 – 4.93 (m, 1H, $H2^B$), 4.88 – 4.58 (m, 12H, $H5^D$, $H2^D$, $H1^A$, $H5^B$, 4 × CH_2Bn), 4.50 (bd, *J* = 11.2 Hz, 2H, NCH_2Bn), 4.44 (d, *J* = 12.0 Hz, 1H, $H6a^A$), 4.38 (dd, *J* = 12.5, 1.7 Hz, 1H, $H6a^C$), 4.25 (d, *J* = 12.0 Hz, 1H, $H6b^A$), 4.19 (dd, *J* = 12.5, 1.7 Hz, 1H, $H6b^C$), 4.02 – 3.59 (m, 11H, $H4^B$, $H4^D$, $H3^B$, $H4^C$, $H5^A$, $H4^A$, $H3^A$, $H5^C$, $H3^D$, $H3^C$, OCH*H* linker), 3.48 (s, 3H, CO_2CH_3), 3.43 (s, 3H, CO_2CH_3), 3.42 – 3.16 (m, 5H, OCH*H* linker, $H2^A$, $H2^C$, CH_2N linker), 2.79 – 2.52 (m, 16H, 8 × CH_2 Lev), 2.18 (s, 3H, CH_3 Lev), 2.17 (s, 3H, CH_3 Lev), 2.15 (s, 3H, CH_3 Lev), 2.11 (s, 3H, CH_3 Lev), 1.70 – 1.20 (m, 6H, 3 × CH_2 linker). ^{13}C NMR (151 MHz, $CDCl_3$) δ 206.9 ($CH_3\underline{C}O$ Lev), 206.7 ($CH_3\underline{C}O$ Lev), 206.6 ($CH_3\underline{C}O$ Lev), 206.3 ($CH_3\underline{C}O$ Lev), 172.5 ($CH_2\underline{C}O$ Lev), 172.34 ($CH_2\underline{C}O$ Lev), 172.28 ($CH_2\underline{C}O$ Lev), 171.5 ($CH_2\underline{C}O$ Lev), 169.7 ($\underline{C}O_2CH_3$), 169.5 ($\underline{C}O_2CH_3$), 154.4, 138.2, 138.1, 137.9, 137.5, 137.4, 128.7, 128.6, 128.3, 128.2, 128.1, 128.0, 127.9, 127.6, 127.5, 127.4, 98.1 ($C1^B$), 97.9 ($C1^D$), 97.7 ($C1^A$), 96.8 ($C1^C$), 78.4 ($C3^A$), 78.3 ($C3^C$), 75.6 ($C4^A$), 75.2 ($C3^D$), 74.9 ($O\underline{C}H_2$Bn), 74.6 ($O\underline{C}H_2$Bn), 74.4 ($C4^C$), 73.4 ($C3^B$), 72.8 (2 × $O\underline{C}H_2$Bn), 71.9

(C4^B), 69.7 (C5^C), 69.4 (C5^B), 69.1 (C2^B, C5^A), 69.0 (C2^D), 68.2 (O<u>C</u>H$_2$ linker, C5^D), 67.8 (C4^D), 67.3 (<u>C</u>H$_2$Cbz), 63.43 (C2^A), 63.40 (C2^C), 62.6 (C6^A), 62.2 (C6^C), 52.2 (CO$_2$<u>C</u>H$_3$), 51.9 (CO$_2$<u>C</u>H$_3$), 50.7 (N<u>C</u>H$_2$Bn), 50.4 (N<u>C</u>H$_2$Bn), 47.2 (<u>C</u>H$_2$N linker), 46.3 (<u>C</u>H$_2$N linker), 38.1 (<u>C</u>H$_2$ Lev), 39.0 (<u>C</u>H$_2$ Lev), 37.92 (<u>C</u>H$_2$ Lev), 37.87 (<u>C</u>H$_2$ Lev), 30.0 (2 × <u>C</u>H$_3$ Lev), 29.8 (2 × <u>C</u>H$_3$ Lev), 29.2 (<u>C</u>H$_2$ linker), 28.11 (<u>C</u>H$_2$ Lev, <u>C</u>H$_2$ linker), 28.07 (2 × <u>C</u>H$_2$ Lev), 27.8 (<u>C</u>H$_2$ Lev), 23.5 (<u>C</u>H$_2$ linker). HRMS (ESI-MS) (pos): m/z calculated for $C_{94}H_{115}N_8O_{31}$ [M+NH$_4$]$^+$: 1852.7697; found: 1852.7680.

***N*-(Benzyl)-benzyloxycarbonyl-5-aminopentyl *O*-[methyl-2-*O*-acetyl-3-*O*-benzyl-4-*O*-(9-fluorenylmethyloxycarbonyl)-β-D-glucopyranosyluronate]-(1→4)-*O*-(2-azido-3-*O*-benzyl-6-*O*-levulinoyl-2-deoxy-α-D-glucopyranosyl)-(1→4)-*O*-(methyl-2-*O*-levulinoyl-3-*O*-benzyl-α-L-idopyranosyluronate)-(1→4)-*O*-(2-azido-3-*O*-benzyl-6-*O*-levulinoyl-2-deoxy-α-D-glucopyranosyl)-(1→4)-*O*-(methyl-2-*O*-levulinoyl-3-*O*-benzyl-α-L-idopyranosyluronate)-(1→4)-*O*-(2-azido-3-*O*-benzy-6-*O*-levulinoyl-2-deoxy-α-D-glucopyranoside (10).**

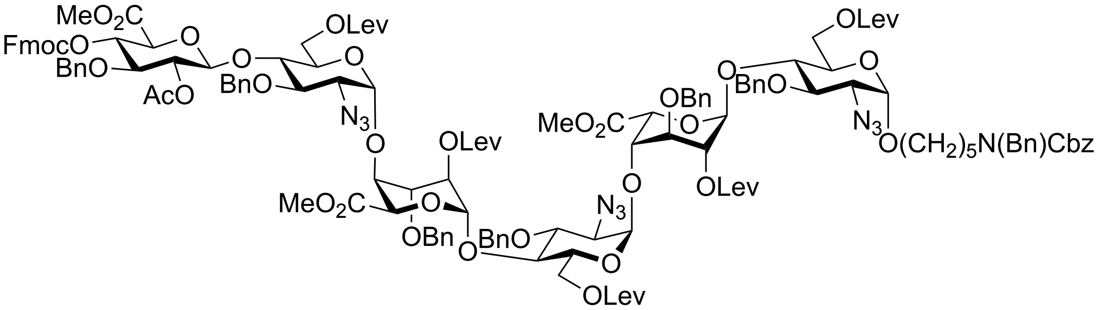

Compound **10** was prepared as illustrated in **Scheme S1**. To a solution of glycosyl donor **7**[2,3] (250 mg, 0.225 mmol) and gylcosyl acceptor **S2** (338 mg, 0.184 mmol) in anhydrous DCM (4.5 mL) was added freshly activated 4 Å molecular sieves (700 mg) after stirring for 30 min at room temperature and the solution was cooled to -40 °C followed by adding TfOH (8.1 μL, 0.092 mmol). The reaction mixture was stirred for 1 h, followed by quenching with pyridine. The mixture was filtered, the filtrate was concentrated *in vacuo* and the residue was purified by silica gel column chromatography using a stepwise gradient of petroleum ether and EtOAc (3/1 to 1/1, v/v) to obtain compound **10** (361 mg, 71%). R_f = 0.48 (petroleum ether/EtOAc, 1.5/1, v/v). ^{1}H NMR (600 MHz, CDCl$_3$) δ 7.77 – 7.71 (m, 48H, CH Aromatic), 5.27 (d, *J* = 4.9

Hz, 1H, H1^{D}), 5.26 (d, *J* = 4.2 Hz, 1H, H1^{B}), 5.21 (d, *J* = 11.4 Hz, 1H, C*H*HBn), 5.17 (bd, *J* = 20.0 Hz, 2H, C*H*$_2$Cbz), 5.12 – 5.06 (m, 2H, H2^{F}, H4^{F}), 5.06 – 5.03 (m, H1^{E}, H1^{C}), 4.94 (t, *J* = 4.2 Hz, 1H, H^{B}), 4.91 – 4.78 (m, 5H, H2^{D}, 2 × C*H*HBn, H5^{B}, H1^{A}), 4.76 – 4.57 (m, 10H, 3 × C*H*HBn, 3 × C*H*$_2$ Bn, H1^{F}), 4.52 – 4.47 (m, 3H, NC*H*$_2$Bn, H5^{D}), 4.44 – 4.15 (m, 10H, H6a^{A}, H6a^{C}, H6a^{E}, H6b^{A}, H6b^{C}, H6b^{E}, C*H*$_2$ Fmoc, C5^{F}, C*H* Fmoc), 4.05 (t, *J* = 4.9 Hz, 1H, H4^{B}), 4.02 – 3.81 (m, 11H, H4^{D}, H3^{B}, H3^{D}, H3^{F}, H4^{E}, H4^{A}, H4^{C}, H5^{A}, H5^{C}, H5^{E}, H3^{A}), 3.70 – 3.81 (m, 3H, H3^{E}, H3^{C}, OCH*H* linker), 3.58 (s, 3H, CO$_2$C*H*$_3$), 3.50 (s, 3H, CO$_2$C*H*$_3$), 3.45 (s, 3H, CO$_2$C*H*$_3$), 3.40 – 3.16 (m, 6H, OCH*H* linker, C*H*$_2$N linker, H2^{A}, H2^{C}, H2^{E}), 2.97 – 2.40 (m, 20H, 10 × C*H*$_2$ Lev), 2.21 (s, 3H, C*H*$_3$ Lev), 2.17 (s, 3H, C*H*$_3$ Lev), 2.17 (s, 3H, C*H*$_3$ Lev), 2.11 (s, 3H, C*H*$_3$ Lev), 2.07 (s, 3H, C*H*$_3$ Lev), 1.97 (s, 3H, C*H*$_3$ Ac), 1.64 – 1.26 (m, 6H, 3 × C*H*$_2$ linker). ^{13}C NMR (151 MHz, CDCl$_3$) δ 207.0 (CH$_3$CO Lev), 206.7 (CH$_3$CO Lev), 206.6 (CH$_3$CO Lev), 206.3 (CH$_3$CO Lev), 206.2 (CH$_3$CO Lev), 172.7 (CH$_2$CO Lev), 172.6 (CH$_2$CO Lev), 172.5 (CH$_2$CO Lev), 172.2 (CH$_2$CO Lev), 172.0 (CH$_2$CO Lev), 169.9 (CO$_2$CH$_3$), 169.7 (CO$_2$CH$_3$), 169.2 (CO Ac), 167.6 (CO$_2$CH$_3$), 156.8, 156.3, 154.2, 143.5, 143.2, 141.4, 138.3, 138.2, 138.0, 137.8, 137.7, 137.6, 137.0, 136.9, 128.7, 128.6, 128.5, 128.4, 128.3, 128.2, 128.1, 128.0, 127.9, 127.8, 127.7, 127.6, 127.3, 125.3, 125.2, 100.7 (C1^{F}), 98.09 (C1^{D}), 98.06 (C1^{B}), 97.7 (C1^{A}, C1^{E}), 96.9 (C1^{C}), 79.4 (C3^{F}), 78.4 (C3^{A}), 78.0 (C3^{C}), 77.7 (C4^{E}), 77.5 (C3^{E}), 76.0 (C4^{A} or C4^{C}), 75.8 (C4^{A} or C4^{C}), 75.5 (2 × OCH$_2$Bn, C4^{F}), 75.4 (C3^{D}), 75.0 (OCH$_2$Bn), 74.6 (OCH$_2$Bn, C3^{B}), 74.1 (OCH$_2$Bn), 73.6 (OCH$_2$Bn), 73.0 (C4^{D}), 72.54 (C5^{F}), 72.45 (C2^{F}), 72.1 (C4^{B}), 70.6 (C2^{D}, C5^{D}), 70.4 (CH$_2$ Fmoc), 69.7 (C2^{B}, C5^{B}, C5^{A} or C5^{C} or C5^{E}), 69.2 (C5^{A} or C5^{C} or C5^{E}), 69.1 (C5^{A} or C5^{C} or C5^{E}), 68.2 (OCH$_2$ linker), 67.3 (CH$_2$Cbz), 63.3 (C2^{A} or C2^{C} or C2^{E}), 63.1 (C2^{A} or C2^{C} or C2^{E}), 62.7 (C2^{A} or C2^{C} or C2^{E}), 62.6 (C6^{A}), 62.1 (C6^{C}), 61.9 (C6^{E}), 52.7 (CO$_2$CH$_3$), 52.3 (CO$_2$CH$_3$), 52.0 (CO$_2$CH$_3$), 50.7 (NCH$_2$Bn), 50.4 (NCH$_2$Bn), 47.2 (CH$_2$N linker), 46.7 (CH Fmoc), 46.3 (CH$_2$N linker), 38.2 (CH$_2$ Lev), 38.0 (2 × CH$_2$ Lev), 37.8 (CH$_2$ Lev), 37.7 (CH$_2$ Lev), 29.9 (2 × CH$_3$ Lev), 29.9 (CH$_3$ Lev), 29.8 (2 × CH$_3$ Lev), 29.6 (CH$_2$ linker), 29.0 (CH$_2$ linker), 28.0 (3 × CH$_2$ Lev), 27.8 (2 × CH$_2$ Lev), 27.5 (CH$_2$ linker), 23.4 (CH$_2$ linker), 20.8 (CH$_3$ Ac). HRMS (ESI-MS) (pos): m/z calculated for $C_{94}H_{115}N_8O_{31}$ [M+NH$_4$]$^+$: 2772.0860; found: 2772.0888.

***N*-(Benzyl)-benzyloxycarbonyl-5-aminopentyl *O*-(methyl-3-*O*-benzyl-β-D-glucopyranosyluronate)-(1→4)-*O*-(2-azido-3-*O*-benzyl-2-deoxy-α-D-glucopyranosyl)-(1→4)-*O*-(methyl-3-*O*-benzyl-α-L-idopyranosyluronate)-(1→4)-*O*-(2-azido-3-*O*-benzyl-2-deoxy-α-D-glucopyranosyl)-(1→4)-*O*-(methyl-3-*O*-benzyl-α-L-idopyranosyluronate)-(1→4)-*O*-(2-azido-3-*O*-benzy-2-deoxy-α-D-glucopyranoside, sodium salt (11).**

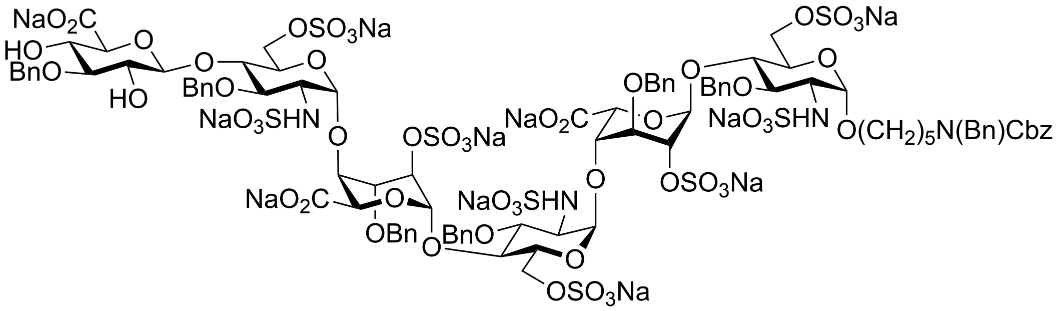

Compound **11** was prepared as illustrated in **Scheme S1**. Compound **10** (350 mg, 0.127 mmol) was subjected to (1) Fmoc removal, (2) *O*-acetylation, (3) Lev ester removal, (4) *O*-sulfation, (5) saponification, de-*O*-acetylation reaction, (6) azido reduction, and (7) *N*-sulfation according to the general procedure to provide compound **11** as a white powder (77.9 mg, 22% over seven steps). ^{1}H NMR (600 MHz, $CDCl_3$) δ 7.60 – 7.20 (m, 40H, CH Aromatic), 5.54 (bs, 1H, H1^{B}), 5.36 (bs, 1H, H1^{D}), 5.32 – 5.27 (m, 2H, H1^{C}, H1^{E}), 5.18 – 5.09 (m, 2H, C*H*$_2$Cbz), 5.09 – 5.02 (m, 1H, H1^{A}), 4.95 – 4.58 (m, 15H, H5^{B}, H5^{D}, H2^{B}, H2^{D}, 5 × C*H*$_2$ Bn, H1^{F}), 4.70 – 4.47 (m, 4H, C*H*HBn, NC*H*$_2$Bn, H6a^{E}), 4.47 – 4.24 (m, 7H, H6a^{A}, H6a^{C}, H6b^{A}, H6b^{C}, H3^{D}, C3^{B}, C*H*HBn), 4.24 – 4.15 (m, 3H, H6b^{E}, H4^{B}, H4^{D}), 4.05 – 3.82 (m, 6H, H5^{A}, H5^{C}, H5^{E}, H4^{E}, H4^{C}, H4^{A}), 3.80 (t, *J* = 9.4 Hz, 1H, H3^{E}), 3.75 – 3.55 (m, 6H, OCH*H* linker, H4^{F}, H5^{F}, H3^{A}, H3^{C}, H3^{E}), 3.48 – 3.34 (m, 5H, OCH*H* linker, H2^{E}, H2^{C}, H2^{A}, H2^{F}), 3.33 – 3.23 (m, 2H, C*H*$_2$N linker), 1.63 – 1.24 (m, 6H, 3 × C*H*$_2$ linker). ^{13}C NMR from HSQC (151 MHz, $CDCl_3$) 129.3, 128.8, 128.6, 128.5, 101.9 (C1^{F}), 98.5 (C1^{C}, C1^{E}), 97.7 (C1^{B}), 97.4 (C1^{D}), 96.9 (C1^{A}), 83.5 (C3^{F}), 77.4 (C3^{A}, C3^{C}, C3^{E}), 76.3 (C5^{F}), 75.6 (O$\underline{C}H_2$Bn), 75.3 (C4^{A}), 75.2 (C4^{D}), 74.8 (2 × O$\underline{C}H_2$Bn), 74.7 (C4^{E}, C4^{B}), 74.0 (O$\underline{C}H_2$Bn, C3^{B}, C3^{D}), 73.1 (C2^{F}), 72.4 (2 × O$\underline{C}H_2$Bn, C4^{C}), 71.6 (C2^{D}), 71.5 (C2^{B}, C4^{F}), 69.5 (C5^{C}, C5^{E}), 69.0 (C5^{A}), 68.2 (O$\underline{C}H_2$ linker), 67.8 (C5^{B}), 67.6 ($\underline{C}H_2$Cbz, C5^{D}), 67.1 (C6^{C}), 66.5 (C6^{A}), 66.0 (C6^{E}), 57.8 (C2^{C}), 57.3 (C2^{A}, C2^{E}), 50.5 (N$\underline{C}H_2$Bn), 47.2 ($\underline{C}H_2$N linker), 28.2 ($\underline{C}H_2$ linker), 27.2 ($\underline{C}H_2$ linker), 23.0 ($\underline{C}H_2$ linker). ESI-

MS (neg): m/z calculated for $C_{98}H_{115}N_4NaO_{57}S_8$ [M-10Na+8H]$^{2-}$: 1269.6965; found: 1269.6923.

^{1}H NMR (600 MHz, D_2O)

	H1	H2	H3	H4	H5	H6
**A**	5.05	3.37	3.64	3.85	3.99	4.42, 4.34
**B**	5.54	4.65	4.31	4.20	4.81	–
**C**	5.31	3.41	3.65	3.90	3.99	4.42, 4.29
**D**	5.36	4.63	4.33	4.18	4.67	–
**E**	5.31	3.44	3.80	3.96	3.99	4.50, 4.16
**F**	4.64	3.38	3.58	3.70	3.70	–

^{13}C NMR from HSQC (151 MHz, D_2O)

	C1	C2	C3	C4	C5	C6
**A**	96.9	57.3	77.4	75.3	69.0	66.5
**B**	97.7	71.5	74.0	74.7	67.8	–
**C**	98.5	57.8	77.4	72.4	69.5	67.1
**D**	97.4	71.6	74.0	75.2	67.6	–
**E**	98.5	57.3	77.4	74.7	69.5	66.0
**F**	101.9	73.1	83.5	71.5	76.3	–

5-Aminopentyl *O*-(β-D-glucopyranosyluronate)-(1→4)-*O*-(2-sulfamino-6-*O*-sulfate-2-deoxy-α-D-glucopyranosyl)-(1→4)-*O*-(2-*O*-sulfate-α-L-idopyranosyluronate)-(1→4)-*O*-(2-sulfamino-6-*O*-sulfate-2-deoxy-α-D-glucopyranosyl)-(1→4)-*O*-(2-*O*-sulfate-α-L-idopyranosyluronate)-(1→4)-*O*-2-sulfamino-6-*O*-sulfate-2-deoxy-α-D-glucopyranoside, sodium salt (1).

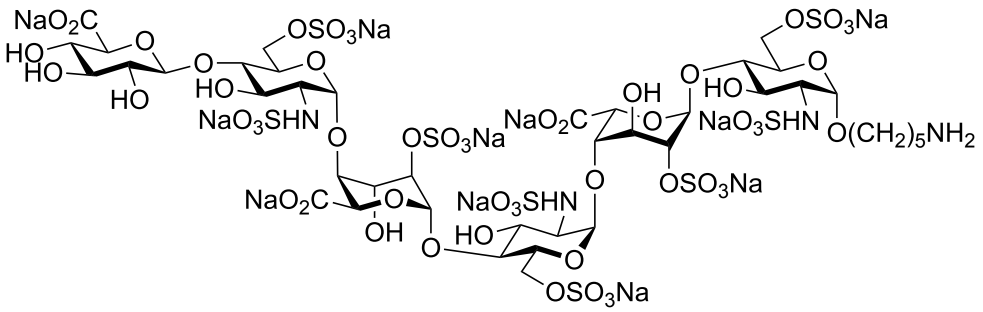

Compound **11** (67.7 mg, 24.51 μmol) was subjected to global deprotection according to the general procedure to give the title compound **1** as a white powder (41.6 mg, 85%). ^{1}H NMR (600 MHz, D_2O) δ 5.44 (d, *J* = 3.5 Hz, 1H, H1^{C}), 5.42 (d, *J* = 3.5 Hz, 1H, H1^{E}), 5.31 (d, *J* = 3.1 Hz, 1H, H1^{B}), 5.22 (d, *J* = 2.2 Hz, 1H, H1^{D}), 5.13 (d, *J* = 3.5 Hz, 1H, H1^{A}), 4.90 (d, *J* = 2.2 Hz, 1H, H5^{D}), 4.80 (1H, H5^{B}), 4.61 (d, *J* = 7.9 Hz, 1H, H1^{F}), 4.48 (dd, *J* = 11.0, 1.7 Hz, 1H, H6a^{E}), 4.42 – 4.19 (m, 9H, H6a^{C}, H6a^{A}, H6b^{A}, H6b^{C}, H6b^{E}, H2^{D}, H2^{B}, H3^{D}, H3^{B}), 4.15 – 4.03 (m, 4H, H4^{B}, H4^{D}, H5^{E}, H5^{A}), 4.01 – 3.97 (m, 1H, H5^{C}), 3.82 – 3.69 (m, 7H, H3^{A}, OCH*H* linker, H3^{E}, H4^{C}, H4^{E}, H5^{F}, H4^{A}), 3.66 – 3.62 (m, 1H, H3^{C}), 3.60 – 3.50 (m, 3H, OCH*H* linker, H3^{F}, H4^{F}), 3.36 (t, *J* = 8.5 Hz, 1H, H2^{F}), 3.31 – 3.26 (m, 3H, H2^{C}, H2^{E}, H2^{A}), 3.04 (t, *J* = 7.3 Hz, 2H, NCH_2 linker), 1.78 – 1.47 (m, 6H, 3 × CH_2 linker). ^{13}C NMR from HSQC (151 MHz, D_2O) δ 101.7 (C1^{F}), 99.1 (C1^{D}), 98.2 (C1^{B}), 97.0 (C1^{A}), 96.7 (C1^{C}, C1^{E}), 77.0 (C4^{A}, C5^{F}), 76.3 (C2^{B}), 76.1 (C4^{D}), 75.9 (C2^{D}, C4^{B}, C4^{C}, C4^{E}), 75.0 (C3^{F}), 72.9 (C2^{F}), 71.9 (C4^{F}), 69.6 (C3^{C}), 69.3 (C3^{E}, C5^{B}), 69.2 (C5^{C}), 69.1 (C3^{B}, C3^{A}), 68.9 (C5^{D}, C3^{D}, C5^{E}), 68.5 (C5^{A}), 67.9 (O$\underline{C}H_2$ linker), 67.0 (C6^{A}), 66.4 (C6^{C}), 65.8 (C6^{E}), 57.7 (C2^{A}, C2^{C}, C2^{E}), 39.5 (N$\underline{C}H_2$ linker), 28.0 ($\underline{C}H_2$ linker), 26.3 ($\underline{C}H_2$ linker), 22.5 ($\underline{C}H_2$ linker). ESI-MS (neg): m/z calculated for $C_{41}H_{66}N_4Na_2O_{55}S_8$ [M-9Na+7H]$^{2-}$: 898.0031; found: 898.0012.

^{1}H NMR (600 MHz, D_2O)

	H1	H2	H3	H4	H5	H6
**A**	5.13 (d, *J* = 3.5 Hz)	3.29	3.80	3.74	4.06	4.38, 4.30
**B**	5.31 (d, *J* = 3.1 Hz)	4.33	4.22	4.13	4.80	–

**C**	5.44 (d, J = 3.5 Hz)	3.30	3.65	3.78	4.00	4.40, 4.28
**D**	5.22 (d, J = 2.2 Hz)	4.34	4.24	4.11	4.91	–
**E**	5.42 (d, J = 3.5 Hz)	3.30	3.71	3.78	4.09	4.49, 4.25
**F**	4.61 (d, J = 7.9 Hz)	3.36	3.54	3.52	3.77	–

^{13}C NMR from HSQC (151 MHz, D_2O)

	**C1**	**C2**	**C3**	**C4**	**C5**	**C6**
**A**	97.0	57.7	69.1	77.0	68.5	67.0
**B**	98.2	76.3	69.1	75.9	69.3	–
**C**	96.7	57.7	69.6	75.9	69.2	66.4
**D**	99.1	75.9	68.9	76.1	68.9	–
**E**	96.7	57.7	69.3	75.9	68.9	65.8
**F**	101.7	72.9	75.0	71.9	77.0	–

5-(Pent-4-ynamido) pentyl *O*-(β-D-glucopyranosyluronate)-(1→4)-*O*-(2-sulfamino-6-*O*-sulfate-2-deoxy-α-D-glucopyranosyl)-(1→4)-*O*-(2-*O*-sulfate-α-L-idopyranosyluronate)-(1→4)-*O*-(2-sulfamino-6-*O*-sulfate-2-deoxy-α-D-glucopyranosyl)-(1→4)-*O*-(2-*O*-sulfate-α-L-idopyranosyluronate)-(1→4)-*O*-2-sulfamino-6-*O*-sulfate-2-deoxy-α-D-glucopyranoside, sodium salt (2).

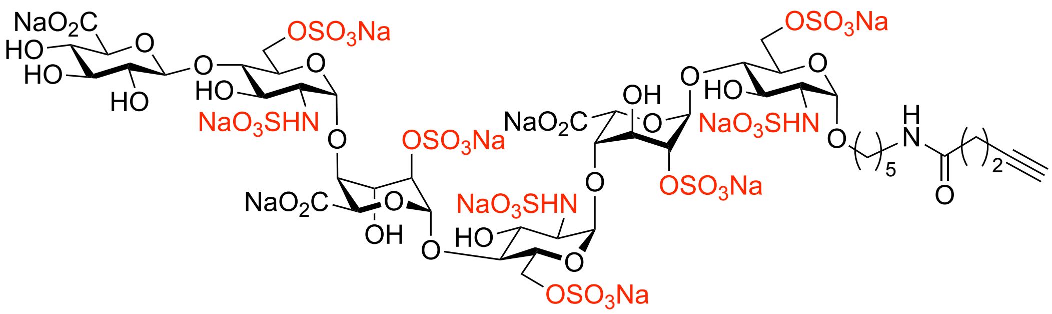

Compound **1** (14.3 mg, 7.16 μmol) was subjected to installation of alkyne moiety according to the general procedure to give the title compound **2** as a white powder (12.8 mg, 86%). ^{1}H NMR (600 MHz, D_2O) δ 5.45 (d, *J* = 3.2 Hz, 1H, H1^{C}), 5.42 (d, *J* = 3.5 Hz, 1H, H1^{E}), 5.26 – 5.15 (m, 2H, H1^{D}, H1^{B}), 5.13 (d, *J* = 3.5 Hz, 1H, H1^{A}), 4.83 (1H, H5^{D}), 4.74 (1H, H5^{B}), 4.61 (d, *J* = 7.7 Hz, 1H, H1^{F}), 4.50 (d, *J* = 10.5 Hz, 1H, H6a^{E}), 4.44 – 4.16 (m, 9H, H6a^{C}, H6a^{A}, H6b^{A}, H6b^{C}, H6b^{E}, H2^{D}, H2^{B}, H3^{D}, H3^{B}), 4.15 – 4.08 (m, 3H, H5^{E}, H4^{B}, H4^{D}), 4.05 – 3.98 (m, 2H, H5^{C}, H5^{A}), 3.81 – 3.63 (m, 8H, H4^{C}, H4^{E}, H5^{F}, H4^{A}, OCH*H* linker, H3^{E}, H3^{A}, H3^{C}), 3.57 – 3.49 (m, 3H, OCH*H* linker, H3^{F}, H4^{F}), 3.36 (t, *J* = 8.5 Hz, 1H, H2^{F}), 3.33 – 3.18 (m, 5H, H2^{C}, H2^{E}, H2^{A}, NCH_2 linker), 2.56 – 2.38 (m, 5H, CH_2C≡CH, CH_2CO, C≡C*H*), 1.78 – 1.38 (m, 6H, 3 × CH_2 linker). ^{13}C NMR from HSQC (151 MHz, D_2O) δ 101.8 (C1^{F}), 99.5 (C1^{D}, C1^{B}), 96.9 (C1^{A}), 96.5 (C1^{E}), 96.2 (C1^{C}), 77.0 (C4^{A}, C4^{C}, C4^{E}), 76.7 (C2^{B}), 75.9 (C2^{D}, C4^{B}, C4^{D}, C5^{F}), 75.1 (C3^{F}), 73.0 (C2^{F}), 72.0 (C4^{F}), 70.6 (C≡$\underline{C}$H), 69.8 (C5^{B}, C3^{A}, C3^{C}), 69.7 (C3^{E}, C3^{B}), 69.2 (C5^{D}, C3^{D}, C5^{C}), 68.8 (C5^{E}, C5^{A}), 68.4 (O$\underline{C}H_2$ linker), 67.0 (C6^{A}), 66.4 (C6^{C}), 65.8 (C6^{E}), 57.8 (C2^{A}, C2^{C}, C2^{E}), 39.2 (N$\underline{C}H_2$ linker), 34.6 ($\underline{C}H_2$CO), 28.1 ($\underline{C}H_2$ linker), 28.1 ($\underline{C}H_2$ linker), 22.8 ($\underline{C}H_2$ linker), 14.6 ($\underline{C}H_2$C≡CH). ESI-MS (neg): m/z calculated for $C_{46}H_{72}N_4O_{56}S_8$ [M-11Na+9H]$^{2-}$: 916.0343; found: 915.9842.

^{1}H NMR (600 MHz, D_2O)

	**H1**	**H2**	**H3**	**H4**	**H5**	**H6**
**A**	5.13 (d, *J* = 3.5 Hz)	3.29	3.67	3.73	4.01	4.36
**B**	5.19	4.32	4.18	4.13	4.74	–
**C**	5.45 (d, *J* = 3.2 Hz)	3.30	3.66	3.78	4.03	4.40, 4.28
**D**	5.22	4.34	4.20	4.10	4.83	–
**E**	5.42 (d, *J* = 3.5 Hz)	3.30	3.72	3.78	4.12	4.48, 4.24
**F**	4.61 (d, *J* = 7.7 Hz)	3.36	3.54	3.51	3.77	–

^{13}C NMR from HSQC (151 MHz, D_2O)

	C1	C2	C3	C4	C5	C6
**A**	96.9	57.8	69.8	77.0	68.8	67.0
**B**	99.5	76.7	69.7	75.9	69.8	–
**C**	96.2	57.8	69.8	77.0	69.2	66.4
**D**	99.5	75.9	69.2	75.9	69.2	–
**E**	96.5	57.8	69.7	77.0	68.8	65.8
**F**	101.8	73.0	75.1	72.0	75.9	–

5-Aminopentyl *O*-(2-acetamido-6-azido-2-deoxy-α-D-glucopyranosyl)-(1→4)-*O*-(β-D-glucopyranosyluronate)-(1→4)-*O*-(2-sulfamino-6-*O*-sulfate-2-deoxy-α-D-glucopyranosyl)-(1→4)-*O*-(2-*O*-sulfate-α-L-idopyranosyluronate)-(1→4)-*O*-(2-sulfamino-6-*O*-sulfate-2-deoxy-α-D-glucopyranosyl)-(1→4)-*O*-(2-*O*-sulfate-α-L-idopyranosyluronate)-(1→4)-*O*-2-sulfamino-6-*O*-sulfate-2-deoxy-α-D-glucopyranoside, sodium salt (3).

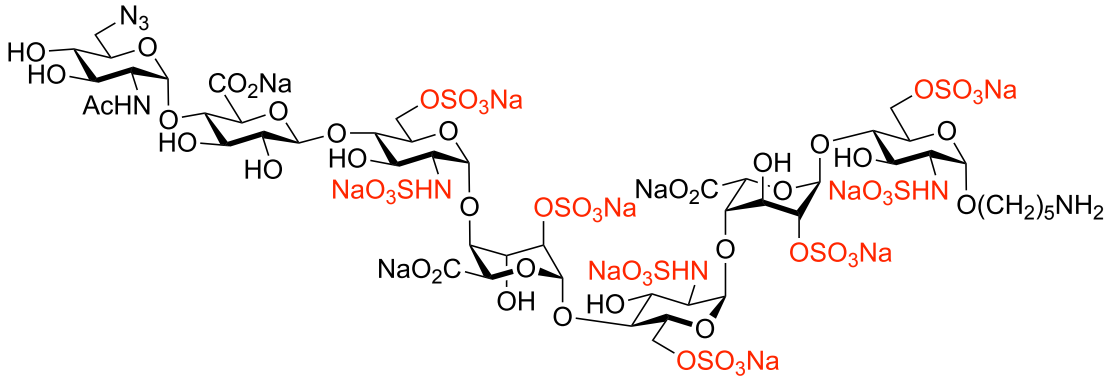

Compound **1** (12.7 mg, 6.36 µmol) was subjected to installation of unnatural α (1→4) 6-azido-GlcNAc according to the general procedure to give the title compound **3** as a white powder (12.0 mg, 85%). ^1H NMR (600 MHz, D_2O) δ 5.48 – 5.40 (m, 3H, $H1^C$, $H1^E$, $H1^G$), 5.30 (d, J = 3.2 Hz, 1H, $H1^B$), 5.21 (bs, 1H, $H1^D$), 5.13 (d, J = 3.2 Hz, 1H, $H1^A$), 4.82 (1H, $H5^D$), 4.69 (d, J = 2.2 Hz, 1H, $H5^B$), 4.61 (d, J = 7.9 Hz, 1H, $H1^F$), 4.48 (d, J = 10.6 Hz, 1H, $H6a^E$), 4.43 –

4.16 (m, 9H, H6a^C, H6a^A, H6b^A, H6b^C, H6b^E, H2^D, H2^B, H3^D, H3^B), 4.15 – 4.07 (m, 3H, H4^B, H4^D, H5^E), 4.07 – 3.98 (m, 2H, H5^A, H5^C), 3.94 – 3.87 (m, 2H, H2^G, H5^G), 3.84 – 3.53 (m, 14H, H3^A, OCH*H* linker, H3^E, H3^G, H3^C, H5^F, H4^F, H3^F, H4^E, H4^C, H4^A, H6a^G, H6b^G, OCH*H* linker), 3.48 (t, *J* = 9.6 Hz, 1H, H4^G), 3.35 (t, *J* = 8.5 Hz, 1H, H2^F), 3.31 – 3.24 (m, 3H, H2^C, H2^E, H2^A), 3.04 (t, *J* = 7.3 Hz, 2H, NCH_2 linker), 2.06 (s, 3H, NHCOCH_3), 1.78 – 1.46 (m, 6H, 3 × CH_2 linker). ^{13}C NMR from HSQC (151 MHz, D_2O) δ 101.6 (C1^F), 99.2 (C1^D), 98.1 (C1^B), 97.0 (C1^A), 96.6 (C1^G, C1^E), 96.1 (C1^C), 76.8 (C2^B), 76.5 (C5^F, C4^F, C3^F, C4^E, C4^C, C4^A), 76.1 (C2^D), 75.9 (C4^B, C4^D), 73.5 (C2^F), 70.7 (C5^G), 70.4 (C4^G), 70.0 (C5^B, C3^G, C3^E), 69.7 (C3^C), 69.4 (C3^D, C3^B), 69.3 (C5^D), 69.1 (C5^C), 68.9 (C3^A), 68.7 (C5^E), 68.6 (C5^A), 67.9 (O$\underline{C}H_2$ linker), 67.1 (C6^A), 66.6 (C6^C), 66.0 (C6^E), 57.8 (C2^A, C2^C, C2^E), 53.6 (C2^G), 50.5 (C6^G), 39.6 (N$\underline{C}H_2$ linker), 28.1 ($\underline{C}H_2$ linker), 26.3 ($\underline{C}H_2$ linker), 22.6 ($\underline{C}H_2$ linker), 21.9 (NHCO$\underline{C}H_3$). ESI-MS (neg): m/z calculated for $C_{49}H_{80}N_8O_{59}S_8$ [M-11Na+9H]$^{2-}$: 990.0641; found: 990.0218.

^{1}H NMR (600 MHz, D_2O)

	**H1**	**H2**	**H3**	**H4**	**H5**	**H6**
**A**	5.13<br>(d, *J* = 3.2 Hz)	3.28	3.81	3.74	4.05	4.36, 4.28
**B**	5.30<br>(d, *J* = 3.2 Hz)	4.32	4.18	4.13	4.69	–
**C**	5.46	3.28	3.64	3.77	4.02	4.41, 4.28
**D**	5.22	4.34	4.20	4.10	4.82	–
**E**	5.42	3.28	3.71	3.78	4.11	4.48, 4.24
**F**	4.61<br>(d, *J* = 7.9 Hz)	3.36	3.73	3.77	3.81	–
**G**	5.43<br>(d, *J* = 3.5 Hz)	3.91	3.73	3.48	3.89	3.65

^{13}C NMR from HSQC (151 MHz, D_2O)

	C1	C2	C3	C4	C5	C6
**A**	97.0	57.8	68.9	76.5	68.6	67.1
**B**	98.1	76.8	69.4	75.9	70.0	–
**C**	96.1	57.8	69.7	76.5	69.1	66.6
**D**	99.2	76.1	69.4	75.9	69.3	–
**E**	96.6	57.8	70.0	76.5	68.7	66.0
**F**	101.6	73.5	76.5	76.5	76.5	–
**G**	96.6	53.6	70.0	70.4	70.7	50.5

5-Aminopentyl *O*-(β-D-glucopyranosyluronate)-(1→4)-*O*-(2-sulfamino-6-*O*-sulfate-2-deoxy-α-D-glucopyranosyl)-(1→4)-*O*-(2-*O*-sulfate-α-L-idopyranosyluronate)-(1→4)-*O*-(2-sulfamino-6-*O*-sulfate-2-deoxy-α-D-glucopyranosyl)-(1→4)-*O*-(2-*O*-sulfate-α-L-idopyranosyluronate)-(1→4)-*O*-2-sulfamino-6-*O*-sulfate-2-deoxy-α-D-glucopyranosyl) -*O*-[*N*-ethyl-3-(1*H*-1,2,3-triazol-4-yl)propanamide]-(1→6)-*N*-(2-acetamido-2-deoxy-α-D-glucopyranosyl)-(1→4)-*O*-(β-D-glucopyranosyluronate)-(1→4)-*O*-(2-sulfamino-6-*O*-sulfate-2-deoxy-α-D-glucopyranosyl)-(1→4)-*O*-(2-*O*-sulfate-α-L-idopyranosyluronate)-(1→4)-*O*-(2-sulfamino-6-*O*-sulfate-2-deoxy-α-D-glucopyranosyl)-(1→4)-*O*-(2-*O*-sulfate-α-L-idopyranosyluronate)-(1→4)-*O*-2-sulfamino-6-*O*-sulfate-2-deoxy-α-D-glucopyranoside, sodium salt (4).

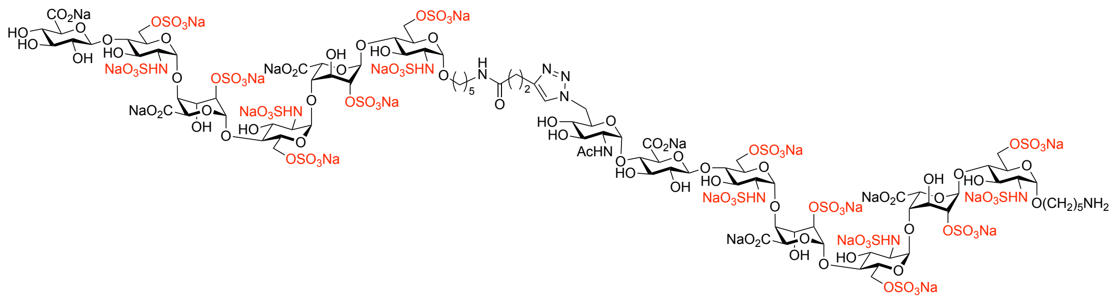

Compound **2** (1.2 mg, 0.578 μmol) and compound **3** (1.0 mg, 0.449 μmol) was subjected to copper (I)-catalyzed azide-alkyne cycloaddition (CuAAC) reaction according the general

procedure (small scale: 10 parallel reactions) to give the title compound **4** as a white powder (13.4 mg, 69% average). ^{1}H NMR (600 MHz, D_2O) δ 7.85 (s, 1H, C*H* triazole), 5.47 – 5.41 (m, 4H, $H1^{C}$, $H1^{C1}$, $H1^{E}$, $H1^{E1}$), 5.34 (d, *J* = 3.5 Hz, 1H, $H1^{G}$), 5.31 (bs, 1H, $H1^{B}$), 5.21 (bs, 2H, $H1^{D}$, $H1^{D1}$), 5.18 (bs, 1H, $H1^{B1}$), 5.15 – 5.12 (m, 2H, $H1^{A}$, $H1^{A1}$), 4.85 – 4.73 (m, 4H, $H6a^{G}$, $H5^{D}$, $H5^{D1}$, $H5^{B1}$), 4.69 (bs, 1H, $H5^{B}$), 4.65 – 4.54 (m, 3H, $H6b^{G}$, $H1^{F}$, $H1^{F1}$), 4.53 – 4.45 (m, 2H, $H6a^{E}$, $H6a^{E1}$), 4.43 – 4.16 (m, 18H, $H6a^{C}$, $H6a^{C1}$, $H6a^{A}$, $H6a^{A1}$, $H6b^{A}$, $H6b^{A1}$, $H6b^{C}$, $H6b^{C1}$, $H6b^{E}$, $H6b^{E1}$, $H2^{D}$, $H2^{D1}$, $H2^{B}$, $H2^{B1}$, $H3^{D}$, $H3^{D1}$, $H3^{B}$, $H3^{B1}$), 4.15 – 3.97 (m, 11H, $H5^{E}$, $H5^{E1}$, $H5^{G}$, $H4^{B}$, $H4^{B1}$, $H4^{D}$, $H4^{D1}$, $H5^{A}$, $H5^{C}$, $H5^{C1}$, $H5^{A1}$), 3.85 – 3.49 (m, 24H, $H3^{A}$, OCH*H* linker, OCH*H* linker 1, $H3^{G}$, $H3^{E}$, $H3^{E1}$, $H2^{G}$, $H3^{C}$, $H3^{C1}$, $H3^{A1}$, OCH*H* linker, OCH*H* linker 1, $H5^{F}$, $H5^{F1}$, $H4^{F}$, $H4^{E}$, $H4^{E1}$, $H4^{C}$, $H4^{C1}$, $H3^{F}$, $H4^{A}$, $H4^{A1}$, $H3^{F1}$, $H4^{F1}$), 3.39 – 3.34 (m, 2H, $H2^{F}$, $H2^{F1}$), 3.31 – 3.25 (m, 6H, $H2^{C}$, $H2^{C1}$, $H2^{E}$, $H2^{E1}$, $H2^{A}$, $H2^{A1}$), 3.20 – 3.12 (m, 2H, NCH_2 linker 1), 3.04 (t, *J* = 7.3 Hz, 2H, NCH_2 linker), 3.00 (t, *J* = 7.7 Hz, 2H, CH_2-triazole), 2.79 – 2.72 (m, 1H, $H4^{G}$), 2.61 (t, *J* = 7.8 Hz, 2H, CH_2CONH), 2.02 (s, 3H, NHCOCH_3), 1.79 – 1.32 (m, 12H, 6 × CH_2 linker). ^{13}C NMR from HSQC (151 MHz, D_2O) δ 125.1 (CH triazole), 101.7 ($C1^{F}$, $C1^{F1}$), 99.5 ($C1^{B1}$), 99.2 ($C1^{D}$, $C1^{D1}$), 98.0 ($C1^{B}$), 97.4 ($C1^{G}$), 97.0 ($C1^{A}$, $C1^{A1}$), 96.5 ($C1^{E}$, $C1^{E1}$), 96.1 ($C1^{C}$, $C1^{C1}$), 76.6 ($C2^{B}$, $C2^{B1}$, $C5^{F}$, $C5^{F1}$, $C4^{F}$, $C4^{E}$, $C4^{E1}$, $C4^{C}$, $C4^{C1}$, $C3^{F}$, $C4^{A}$, $C4^{A1}$), 76.0 ($C2^{D}$, $C2^{D1}$, $C4^{B}$, $C4^{B1}$, $C4^{D}$, $H4^{D1}$), 75.0 ($C3^{F1}$), 73.2 ($C2^{F}$, $C2^{F1}$), 72.0 ($C4^{F1}$), 69.9 ($C5^{B1}$, $C5^{B}$, $C4^{G}$), 69.7 ($C3^{G}$, $C3^{E}$, $C3^{E1}$, $C3^{C}$, $C3^{C1}$, $C3^{A1}$), 69.4 ($C3^{B}$, $C3^{B1}$, $C3^{D}$, $C3^{D1}$), 69.3 ($C5^{D}$, $C5^{D1}$, $C3^{A}$), 69.2 ($C5^{E}$, $C5^{E1}$, $C5^{G}$, $C5^{A}$, $C5^{C}$, $C5^{C1}$, $C5^{A1}$), 68.6 (O$\underline{C}H_2$ linker 1), 68.0 (O$\underline{C}H_2$ linker), 67.0 ($C6^{A}$, $C6^{A1}$), 66.4 ($C6^{C}$, $C6^{C1}$), 65.8 ($C6^{E}$, $C6^{E1}$), 57.8 ($C2^{A}$, $C2^{A1}$, $C2^{C}$, $C2^{C1}$, $C2^{E}$, $C2^{E1}$), 53.5 ($C2^{G}$), 49.7 ($C6^{G}$), 39.5 (2 × N$\underline{C}H_2$ linker), 35.3 ($\underline{C}H_2$CONH), 28.1 ($\underline{C}H_2$ linker, $\underline{C}H_2$ linker 1), 26.4 ($\underline{C}H_2$ linker, $\underline{C}H_2$ linker 1), 22.7 ($\underline{C}H_2$ linker, $\underline{C}H_2$ linker 1), 21.9 (NHCO$\underline{C}H_3$), 21.2 ($\underline{C}H_2$-triazole). ESI-MS (neg): m/z calculated for $C_{95}H_{153}N_{12}O_{115}S_{16}$ $[M-22Na+19H]^{3-}$: 1271.4025; found: 1271.3490.

^{1}H NMR (600 MHz, D_2O)

	H1	H2	H3	H4	H5	H6
**A**	5.13	3.28	3.82	3.75	4.05	4.36, 4.31
**A1**	5.13	3.28	3.65	3.73	4.00	4.36, 4.31
**B**	5.31	4.32	4.19	4.13	4.69	–
**B1**	5.18	4.32	4.18	4.13	4.76	–
**C/C1**	5.45	3.28	3.64	3.77	4.02	4.41, 4.28
**D/D1**	5.22	4.34	4.20	4.10	4.83	–
**E/E1**	5.43	3.28	3.71	3.78	4.11	4.48, 4.24
**F**	4.60	3.36	3.73	3.77	3.81	–
**F1**	4.60	3.36	3.54	3.51	3.77	–
**G**	5.35 (d, J = 3.5 Hz)	3.68	3.72	2.75	4.11	4.87, 4.59

^{13}C NMR from HSQC (151 MHz, D_2O)

	C1	C2	C3	C4	C5	C6
**A**	97.0	57.8	69.3	76.6	69.2	67.0
**A1**	97.0	57.8	69.7	76.6	69.2	67.0
**B**	98.0	76.6	69.4	76.0	69.9	–
**B1**	99.5	76.6	69.4	76.0	69.9	–
**C/C1**	96.1	57.8	69.7	76.6	69.2	66.4
**D/D1**	99.2	76.0	69.4	76.0	69.3	–
**E/E1**	96.5	57.8	69.7	76.6	69.2	65.8
**F**	101.7	73.2	76.6	76.6	76.6	–
**F1**	101.7	73.2	75.0	72.0	76.6	–
**G**	97.4	53.5	69.7	69.9	69.2	49.7

5-(Pent-4-ynamido) pentyl *O*-(β-D-glucopyranosyluronate)-(1→4)-*O*-(2-sulfamino-6-*O*-sulfate-2-deoxy-α-D-glucopyranosyl)-(1→4)-*O*-(2-*O*-sulfate-α-L-idopyranosyluronate)-(1→4)-*O*-(2-sulfamino-6-*O*-sulfate-2-deoxy-α-D-glucopyranosyl)-(1→4)-*O*-(2-*O*-sulfate-α-L-idopyranosyluronate)-(1→4)-*O*-2-sulfamino-6-*O*-sulfate-2-deoxy-α-D-glucopyranosyl) -*O*-[*N*-ethyl-3-(1*H*-1,2,3-triazol-4-yl)propanamide]-(1→6)-*N*-(2-acetamido-2-deoxy-α-D-glucopyranosyl)-(1→4)-*O*-(β-D-glucopyranosyluronate)-(1→4)-*O*-(2-sulfamino-6-*O*-sulfate-2-deoxy-α-D-glucopyranosyl)-(1→4)-*O*-(2-*O*-sulfate-α-L-idopyranosyluronate)-(1→4)-*O*-(2-sulfamino-6-*O*-sulfate-2-deoxy-α-D-glucopyranosyl)-(1→4)-*O*-(2-*O*-sulfate-α-L-idopyranosyluronate)-(1→4)-*O*-2-sulfamino-6-*O*-sulfate-2-deoxy-α-D-glucopyranoside, sodium salt (12).

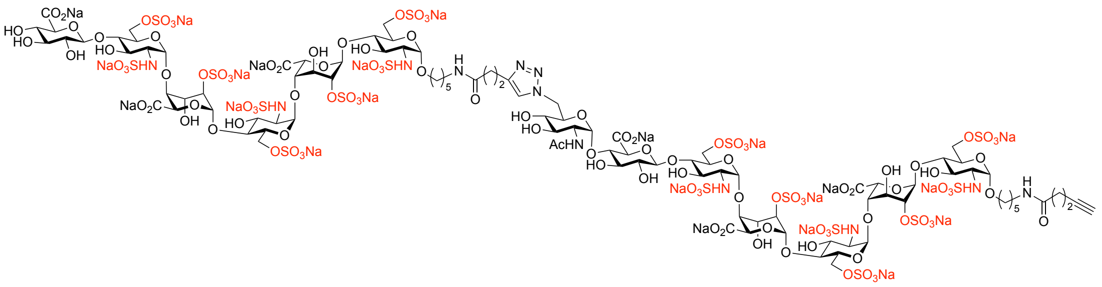

Compound **4** (6.1 mg, 1.42 μmol) was subjected to installation of alkyne moiety according to the general procedure to give the title compound **12** as a white powder (5.0 mg, 81%). ^{1}H NMR (600 MHz, D_2O) δ 7.85 (s, 1H, C*H* triazole), 5.48 – 5.44 (m, 2H, H1^{C}, H1^{C1}), 5.44 – 5.40 (m, 2H, H1^{E}, H1^{E1}), 5.34 (d, J = 2.8 Hz, 1H, H1^{G}), 5.21 (bs, 2H, H1^{D}, H1^{D1}), 5.18 (bs, 1H, H1^{B}, H1^{B1}), 5.15 – 5.12 (m, 2H, H1^{A}, H1^{A1}), 4.89 – 4.71 (m, 5H, H6a^{G}, H5^{D}, H5^{D1}, H5^{B}, H5^{B1}), 4.64 – 4.54 (m, 3H, H6b^{G}, H1^{F}, H1^{F1}), 4.53 – 4.45 (m, 2H, H6a^{E}, H6a^{E1}), 4.44 – 4.16 (m, 18H, H6a^{C}, H6a^{C1}, H6a^{A}, H6a^{A1}, H6b^{A}, H6b^{A1}, H6b^{C}, H6b^{C1}, H6b^{E}, H6b^{E1}, H2^{D}, H2^{D1}, H2^{B}, H2^{B1}, H3^{D}, H3^{D1}, H3^{B}, H3^{B1}), 4.15 – 4.08 (m, 7H, H5^{E}, H5^{E1}, H5^{G}, H4^{B}, H4^{B1}, H4^{D}, H4^{D1}), 4.05 – 3.97 (m, 4H, H5^{C}, H5^{C1}, H5^{A}, H5^{A1}), 3.84 – 3.63 (m, 20H, OCH*H* linker, OCH*H* linker 1, H3^{G}, H3^{E}, H3^{E1}, H2^{G}, H3^{C}, H3^{C1}, H3^{A}, H3^{A1}, H5^{F}, H5^{F1}, H4^{F}, H4^{E}, H4^{E1}, H4^{C}, H4^{C1}, H3^{F}, H4^{A}, H4^{A1}), 3.58 – 3.49 (m, 4H, OCH*H* linker, OCH*H* linker 1, H3^{F1}, H4^{F1}), 3.39 – 3.34 (m, 2H, H2^{F}, H2^{F1}), 3.31 – 3.25 (m, 6H, H2^{C}, H2^{C1}, H2^{E}, H2^{E1}, H2^{A}, H2^{A1}), 3.25 – 3.20 (m, 2H, NCH_2 linker 1),

3.16 (t, J = 7.0 Hz, 2H, $NC\mathit{H}_2$ linker), 3.00 (t, J = 7.5 Hz, 2H, $C\mathit{H}_2$-triazole), 2.78 – 2.71 (m, 1H, H4^{G}), 2.61 (t, J = 7.8 Hz, 2H, $C\mathit{H}_2CONH$), 2.53 – 2.39 (m, 5H, $C\mathit{H}_2C{\equiv}CH$, $C\mathit{H}_2CO$, $CH_2C{\equiv}C\mathit{H}$), 2.02 (s, 3H, $NHCOC\mathit{H}_3$), 1.73 – 1.30 (m, 12H, 6 × $C\mathit{H}_2$ linker). ^{13}C NMR from HSQC (151 MHz, D_2O) δ 124.8 (CH triazole), 101.8 (C1^{F}, C1^{F1}), 99.5 (C1^{B}, C1^{B1}), 99.3 (C1^{D}, C1^{D1}), 97.4 (C1^{G}), 97.0 (C1^{A}, C1^{A1}), 96.5 (C1^{E}, C1^{E1}), 96.1 (C1^{C}, C1^{C1}), 76.7 (C2^{B}, C2^{B1}, C5^{F}, C5^{F1}, C4^{F}, C4^{E}, C4^{E1}, C4^{C}, C4^{C1}, C3^{F}, C4^{A}, C4^{A1}), 76.0 (C2^{D}, C2^{D1}, C4^{B}, C4^{B1}, C4^{D}, H4^{D1}), 75.0 (C3^{F1}), 73.2 (C2^{F}, C2^{F1}), 71.9 (C4^{F1}), 70.5 ($C{\equiv}\underline{C}H$), 69.9 (C5^{B1}, C5^{B}, C4^{G}), 69.6 (C3^{G}, C3^{E}, C3^{E1}, C3^{C}, C3^{C1}, C3^{A}, C3^{A1}), 69.5 (C3^{B}, C3^{B1}, C3^{D}, C3^{D1}), 69.3 (C5^{D}, C5^{D1}), 68.9 (C5^{E}, C5^{E1}, C5^{G}, C5^{C}, C5^{C1}, C5^{A}, C5^{A1}), 68.5 ($O\underline{C}H_2$ linker 1), 67.0 (C6^{A}, C6^{A1}), 66.5 (C6^{C}, C6^{C1}), 65.9 (C6^{E}, C6^{E1}), 62.5 ($O\underline{C}H_2$ linker), 57.8 (C2^{A}, C2^{A1}, C2^{C}, C2^{C1}, C2^{E}, C2^{E1}), 53.4 (C2^{G}), 49.7 (C6^{G}), 39.3 (2 × $N\underline{C}H_2$ linker), 35.2 ($\underline{C}H_2CONH$), 34.8 ($\underline{C}H_2CO$), 28.1 (2 × $\underline{C}H_2$ linker, 2 × $\underline{C}H_2$ linker 1), 22.6 ($\underline{C}H_2$ linker, $\underline{C}H_2$ linker 1), 21.8 ($NHCO\underline{C}H_3$), 21.1 ($\underline{C}H_2$-triazole), 14.5 ($\underline{C}H_2C{\equiv}CH$). ESI-MS (neg): m/z calculated for $C_{100}H_{157}N_{12}O_{116}S_{16}$ [M-22Na+19H]$^{3-}$: 1298.0779; found: 1298.0160.

^{1}H NMR (600 MHz, D_2O)

	H1	H2	H3	H4	H5	H6
**A/A1**	5.13	3.28	3.65	3.73	4.00	4.36, 4.31
**B/B1**	5.18	4.31	4.18	4.13	4.75	–
**C/C1**	5.46	3.28	3.65	3.78	4.02	4.41, 4.28
**D/D1**	5.22	4.34	4.20	4.10	4.84	–
**E/E1**	5.42	3.28	3.72	3.77	4.12	4.48, 4.24
**F**	4.61	3.36	3.73	3.77	3.81	–
**F1**	4.61	3.36	3.54	3.51	3.77	–
**G**	5.34 (d, J = 2.8 Hz)	3.68	3.72	2.75	4.10	4.85, 4.58

^{13}C NMR from HSQC (151 MHz, D_2O)

	**C1**	**C2**	**C3**	**C4**	**C5**	**C6**
**A/A1**	97.0	57.8	69.6	76.7	68.9	67.0
**B/B1**	99.5	76.7	69.5	76.0	69.9	–
**C/C1**	96.1	57.8	69.6	76.7	68.9	66.5
**D/D1**	99.3	76.0	69.5	76.0	69.3	–
**E/E1**	96.5	57.8	69.6	76.7	68.9	65.9
**F**	101.8	73.2	76.7	76.7	76.7	–
**F1**	101.8	73.2	75.0	71.9	76.7	–
**G**	97.4	53.4	69.6	69.9	68.9	49.7

5-Aminopentyl *O*-(2-acetamido-6-azido-2-deoxy-α-D-glucopyranosyl)-(1→4)-*O*-(β-D-glucopyranosyluronate)-(1→4)-*O*-(2-sulfamino-6-*O*-sulfate-2-deoxy-α-D-glucopyranosyl)-(1→4)-*O*-(2-*O*-sulfate-α-L-idopyranosyluronate)-(1→4)-*O*-(2-sulfamino-6-*O*-sulfate-2-deoxy-α-D-glucopyranosyl)-(1→4)-*O*-(2-*O*-sulfate-α-L-idopyranosyluronate)-(1→4)-*O*-2-sulfamino-6-*O*-sulfate-2-deoxy-α-D-glucopyranosyl) - *O*-[*N*-ethyl-3-(1*H*-1,2,3-triazol-4-yl)propanamide]-(1→6)-*N*-(2-acetamido-2-deoxy-α-D-glucopyranosyl)-(1→4)-*O*-(β-D-glucopyranosyluronate)-(1→4)-*O*-(2-sulfamino-6-*O*-sulfate-2-deoxy-α-D-glucopyranosyl)-(1→4)-*O*-(2-*O*-sulfate-α-L-idopyranosyluronate)-(1→4)-*O*-(2-sulfamino-6-*O*-sulfate-2-deoxy-α-D-glucopyranosyl)-(1→4)-*O*-(2-*O*-sulfate-α-L-idopyranosyluronate)-(1→4)-*O*-2-sulfamino-6-*O*-sulfate-2-deoxy-α-D-glucopyranoside, sodium salt (13).

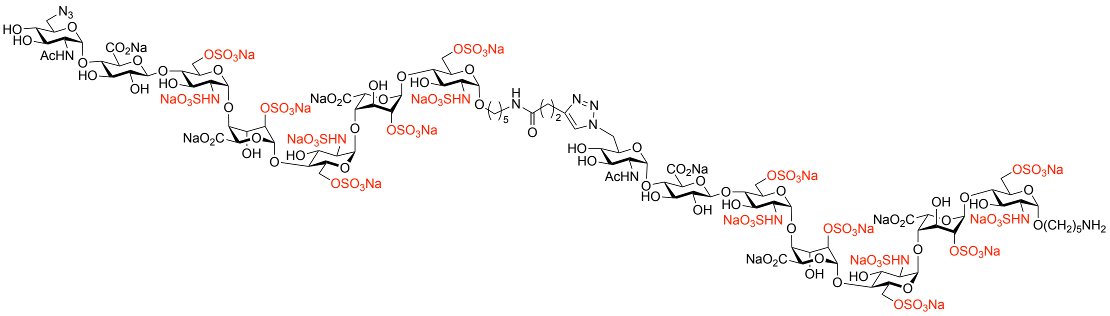

Compound **4** (5.7 mg, 1.32 µmol) was subjected to installation of unnatural α (1→4) 6-azido-GlcNAc according to the general procedure to give the title compound **13** as a white powder (3.5 mg, 58%). ^{1}H NMR (600 MHz, D_2O) δ 7.85 (s, 1H, C*H* triazole), 5.48 – 5.44 (m, 2H, H1^{C}, H1^{C1}), 5.44 – 5.40 (m, 3H, H1^{E}, H1^{E1}, H1^{G1}), 5.34 (d, *J* = 3.5 Hz, 1H, H1^{G}), 5.32 (d, *J* = 3.5 Hz, 1H, H1^{B}), 5.21 (bs, 2H, H1^{D}, H1^{D1}), 5.17 (bs, 1H, H1^{B1}), 5.15 – 5.12 (m, 2H, H1^{A}, H1^{A1}), 4.85 – 4.73 (m, 4H, H6a^{G}, H5^{D}, H5^{D1}, H5^{B1}), 4.69 (d, *J* = 2.9 Hz, 1H, H5^{B}), 4.63 – 4.55 (m, 3H, H6b^{G}, H1^{F}, H1^{F1}), 4.52 – 4.46 (m, 2H, H6a^{E}, H6a^{E1}), 4.43 – 4.15 (m, 18H, H6a^{C}, H6a^{C1}, H6a^{A}, H6a^{A1}, H6b^{A}, H6b^{A1}, H6b^{C}, H6b^{C1}, H6b^{E}, H6b^{E1}, H2^{D}, H2^{D1}, H2^{B}, H2^{B1}, H3^{D}, H3^{D1}, H3^{B}, H3^{B1}), 4.15 – 3.97 (m, 11H, H5^{E}, H5^{E1}, H5^{G}, H4^{B}, H4^{B1}, H4^{D}, H4^{D1}, H5^{A}, H5^{C}, H5^{C1}, H5^{A1}), 3.93 – 3.88 (m, 2H, H2^{G1}, H5^{G1}), 3.87 – 3.46 (m, 28H, H3^{A}, OCH*H* linker, OCH*H* linker 1, H3^{G}, H3^{G1}, H3^{E}, H3^{E1}, H2^{G}, H6a^{G1}, H6b^{G1}, H3^{C}, H3^{C1}, H3^{A1}, OCH*H* linker, OCH*H* linker 1, H5^{F}, H5^{F1}, H4^{F}, H4^{F1}, H3^{F}, H3^{F1}, H4^{E}, H4^{E1}, H4^{C}, H4^{C1}, H4^{A}, H4^{A1}, H4^{G1}), 3.39 – 3.34 (m, 2H, H2^{F}, H2^{F1}), 3.32 – 3.23 (m, 6H, H2^{C}, H2^{C1}, H2^{E}, H2^{E1}, H2^{A}, H2^{A1}), 3.16 (t, *J* = 7.0 Hz, 2H, NCH_2 linker 1), 3.04 (t, *J* = 7.3 Hz, 2H, NCH_2 linker), 3.00 (t, *J* = 7.7 Hz, 2H, CH_2-triazole), 2.77 – 2.72 (m, 1H, H4^{G}), 2.61 (t, *J* = 7.8 Hz, 2H, CH_2CONH), 2.06 (s, 3H, NHCOCH_3), 2.02 (s, 3H, NHCOCH_3), 1.79 – 1.29 (m, 12H, 6 × CH_2 linker). ^{13}C NMR from HSQC (151 MHz, D_2O) δ 124.8 (CH triazole), 101.6 (C1^{F}, C1^{F1}), 99.5 (C1^{B1}), 99.3 (C1^{D}, C1^{D1}), 97.9 (C1^{B}), 97.50 (C1^{G}), 97.0 (C1^{A}, C1^{A1}), 96.7 (C1^{E}, C1^{E1}, C1^{G1}), 96.1 (C1^{C}, C1^{C1}), 76.7 (C2^{B}, C2^{B1}, C5^{F}, C5^{F1}, C4^{F}, C4^{F1}, C4^{E}, C4^{E1}, C4^{C}, C4^{C1}, C3^{F}, C3^{F1}, C4^{A}, C4^{A1}), 76.1 (C2^{D}, C2^{D1}, C4^{B}, C4^{B1}, C4^{D}, C4^{D1}), 73.4 (C2^{F}, C2^{F1}), 70.6 (C5^{G1}), 70.4 (C4^{G1}, C3^{G}, C3^{G1}), 70.0 (C5^{B1}, C5^{B}), 69.8 (C3^{E}, C3^{E1}, C3^{C}, C3^{C1}, C3^{A1}, C4^{G}), 69.5 (C3^{B}, C3^{B1}, C3^{D}, C3^{D1}), 69.3 (C5^{D}, C5^{D1}), 69.1 (C5^{E}, C5^{E1}, C5^{G}, C5^{A}, C5^{C}, C5^{C1}, C5^{A1}), 68.8 (C3^{A}), 68.6 (O<u>C</u>H_2 linker 1), 68.0 (O<u>C</u>H_2 linker), 67.0 (C6^{A},

$C6^{A1}$), 66.4 ($C6^{C}$, $C6^{C1}$), 65.8 ($C6^{E}$, $C6^{E1}$), 57.8 ($C2^{A}$, $C2^{A1}$, $C2^{C}$, $C2^{C1}$, $C2^{E}$, $C2^{E1}$), 53.6 ($C2^{G}$, $C2^{G1}$), 50.5 ($C6^{G1}$), 49.7 ($C6^{G}$), 39.5 (2 × N$\underline{C}H_2$ linker), 35.3 ($\underline{C}H_2$CONH), 28.4 (3 × CH_2 linker 1), 26.5 ($\underline{C}H_2$ linker 1), 22.6 ($\underline{C}H_2$ linker, $\underline{C}H_2$ linker 1), 21.9 (2 × NHCO$\underline{C}H_3$), 21.2 ($\underline{C}H_2$-triazole). ESI-MS (neg): m/z calculated for $C_{104}H_{169}N_{16}O_{119}S_{16}$ $[M-22Na+19H]^{3-}$: 1352.7748; found: 1352.6670.

^{1}H NMR (600 MHz, D_2O)

	**H1**	**H2**	**H3**	**H4**	**H5**	**H6**
**A**	5.14	3.28	3.84	3.76	4.05	4.36, 4.31
**A1**	5.14	3.28	3.65	3.75	4.00	4.36, 4.31
**B**	5.31 (d, *J* = 3.5 Hz)	4.32	4.19	4.13	4.69	–
**B1**	5.18	4.32	4.18	4.13	4.76	–
**C/C1**	5.46	3.28	3.65	3.77	4.02	4.41, 4.28
**D/D1**	5.22	4.34	4.20	4.10	4.84	–
**E/E1**	5.43	3.28	3.73	3.77	4.12	4.48, 4.24
**F/F1**	4.61	3.36	3.73	3.77	3.81	–
**G**	5.34 (d, *J* = 3.5 Hz)	3.68	3.72	2.75	4.11	4.85, 4.58
**G1**	5.43	3.91	3.73	3.49	3.89	3.65

^{13}C NMR from HSQC (151 MHz, D_2O)

	**C1**	**C2**	**C3**	**C4**	**C5**	**C6**
**A**	97.0	57.8	68.8	76.7	69.1	67.0
**A1**	97.0	57.8	69.8	76.7	69.1	67.0
**B**	97.9	76.7	69.5	76.1	70.0	–
**B1**	99.5	76.7	69.5	76.1	70.0	–
**C/C1**	96.1	57.8	69.8	76.7	69.1	66.4

**D/D1**	99.3	76.1	69.5	76.1	69.3	–
**E/E1**	96.7	57.8	69.8	76.7	69.1	65.8
**F/F1**	101.6	73.4	76.7	76.7	76.7	–
**G**	97.5	53.6	70.4	69.8	69.1	49.7
**G1**	96.7	53.6	70.4	70.4	70.6	50.5

5-Aminopentyl *O*-[(β-D-glucopyranosyluronate)-(1→4)-*O*-(2-sulfamino-6-*O*-sulfate-2-deoxy-α-D-glucopyranosyl)-(1→4)-*O*-(2-*O*-sulfate-α-L-idopyranosyluronate)-(1→4)-*O*-(2-sulfamino-6-*O*-sulfate-2-deoxy-α-D-glucopyranosyl)-(1→4)-*O*-(2-*O*-sulfate-α-L-idopyranosyluronate)-(1→4)-*O*-2-sulfamino-6-*O*-sulfate-2-deoxy-α-D-glucopyranosyl) - *O*-(*N*-ethyl-3-(1*H*-1,2,3-triazol-4-yl)propanamide)-(1→6)-*N*-(2-acetamido-2-deoxy-α-D-glucopyranosyl)-(1→4)-*O*-]$_2$-(β-D-glucopyranosyluronate)-(1→4)-*O*-(2-sulfamino-6-*O*-sulfate-2-deoxy-α-D-glucopyranosyl)-(1→4)-*O*-(2-*O*-sulfate-α-L-idopyranosyluronate)-(1→4)-*O*-(2-sulfamino-6-*O*-sulfate-2-deoxy-α-D-glucopyranosyl)-(1→4)-*O*-(2-*O*-sulfate-α-L-idopyranosyluronate)-(1→4)-*O*-2-sulfamino-6-*O*-sulfate-2-deoxy-α-D-glucopyranoside, sodium salt (5).

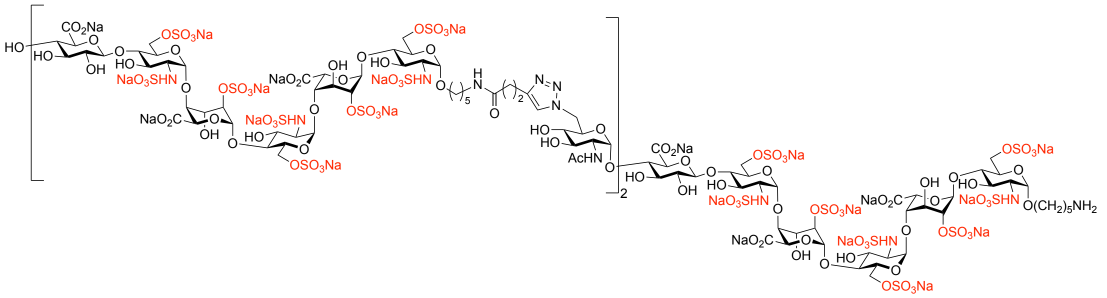

Compound **12** (1.7 mg, 0.388 μmol) and compound **3** (1.1 mg, 0.494 μmol) was subjected to copper (I)-catalyzed azide-alkyne cycloaddition (CuAAC) reaction according to the general procedure to give the title compound **5** as a white powder (1.3 mg, 51%). ^{1}H NMR (600 MHz, D_2O) δ 7.85 (s, 2H, 2 × C*H* triazole), 5.51 – 5.44 (m, 3H, H1^C, H1^{C1}, H1^{C2}), 5.44 – 5.40 (m, 3H, H1^E, H1^{E1}, H1^{E2}), 5.37 – 5.29 (m, 3H, H1^G, H1^{G1}, H1^B), 5.25 – 5.16 (m, 5H, H1^D, H1^{D1}, H1^{D2}, H1^{B1}, H1^{B2}), 5.16 – 5.12 (m, 3H, H1^A, H1^{A1}, H1^{A2}), 4.90 – 4.67 (m, 8H, H6a^G, H6a^{G1}, H5^D, H5^{D1}, H5^{D2}, H5^{B1}, H5^{B2}, H5^B), 4.63 – 4.55 (m, 5H, H6b^G, H6b^{G1}, H1^F, H1^{F1}, H1^{F2}), 4.51

– 4.46 (m, 3H, H6a^{E}, H6a^{E1}, H6^{E2}), 4.43 – 3.97 (m, 44H, H6a^{C}, H6a^{C1}, H6a^{C2}, H6a^{A}, H6a^{A1}, H6a^{A2}, H6b^{A}, H6b^{A1}, H6b^{A2}, H6b^{C}, H6b^{C1}, H6b^{C2}, H6b^{E}, H6b^{E1}, H6b^{E2}, H2^{D}, H2^{D1}, H2^{D2}, H2^{B}, H2^{B1}, H2^{B2}, H3^{D}, H3^{D1}, H3^{D2}, H3^{B}, H3^{B1}, H3^{B2}, H5^{E}, H5^{E1}, H5^{E2}, H5^{G}, H5^{G1}, H4^{B}, H4^{B1}, H4^{B2}, H4^{D}, H4^{D1}, H4^{D2}, H5^{C}, H5^{C1}, H5^{C2}, H5^{A}, H5^{A1}, H5^{A2}), 3.87 – 3.46 (m, 37H, H2^{G1}, H2^{G1}, 3 × OCH_2 linker, H3^{G}, H3^{G1}, H3^{E}, H3^{E1}, H3^{E2}, H3^{C}, H3^{C1}, H3^{C2}, H3^{A}, H3^{A1}, H3^{A2}, H5^{F}, H5^{F1}, H5^{F2}, H4^{F}, H4^{F1}, H3^{F}, H3^{F1}, H4^{E}, H4^{E1}, H4^{E2}, H4^{C}, H4^{C1}, H4^{C2}, H4^{A}, H4^{A1}, H4^{A2}, H3^{F2}, H4^{F2}), 3.40 – 3.33 (m, 3H, H2^{F}, H2^{F1}, H2^{F2}), 3.33 – 3.23 (m, 9H, H2^{C}, H2^{C1}, H2^{C2}, H2^{E}, H2^{E1}, H2^{E2}, H2^{A}, H2^{A1}, H2^{A2}), 3.21 – 3.12 (m, 4H, 2 × NCH_2 linker), 3.08 – 2.96 (m, 6H, NCH_2 linker, 2 × CH_2-triazole), 2.78 – 2.70 (m, 2H, H4^{G}, H4^{G1}), 2.66 – 2.57 (m, 4H, 2 × CH_2CONH), 2.02 (s, 6H, 2 × NHCOCH_3), 1.77 – 1.29 (m, 18H, 9 × CH_2 linker). ^{13}C NMR from HSQC (151 MHz, D_2O) δ 124.77 (2 × CH triazole), 101.7 (C1^{F}, C1^{F1}, C1^{F2}), 99.4 (C1^{B1}, C1^{B2}), 99.1 (C1^{D}, C1^{D1}, C1^{D2}), 97.4 (C1^{B}, C1^{G}, C1^{G1}), 97.0 (C1^{A}, C1^{A1}, C1^{A2}), 96.4 (C1^{C}, C1^{C1}, C1^{C2}, C1^{E}, C1^{E1}, C1^{E2}), 76.6 (C2^{B}, C2^{B1}, C2^{B2}, C5^{F}, C5^{F1}, C5^{F2}, C4^{F}, C4^{F1}, C4^{E}, C4^{E1}, C4^{E2}, C4^{C}, C4^{C1}, C4^{C2}, C3^{F}, C3^{F1}, C4^{A}, C4^{A1}, C4^{A2}), 76.0 (C2^{D}, C2^{D1}, C2^{D2}, C4^{B}, C4^{B1}, C4^{B2}, C4^{D}, C4^{D1}, C4^{D2}), 74.8 (C3^{F2}), 73.2 (C2^{F}, C2^{F1}, C2^{F2}), 72.0 (C4^{F2}), 69.8 (C5^{B}, C5^{B1}, C5^{B2}, C3^{E}, C3^{E1}, C3^{E2}, C3^{C}, C3^{C1}, C3^{C2}, C3^{A}, C3^{A1}, C3^{A2}, C3^{G}, C3^{G1}, C4^{G}, C4^{G1}), 69.5 (C3^{B}, C3^{B1}, C3^{B2}, C3^{D}, C3^{D1}, C3^{D2}), 69.3 (C5^{D}, C5^{D1}, C5^{D2}), 69.1 (C5^{E}, C5^{E1}, C5^{E2}, C5^{G}, C5^{G1}, C5^{A}, C5^{A1}, C5^{A2}, C5^{C}, C5^{C1}, C5^{C2}), 68.6 (2 × O$\underline{C}H_2$ linker), 68.0 (O$\underline{C}H_2$ linker), 67.0 (C6^{A}, C6^{A1}, C6^{A2}), 66.4 (C6^{C}, C6^{C1}, C6^{C2}), 65.8 (C6^{E}, C6^{E1}, C6^{E2}), 57.8 (C2^{A}, C2^{A1}, C2^{A2}, C2^{C}, C2^{C1}, C2^{C2}, C2^{E}, C2^{E1}, C2^{E2}), 53.4 (C2^{G}, C2^{G1}), 49.7 (C6^{G}, C6^{G1}), 39.4 (3 × N$\underline{C}H_2$ linker), 35.3 (2 × $\underline{C}H_2$CONH), 28.2 (6 × $\underline{C}H_2$ linker), 22.7 (3 × $\underline{C}H_2$ linker), 22.0 (2 × NHCO$\underline{C}H_3$), 21.2 (2 × $\underline{C}H_2$-triazole). ESI-MS (neg): m/z calculated for $C_{149}H_{235}N_{20}Na_3O_{175}S_{24}$ [M-30Na+26H]$^{4-}$: 1485.8277; found: 1485.7873.

^{1}H NMR (600 MHz, D_2O)

	**H1**	**H2**	**H3**	**H4**	**H5**	**H6**
**A**	5.14	3.28	3.82	3.75	4.05	4.36, 4.31
**A1/A2**	5.14	3.28	3.65	3.74	4.00	4.36, 4.31
**B**	5.31	4.32	4.19	4.13	4.69	–

**B1/B2**	5.18	4.32	4.18	4.13	4.76	–
**C/C1/C2**	5.46	3.28	3.65	3.77	4.02	4.41, 4.28
**D/D1/D2**	5.22	4.34	4.20	4.10	4.84	–
**E/E1/E2**	5.43	3.28	3.71	3.78	4.11	4.48, 4.24
**F/F1**	4.60	3.36	3.73	3.77	3.81	–
**F2**	4.60	3.36	3.54	3.51	3.77	
**G/G1**	5.34	3.68	3.72	2.75	4.12	4.87, 4.59

^{13}C NMR from HSQC (151 MHz, D_2O)

	**C1**	**C2**	**C3**	**C4**	**C5**	**C6**
**A/A1/A2**	97.0	57.8	69.8	76.6	69.1	67.0
**B**	97.4	76.6	69.5	76.0	69.8	–
**B1/B2**	99.4	76.6	69.5	76.0	69.8	–
**C/C1/C2**	96.4	57.8	69.8	76.6	69.1	66.4
**D/D1/D2**	99.1	76.0	69.5	76.0	69.3	–
**E/E1/E2**	96.4	57.8	69.8	76.6	69.1	65.8
**F/F1**	101.7	73.2	76.6	76.6	76.6	–
**F2**	101.7	73.2	74.8	72.0	76.6	–
**G/G1**	97.4	53.4	69.8	69.8	69.1	49.7

5-Aminopentyl *O*-[(β-D-glucopyranosyluronate)-(1→4)-*O*-(2-sulfamino-6-*O*-sulfate-2-deoxy-α-D-glucopyranosyl)-(1→4)-*O*-(2-*O*-sulfate-α-L-idopyranosyluronate)-(1→4)-*O*-(2-sulfamino-6-*O*-sulfate-2-deoxy-α-D-glucopyranosyl)-(1→4)-*O*-(2-*O*-sulfate-α-L-idopyranosyluronate)-(1→4)-*O*-2-sulfamino-6-*O*-sulfate-2-deoxy-α-D-glucopyranosyl) -*O*-(*N*-ethyl-3-(1*H*-1,2,3-triazol-4-yl)propanamide)-(1→6)-*N*-(2-acetamido-2-deoxy-α-D-glucopyranosyl)-(1→4)-*O*-]$_3$-(β-D-glucopyranosyluronate)-(1→4)-*O*-(2-sulfamino-6-*O*-

sulfate-2-deoxy-α-D-glucopyranosyl)-(1→4)-*O*-(2-*O*-sulfate-α-L-idopyranosyluronate)-(1→4)-*O*-(2-sulfamino-6-*O*-sulfate-2-deoxy-α-D-glucopyranosyl)-(1→4)-*O*-(2-*O*-sulfate-α-L-idopyranosyluronate)-(1→4)-*O*-2-sulfamino-6-*O*-sulfate-2-deoxy-α-D-glucopyranoside, sodium salt (6).

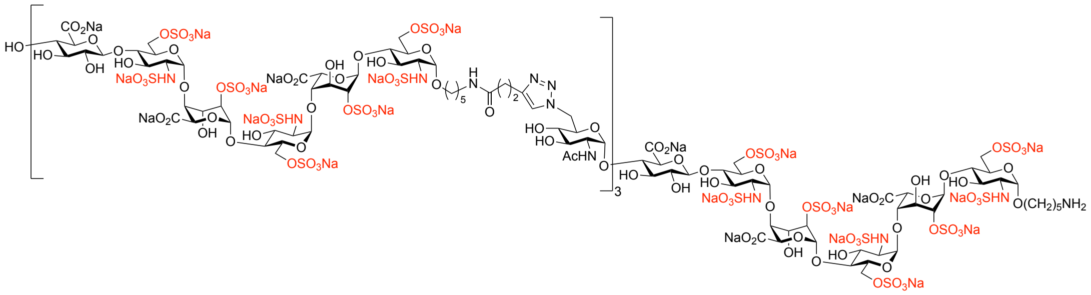

Compound **12** (2.3 mg, 0.525 μmol) and compound **13** (2.0 mg, 0.441 μmol) was subjected to copper (I)-catalyzed azide-alkyne cycloaddition (CuAAC) reaction according to the general procedure to give the title compound **6** as a white powder (1.9 mg, 48%). ^{1}H NMR (600 MHz, D_2O) δ 7.85 (s, 3H, 3 × C*H* triazole), 5.49 – 5.44 (m, 4H, H1^{C}, H1^{C1}, H1^{C2}, H1^{C3}), 5.44 – 5.40 (m, 4H, H1^{E}, H1^{E1}, H1^{E2}, H1^{E3}), 5.37 – 5.29 (m, 4H, H1^{G}, H1^{G1}, H1^{G2}, H1^{B}), 5.22 (bs, 4H, H1^{D}, H1^{D1}, H1^{D2}, H1^{D3}), 5.18 (bs, 3H, H1^{B1}, H1^{B2}, H1^{B3}), 5.16 – 5.09 (m, 4H, H1^{A}, H1^{A1}, H1^{A2}, H1^{A3}), 4.90 – 4.67 (m, 11H, H6a^{G}, H6a^{G1}, H6a^{G2}, H5^{D}, H5^{D1}, H5^{D2}, H5^{D3},H5^{B1}, H5^{B2}, H5^{B3}, H5^{B}), 4.64 – 4.55 (m, 7H, H6b^{G}, H6b^{G1}, H6b^{G2}, H1^{F}, H1^{F1}, H1^{F2}, H1^{F3}), 4.52 – 4.45 (m, 4H, H6a^{E}, H6a^{E1}, H6^{E2}, H6a^{E3}), 4.43 – 3.97 (m, 59H, H6a^{C}, H6a^{C1}, H6a^{C2}, H6a^{C3}, H6a^{A}, H6a^{A1}, H6a^{A2}, H6a^{A3}, H6b^{A}, H6b^{A1}, H6b^{A2}, H6b^{A3}, H6b^{C}, H6b^{C1}, H6b^{C2}, H6b^{C3}, H6b^{E}, H6b^{E1}, H6b^{E2}, H6b^{E3}, H2^{D}, H2^{D1}, H2^{D2}, H2^{D3}, H2^{B}, H2^{B1}, H2^{B2}, H2^{B3}, H3^{D}, H3^{D1}, H3^{D2}, H3^{D3}, H3^{B}, H3^{B1}, H3^{B2}, H3^{B3}, H5^{E}, H5^{E1}, H5^{E2}, H5^{E3}, H5^{G}, H5^{G1}, H5^{G2}, H4^{B}, H4^{B1}, H4^{B2}, H4^{B3}, H4^{D}, H4^{D1}, H4^{D2}, H4^{D3}, H5^{C}, H5^{C1}, H5^{C2}, H5^{C3}, H5^{A}, H5^{A1}, H5^{A2}, H5^{A3}), 3.87 – 3.48 (m, 50H, H2^{G1}, H2^{G1}, H2^{G2}, 4 × OC*H*2 linker, H3^{G}, H3^{G1}, H3^{G2}, H3^{E}, H3^{E1}, H3^{E2}, H3^{E3}, H3^{C}, H3^{C1}, H3^{C2}, H3^{C3}, H3^{A}, H3^{A1}, H3^{A2}, H3^{A3}, H5^{F}, H5^{F1}, H5^{F2}, H5^{F3}, H4^{F}, H4^{F1}, H4^{F2}, H3^{F}, H3^{F1}, H3^{F2}, H4^{E}, H4^{E1}, H4^{E2}, H4^{E3}, H4^{C}, H4^{C1}, H4^{C2}, H4^{C3}, H4^{A}, H4^{A1}, H4^{A2}, H4^{A3}, H3^{F3}, H4^{F3}), 3.40 – 3.33 (m, 4H, H2^{F}, H2^{F1}, H2^{F2}, H2^{F3}), 3.33 – 3.23 (m, 12H, H2^{C}, H2^{C1}, H2^{C2}, H2^{C3}, H2^{E}, H2^{E1}, H2^{E2}, H2^{E3}, H2^{A}, H2^{A1}, H2^{A2}, H2^{A3}), 3.20 – 3.12 (m, 6H, 3 × NC*H*2 linker), 3.08 – 2.96 (m, 8H, NC*H*2 linker, 3 × C*H*2-

triazole), 2.79 – 2.71 (m, 3H, $H4^{G}$, $H4^{G1}$, $H4^{G2}$), 2.66 – 2.56 (m, 6H, 3 × CH_2CONH), 2.03 (s, 9H, 3 × NHCOCH_3), 1.77 – 1.28 (m, 24H, 12 × CH_2 linker). ^{13}C NMR from HSQC (151 MHz, D_2O) δ 124.7 (3 × CH triazole), 101.7 ($C1^{F}$, $C1^{F1}$, $C1^{F2}$, $C1^{F3}$), 99.4 ($C1^{B1}$, $C1^{B2}$, $C1^{B3}$), 99.1 ($C1^{D}$, $C1^{D1}$, $C1^{D2}$, $C1^{D3}$), 97.4 ($C1^{B}$, $C1^{G}$, $C1^{G1}$, $C1^{G2}$), 97.0 ($C1^{A}$, $C1^{A1}$, $C1^{A2}$, $C1^{A3}$), 96.4 ($C1^{C}$, $C1^{C1}$, $C1^{C2}$, $C1^{C3}$, $C1^{E}$, $C1^{E1}$, $C1^{E2}$, $C1^{E3}$), 76.5 ($C2^{B}$, $C2^{B1}$, $C2^{B2}$, $C2^{B3}$, $C5^{F}$, $C5^{F1}$, $C5^{F2}$, $C5^{F3}$, $C4^{F}$, $C4^{F1}$, $C4^{F2}$, $C4^{E}$, $C4^{E1}$, $C4^{E2}$, $C4^{E3}$, $C4^{C}$, $C4^{C1}$, $C4^{C2}$, $C4^{C3}$, $C3^{F}$, $C3^{F1}$, $C3^{F2}$, $C4^{A}$, $C4^{A1}$, $C4^{A2}$, $C4^{A3}$), 75.9 ($C2^{D}$, $C2^{D1}$, $C2^{D2}$, $C2^{D3}$, $C4^{B}$, $C4^{B1}$, $C4^{B2}$, $C4^{B3}$, $C4^{D}$, $C4^{D1}$, $C4^{D2}$, $C4^{D3}$), 75.0 ($C3^{F3}$), 73.2 ($C2^{F}$, $C2^{F1}$, $C2^{F2}$, $C2^{F3}$), 71.9 ($C4^{F3}$), 69.9 ($C4^{G}$, $C4^{G1}$, $C4^{G2}$), 69.7 ($C5^{B}$, $C5^{B1}$, $C5^{B2}$, $C5^{B3}$, $C3^{G}$, $C3^{G1}$, $C3^{G2}$, $C3^{E}$, $C3^{E1}$, $C3^{E2}$, $C3^{E3}$, $C3^{C}$, $C3^{C1}$, $C3^{C2}$, $C3^{C3}$, $C3^{A}$, $C3^{A1}$, $C3^{A2}$, $C3^{A3}$), 69.4 ($C3^{B}$, $C3^{B1}$, $C3^{B2}$, $C3^{B3}$, $C3^{D}$, $C3^{D1}$, $C3^{D2}$, $C3^{D3}$), 69.3 ($C5^{D}$, $C5^{D1}$, $C5^{D2}$, $C5^{D3}$), 69.1 ($C5^{E}$, $C5^{E1}$, $C5^{E2}$, $C5^{E3}$, $C5^{G}$, $C5^{G1}$, $C5^{G2}$, $C5^{A}$, $C5^{A1}$, $C5^{A2}$, $C5^{A3}$, $C5^{C}$, $C5^{C1}$, $C5^{C2}$, $C5^{C3}$), 68.4 (3 × O<u>C</u>H_2 linker), 67.8 (O<u>C</u>H_2 linker), 67.0 ($C6^{A}$, $C6^{A1}$, $C6^{A2}$, $C6^{A3}$), 66.4 ($C6^{C}$, $C6^{C1}$, $C6^{C2}$, $C6^{C3}$), 65.8 ($C6^{E}$, $C6^{E1}$, $C6^{E2}$, $C6^{E3}$), 57.7 ($C2^{A}$, $C2^{A1}$, $C2^{A2}$, $C2^{A3}$, $C2^{C}$, $C2^{C1}$, $C2^{C2}$, $C2^{C3}$, $C2^{E}$, $C2^{E1}$, $C2^{E2}$, $C2^{E3}$), 53.4 ($C2^{G}$, $C2^{G1}$, $C2^{G2}$), 49.7 ($C6^{G}$, $C6^{G1}$, $C6^{G2}$), 39.4 (4 × N<u>C</u>H_2 linker), 35.3 (3 × <u>C</u>H_2CONH), 28.0 (8 × <u>C</u>H_2 linker), 22.6 (4 × <u>C</u>H_2 linker), 21.8 (3 × NHCO<u>C</u>H_3), 21.1 (3 × <u>C</u>H_2-triazole). ESI-MS (neg): m/z calculated for $C_{203}H_{316}N_{28}Na_7O_{235}S_{32}$ $[M-37Na+32H]^{5-}$: 1618.6807; found: 1618.6693.

^{1}H NMR (600 MHz, D_2O)

	**H1**	**H2**	**H3**	**H4**	**H5**	**H6**
**A**	5.14	3.28	3.82	3.75	4.05	4.36, 4.31
**A1/A2/A3**	5.14	3.28	3.65	3.74	4.00	4.36, 4.31
**B**	5.31	4.32	4.19	4.13	4.69	–
**B1/B2/B3**	5.18	4.32	4.18	4.13	4.77	–
**C/C1/C2/C3**	5.46	3.28	3.65	3.77	4.02	4.41, 4.28
**D/D1/D2/D3**	5.22	4.34	4.20	4.10	4.83	–
**E/E1/E2/E3**	5.43	3.28	3.71	3.78	4.11	4.48, 4.24
**F/F1/F2**	4.60	3.36	3.73	3.77	3.81	–

**F3**	4.60	3.36	3.54	3.51	3.77	
**G/G1/G2**	5.34	3.68	3.72	2.75	4.12	4.87, 4.59

^{13}C NMR from HSQC (151 MHz, D_2O)

	**C1**	**C2**	**C3**	**C4**	**C5**	**C6**
**A/A1/A2/A3**	97.0	57.7	69.7	76.5	69.1	67.0
**B**	97.4	76.5	69.4	75.9	69.7	–
**B1/B2/B3**	99.4	76.5	69.4	75.9	69.7	–
**C/C1/C2/C3**	96.4	57.7	69.7	76.5	69.1	66.4
**D/D1/D2/D3**	99.1	75.9	69.4	75.9	69.3	–
**E/E1/E2/E3**	96.4	57.7	69.7	76.5	69.1	65.8
**F/F1/F2**	101.7	73.2	76.5	76.5	76.5	–
**F3**	101.7	73.2	75.0	71.9	76.5	–
**G/G1/G2**	97.4	53.4	69.7	69.9	69.1	49.7

3. Materials for SPR and Vero E6 inhibition experiments

High grade heparin (HG-Hep, Mw 15,700) for SPR was obtained from Iduron Ltd, UK (#HEP-HG 100). The SARS-CoV-2 RBD modified by an His6-tag and trimerized, soluble spike with His6-tag were expressed in HEK cells and purified as previously described.[4,5] The open reading frame of the SARS-CoV RBDs were a gift from Rogier W. Sanders, Amsterdam Medical Center. Recombinant SARS-CoV-2 RBD, containing amino acid residue 319-541, was expressed in HEK293T cells having a C-terminal mOrange fusion, trimerization domain and twinstrep as previously described.[6,7]

4. Surface plasma resonance (SPR) experiments

Preparation of heparin chip: Heparin functionalized sensor chip was prepared according to our previously published protocol.[7,8] Briefly, the surface of CM5 chip (Biacore Inc., GE Healthcare) was activated using freshly mixed N-hydroxysuccinimide (NHS; 100 mM) and 1-(3-dimethylaminopropyl)-ethylcarbodiimide (EDC; 350 mM) (1/1, v/v) in water. Next, streptavidin (50 μg/mL, Invitrogen) in aqueous NaOAc (10 mM, pH 4.5) was passed over the chip surface until a ligand density of approximately 2000 RU was achieved. The remaining NHS-activated esters were quenched by aqueous ethanolamine (1.0 M, pH 8.5). Next, biotin-heparin (50 μg/mL) was passed over one of the flow channels at a flow rate of 10 μL/min for 60 sec resulting in a response of 100 RU. Next, the reference and modified flow cells were washed with three consecutive injections of 60 sec with 2.0 M NaCl. HBS-EP (0.01 M HEPES, 150 mM NaCl, 3 mM EDTA, 0.005% polysorbate 20; pH 7.4) was used as the running buffer for the immobilization, kinetic studies, and competition assays. To correct for non-specific interactions, upon reaching desired response units (RUs) of immobilized heparin, both active and control flow cells were treated with biocytin (a biotin-lysine conjugate, 100 μg/mL, 2 x 60 sec injection, 30 μL/min).

SARS-CoV-2 spike protein competition assays: Spike trimer (100 nM) alone or in the presence of compound (**1**, **4**, **5**, and **6**) at various concentrations (ranging from 10 μM to 0.02

μM, 2-fold dilutions, Figures S3-S6) was flowed over heparin chip. In addition, control run was performed in the presence of unfractionated heparin (UFH, at concentration ranging from 1000 μg/mL to 0.001 μg/mL, 10-fold dilutions, Figure S7). The samples were diluted in HBS-EP (0.01 M HEPES, 150 mM NaCl, 3 mM EDTA, 0.005% polysorbate 20; pH 7.4) running buffer and a flow rate of 30 μL/min was employed for association (180 sec) and dissociation (600 sec) at a constant temperature of 25 °C. A 60 sec injection of 2.0 M NaCl at a flow rate of 30 μL/min was used for regeneration and achieved prior baseline status. The half-maximal inhibitory concentrations (IC_{50} values, Figures S3-S7) were calculated using dose−response equations [nonlinear regression, log(inhibitor) vs response-variable slope (four parameters)] built in Prism software 9 (GraphPad Software, Inc.). All experiments were performed two times at the minimum.

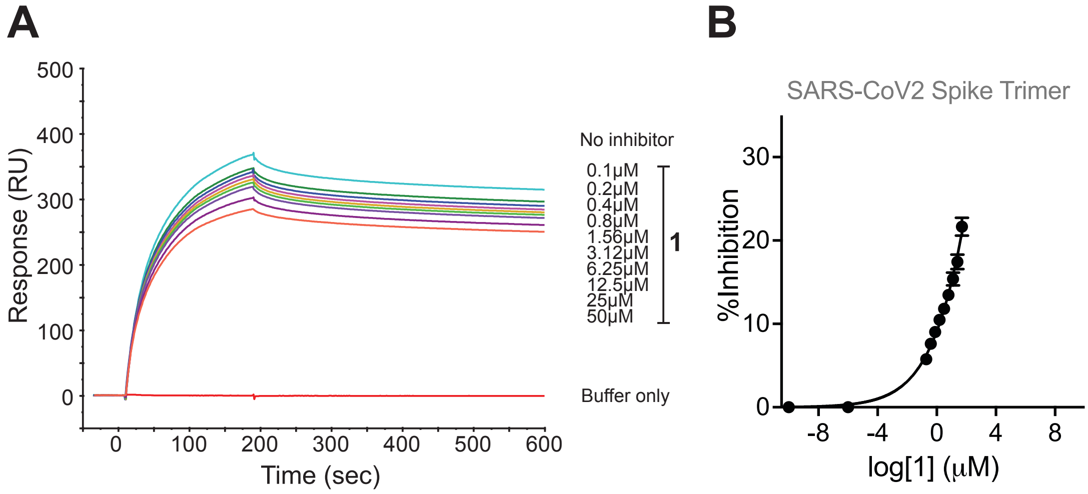

Figure S3. SARS-CoV-2 spike protein competition SPR data of compound **1**. (A) Sensorgram of SARS-CoV-2 spike trimer in the presence of **1** at various concentrations (ranging from 10 μM to 0.02 μM, 2-fold dilutions); (B) Inhibition curves of compound **1** for binding of SARS-CoV-2 spike trimer to heparin-immobilized surface.

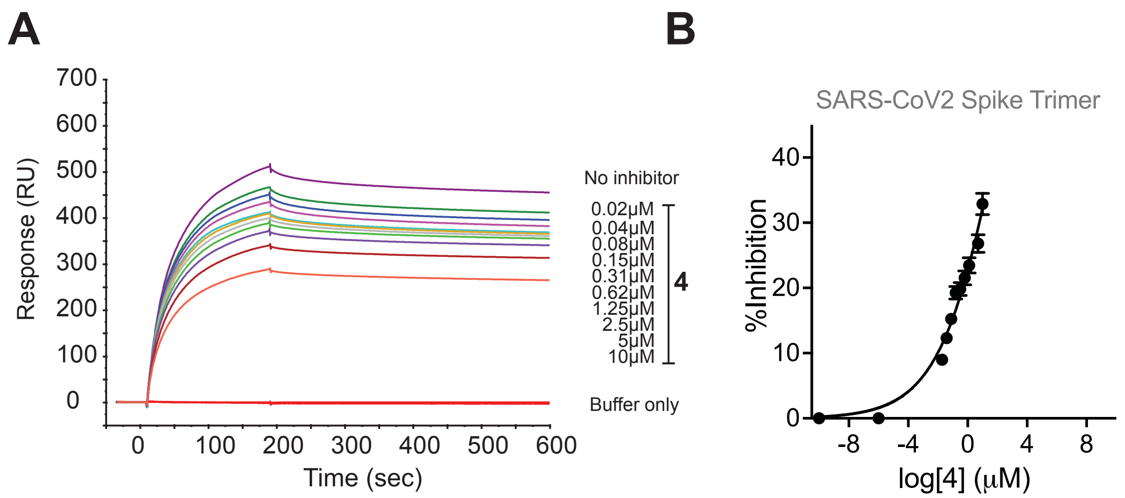

Figure S4. SARS-CoV-2 spike protein competition SPR data of compound **4**. (A) Sensorgram of SARS-CoV-2 spike trimer in the presence of **4** at various concentrations (ranging from 10 μM to 0.02 μM, 2-fold dilutions); (B) Inhibition curves of compound **4** for binding of SARS-CoV-2 spike trimer to heparin-immobilized surface.

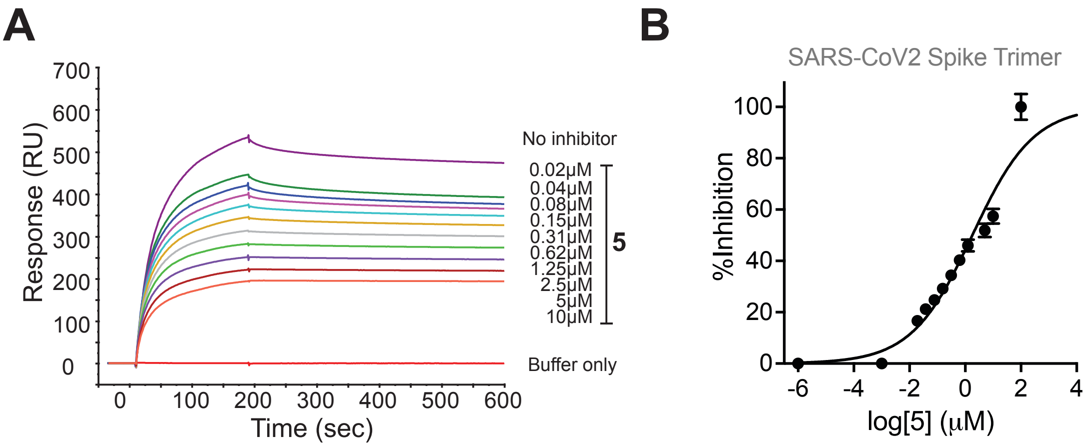

Figure S5. SARS-CoV-2 spike protein competition SPR data of compound **5**. (A) Sensorgram of SARS-CoV-2 spike trimer in the presence of **5** at various concentrations (ranging from 10 μM to 0.02 μM, 2-fold dilutions); (B) Inhibition curves of compound **5** for binding of SARS-CoV-2 spike trimer to heparin-immobilized surface.

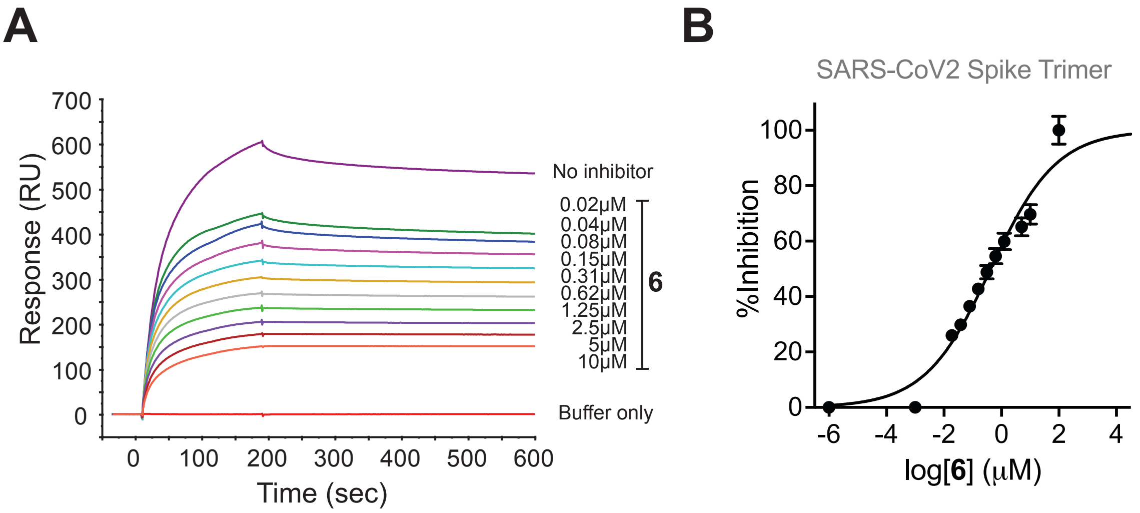

Figure S6. SARS-CoV-2 spike protein competition SPR data of compound **6**. (A) Sensorgram of SARS-CoV-2 spike trimer in the presence of **6** at various concentrations (ranging from 10 μM to 0.02 μM, 2-fold dilutions); (B) Inhibition curves of compound **6** for binding of SARS-CoV-2 spike trimer to heparin-immobilized surface.

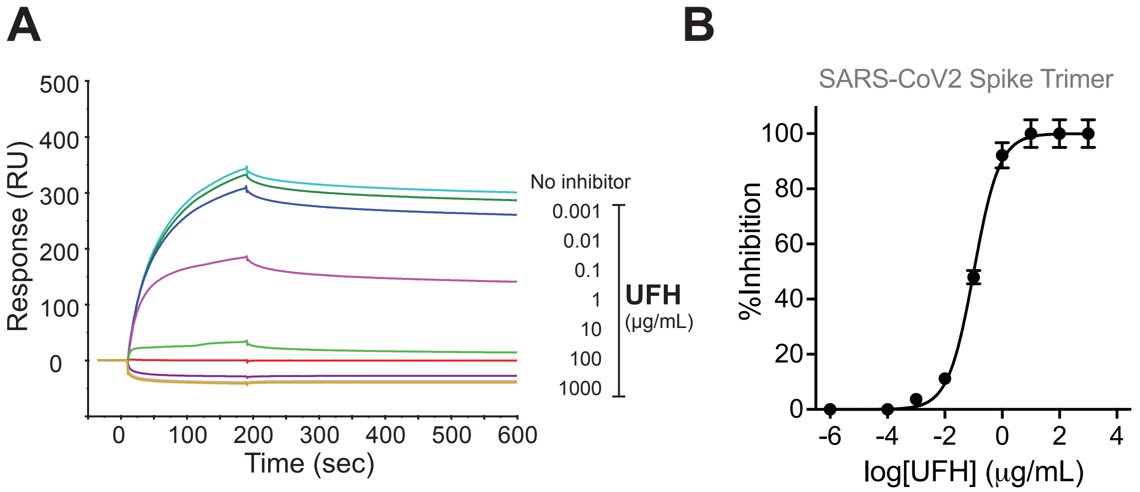

Figure S7. SARS-CoV-2 spike protein competition SPR data of UFH. (A) Sensorgram of SARS-CoV-2 spike trimer in the presence of UFH at various concentrations (at concentration ranging from 1000 μg/mL to 0.001 μg/mL, 10-fold dilutions); (B) Inhibition curves of UFH for binding of SARS-CoV-2 spike trimer to heparin-immobilized surface.

5. Microarray printing and screening

All compounds were printed on NHS-ester activated glass slides (NEXTERION® Slide H, Schott Inc.) using a Scienion sciFLEXARRAYER S3 non-contact microarray equipped with a PDC80 nozzle (Scienion Inc.). Individual samples were dissolved in sodium phosphate buffer (50 μL, 0.225 M, pH 8.5) at 100 μM and were printed in replicates of 6 with spot volume ~ 400 pL at 20 °C and 50% humidity. Each slide has 24 subarrays in a 3x8 layout. After printing, slides were incubated in a humidity chamber for 8 h and then blocked for 30 min with a 5mM ethanolamine in a Tris buffer (pH 9.0, 50 mM) at 40 °C. Blocked slides were rinsed with DI water, spun dry, and kept in a desiccator at room temperature for future use.

Screening was performed by incubating the slides with a protein solution for a certain amount of time followed by washing and drying. The buffers used in screening are TSM buffer (TSM, 20 mM Tris·Cl, pH 7.4, 150 mM NaCl, 2 mM $CaCl_2$,and 2 mM $MgCl_2$), TSM binding buffer (TSMBB, TSM buffer with 0.05% Tween-20 and 1% BSA) and TSM washing buffer (TSMWB, TSM buffer with 0.05% Tween-20). A typical washing procedure includes sequentially dipping the glass slide in TSM wash buffer (2 min, containing 0.05 % Tween 20), TSM buffer (2 min) and, water (2 x 2 min), followed by spun dry.

The slides were incubated with His-tagged proteins diluted in TSMBB at different concentrations for 1 h, followed by washing and incubation with suitable detection reagent. For His tagged proteins, a solution of AlexaFluor® 647 conjugated anti-His antibody (10 μg/mL) was used. For Strep tagged proteins, a solution of StrepMAB-Classic Oyster 645 (10 μg/mL) was used. After washing and drying, the slides were scanned using a GenePix 4000B microarray scanner (Molecular Devices) at the appropriate excitation wavelength with a resolution of 5 μM. Various gains and PMT values were employed for the scanning to ensure that all the signals were within the linear range of the scanner's detector and there was no saturation of signals. The images were analyzed using GenePix Pro 7 software (version 7.2.29.2, Molecular Devices). The data was analyzed with a home written Excel macro.[9] The highest and the lowest value of the total fluorescence intensity of the replicates were removed, and the remaining values were used to provide the mean value and standard deviation.

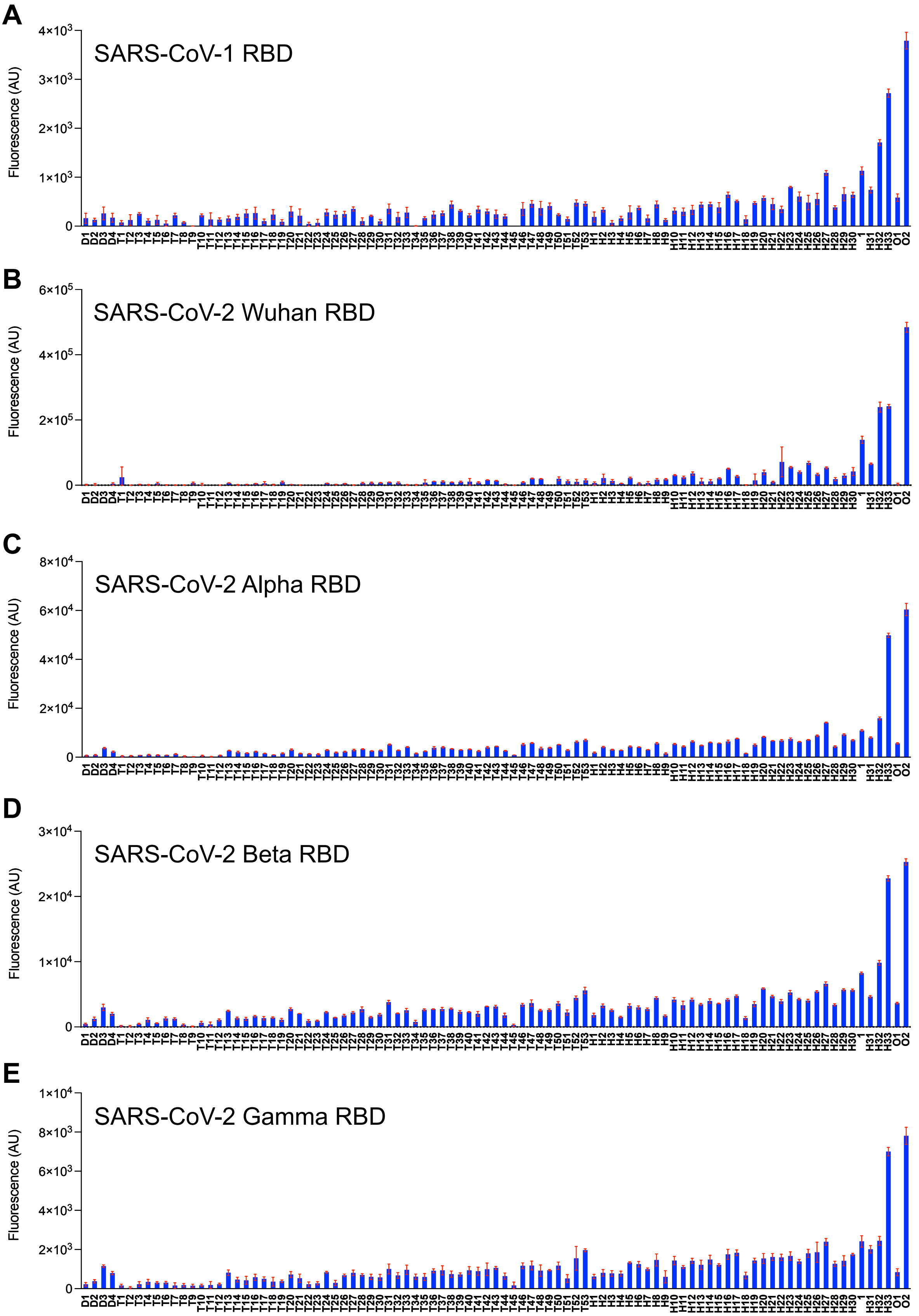
A
SARS-CoV-1 RBD
Fluorescence (AU)
B
SARS-CoV-2 Wuhan RBD
Fluorescence (AU)
C
SARS-CoV-2 Alpha RBD
Fluorescence (AU)
D
SARS-CoV-2 Beta RBD
Fluorescence (AU)
E
SARS-CoV-2 Gamma RBD
Fluorescence (AU)

F

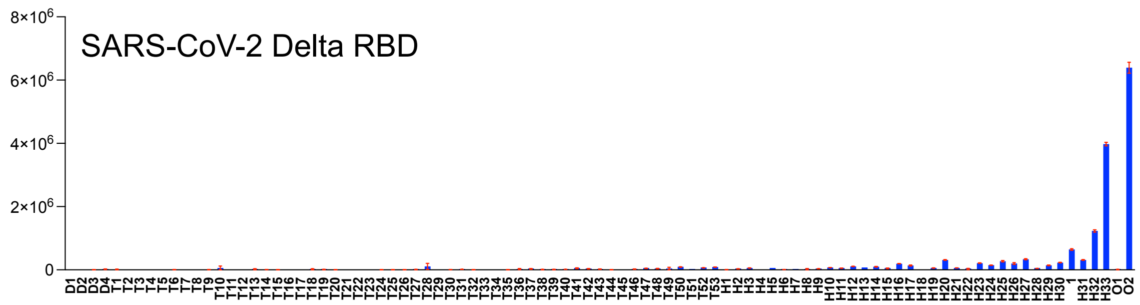

G

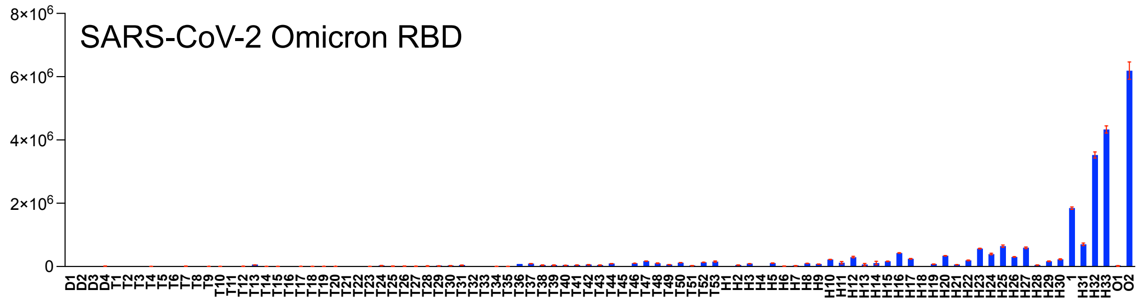

H

**D1**	IdoA-GlcNAc6S	**T28**	GlcA-GlcNAc-IdoA2S-GlcNS6S	**H6**	GlcA-GlcNS6S-GlcA-GlcNS6S-IdoA-GlcNS6S
**D2**	GlcA-GlcNAc6S	**T29**	GlcA-GlcNS-IdoA-GlcNS6S	**H7**	GlcA-GlcNS6S-IdoA-GlcNS6S-IdoA-GlcNS6S
**D3**	IdoA2S-GlcNAc6S	**T30**	IdoA-GlcNS6S-GlcA-GlcNS	**H8**	GlcA-GlcNS6S-GlcA-GlcNS3S-GlcA-GlcNS6S
**D4**	IdoA2S-GlcNS6S	**T31**	IdoA2S-GlcNAc6S-GlcA-GlcNAc6S	**H9**	GlcA-GlcNS6S-IdoA-GlcNS3S-GlcA-GlcNS6S
**T1**	GlcA-GlcNAc-GlcA-GlcNAc	**T32**	IdoA2S-GlcNS-GlcA-GlcNS	**H10**	GlcA-GlcNS6S-GlcA-GlcNS3S-IdoA-GlcNS6S
**T2**	GlcA-GlcNAc-IdoA-GlcNAc	**T33**	GlcA-GlcNS6S-IdoA-GlcNS	**H11**	GlcA-GlcNS6S-IdoA-GlcNS3S-IdoA-GlcNS6S
**T3**	GlcA-GlcNAc-IdoA2S-GlcNAc	**T34**	IdoA-GlcNS-IdoA-GlcNS6S	**H12**	GlcA-GlcNS6S-GlcA-GlcNS3S-IdoA2S-GlcNS6S
**T4**	GlcA-GlcNAc-GlcA2S-GlcNAc	**T35**	GlcA-GlcNAc6S-GlcA2S-GlcNAc6S	**H13**	GlcA-GlcNS-IdoA2S-GlcNS6S-IdoA2S-GlcNS
**T5**	GlcA-GlcNAc-IdoA-GlcNAc6S	**T36**	IdoA-GlcNS6S-GlcA-GlcNS6S	**H14**	GlcA-GlcNAc6S-IdoA2S-GlcNS6S-IdoA2S-GlcNS
**T6**	IdoA-GlcNAc6S-GlcA-GlcNAc	**T37**	GlcA-GlcNS6S-IdoA-GlcNS6S	**H15**	GlcA-GlcNS6S-GlcA-GlcNS3S6S-GlcA-GlcNS6S
**T7**	IdoA2S-GlcNAc-GlcA-GlcNAc	**T38**	GlcA-GlcNS6S-GlcA-GlcNS6S	**H16**	GlcA-GlcNS6S-IdoA-GlcNS3S6S-GlcA-GlcNS6S
**T8**	IdoA-GlcNAc-IdoA-GlcNAc6S	**T39**	IdoA-GlcNS6S-IdoA-GlcNS6S	**H17**	GlcA-GlcNS6S-IdoA2S-GlcNS6S-GlcA-GlcNS6S
**T9**	IdoA-GlcNAc6S-IdoA-GlcNAc	**T40**	GlcA-GlcNS-GlcA2S-GlcNS6S	**H18**	GlcA-GlcNS6S-GlcA-GlcNS3S6S-IdoA-GlcNS6S
**T10**	GlcA-GalNAc-GlcA-GalNAc4S	**T41**	GlcA-GlcNS-IdoA2S-GlcNS6S	**H19**	GlcA-GlcNS6S-IdoA-GlcNS3S6S-IdoA-GlcNS6S
**T11**	IdoA-GlcNS-IdoA-GlcNAc	**T42**	IdoA2S-GlcNS6S-GlcA-GlcNS	**H20**	GlcA-GlcNS6S-IdoA2S-GlcNS6S-IdoA-GlcNS6S
**T12**	IdoA-GlcNAc-IdoA-GlcNS	**T43**	IdoA2S-GlcNAc6S-IdoA2S-GlcNAc6S	**H21**	GlcA-GlcNS6S-GlcA-GlcNS6S-IdoA2S-GlcNS6S
**T13**	IdoA-GlcNAc6S-IdoA-GlcNAc6S	**T44**	IdoA-GlcNS-IdoA2S-GlcNS6S	**H22**	GlcA-GlcNS6S-IdoA-GlcNS6S-IdoA2S-GlcNS6S
**T14**	IdoA-GlcNAc6S-GlcA-GlcNAc6S	**T45**	IdoA2S-GlcNS6S-IdoA-GlcNAc6S	**H23**	GlcA-GlcNS6S-IdoA2S-GlcNS3S-GlcA-GlcNS6S
**T15**	GlcA-GlcNAc6S-IdoA-GlcNAc6S	**T46**	GlcA-GlcNS6S-IdoA2S-GlcNS6S	**H24**	GlcA-GlcNS6S-IdoA2S-GlcNS3S-IdoA-GlcNS6S
**T16**	GlcA-GlcNAc6S-GlcA-GlcNAc6S	**T47**	IdoA2S-GlcNS6S-GlcA-GlcNS6S	**H25**	GlcA-GlcNS6S-IdoA-GlcNS3S-IdoA2S-GlcNS6S
**T17**	GlcA-GlcNAc-GlcA2S-GlcNAc6S	**T48**	IdoA-GlcNS6S-IdoA2S-GlcNS6S	**H26**	GlcA-GlcNS6S-IdoA2S-GlcNS6S-IdoA2S-GlcNS
**T18**	GlcA-GlcNAc-IdoA-GlcNS6S	**T49**	GlcA-GlcNS3S-IdoA2S-GlcNS6S	**H27**	GlcA-GlcNS6S-IdoA2S-GlcNS3S6S-GlcA-GlcNS6S
**T19**	GlcA-GlcNAc-IdoA2S-GlcNAc6S	**T50**	IdoA2S-GlcNS6S-IdoA2S-GlcNS	**H28**	GlcA-GlcNS6S-IdoA2S-GlcNS3S6S-IdoA-GlcNS6S
**T20**	IdoA2S-GlcNAc6S-GlcA-GlcNAc	**T51**	GlcA-GlcNS6S-GlcA2S-GlcNS6S	**H29**	GlcA-GlcNS6S-GlcA-GlcNS3S6S-IdoA2S-GlcNS6S
**T21**	GlcA-GalNAc4S-GlcA-GalNAc4S	**T52**	IdoA2S-GlcNS6S-IdoA2S-GlcNS6S	**H30**	GlcA-GlcNS6S-IdoA-GlcNS3S6S-IdoA2S-GlcNS6S
**T22**	GlcA-GlcNS6S-GlcA-GlcNAc	**T53**	GlcA-GlcNS3S6S-IdoA2S-GlcNS6S	**1**	GlcA-GlcNS6S-IdoA2S-GlcNS6S-IdoA2S-GlcNS6S
**T23**	IdoA-GlcNS-IdoA-GlcNS	**H1**	GlcA-GlcNAc-IdoA2S-GlcNAc6S-GlcA-GlcNAc	**H31**	GlcA-GlcNS6S-IdoA2S-GlcNS3S-IdoA2S-GlcNS6S
**T24**	GlcA-GlcNAc6S-IdoA2S-GlcNAc6S	**H2**	GlcA-GlcNS-IdoA2S-GlcNS6S-GlcA-GlcNS	**H32**	GlcA-GlcNS6S-IdoA2S-GlcNS3S6S-IdoA2S-GlcNS6S
**T25**	IdoA-GlcNAc6S-IdoA2S-GlcNAc6S	**H3**	GlcA-GlcNAc-IdoA2S-GlcNS6S-IdoA2S-GlcNS	**H33**	IdoA2S-GlcNS6S-IdoA2S-GlcNS6S-IdoA2S-GlcNS6S
**T26**	GlcA-GlcNS-IdoA2S-GlcNS	**H4**	GlcA-GlcNS6S-GlcA-GlcNS6S-GlcA-GlcNS6S	**O1**	GlcA-GlcNS6S-IdoA-GlcNS-IdoA2S-GlcNS6S-IdoA-GlcNAc6S
**T27**	GlcA-GlcNS-GlcA2S-GlcNS	**H5**	GlcA-GlcNS6S-IdoA-GlcNS6S-GlcA-GlcNS6S	**O2**	IdoA2S-GlcNS6S-[IdoA2S-GlcNS6S]$_2$-IdoA2S-GlcNS6S

Figure S8. Binding analysis of synthetic HS oligosaccharides to SARS-CoV-2 related proteins by microarray. (A) SARS-CoV-1 RBD (20 μg/mL), (B) SARS-CoV-2 Wuhan RBD (20 μg/mL), (C) SARS-CoV-2 Alpha RBD (20 μg/mL), (D). SARS-CoV-2 Beta RBD (20 μg/mL), (E). SARS-CoV-2 Gamma RBD (20 μg/mL), (F). SARS-CoV-2 Delta RBD (20 μg/mL), (G) SARS-CoV-2 Omicron RBD (20 μg/mL), (H) Compounds numbering and structures (D: disaccharide, T: tetrasaccharide, H: hexasaccharide, O: octasaccharide).

6. Coagulation-related proteins SPR binding and competition studies

Coagulation-related proteins SPR binding studies: Human antithrombin-III (AT-III) was obtained from Haematologic technologies. Recombinant human platelet factor-4 (PF-4, also known as CXCL4) was obtained from Sino Biological, Inc. For kinetic studies, various concentration of AT-III or PF-4 (ranging from 1000 nM to 3.9 nM, 2-fold dilutions) were dissolved in running buffer and flowed over heparin chip (association time: 180 sec and dissociation time: 600 sec) at a flow rate of 30 μL/min and a constant temperature of 25 °C. A 60 sec injection of 2.0 M NaCl at a flow rate of 30 μL/min was used for regeneration and to achieve prior baseline status. To further stabilize the baseline, washing was continued for another 240 sec with running buffer at a flow rate of 30 μL/min. Using Biacore T100 evaluation software, AT-III equilibrium dissociation constant (K_D) was determined by fitting binding curves to two-state reaction model built in Biacore T100 evaluation software. For PF-4, steady-state affinity analysis was performed on the response curves of various PF-4 concentrations and K_D value was calculated (Figure S9).

Coagulation-related proteins SPR competition assays: AT-III (100 nM) or PF-4 (100 nM) alone or in the presence of structures (**1**, **6** and UFH, at a concentration of 100 to 0.001 μg/mL, 10-fold dilutions) was flowed over heparin chip. The samples were diluted in running buffer and a flow rate of 30 μL/min was employed for association (120 sec for AT-III and 180 sec for PF-4) and dissociation (400 sec for AT-III and 600 sec for PF-4) at a constant temperature of 25 °C. A 30 sec injection of 2.0 M NaCl at a flow rate of 30 μL/min was used for regeneration and achieved prior baseline status. The IC_{50} values (Figures S10-S15) were calculated using dose−response equations [nonlinear regression, log(inhibitor) vs response-variable slope (four parameters)] built in Prism software 9 (GraphPad Software, Inc.). All experiments were performed (in duplicate) three times at the minimum.

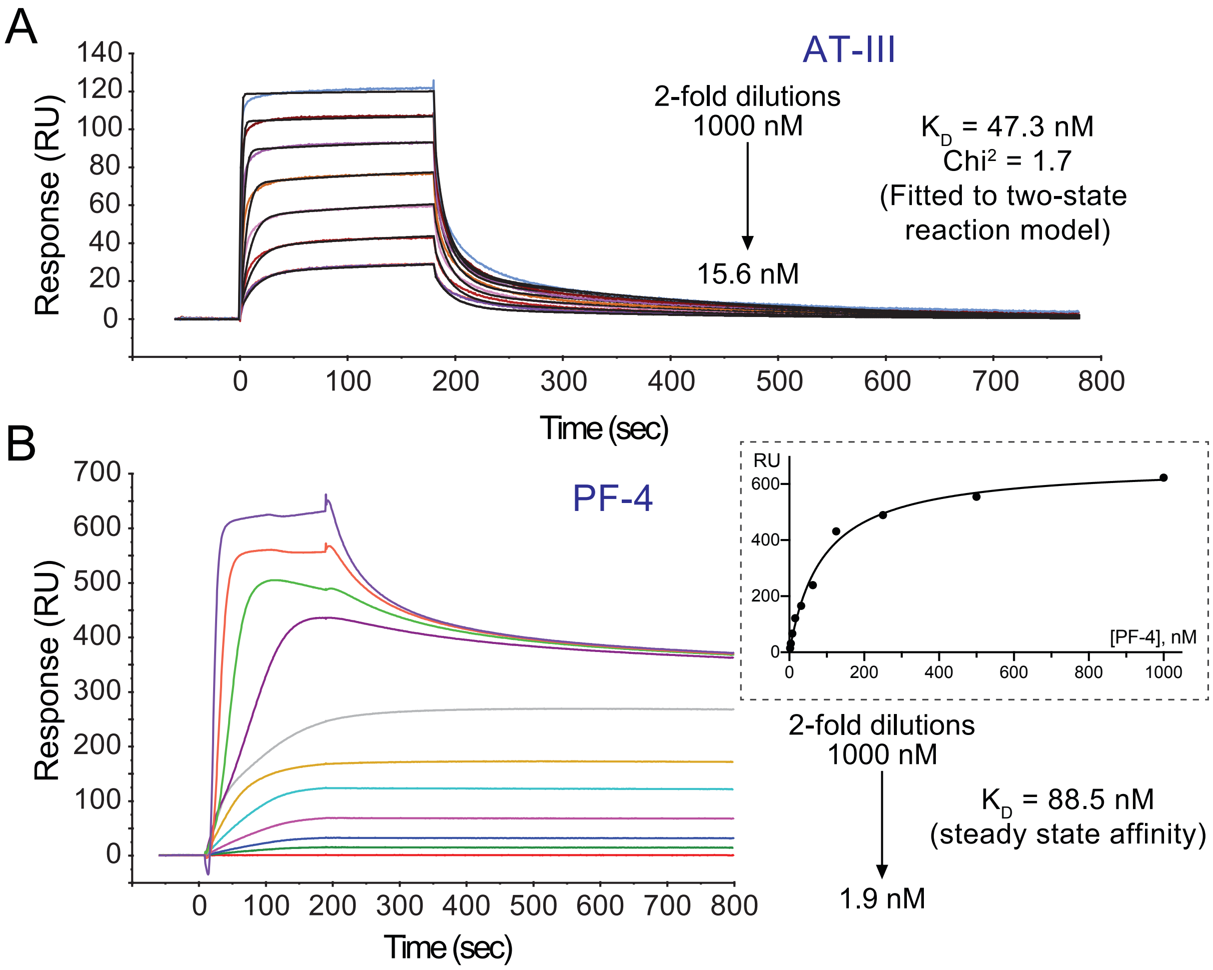

Figure S9. Surface Plasmon Resonance (SPR) binding analysis of Antithrombin-III (AT-III) and Platelet factor-4 (PF-4) to heparin chip. (A) Sensorgram of AT-III showing concentration-dependent binding to immobilized heparin, binding curves were fitted to two-state reaction model; (B) Sensorgram of PF-4 binding to heparin, steady state affinity analysis was performed to calculate equilibrium dissociation constant (K_D). Each experiment was performed in duplicate (for each concentration of AT-III/PF-4) at least three times, representative sensorgrams are presented. Data were analyzed using Biacore T100 evaluation software.

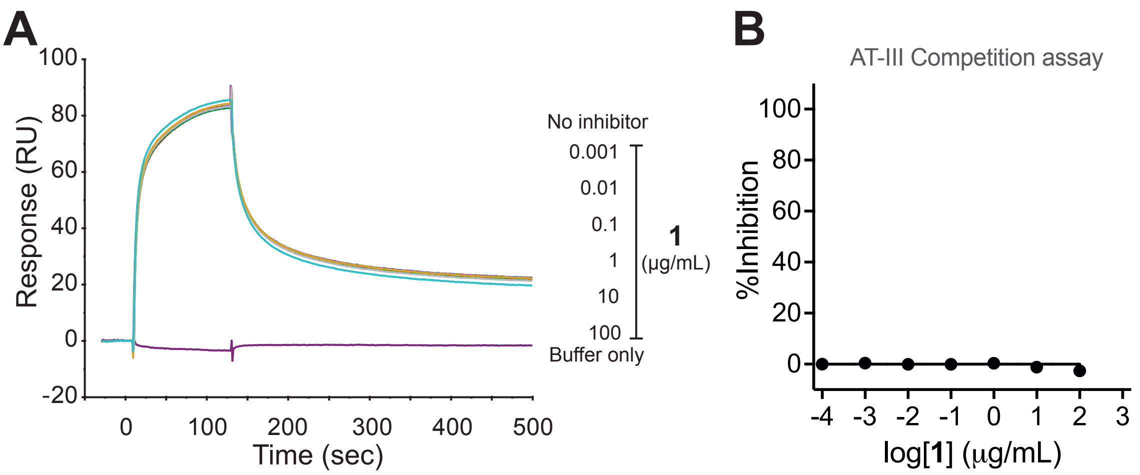

Figure S10. AT-III competition SPR data of compound **1.** (A) Sensorgram of AT-III in the presence of **1** at various concentrations (ranging from 100 to 0.001 μg/mL, 10-fold dilutions); (B) Inhibition curve of compound **1** for binding of AT-III to heparin-immobilized surface.

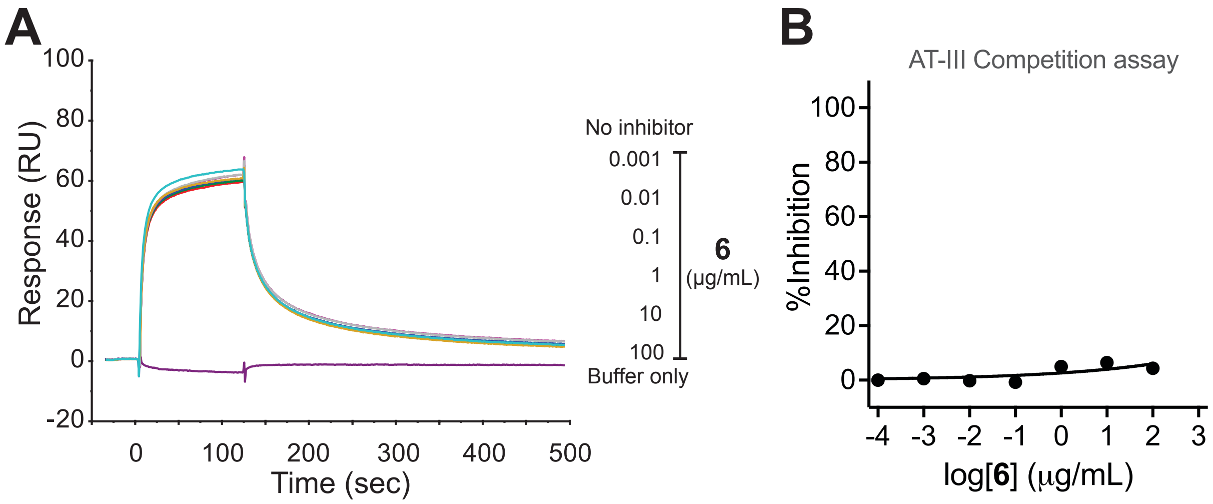

Figure S11. AT-III competition SPR data of compound **6**. (A) Sensorgram of AT-III in the presence of **6** at various concentrations (ranging from 100 to 0.001 μg/mL, 10-fold dilutions); (B) Inhibition curve of compound **6** for binding of AT-III to heparin-immobilized surface.

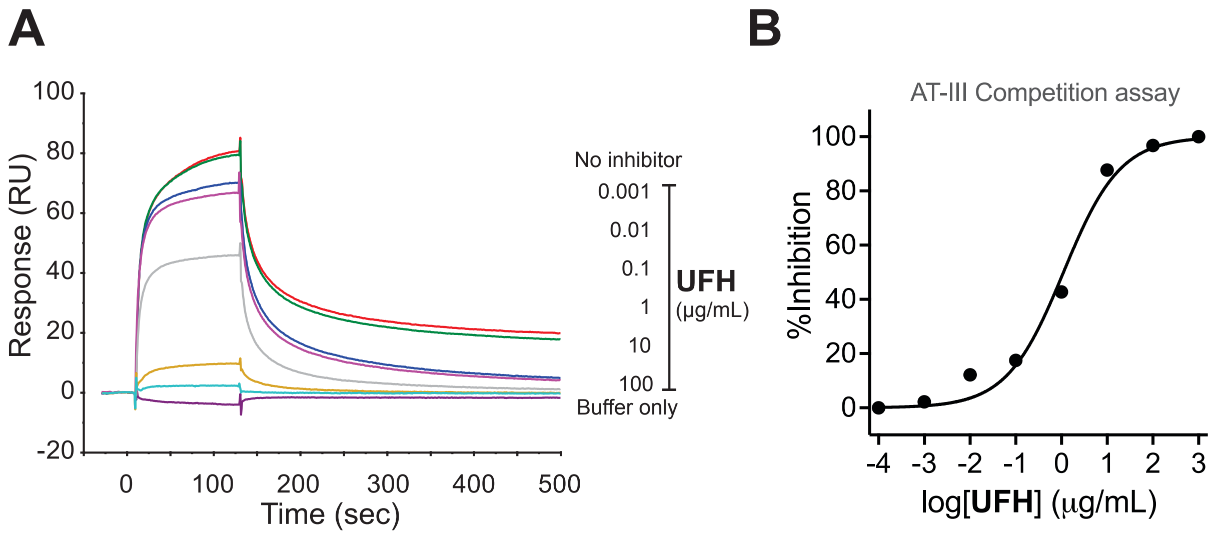

Figure S12. AT-III competition SPR data of unfractionated heparin (**UFH**). (A) Sensorgram of AT-III in the presence of **UFH** at various concentrations (ranging from 100 to 0.001 μg/mL, 10-fold dilutions); (B) Inhibition curve of **UFH** for binding of AT-III to heparin-immobilized surface.

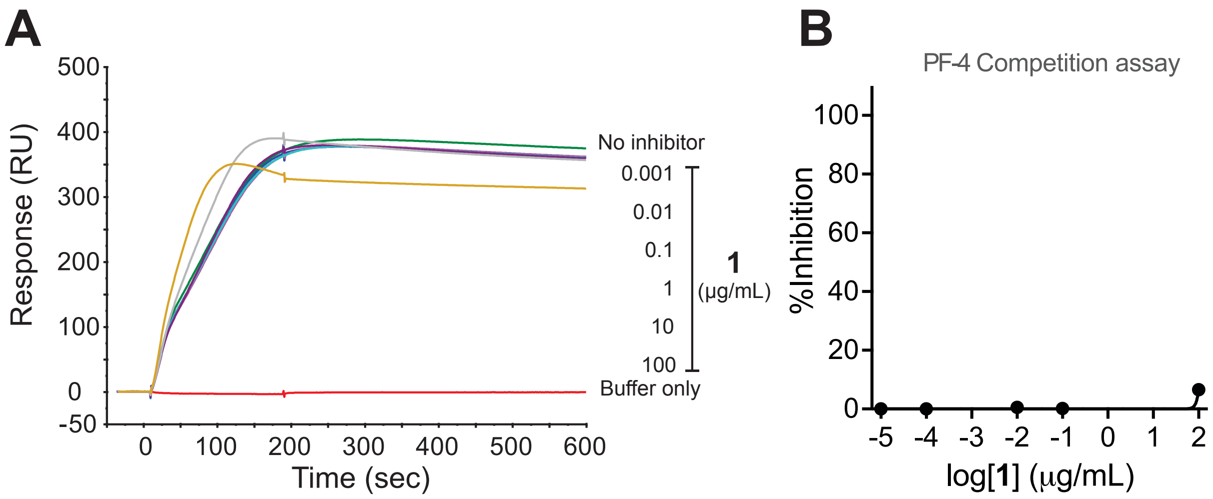

Figure S13. PF-4 competition SPR data of compound **1**. (A) Sensorgram of PF-4 in the presence of **1** at various concentrations (ranging from 100 to 0.001 μg/mL, 10-fold dilutions); (B) Inhibition curve of compound **1** for binding of PF-4 to heparin-immobilized surface.

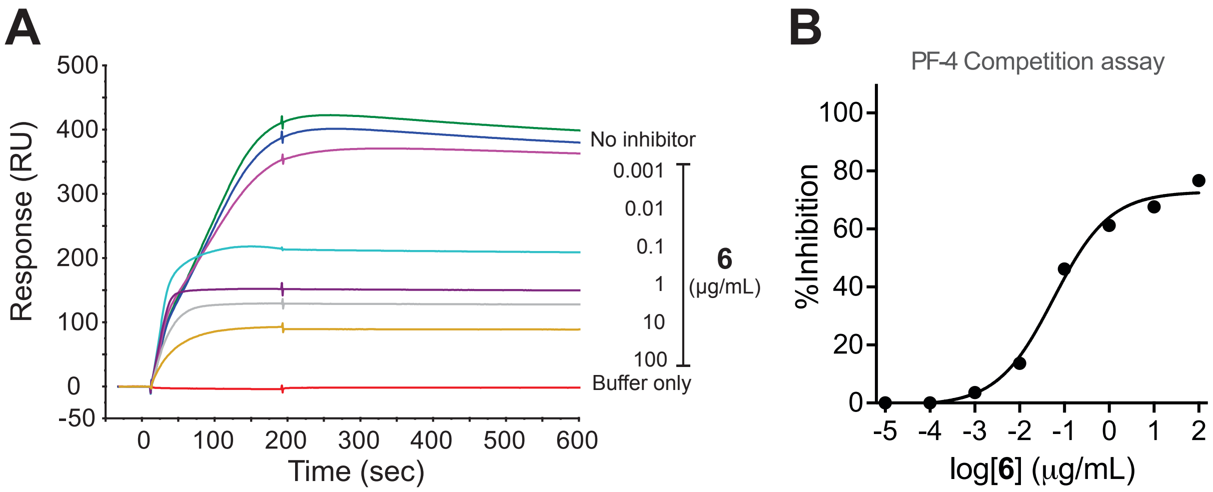

Figure S14. PF-4 competition SPR data of compound **6**. (A) Sensorgram of PF-4 in the presence of **6** at various concentrations (ranging from 100 to 0.001 μg/mL, 10-fold dilutions); (B) Inhibition curve of compound **6** for binding of PF-4 to heparin-immobilized surface.

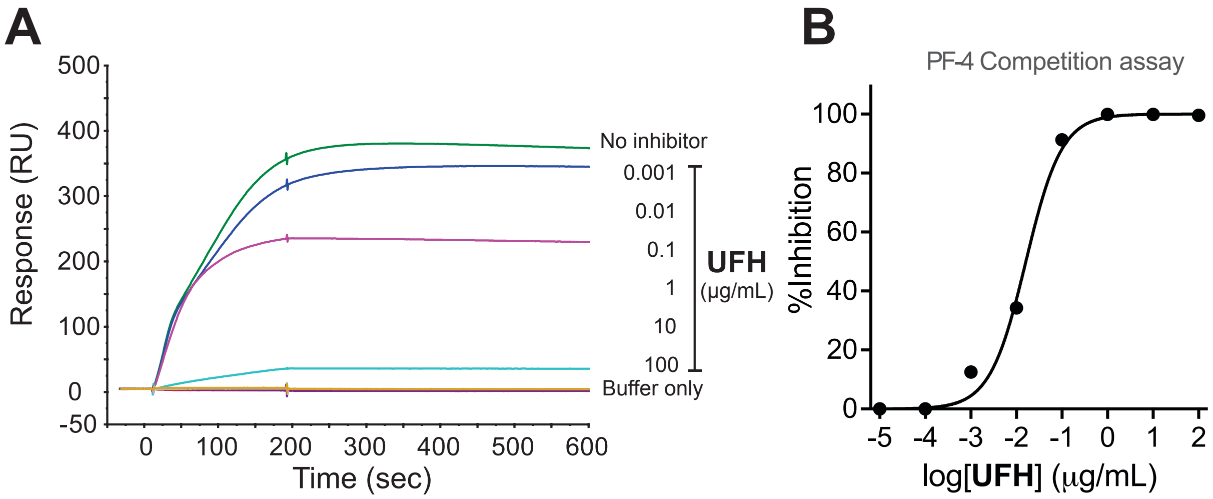

Figure S15. PF-4 competition SPR data of unfractionated heparin (**UFH**). (A) Sensorgram of PF-4 in the presence of **UFH** at various concentrations (ranging from 100 to 0.001 μg/mL, 10-fold dilutions); (B) Inhibition curve of **UFH** for binding of PF-4 to heparin-immobilized surface.

7. Anticoagulant assays

AT-III mediated Factor-IIa inhibition assays: Anti-Factor IIa activity of mimetics (**1** and **6**) and UFH was determined by BIOPHEN™ ANTI-IIa (2 Stages Heparin Assay) kit following manufacturer's instructions. Human antithrombin (AT-III), purified human thrombin (Factor IIa) and thrombin specific chromogenic substrate [CS-01(38)] were reconstituted and prepared in specific buffers in accordance with kit protocol. The solution of AT-III (0.01 IU, 40 μL) and sample or control (different concentrations, 40 μL) in triplicate in a 96-well plate (flat-bottom, clear) was incubated at 37 °C for 2 min. Factor IIa (0.96 NIH units, 40 μL) was then introduced, mixed and incubated at 37 °C for 2 min, followed by addition of chromogenic substrate (0.05 μmol, 40 μL). After exactly 2 min, the reaction was stopped by introducing citric acid (2 wt.%, 80 μL), mixed and absorbance at 405 nm (POLARstar Optima, BMG Labtech) against the corresponding blank was measured. The sample blank is obtained by mixing the test components in a reverse order: citric acid, Factor IIa substrate, Factor IIa, AT-III, and the sample. Measured blank value were subtracted from the absorbance measured for the corresponding test. The IC_{50} values (Figures S16) were calculated using dose-response equations [nonlinear regression, log(inhibitor) vs. response-variable slope (four parameters)] built in Prism software 9 (GraphPad Software, Inc.). All experiments were performed (in triplicate) two times at the minimum.

AT-III mediated Factor-Xa inhibition assays: Anti-Factor Xa activity of mimetics (**1** and **6**) and UFH was determined by BIOPHEN™ ANTI-Xa (2 Stages Heparin Assay) kit following manufacturer's instructions. Human antithrombin (AT-III), purified bovine Factor Xa and Factor Xa specific chromogenic substrate [CS-11(65)] were reconstituted and prepared in specific buffers in accordance with kit protocol. The solution of AT-III (0.04 IU, 40 μL) and sample or control (different concentrations, 40 μl) in triplicate in a 96-well plate (flat-bottom, clear) was incubated at 37 °C for 2 min. Factor Xa (0.32 mg, 40 μL) was then introduced, mixed and incubated at 37 °C for 2 min, followed by addition of chromogenic substrate (0.032 mg, 40 μL). After exactly 2 mins, the reaction was stopped by introducing citric acid (2 wt.%, 80 μL), mixed and absorbance at 405 nm (POLARstar Optima, BMG Labtech) against the

corresponding blank was measured. The sample blank is obtained by mixing the test components in a reverse order: citric acid, Factor Xa substrate, Factor Xa, AT-III, and the sample. Measured blank value were subtracted from the absorbance measured for the corresponding test. The IC_{50} values (Figures S17) were calculated using dose-response equations [nonlinear regression, log(inhibitor) vs. response-variable slope (four parameters)] built in Prism software 9 (GraphPad Software, Inc.). All experiments were performed (in triplicate) two times at the minimum.

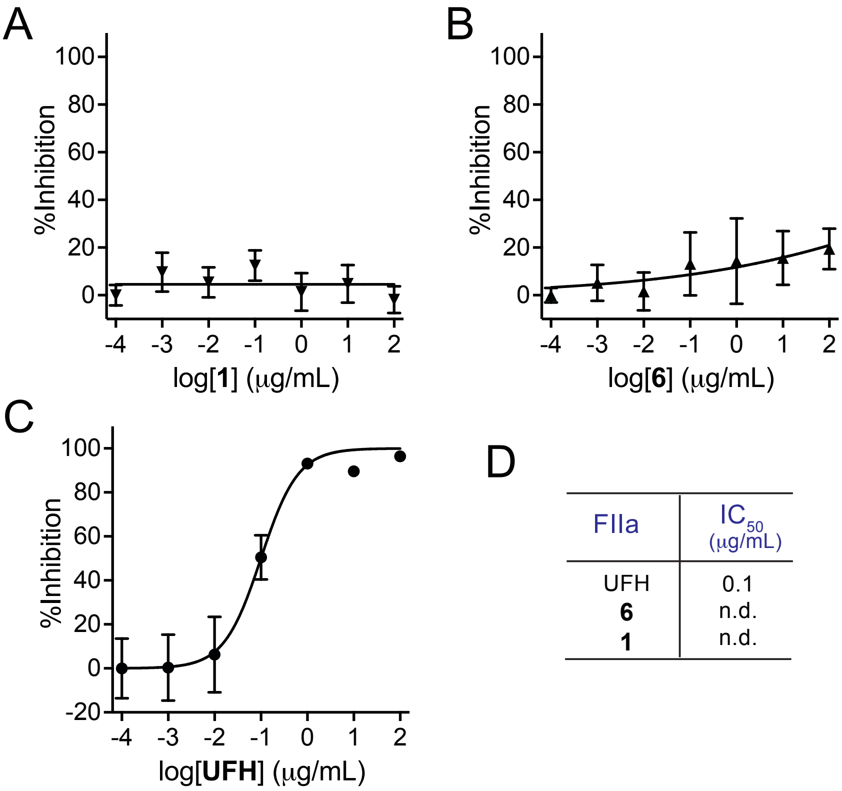

FIIa	IC_{50} (μg/mL)
UFH	0.1
**6**	n.d.
**1**	n.d.

Figure S16. Anticoagulant activity of heparin mimetics was determined by utilizing AT-III mediated Factor-IIa inhibition assays. (A) Inhibition curve of compound **1**; (B) Inhibition curve of compound **6**; (C) Inhibition curve of unfractionated heparin **UFH**; (D) Table of half-maximal inhibitory concentration (IC50) values of each compound tested. Data is presented as mean ± SD (n = 3). Representative data is shown, each experiment was repeated at least two times.

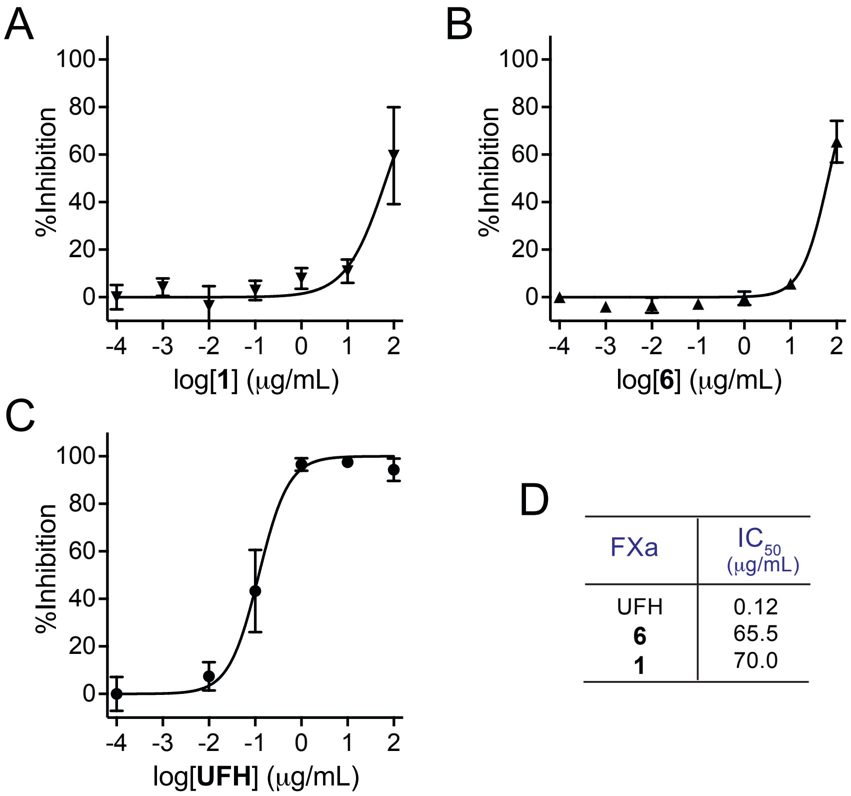

D

FXa	IC_{50} (μg/mL)
UFH	0.12
**6**	65.5
**1**	70.0

Figure S17. Anticoagulant activity of heparin mimetics was determined by utilizing AT-III mediated Factor-Xa inhibition assays. (A) Inhibition curve of compound **1**; (B) Inhibition curve of compound **6**; (C) Inhibition curve of unfractionated heparin **UFH**; (D) Table of half-maximal inhibitory concentration (IC50) values of each compound tested. Data is presented as mean ± SD (n = 3). Representative data is shown, each experiment was repeated at least two times.

8. Vero E6 cell inhibition experiments

Recombinantly expressed trimeric RBD plasmid generation

Recombinant SARS-CoV-2 envelope protein RBD (GenBank: MN908947.3; AA 319-541) was cloned into a pCD5 expression vector using Gibson assembly from cDNAs encoding codon-optimized open reading frames of full-length SARS-CoV-2 Wuhan, Alpha, Beta and Gamma spike, as previously described and provided by Rogier W. Sanders.[6] The Delta RBD was generated by mutating amino acids L452R and T478K in SARS-CoV-2 Wuhan RBD. The Omicron RBD was ordered from GenScript with mutations G339D, S371L, S373P, S375F, K417N, N440K, G446S, S477N, E484A, Q493R, G496S, Q498R, N501Y and N505H in the SARS-CoV-2 Wuhan RBD. The pCD5 expression vector was adapted so that after the signal

sequence, the SARS-COV-2 RBD-encoding cDNAs are cloned in frame with a GCN4-pIL trimerization motif (KQIEDKIEEIESKQKKIENEIARIKK), a TEV cleavage site (ENLYFQG), fluorescent reporter open reading frame, and the Strep-tag II (WSHPQFEKGGGSGGGSWSHPQFEK); IBA, Germany). Transfection was performed using the pCD5 expression vectors, polyethyleneimine I and HEK293T cells.[6] The transfection mixtures were replaced at 6 h post-transfection by 293 SFM II expression medium (Gibco), supplemented with sodium bicarbonate (3.7 g/L), Primatone RL-UF (3.0 g/L), glucose (2.0 g/L), glutaMAX (Gibco), valproic acid (0,4 g/L) and DMSO (1,5%). At 5 to 6 days after transfection, tissue culture supernatants were collected.

Immunofluorescent staining

Vero-E6 cells were grown on coverslips for immunofluorescent staining. Cells were fixed with 4% paraformaldehyde in PBS for 25 min at room temperature, after which permeabilization was performed using 0.1% Triton X-100 in PBS. Untreated SARS-CoV-2 Wuhan and SARS-CoV-2 Delta RBDs or pretreated for 1 h with 25 μg/ml UFH, 10-125 μg/mL compound **1** or **6** were applied to the coverslips at 50 μg/mL for 1 h at room temperature. Primary StrepMab-Classic HRP classic and secondary AlexaFluor555 goat anti-mouse antibodies (Invitrogen) were applied sequentially with PBS washes in between. DAPI (Invitrogen) was used as a nuclear stain. Samples were imaged on a Leica DMi8 confocal microscope equipped with a ×10 HC PL Apo CS2 objective (0.40 NA). Excitation was achieved with a diode 405 or white light for excitation of Alexa555, a pulsed white laser (80 MHz) was used at 549 nm, and emissions were obtained in the range of 594–627 nm. Laser powers were 10–20% with a maximum gain of 200. LAS Application Suite X was used as well as ImageJ for the addition of the scale bars.

Cytotoxicity assay

Cytotoxicity assay on Vero E6 cells was performed using CellTox™ Green Cytotoxicity Assay (G8741, Promega). A black polystyrene plate (204626-100, Agilent) was used with 30000 Vero

E6 cells seeded into each well with a final volume of 50 µL. Compounds of interest were diluted in DMEM (UFH: 25 µg/mL, compound **1**: 100 µg/mL, compound **6**: 100 µg/mL) to a final volume of 50 µL and then added to the seeded Vero E6 cells. After 24 h incubation, lysis solution (1:25) was added to the positive control wells and incubated for 30 min prior to the reading. CellTox™ Green Reagent (2X) was prepared according to the manufacturer's instructions, after which 100 µL of CellTox™ Green Reagent (2X) was added to all wells and incubated for 15 minutes whilst protected from light. Fluorescence signal was measured using a POLARstar Omega platereader using excitation at 485-10 nm and emission at 520 nm.

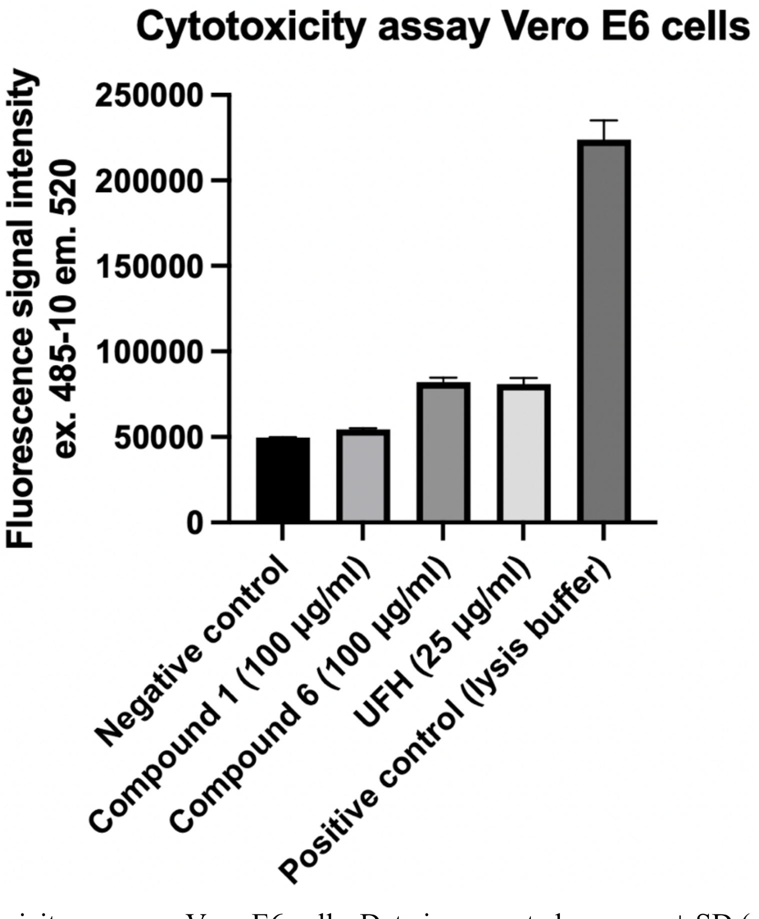

Figure S18. Cytotoxicity assay on Vero E6 cells. Data is presented as mean ± SD (n = 3).

9. References

(1) Liu, R.; Xu, Y.; Chen, M.; Weiwer, M.; Zhou, X.; Bridges, A. S.; DeAngelis, P. L.; Zhang, Q.; Linhardt, R. J.; Liu, J. Chemoenzymatic design of heparan sulfate oligosaccharides. *J. Biol. Chem.* **2010**, *285*, 34240-34249.

(2) Arungundram, S.; Al-Mafraji, K.; Asong, J.; Leach, F. E., III; Amster, I. J.; Venot, A.; Turnbull, J. E.; Boons, G. J. Modular synthesis of heparan sulfate oligosaccharides for structure-activity relationship studies. *J. Am. Chem. Soc.* **2009**, *131*, 17394-17405.

(3) Zong, C.; Venot, A.; Li, X.; Lu, W.; Xiao, W.; Wilkes, J. L.; Salanga, C. L.; Handel, T. M.; Wang, L.; Wolfert, M. A.; Boons, G. J. Heparan sulfate microarray reveals that heparan sulfate-protein binding exhibits different ligand requirements. *J. Am. Chem. Soc.* **2017**, *139*, 9534-9543.

(4) Stadlbauer, D.; Amanat, F.; Chromikova, V.; Jiang, K.; Strohmeier, S.; Arunkumar, G. A.; Tan, J.; Bhavsar, D.; Capuano, C.; Kirkpatrick, E.; Meade, P.; Brito, R. N.; Teo, C.; McMahon, M.; Simon, V.; Krammer, F. SARS-CoV-2 seroconversion in humans: A detailed protocol for a serological assay, antigen production, and test setup. *Curr. Protoc. Microbiol.* **2020**, *57*, e100.

(5) Amanat, F.; Stadlbauer, D.; Strohmeier, S.; Nguyen, T. H. O.; Chromikova, V.; McMahon, M.; Jiang, K.; Arunkumar, G. A.; Jurczyszak, D.; Polanco, J.; Bermudez-Gonzalez, M.; Kleiner, G.; Aydillo, T.; Miorin, L.; Fierer, D. S.; Lugo, L. A.; Kojic, E. M.; Stoever, J.; Liu, S. T. H.; Cunningham-Rundles, C.; Felgner, P. L.; Moran, T.; Garcia-Sastre, A.; Caplivski, D.; Cheng, A. C.; Kedzierska, K.; Vapalahti, O.; Hepojoki, J. M.; Simon, V.; Krammer, F. A serological assay to detect SARS-CoV-2 seroconversion in humans. *Nat. Med.* **2020**, *26*, 1033-1036.

(6) Bouwman, K. M.; Tomris, I.; Turner, H. L.; van der Woude, R.; Shamorkina, T. M.; Bosman, G. P.; Rockx, B.; Herfst, S.; Snijder, J.; Haagmans, B. L.; Ward, A. B.; Boons, G. J.; de Vries, R. P. Multimerization- and glycosylation-dependent receptor binding of SARS-CoV-2 spike proteins. *PLoS Pathog.* **2021**, *17*, e1009282.

(7) Liu, L.; Chopra, P.; Li, X.; Bouwman, K. M.; Tompkins, S. M.; Wolfert, M. A.; de Vries, R. P.; Boons, G. J. Heparan sulfate proteoglycans as attachment factor for SARS-CoV-2. *ACS Cent. Sci.* **2021**, *7*, 1009-1018.

(8) Zhang, F.; Zheng, L.; Cheng, S.; Peng, Y.; Fu, L.; Zhang, X.; Linhardt, R. J. Comparison of the interactions of different growth factors and glycosaminoglycans. *Molecules* **2019**, *24*, 3360.

(9) Liu, L. Carbohydrate Microarray Processing (v1.0). **2017**, DOI: 10.5281/zenodo.5146251.

10. NMR and ESI-MS spectra

^{1}H NMR spectrum of **S1** (600 MHz, $CDCl_3$)

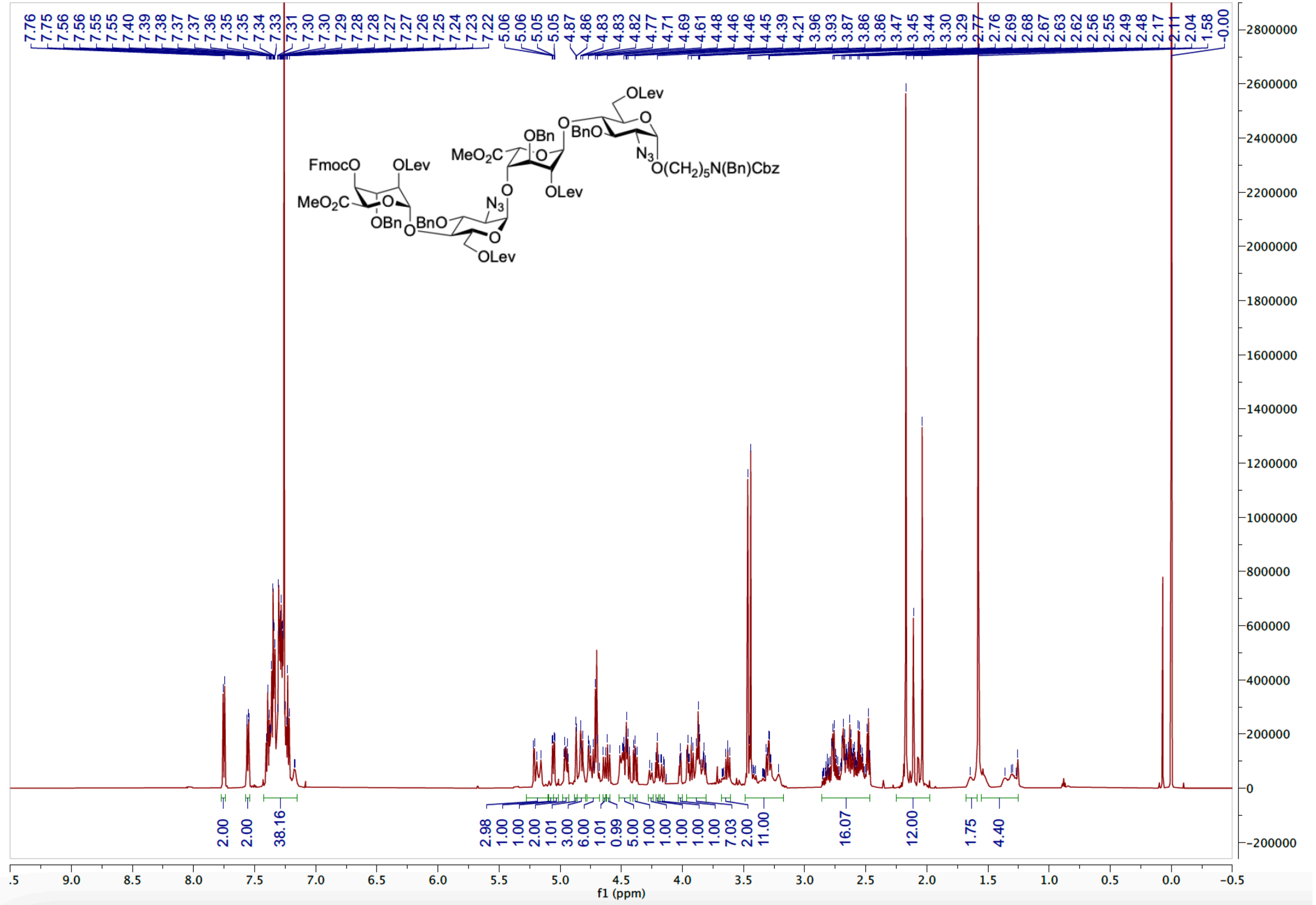

^{13}C NMR spectrum of **S1** (151 MHz, $CDCl_3$)

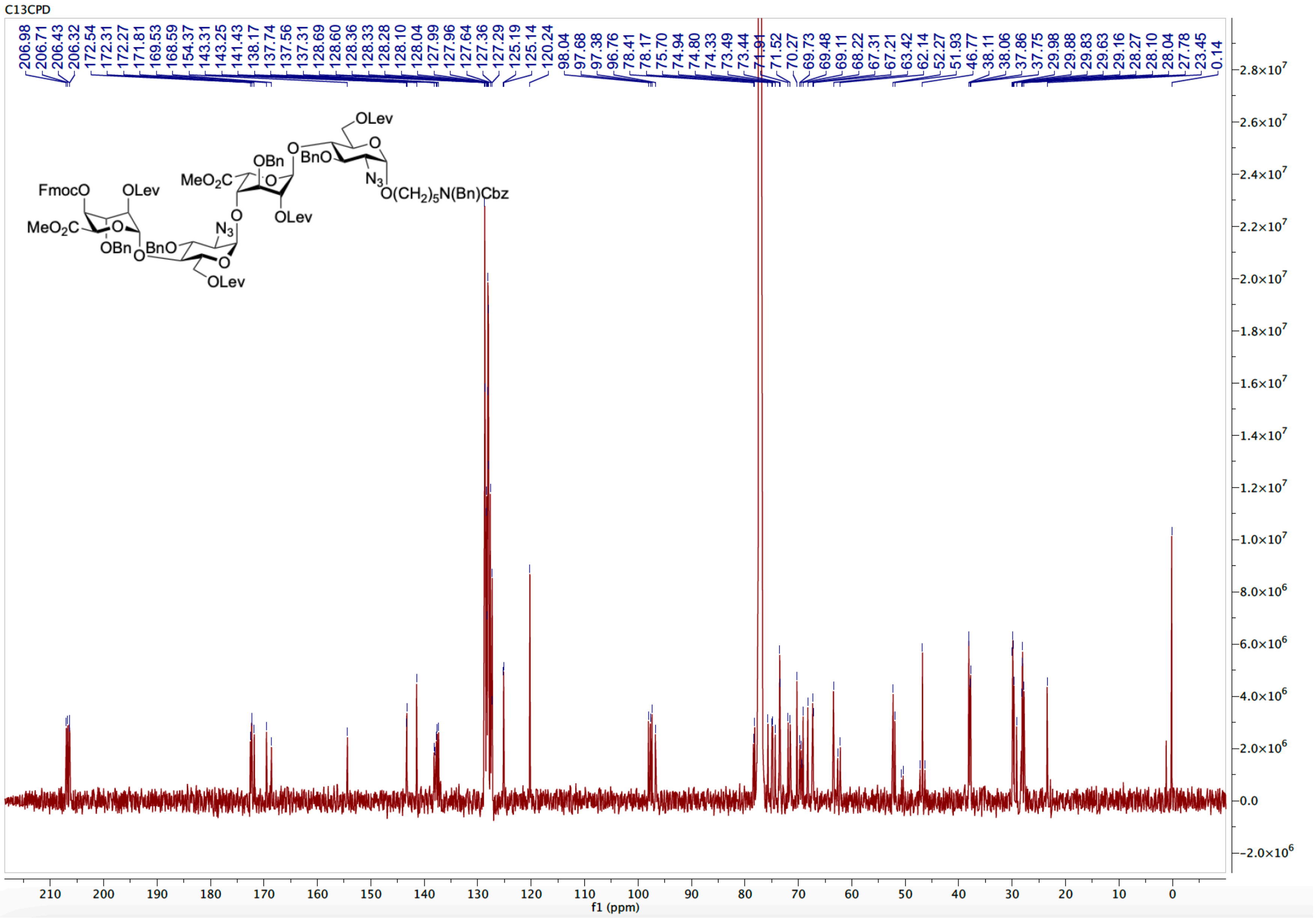

HSQC spectrum of **S1** ($CDCl_3$)

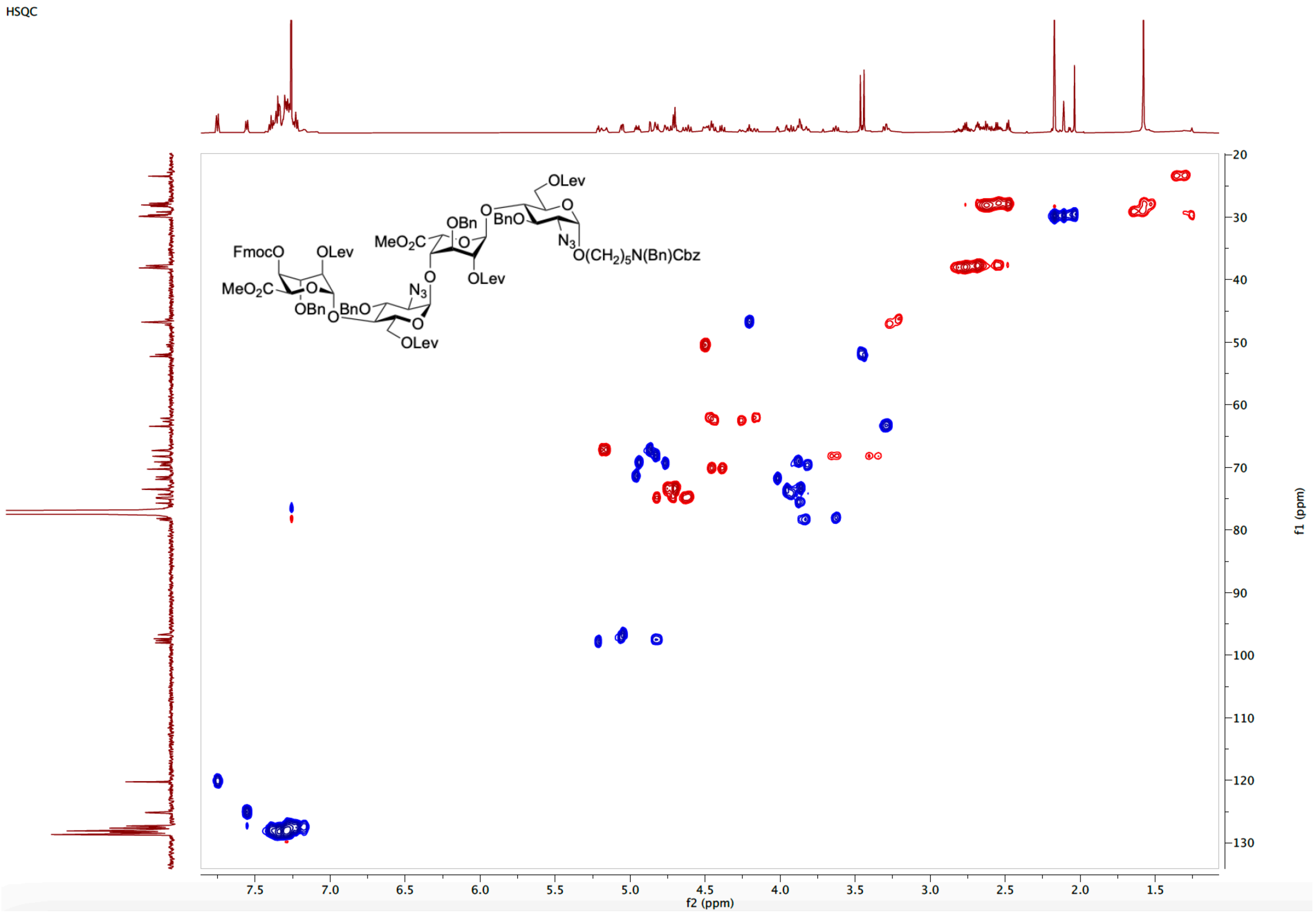

^{1}H NMR spectrum of **S2** (600 MHz, $CDCl_3$)

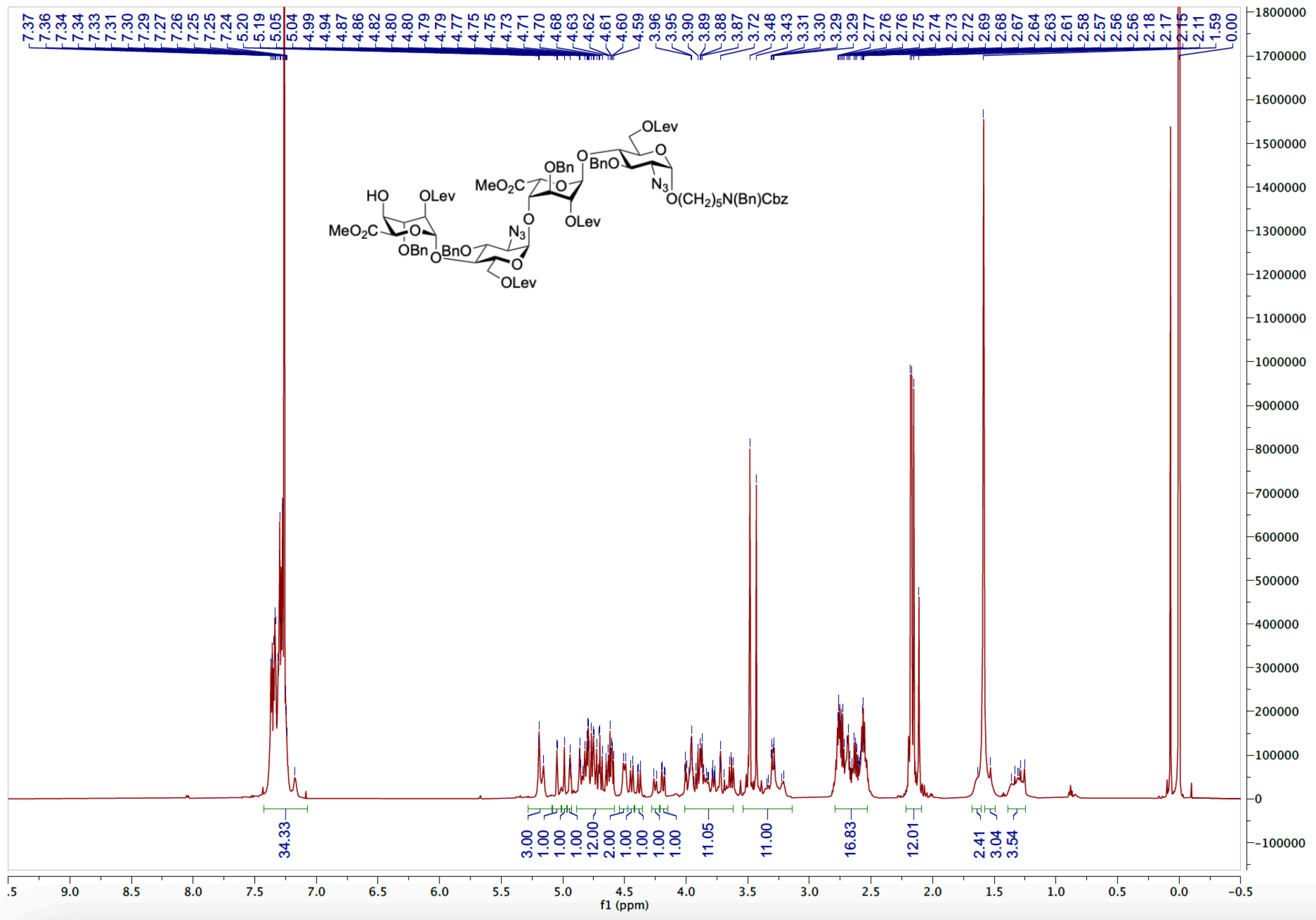

^{13}C NMR spectrum of **S2** (151 MHz, $CDCl_3$)

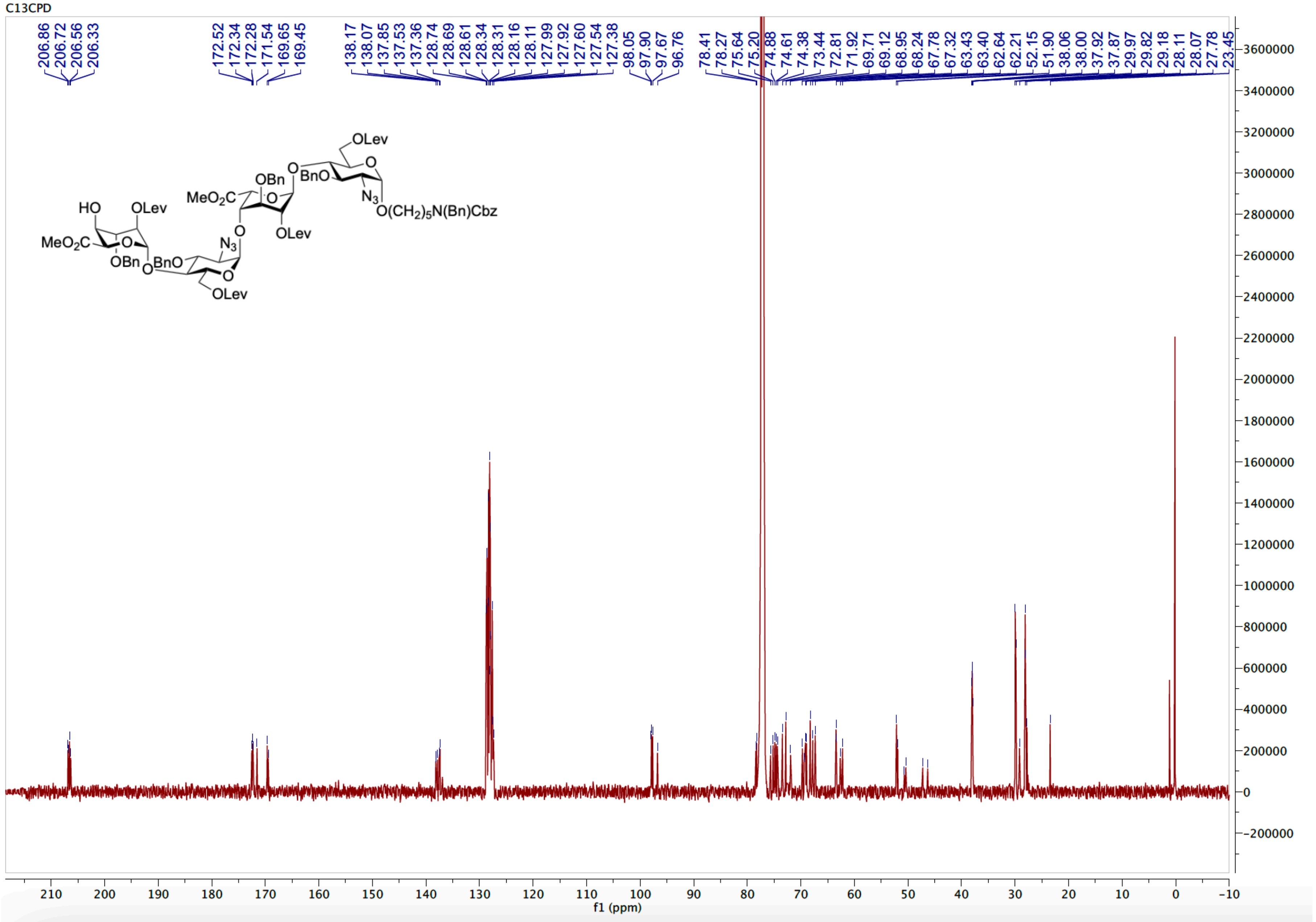

HSQC spectrum of **S2** ($CDCl_3$)

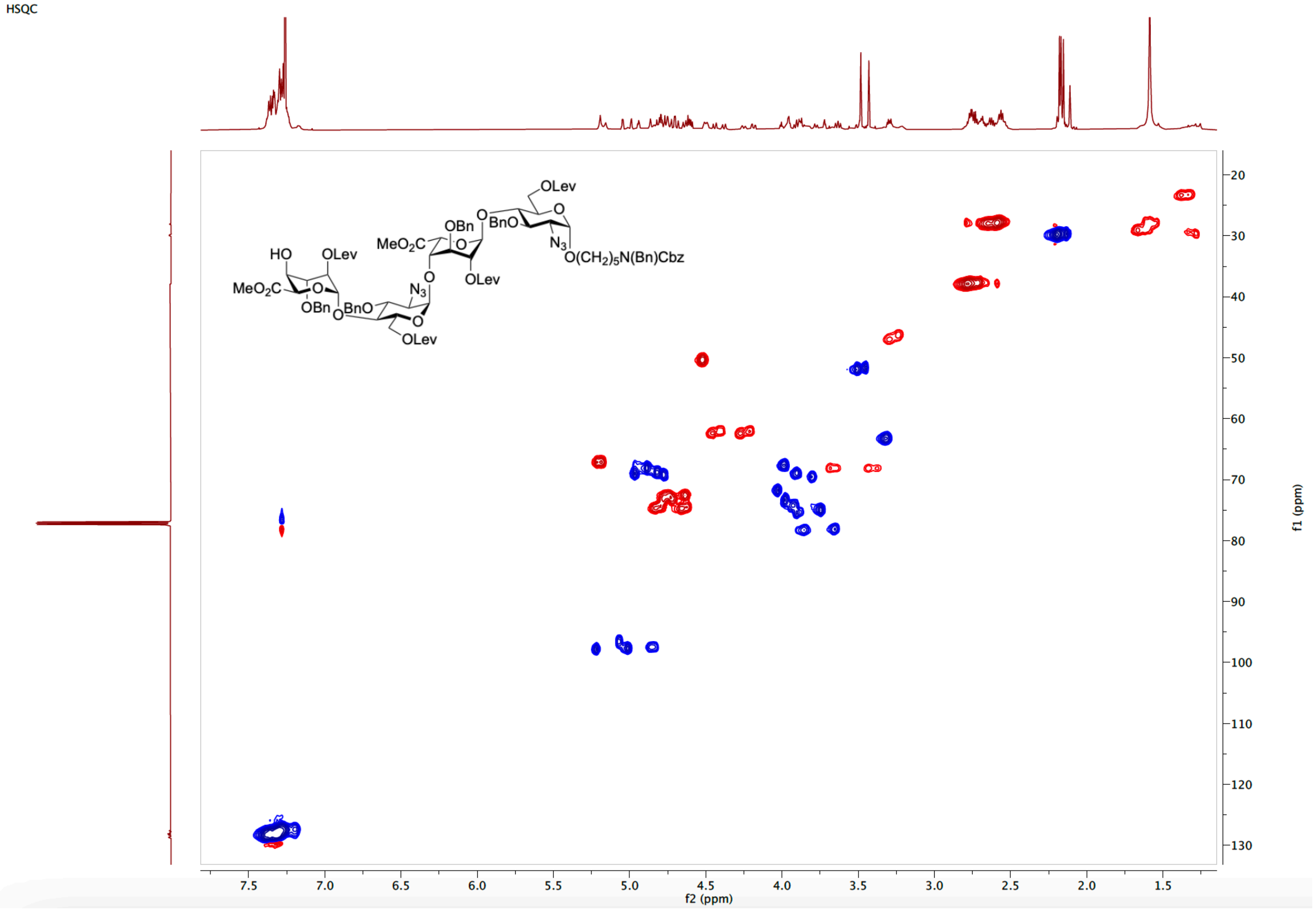

^{1}H NMR spectrum of **10** (600 MHz, $CDCl_3$)

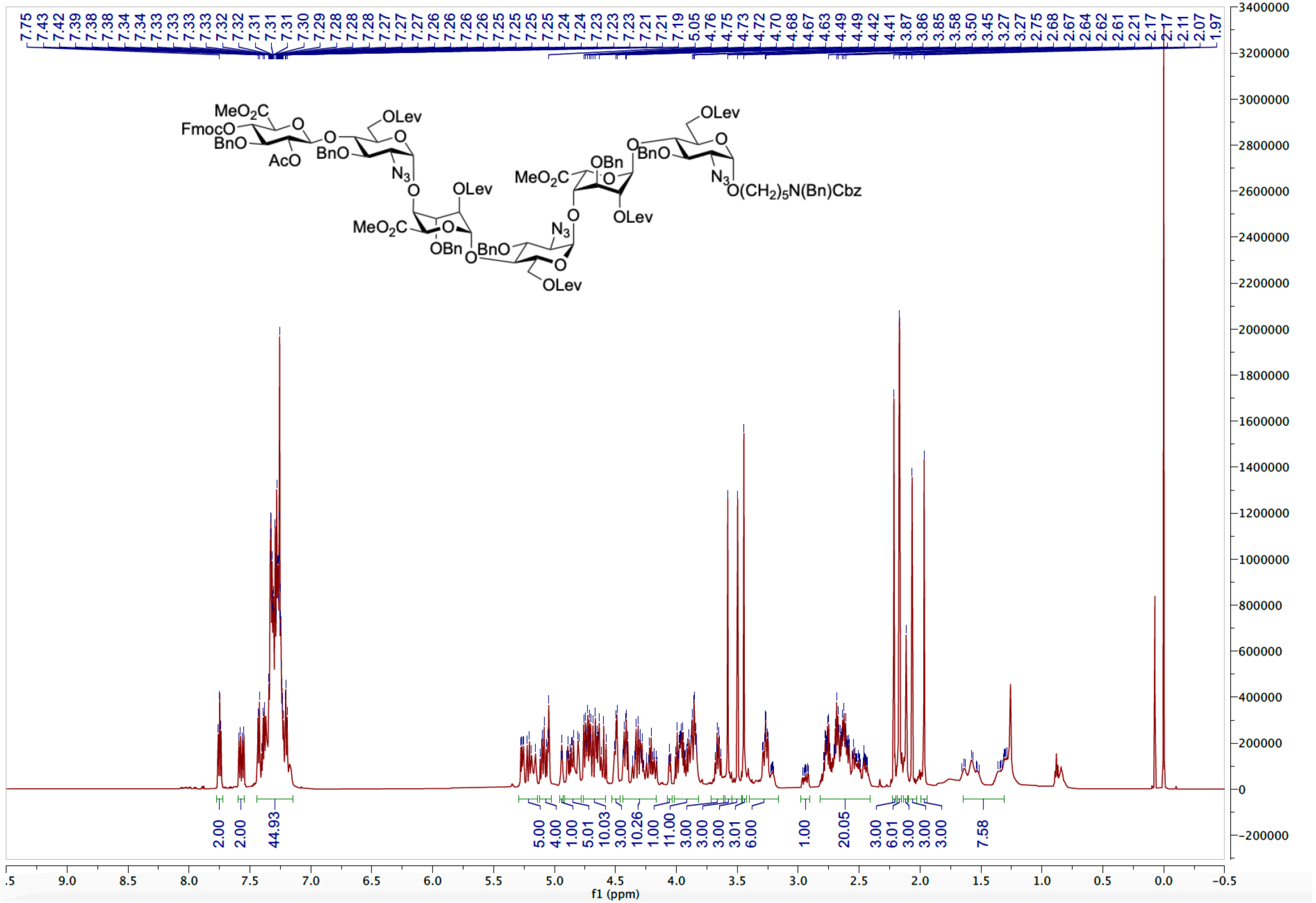

^{13}C NMR spectrum of **10** (151 MHz, $CDCl_3$)

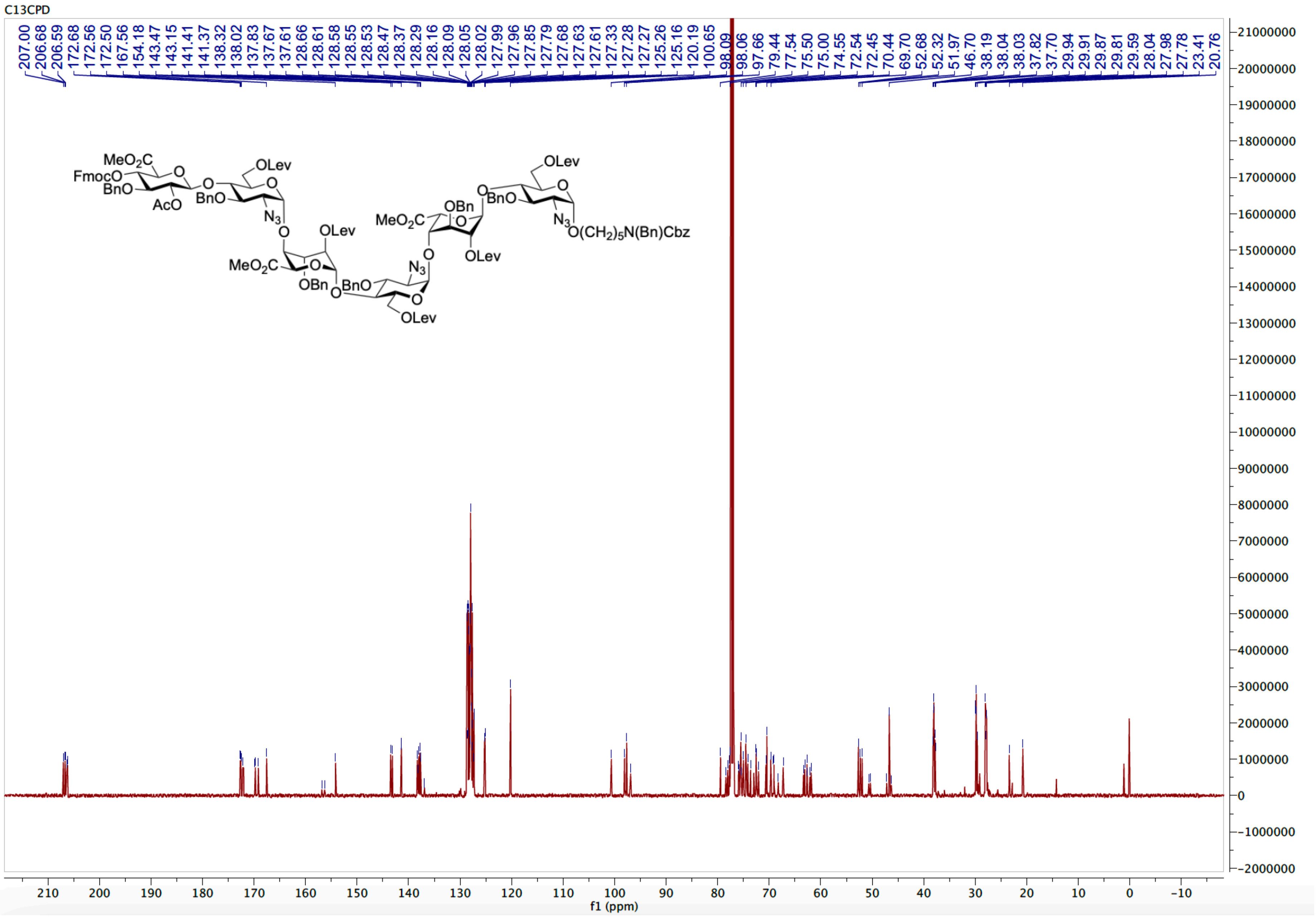

HSQC spectrum of **10** ($CDCl_3$)

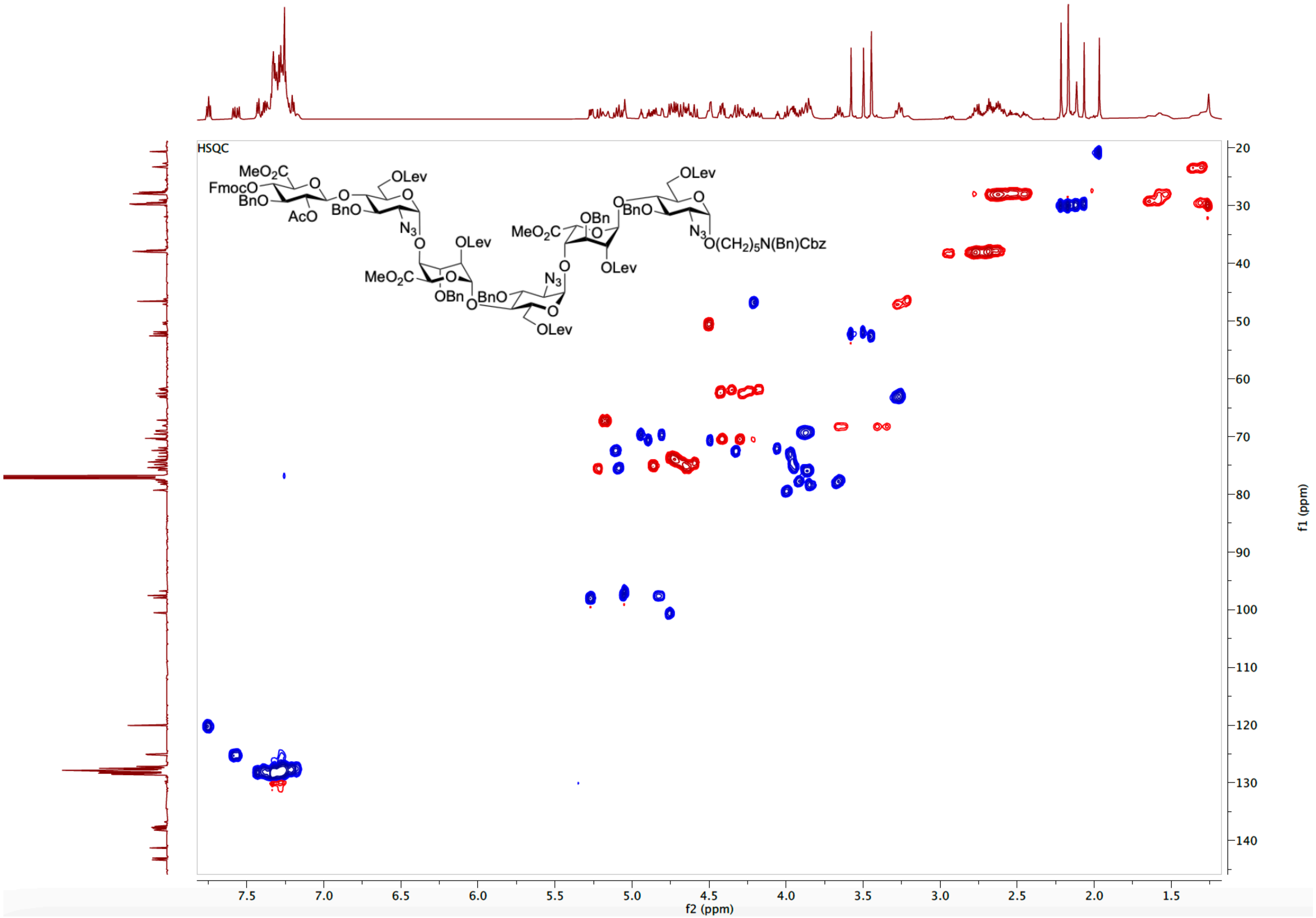

^{1}H NMR spectrum of **11** (600 MHz, D_2O)

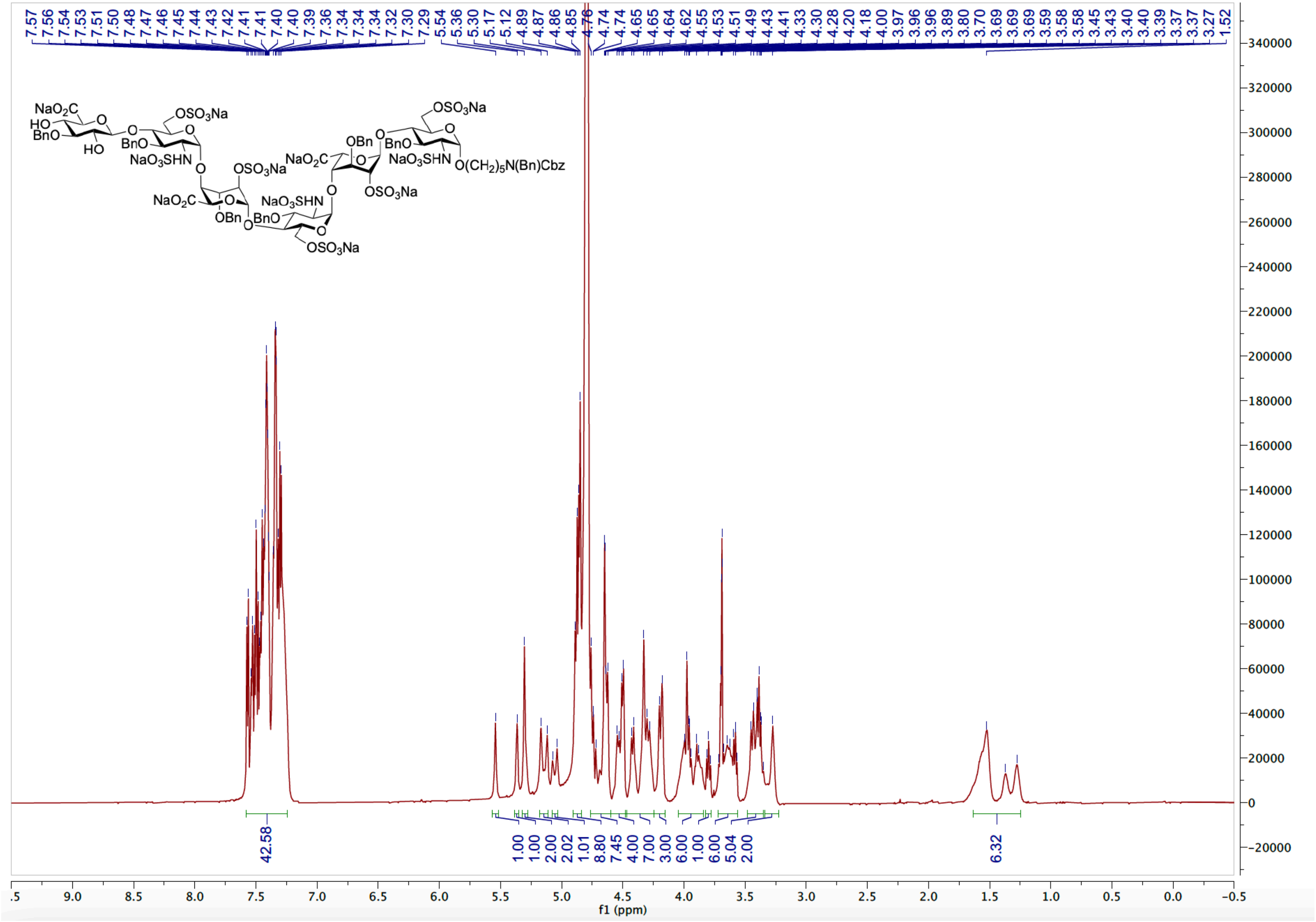

HSQC spectrum of **11** (D_2O)

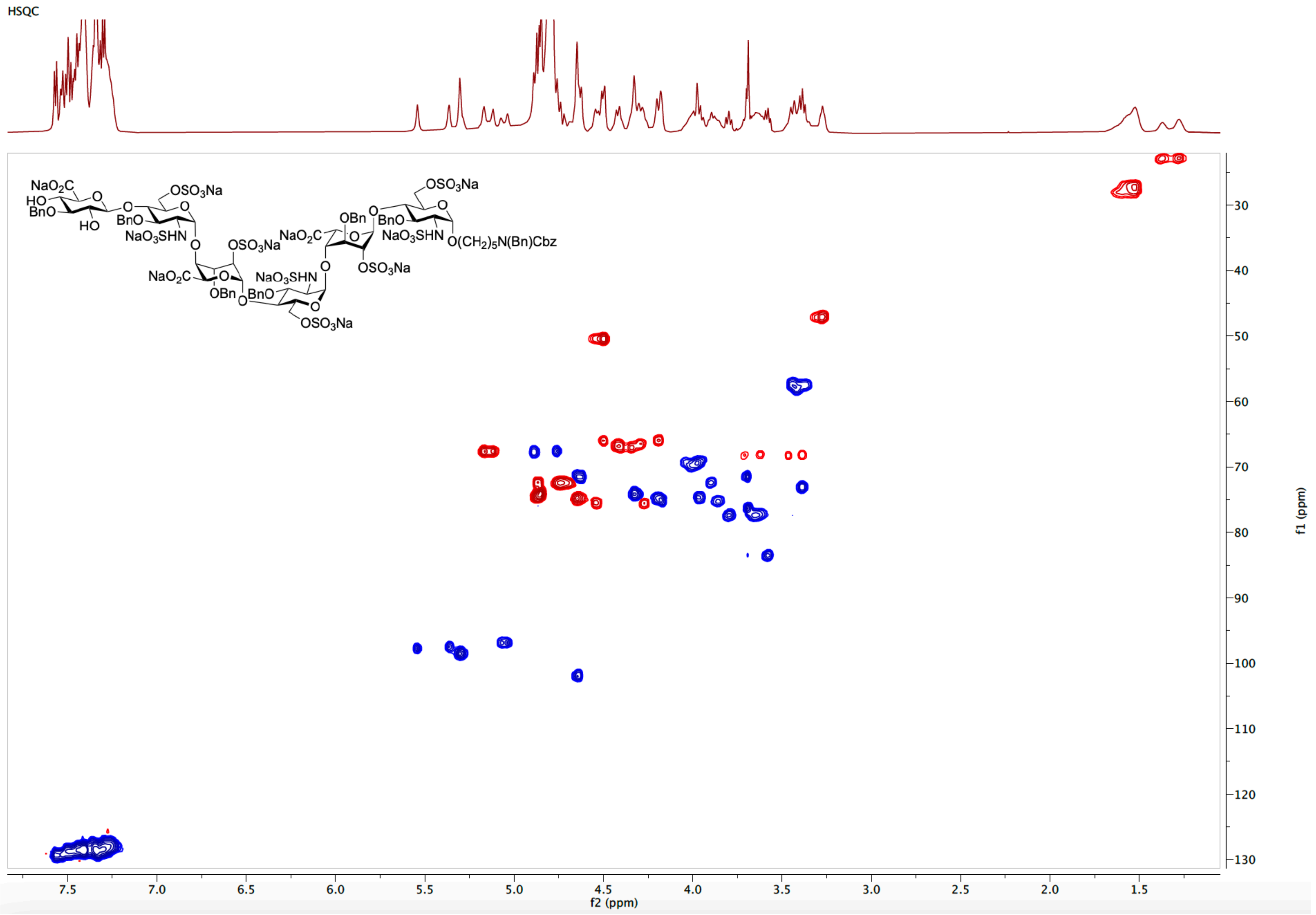

COSY spectrum of **11** (D_2O)

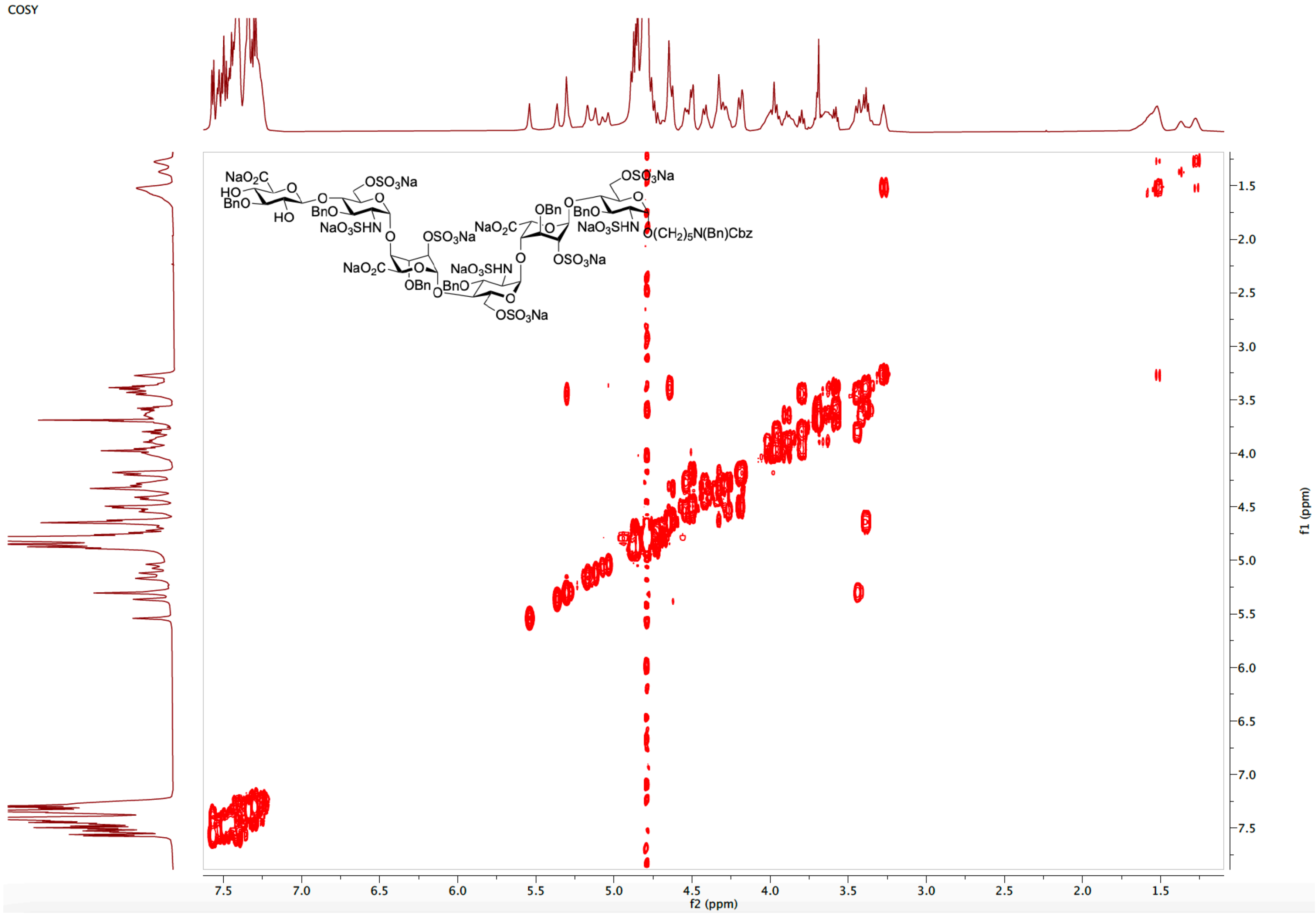

TOCSY spectrum of **11** (D_2O)

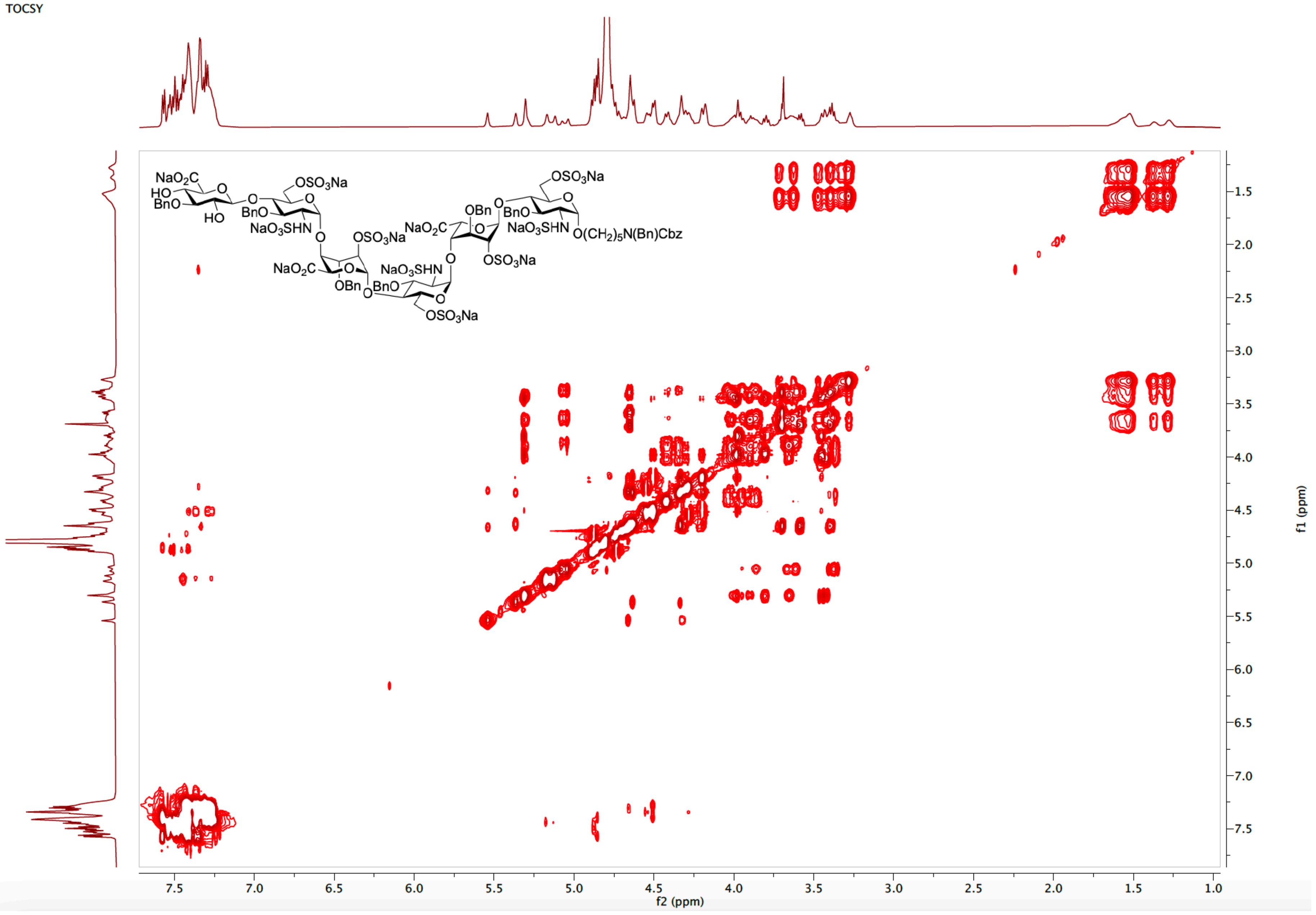

NOESY spectrum of **11** (D_2O)

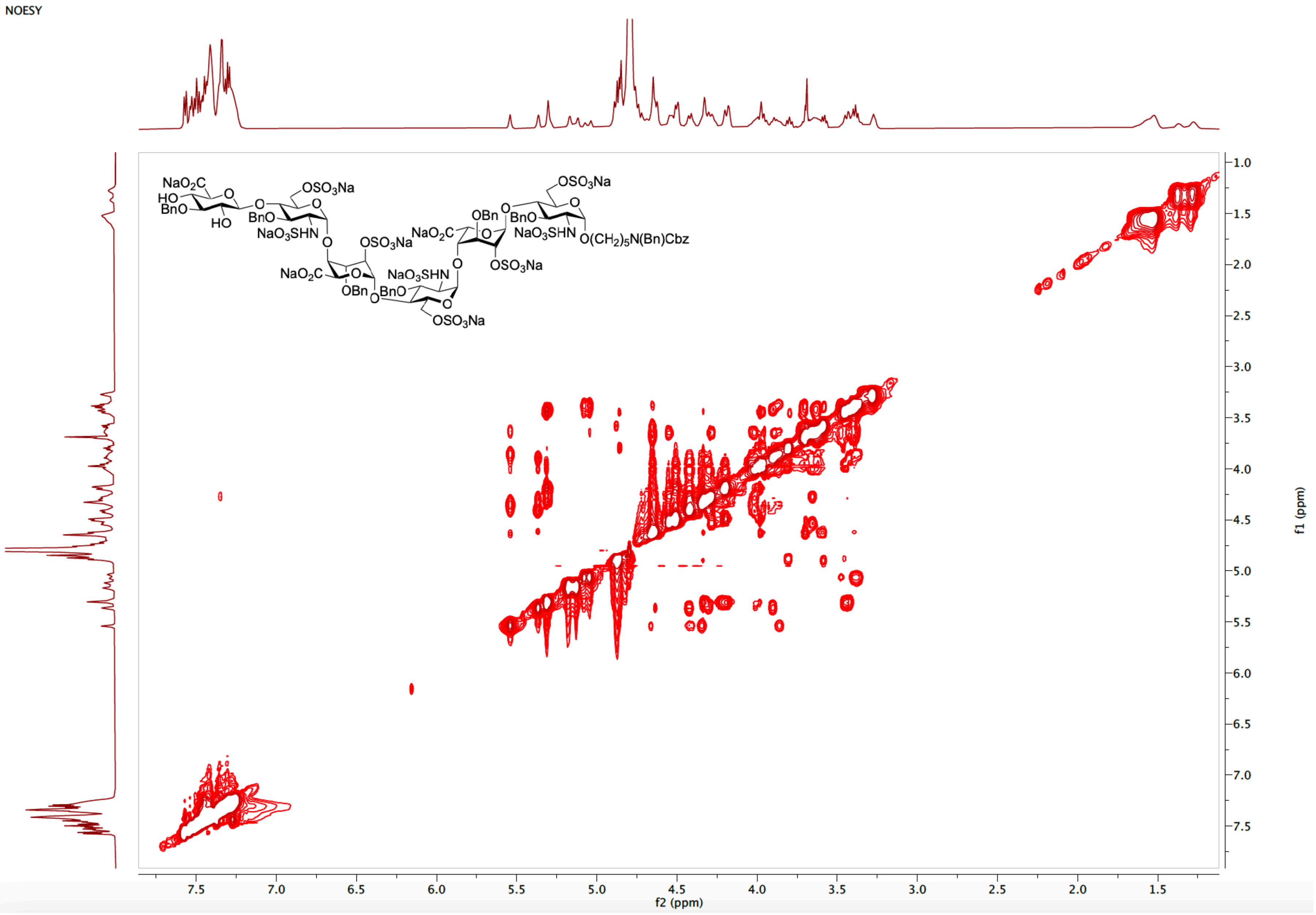

^{1}H NMR spectrum of **1** (600 MHz, D_2O)

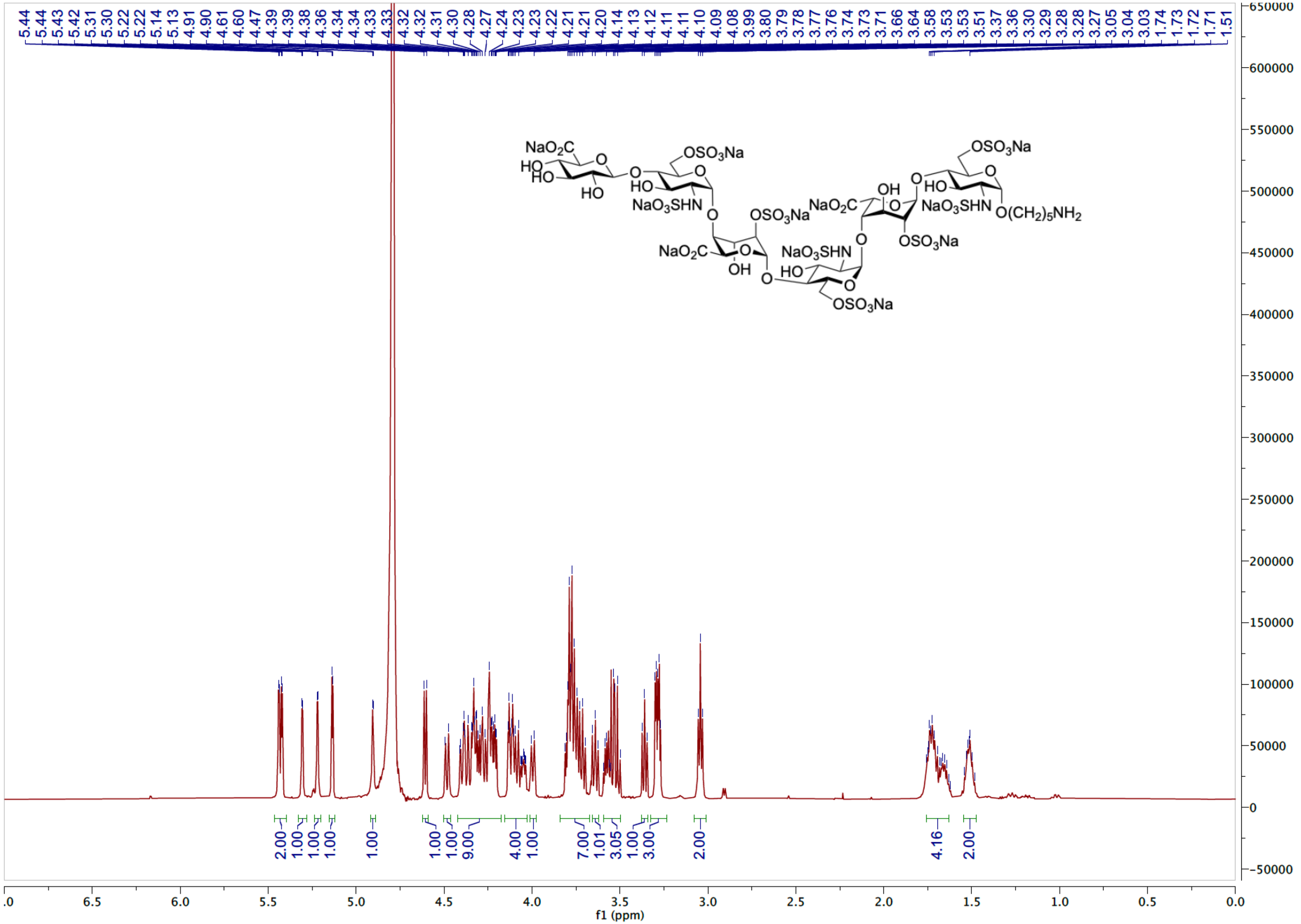

HSQC spectrum of **1** (D_2O)

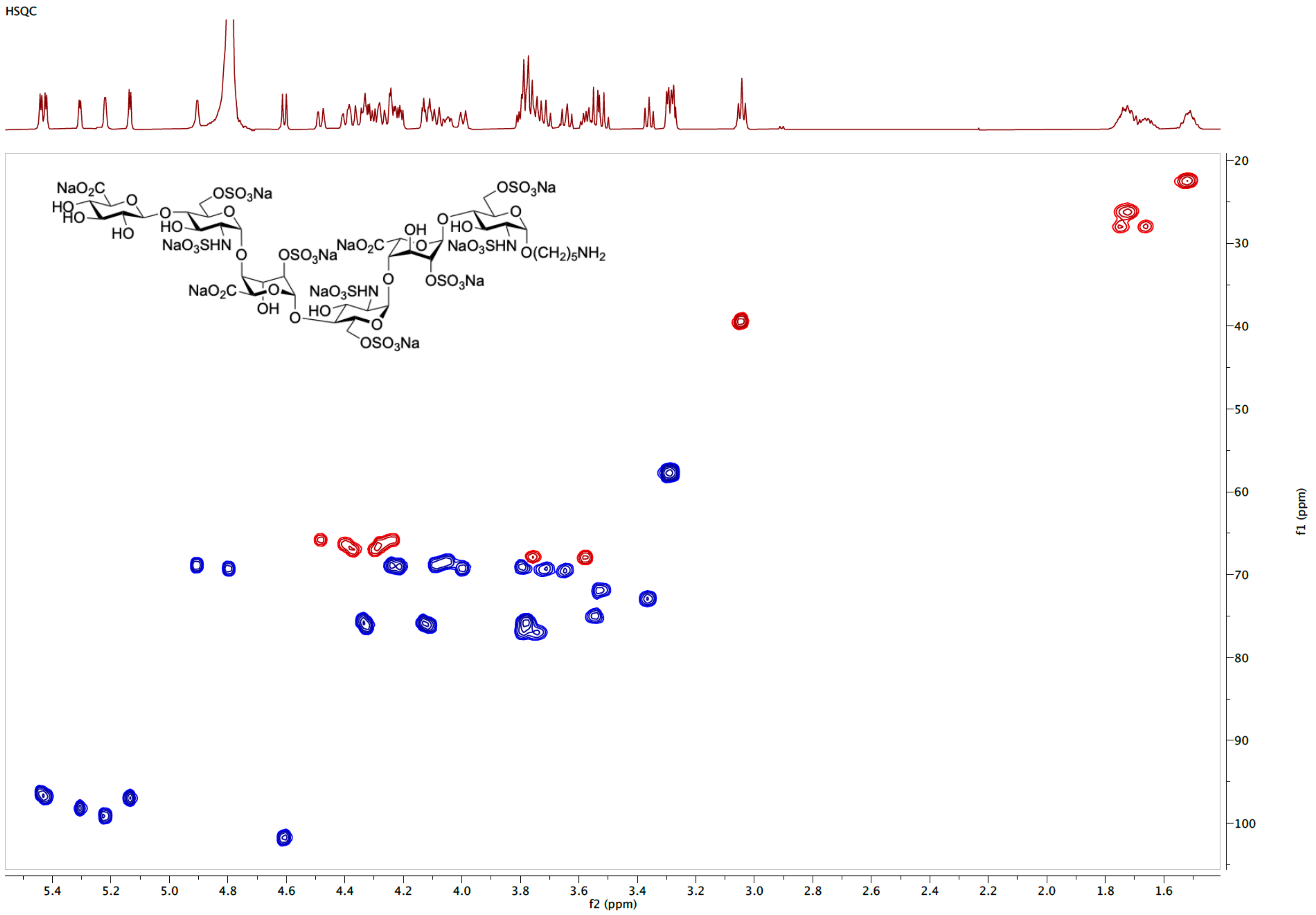

COSY spectrum of **1** (D_2O)

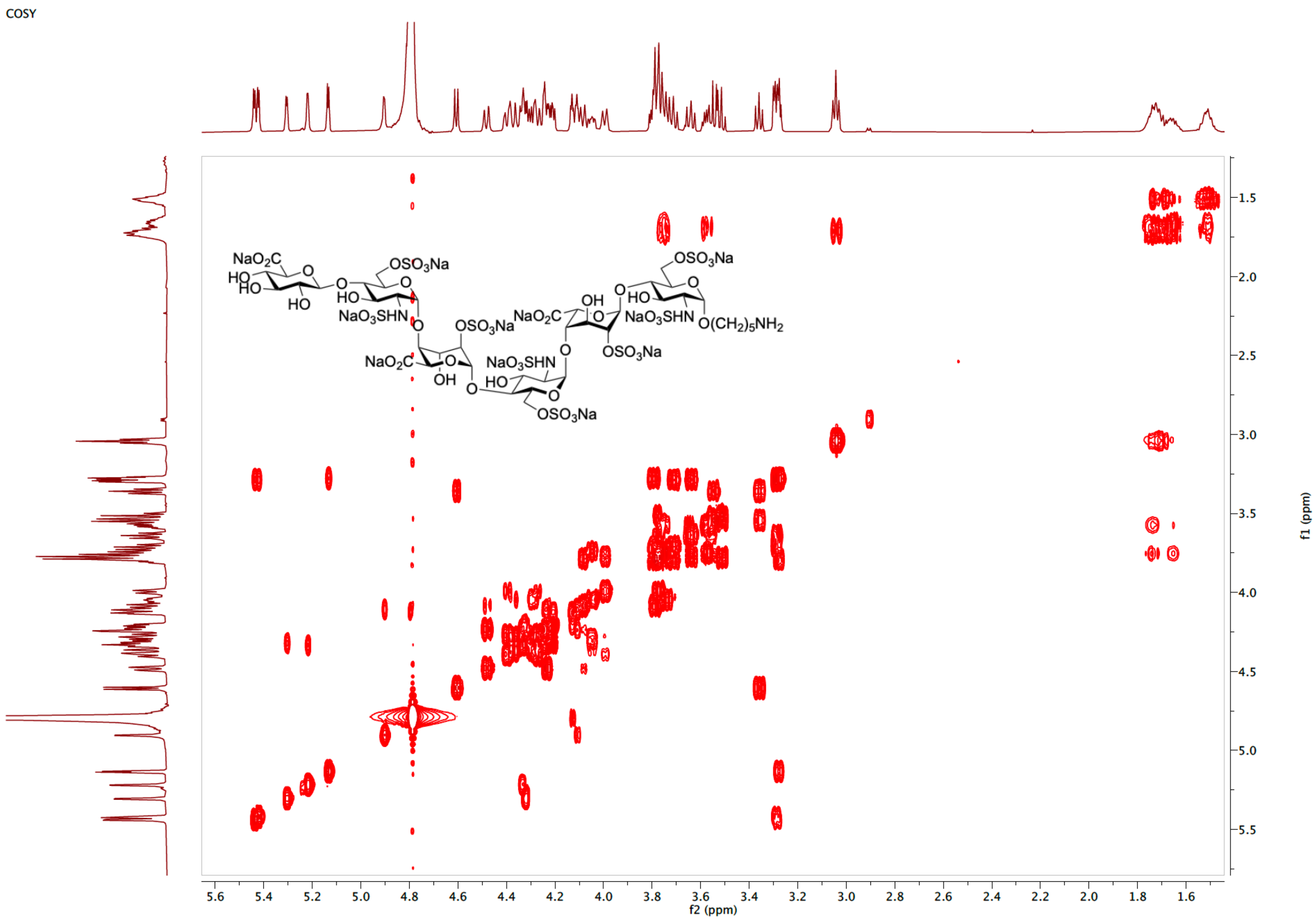

TOCSY spectrum of **1** (D_2O)

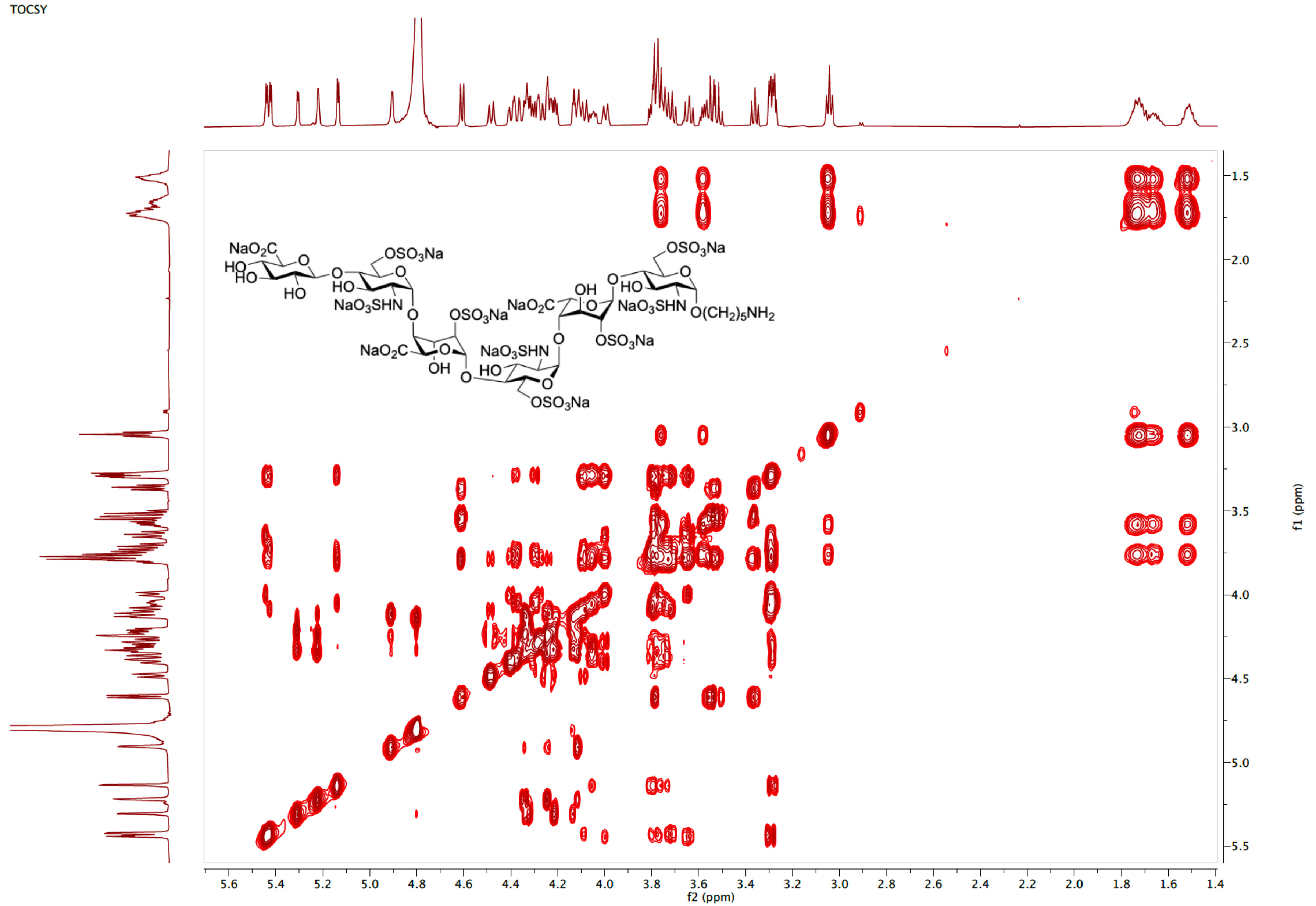

NOESY spectrum of **1** (D_2O)

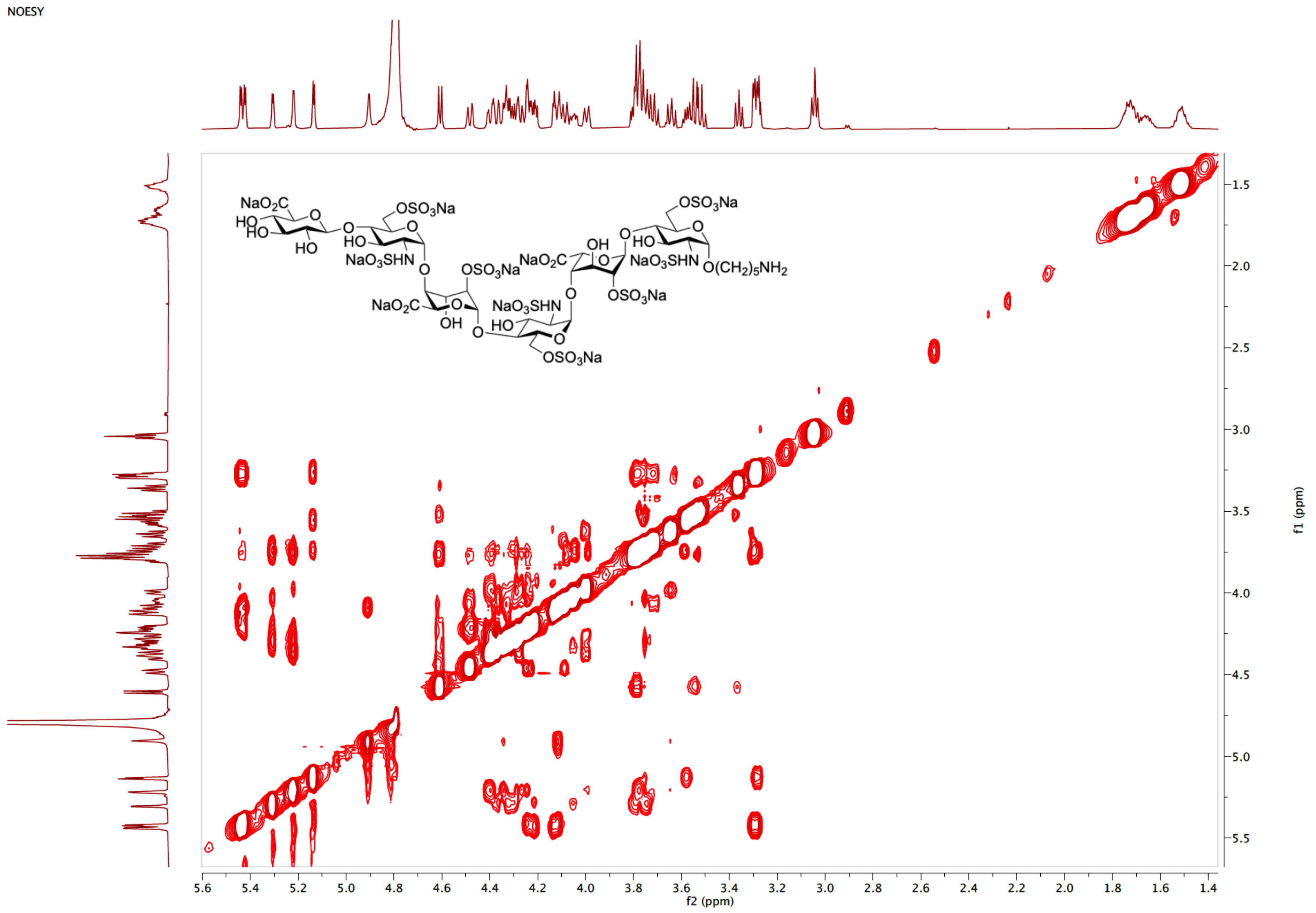

^{1}H NMR spectrum of **2** (600 MHz, D_2O)

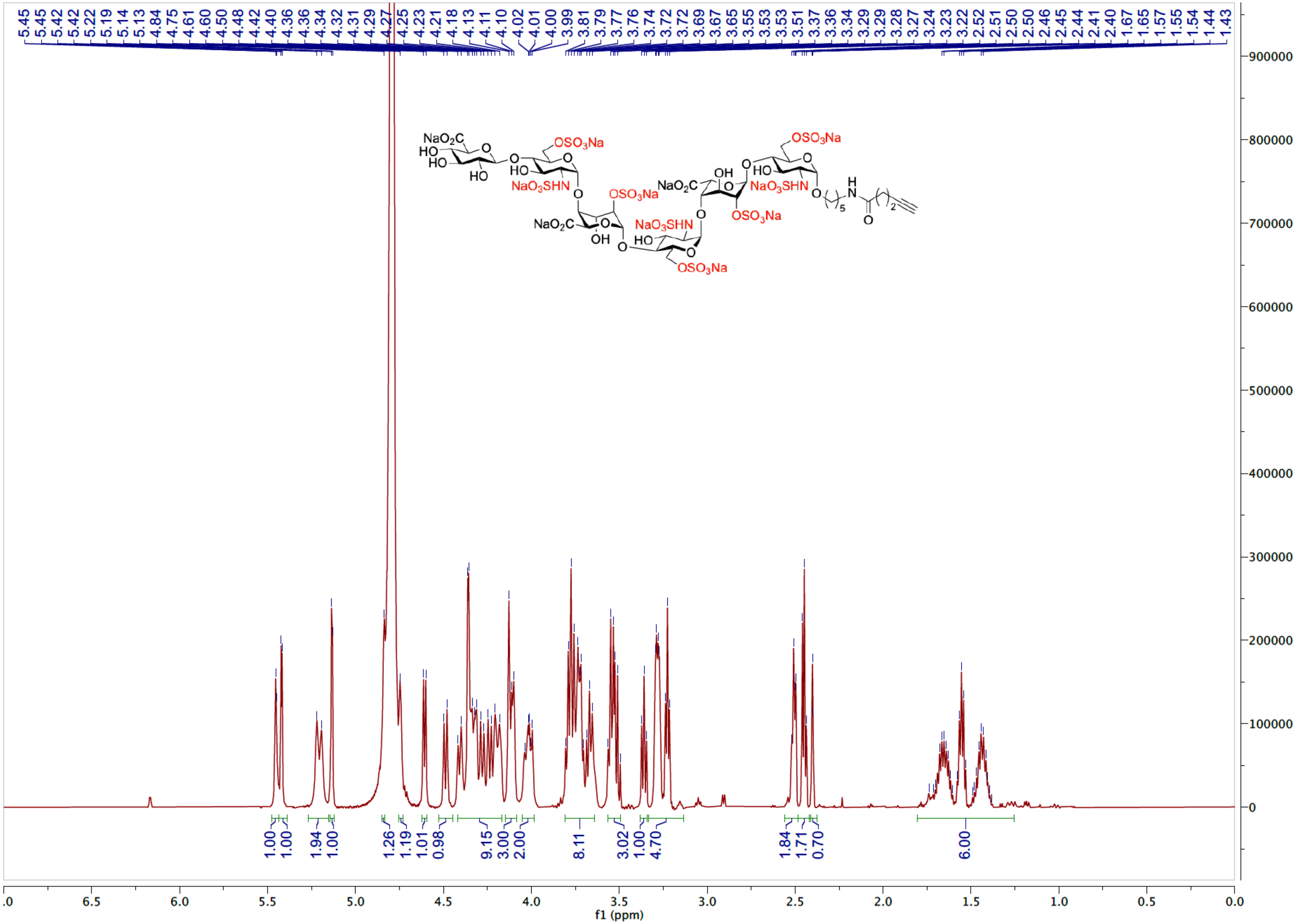

HSQC spectrum of **2** (D_2O)

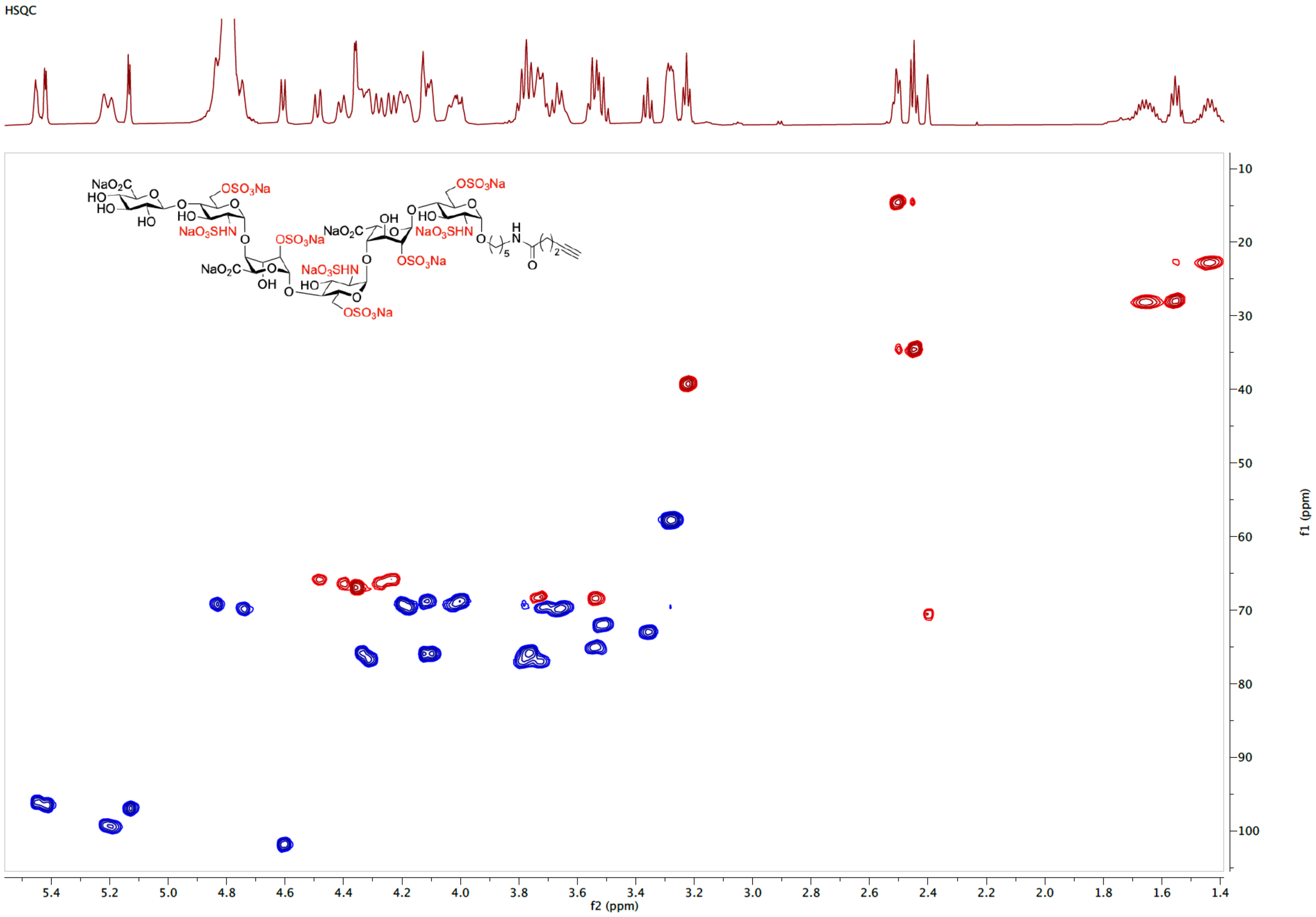

COSY spectrum of **2** (D_2O)

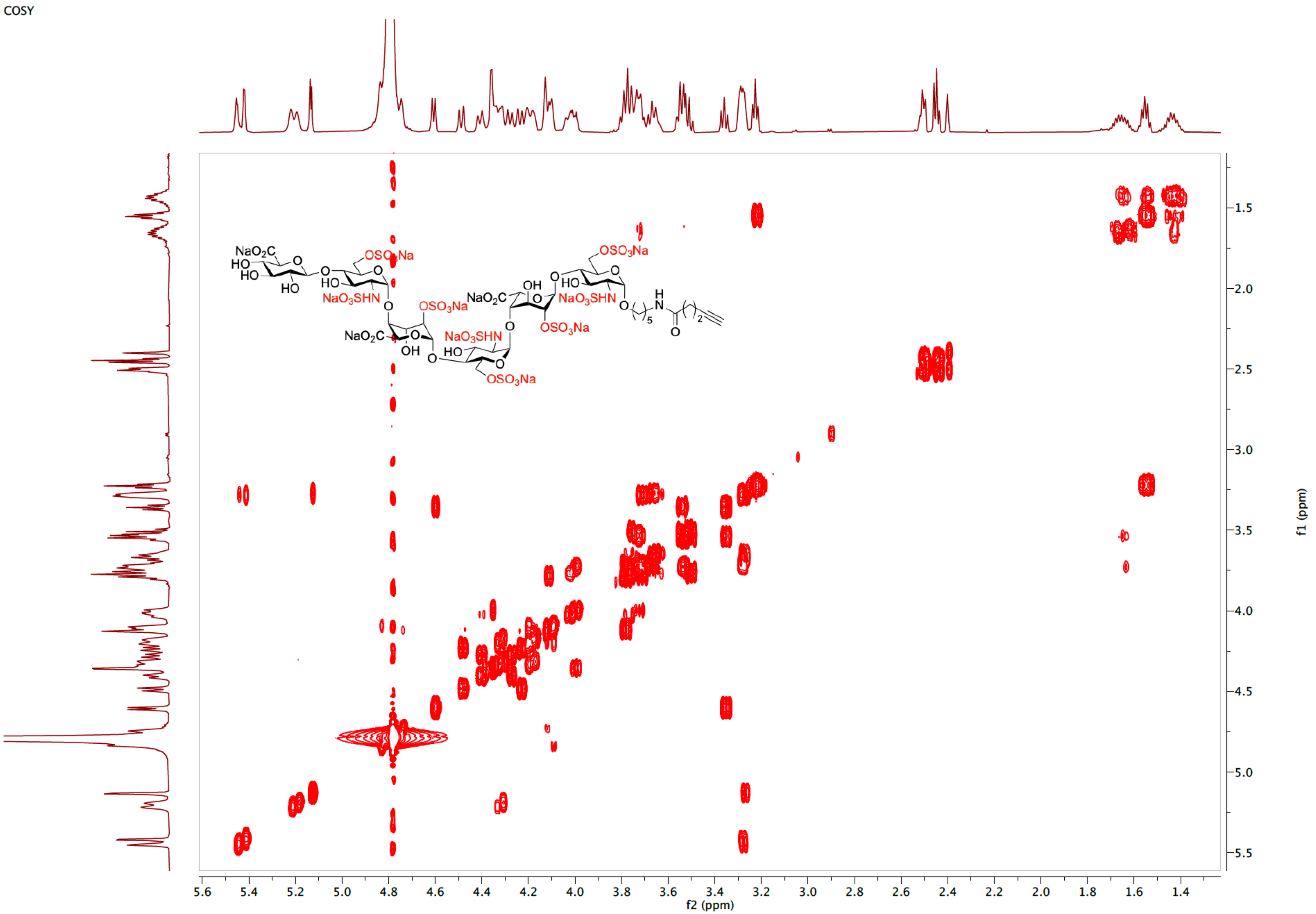

TOCSY spectrum of **2** (D_2O)

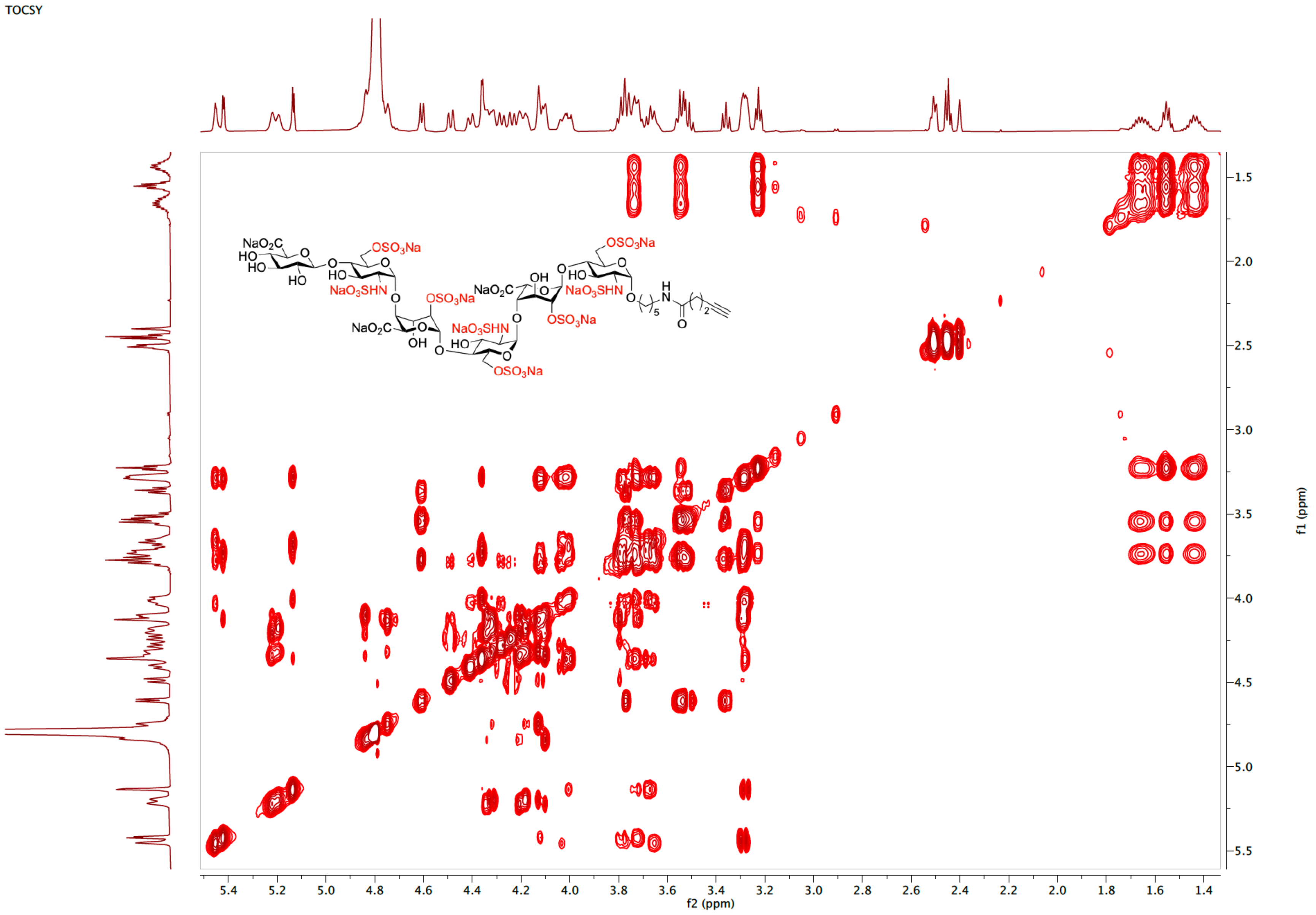

NOESY spectrum of **2** (D_2O)

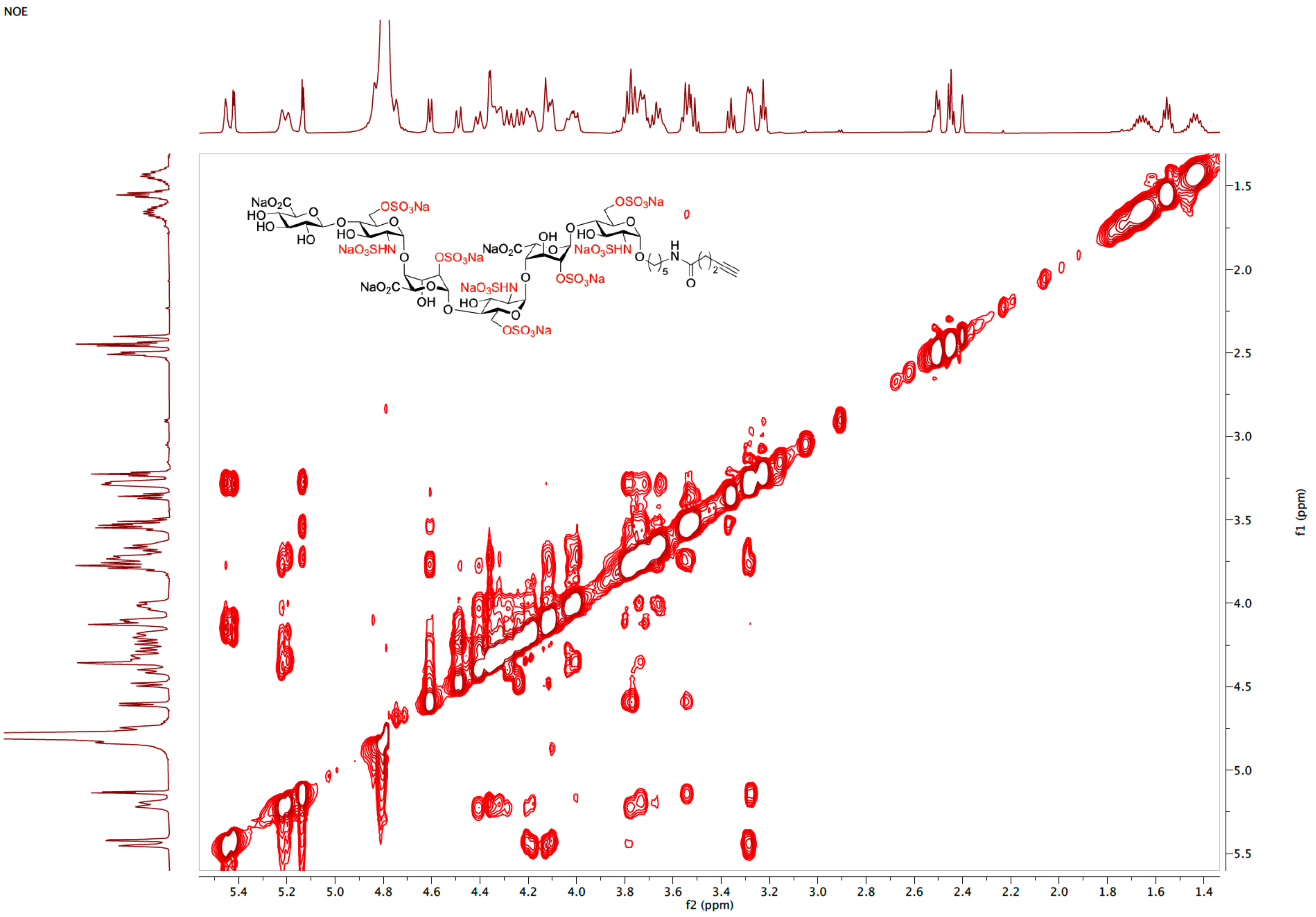

^{1}H NMR spectrum of **3** (600 MHz, D_2O)

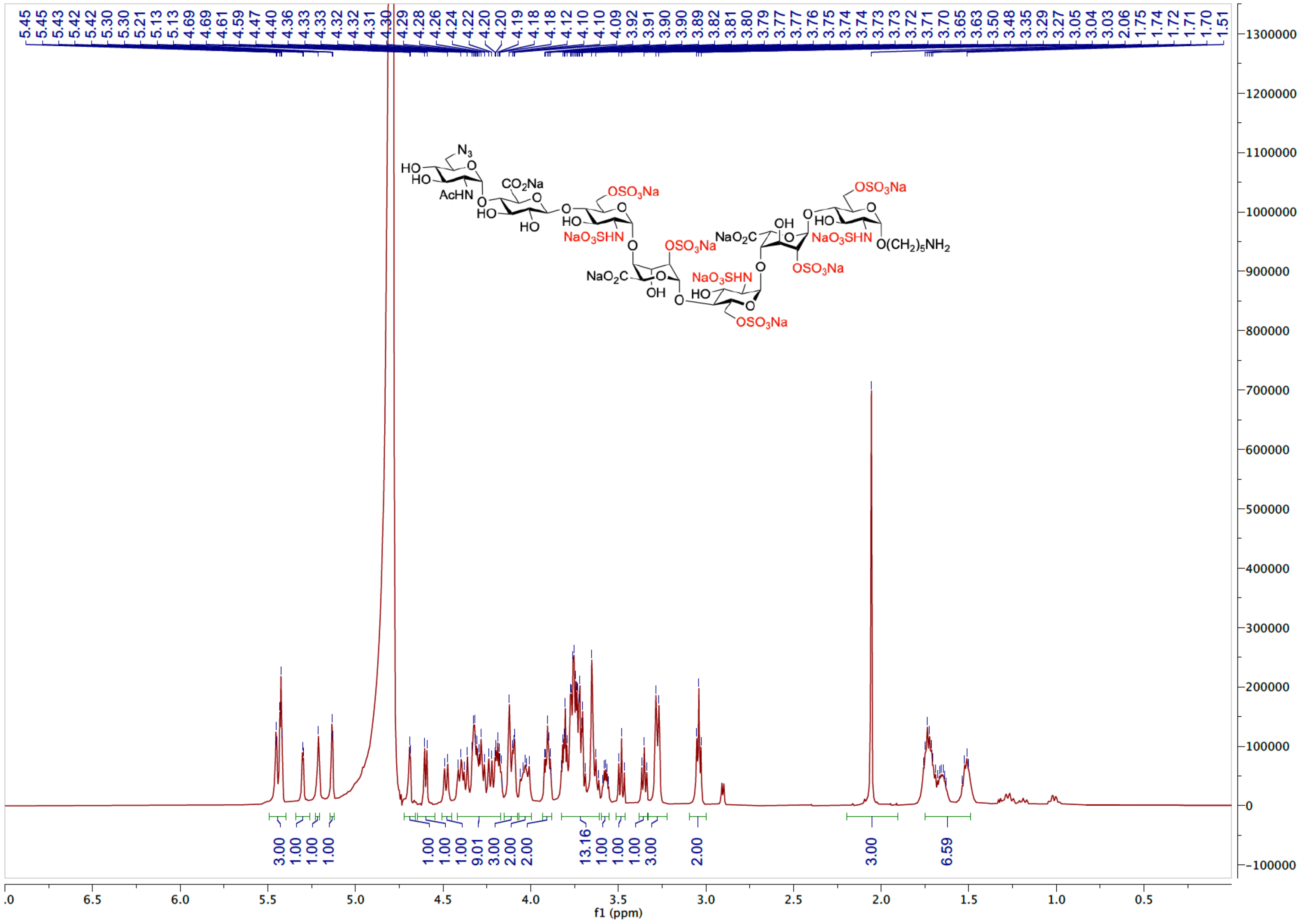

HSQC spectrum of **3** (D_2O)

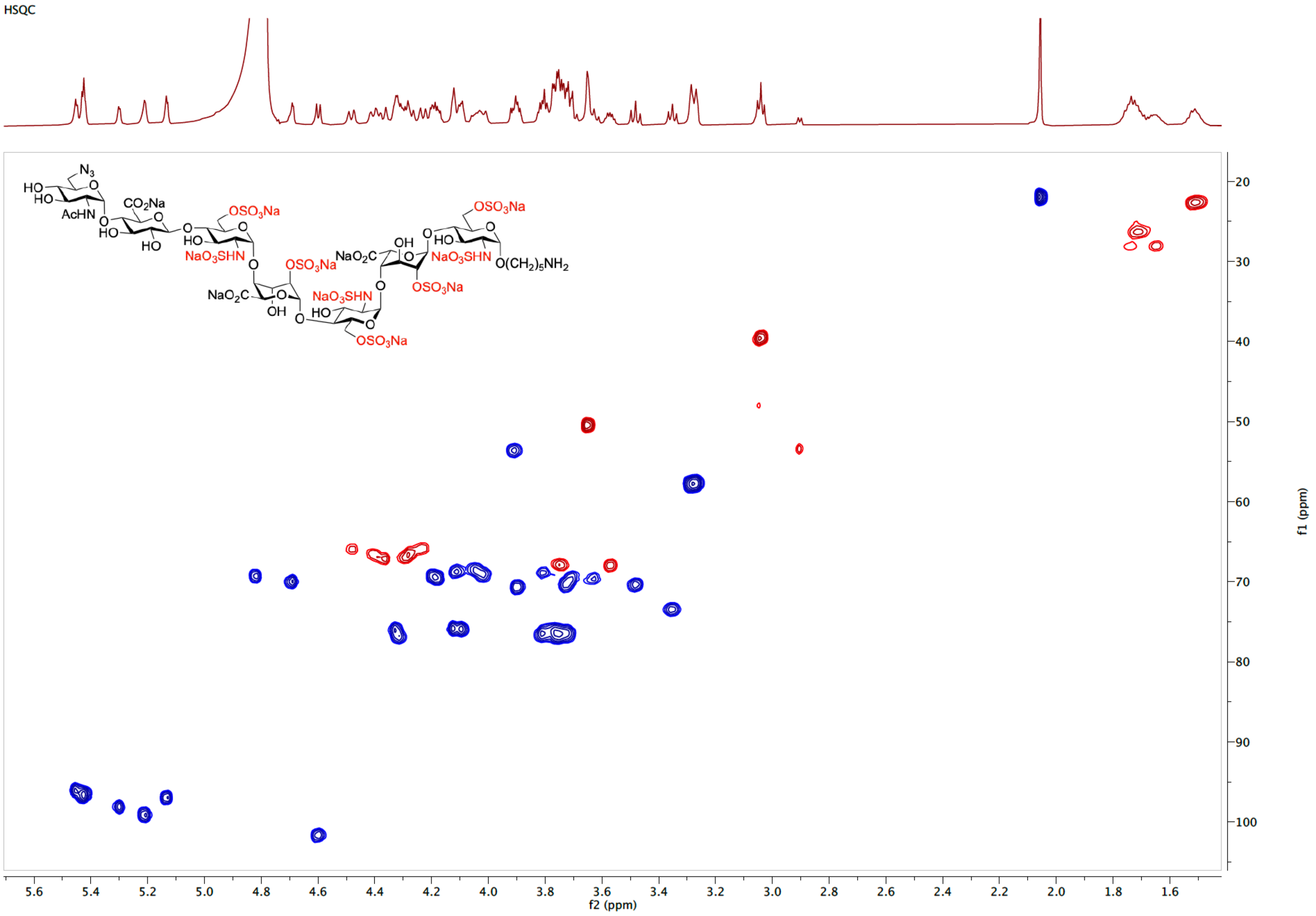

COSY spectrum of **3** (D_2O)

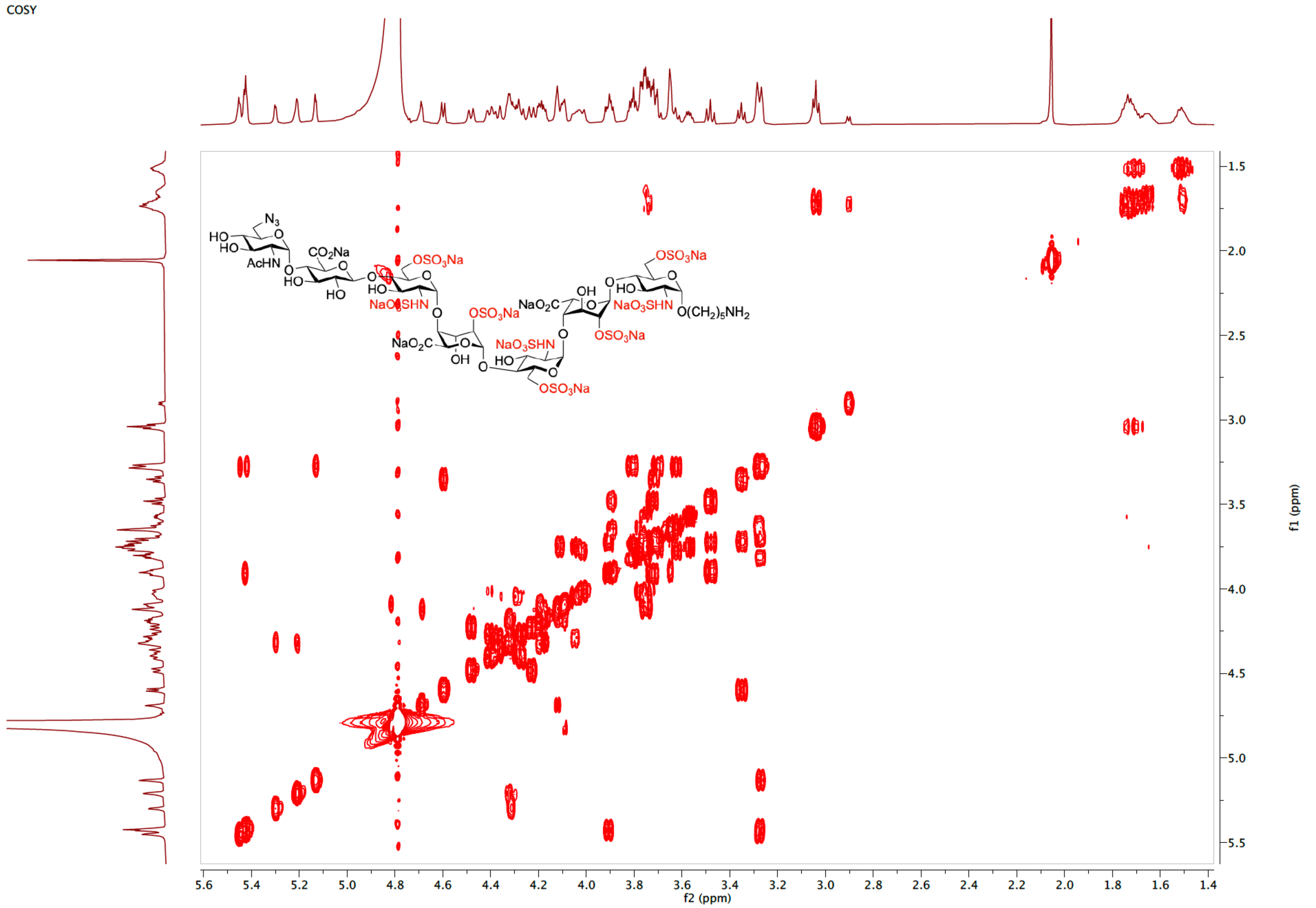

TOCSY spectrum of **3** (D_2O)

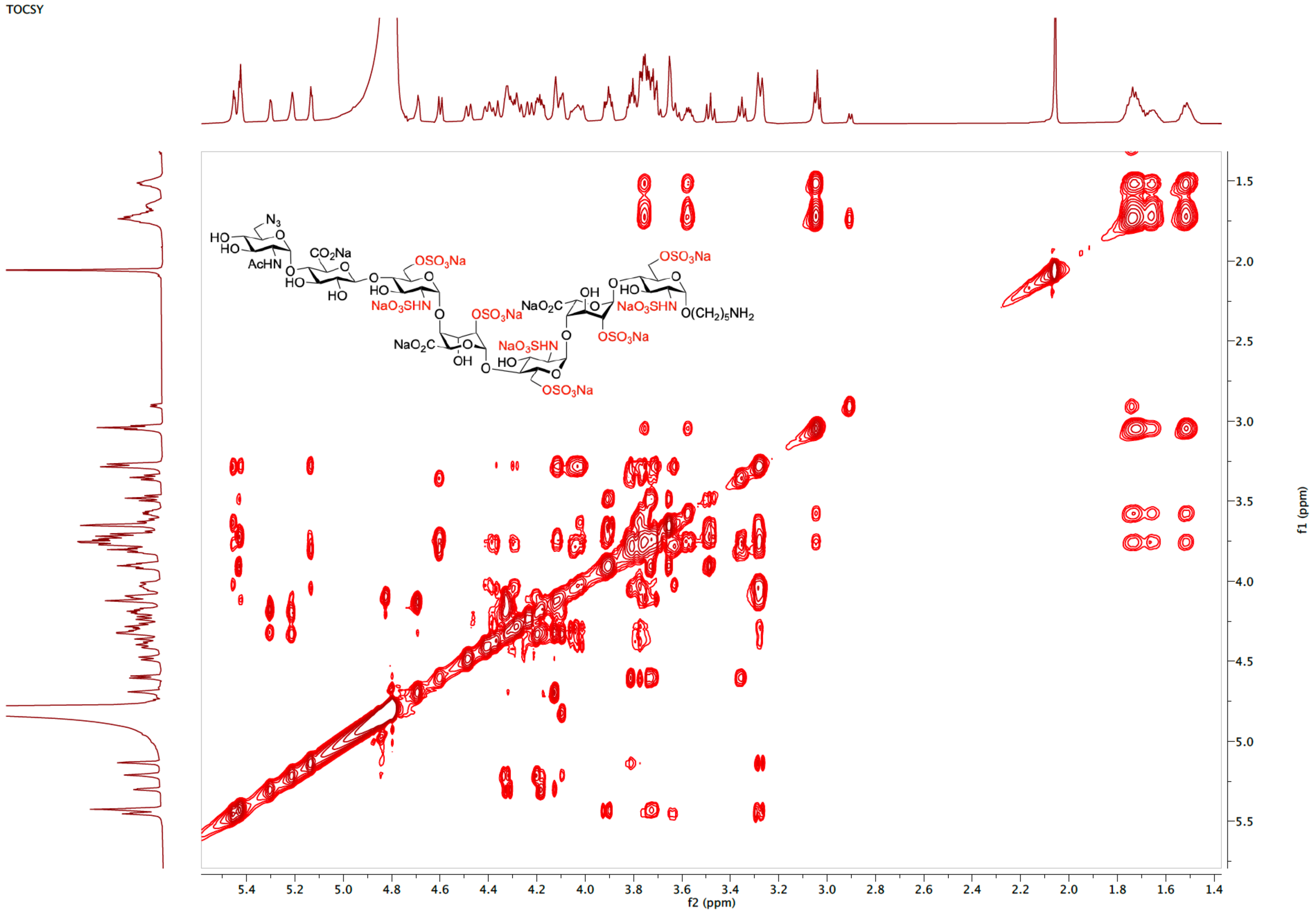

NOESY spectrum of **3** (D_2O)

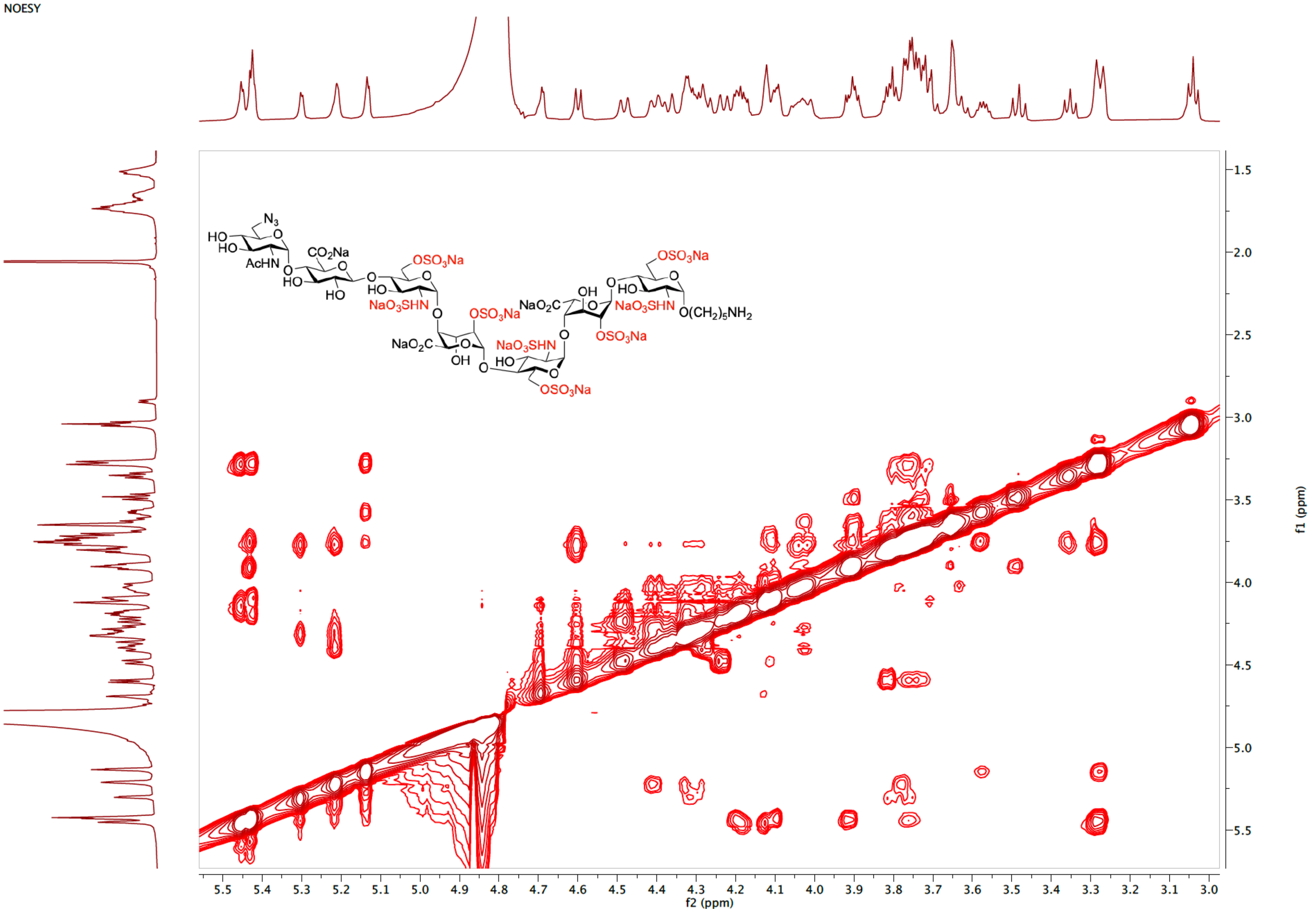

^{1}H NMR spectrum of **4** (600 MHz, D_2O)

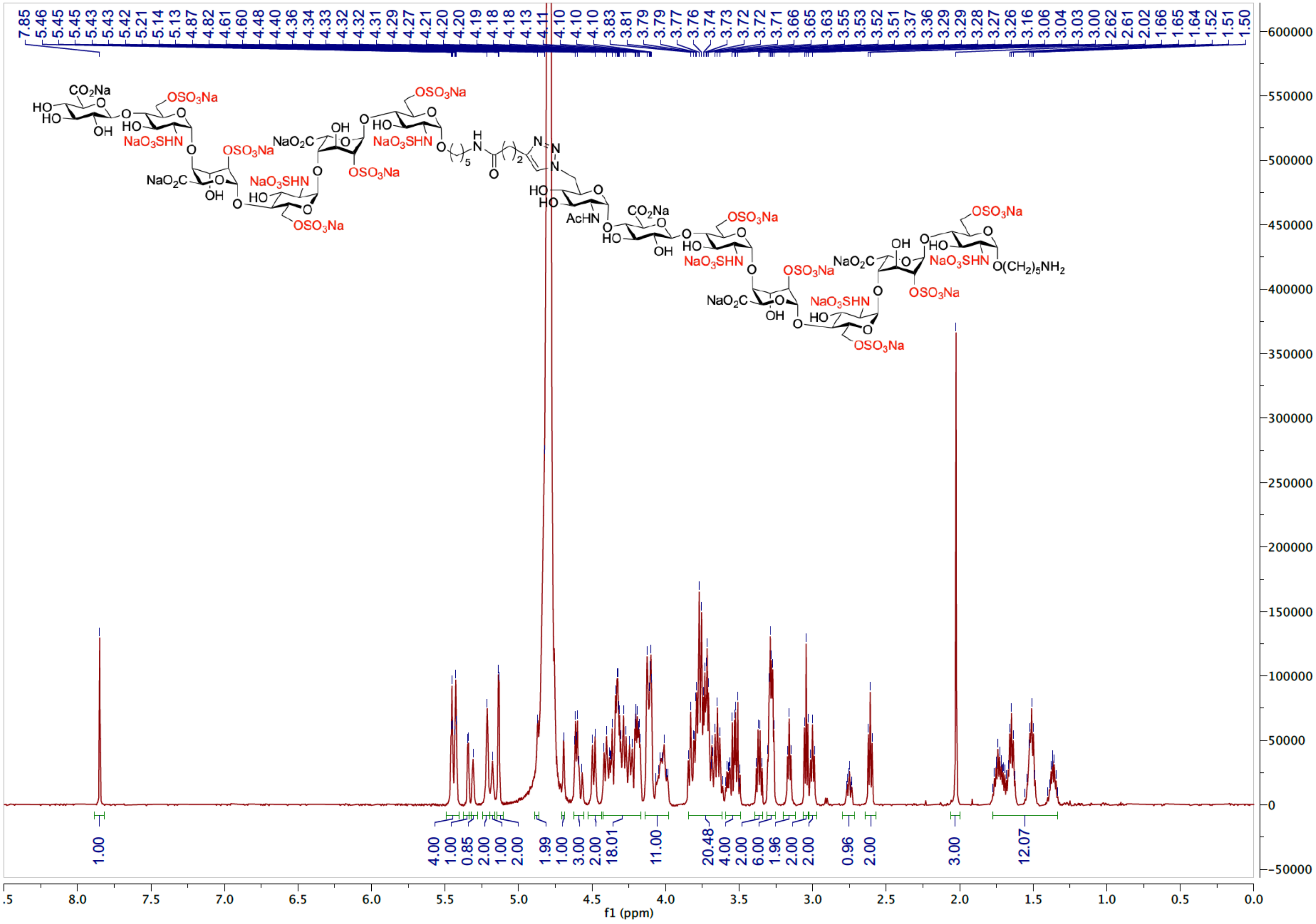

HSQC spectrum of **4** (D_2O)

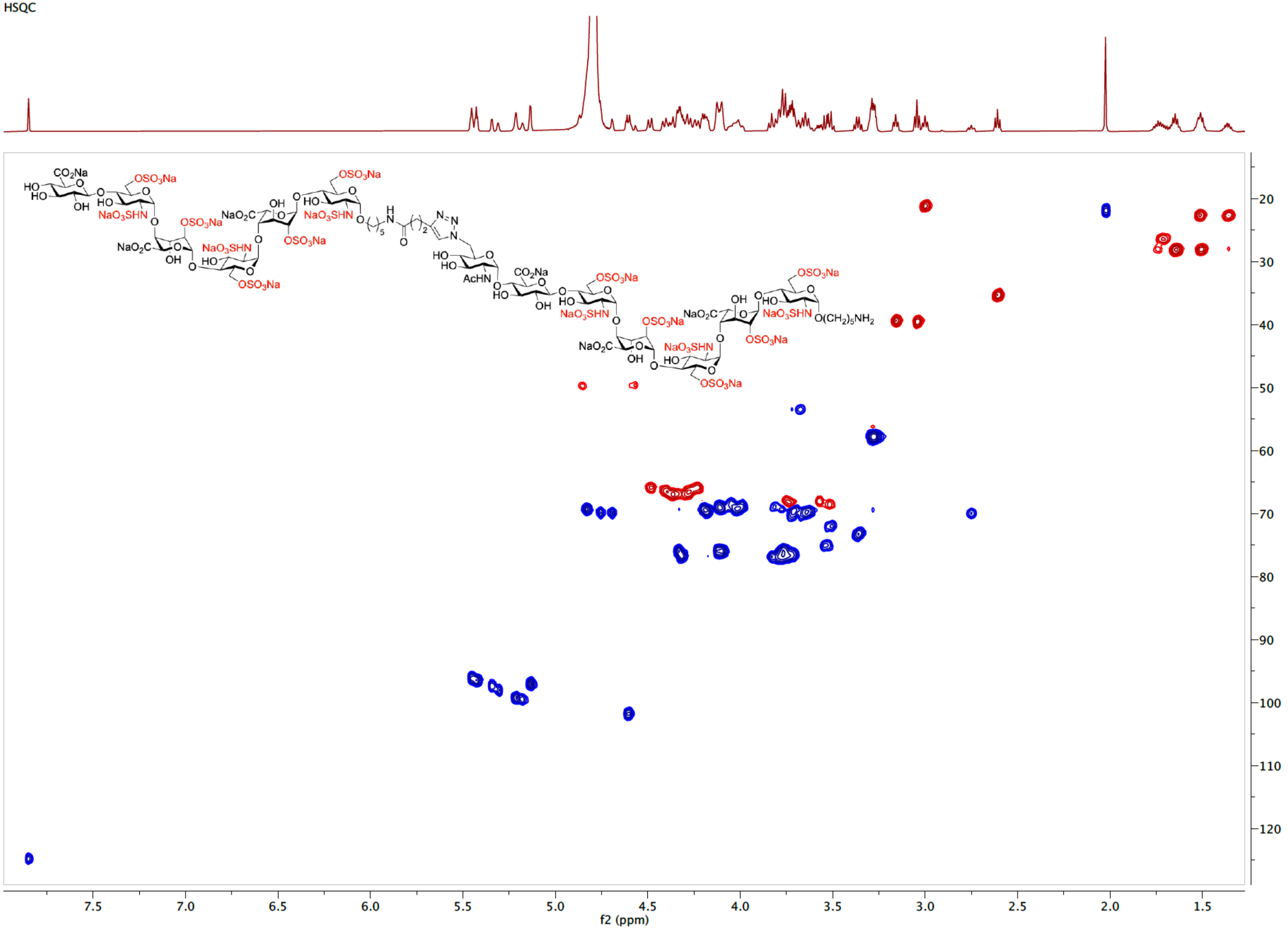

COSY spectrum of **4** (D_2O)

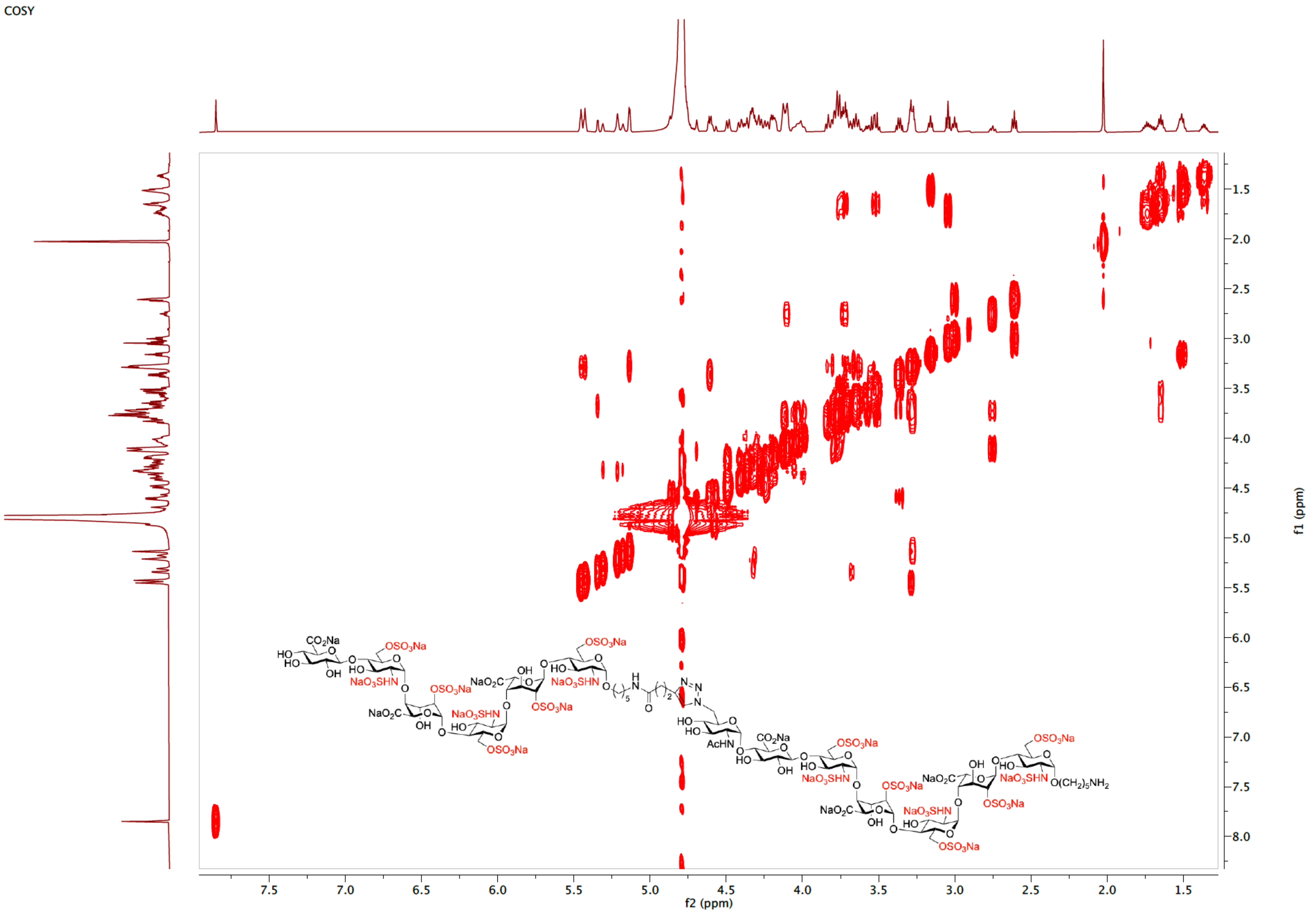

TOCSY spectrum of **4** (D_2O)

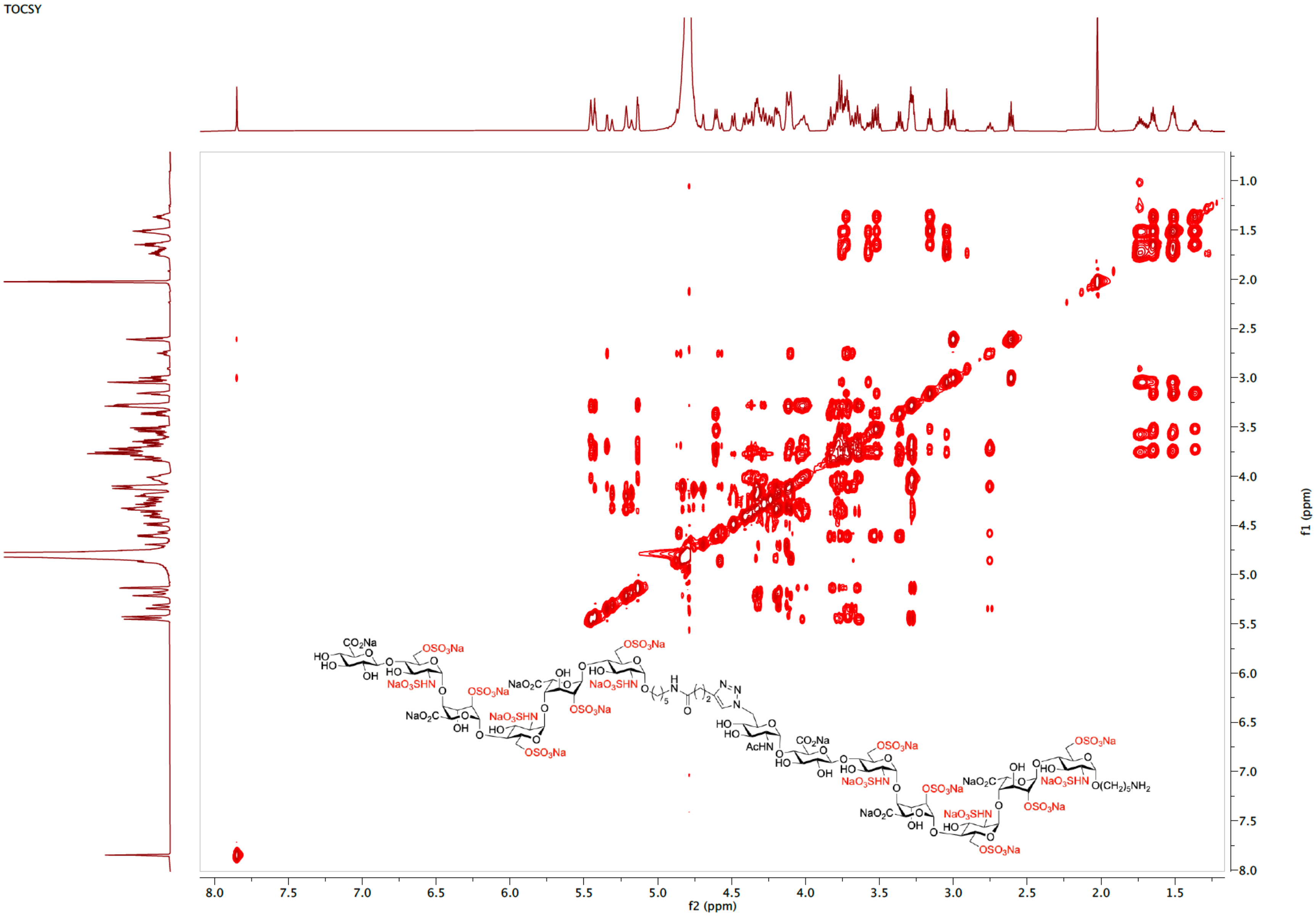

NOESY spectrum of **4** (D_2O)

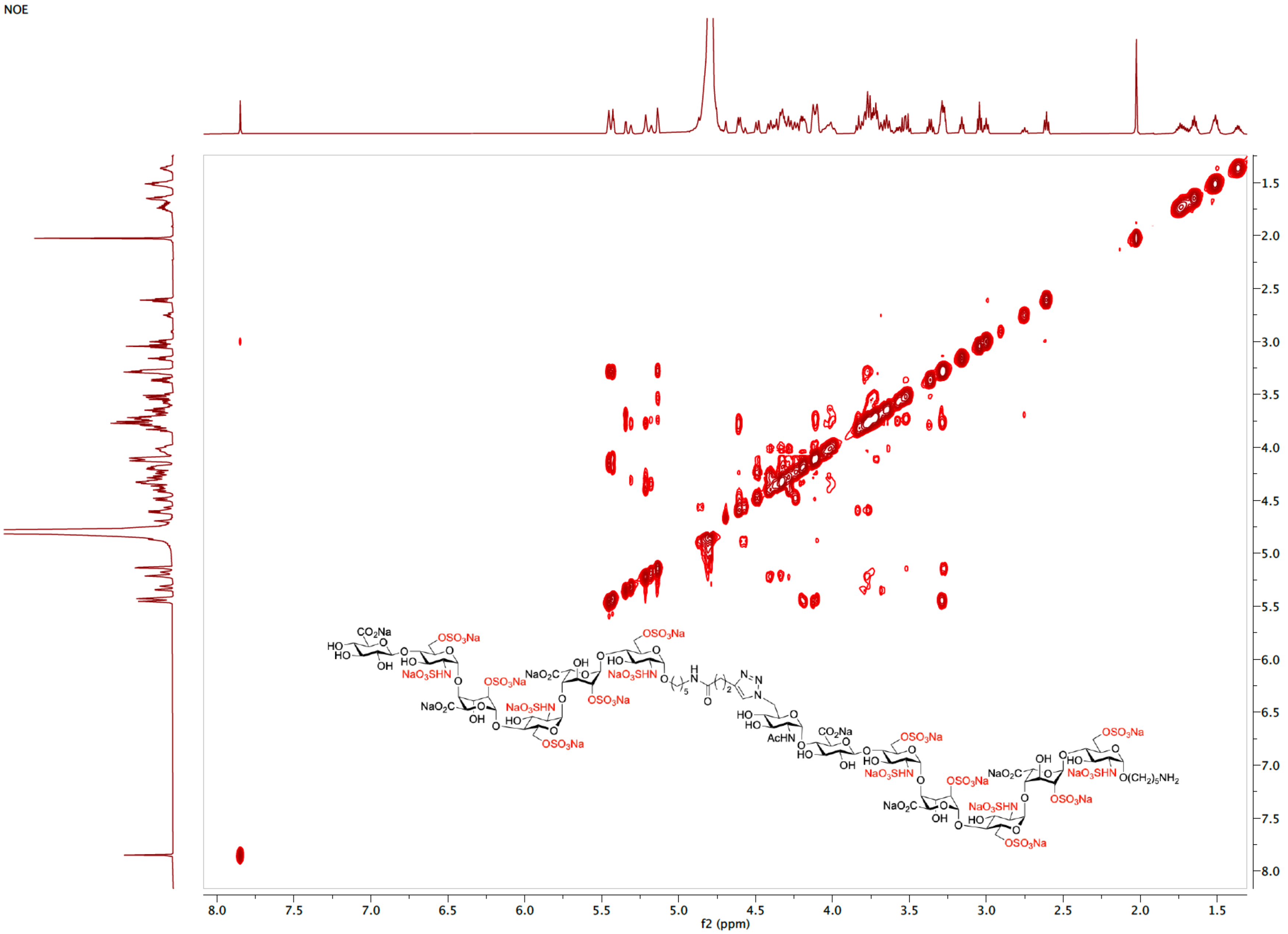

ESI-MS (negative) of **4**

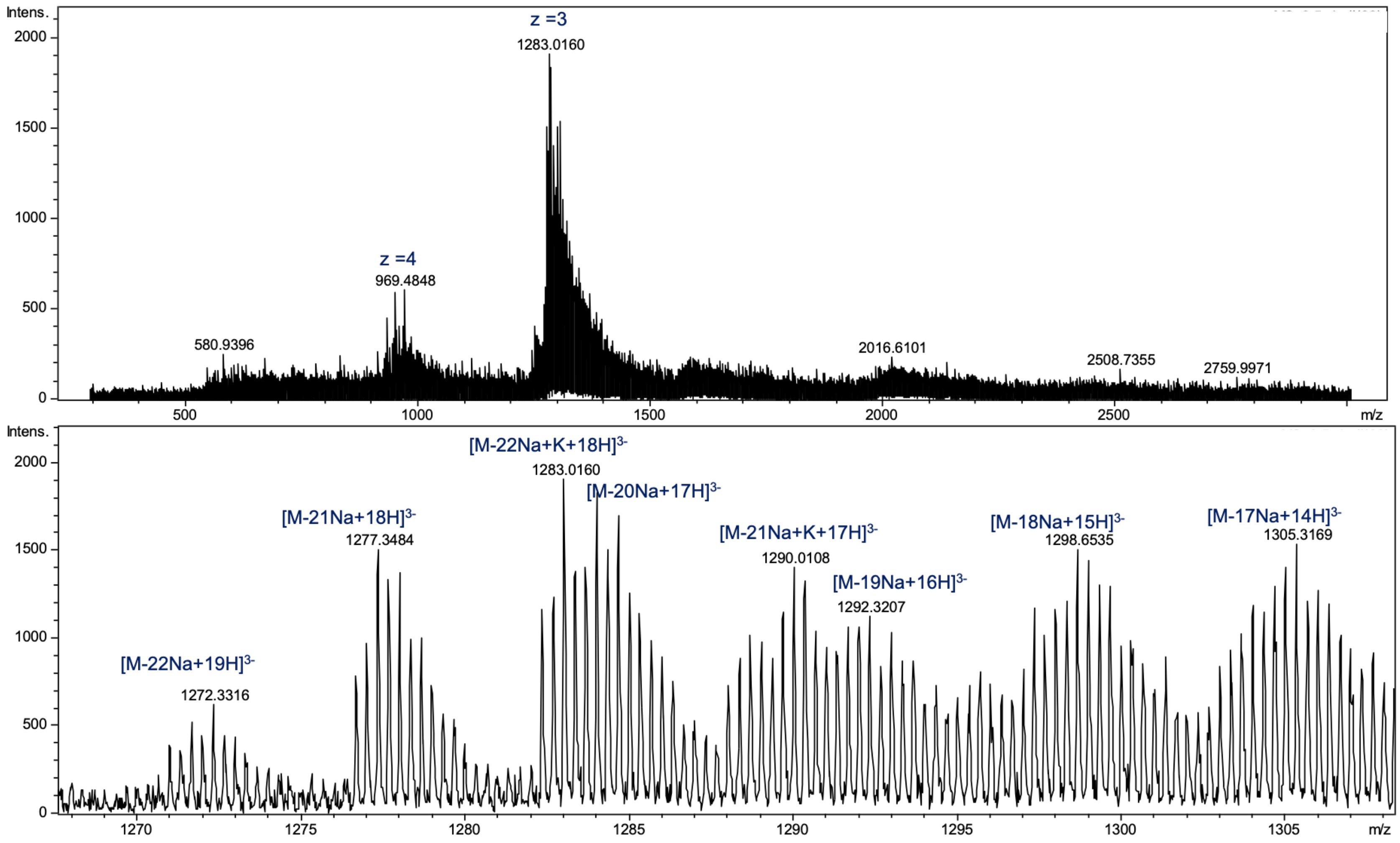

^{1}H NMR spectrum of **12** (600 MHz, D_2O)

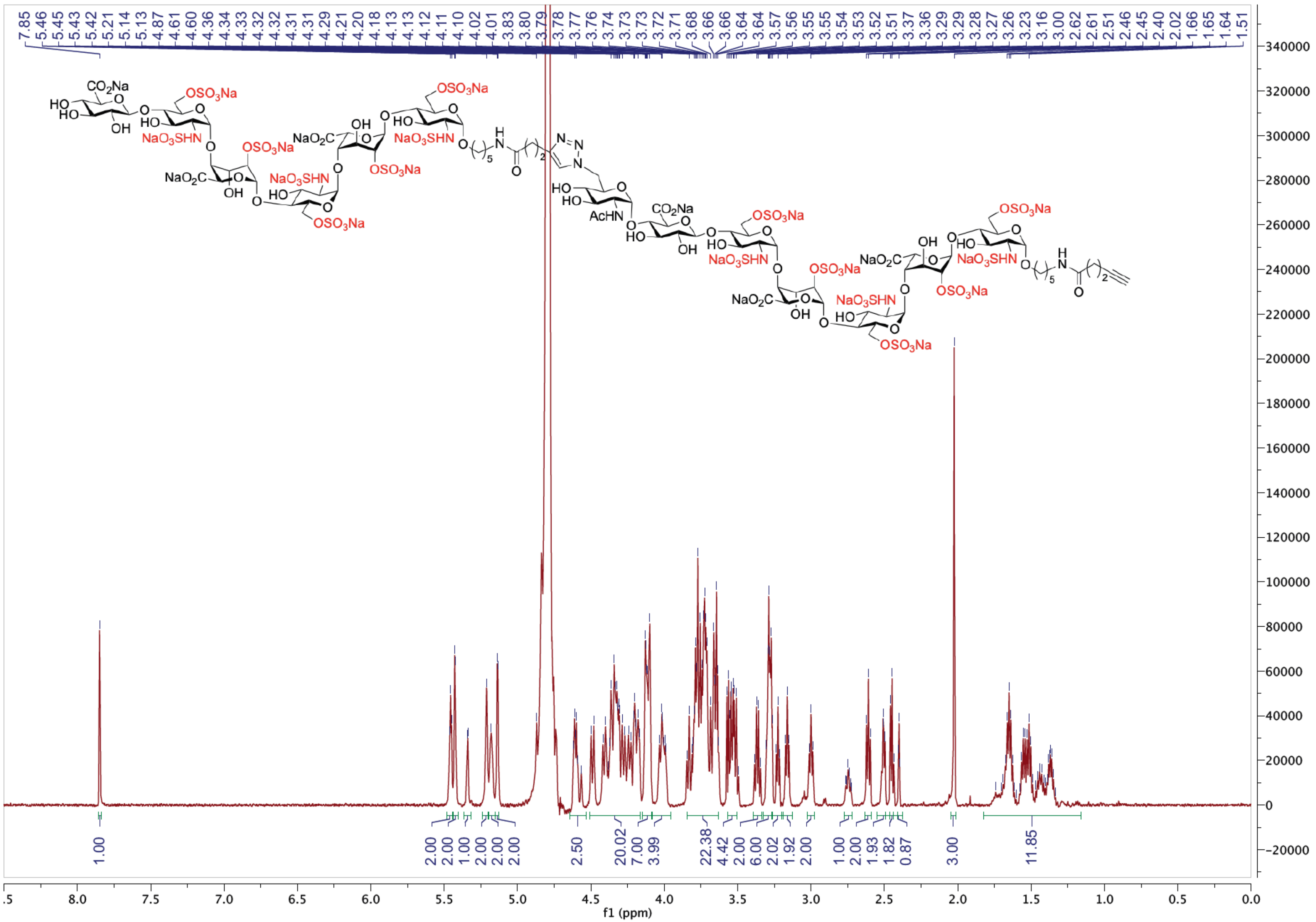

HSQC spectrum of **12** (D_2O)

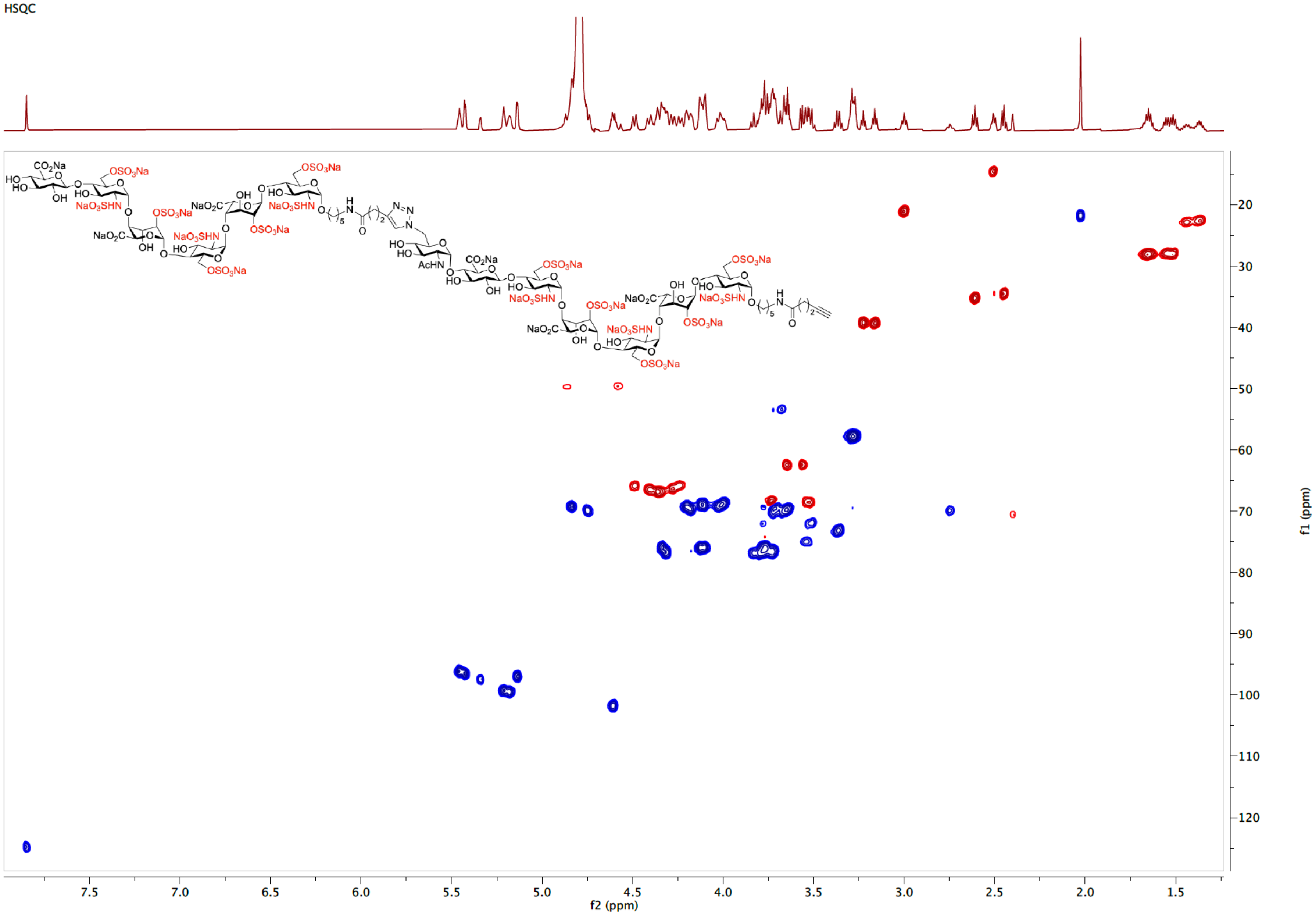

COSY spectrum of **12** (D_2O)

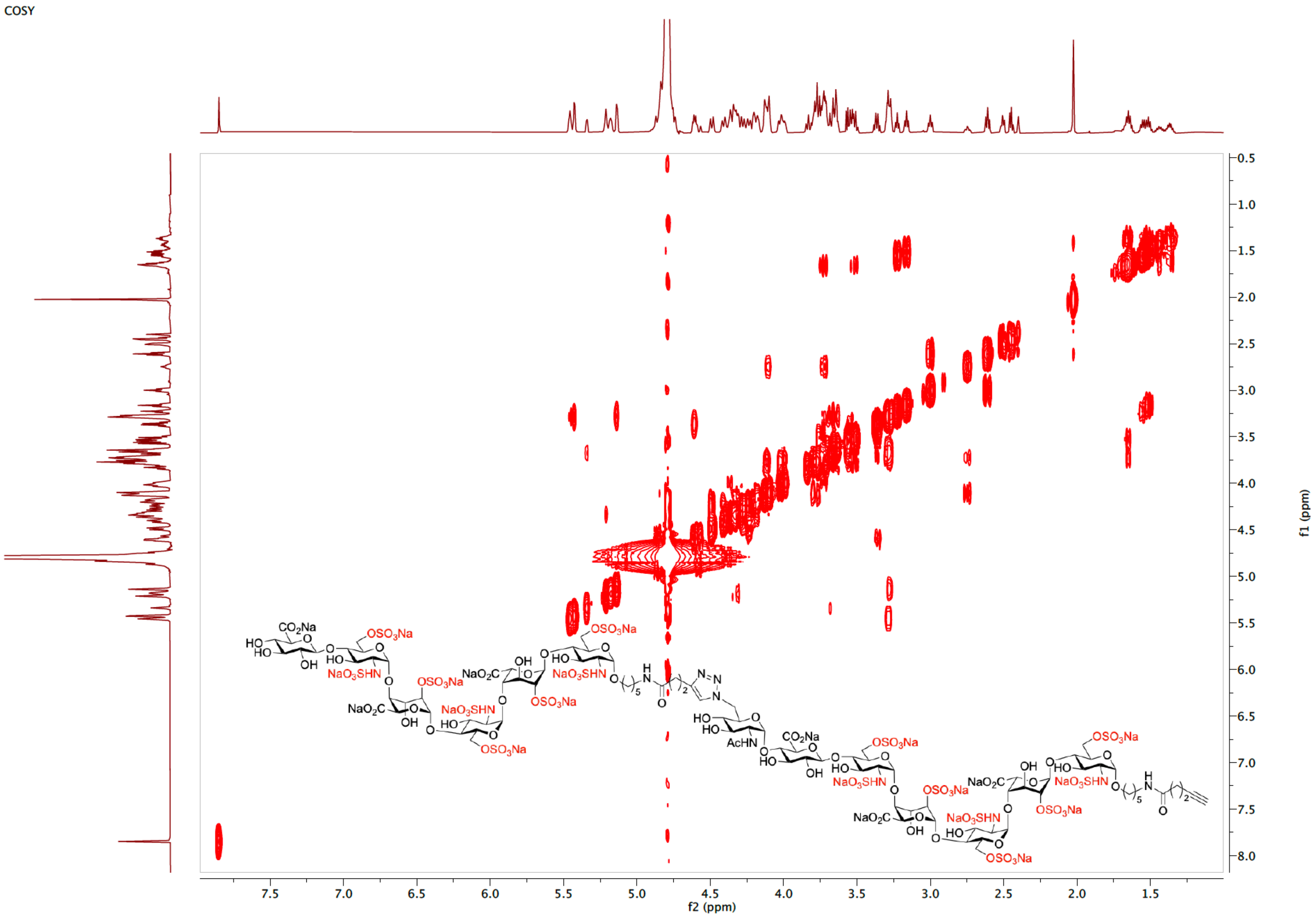

TOCSY spectrum of **12** (D_2O)

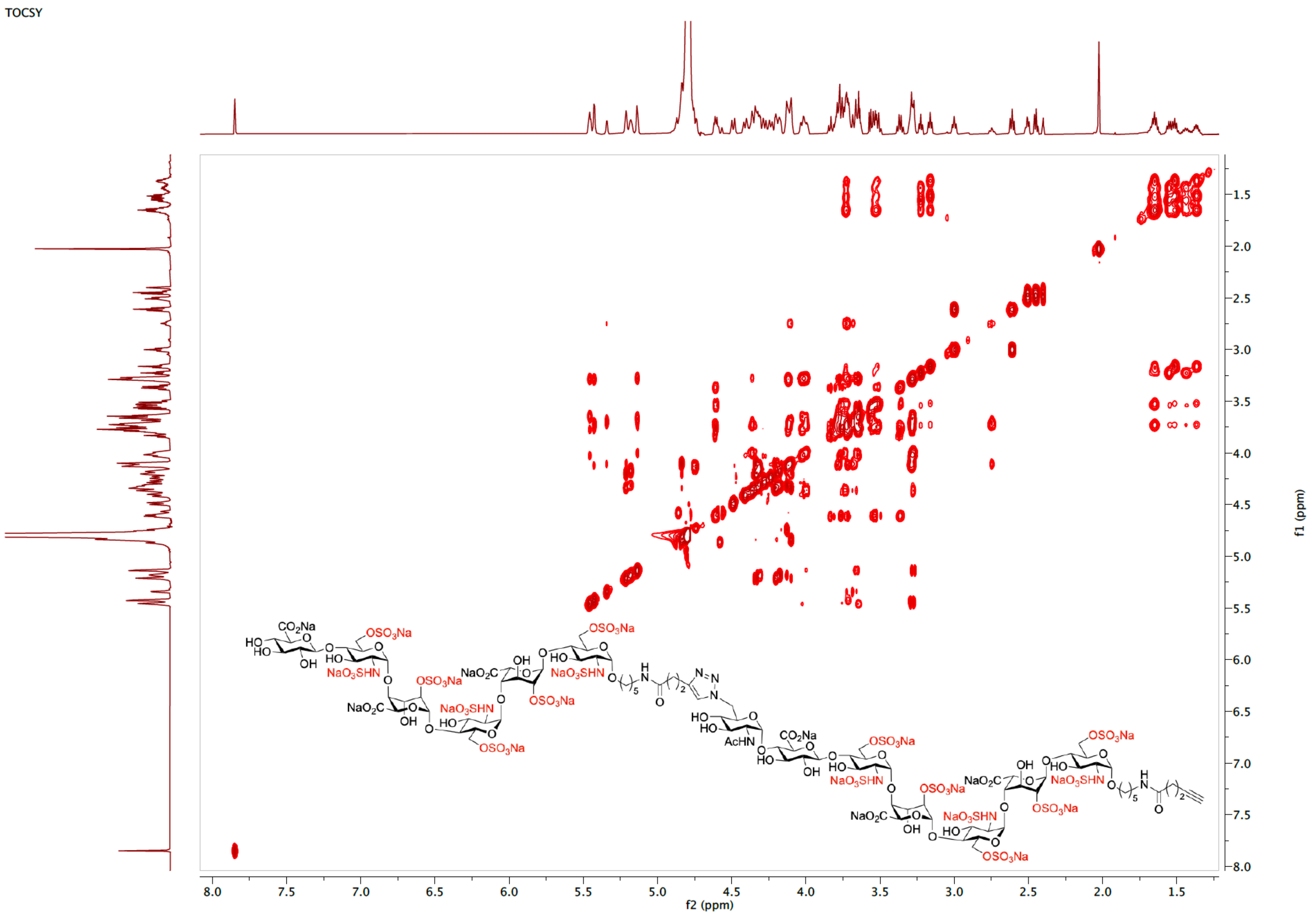

NOESY spectrum of **12** (D_2O)

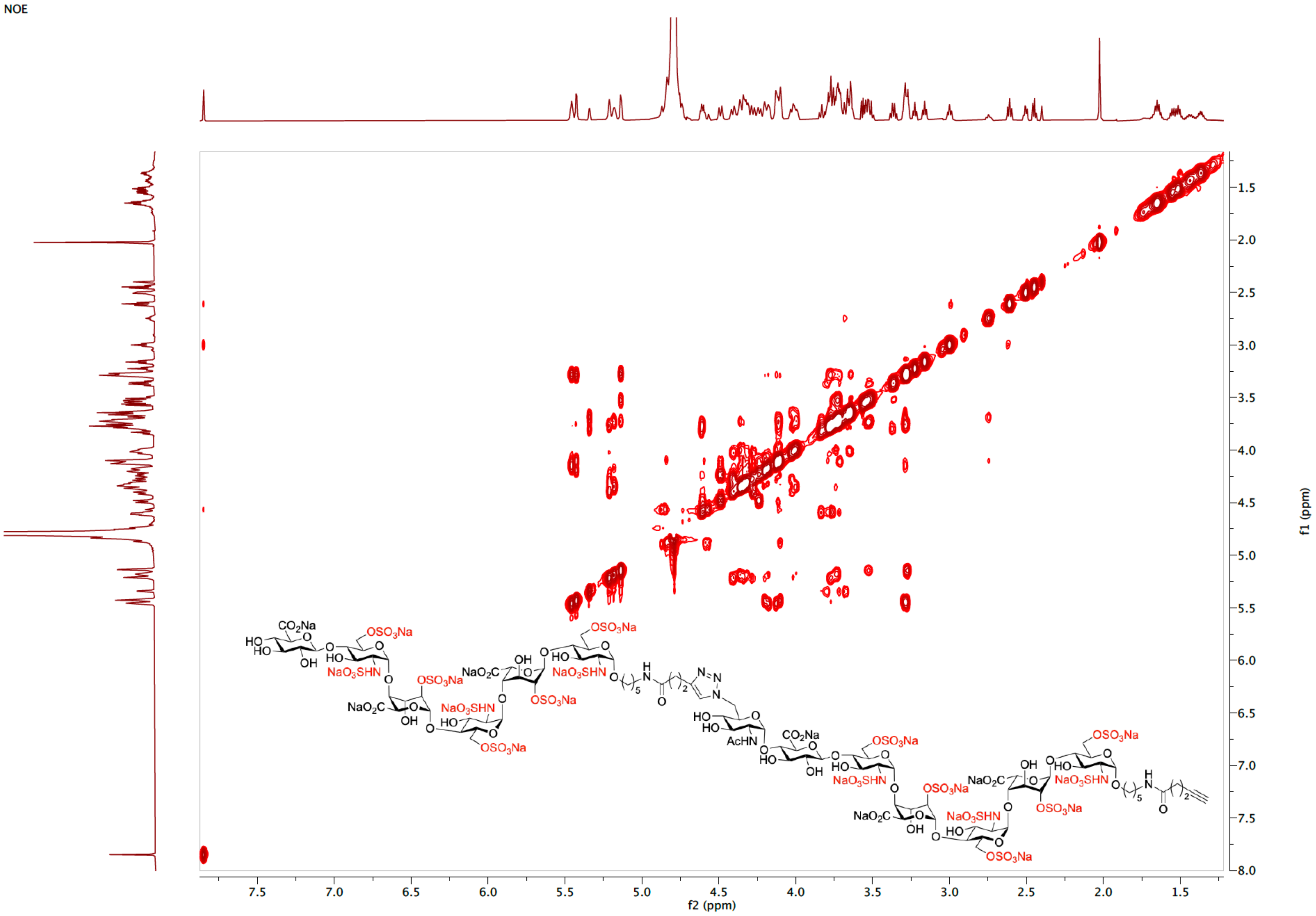

ESI-MS (negative) of **12**

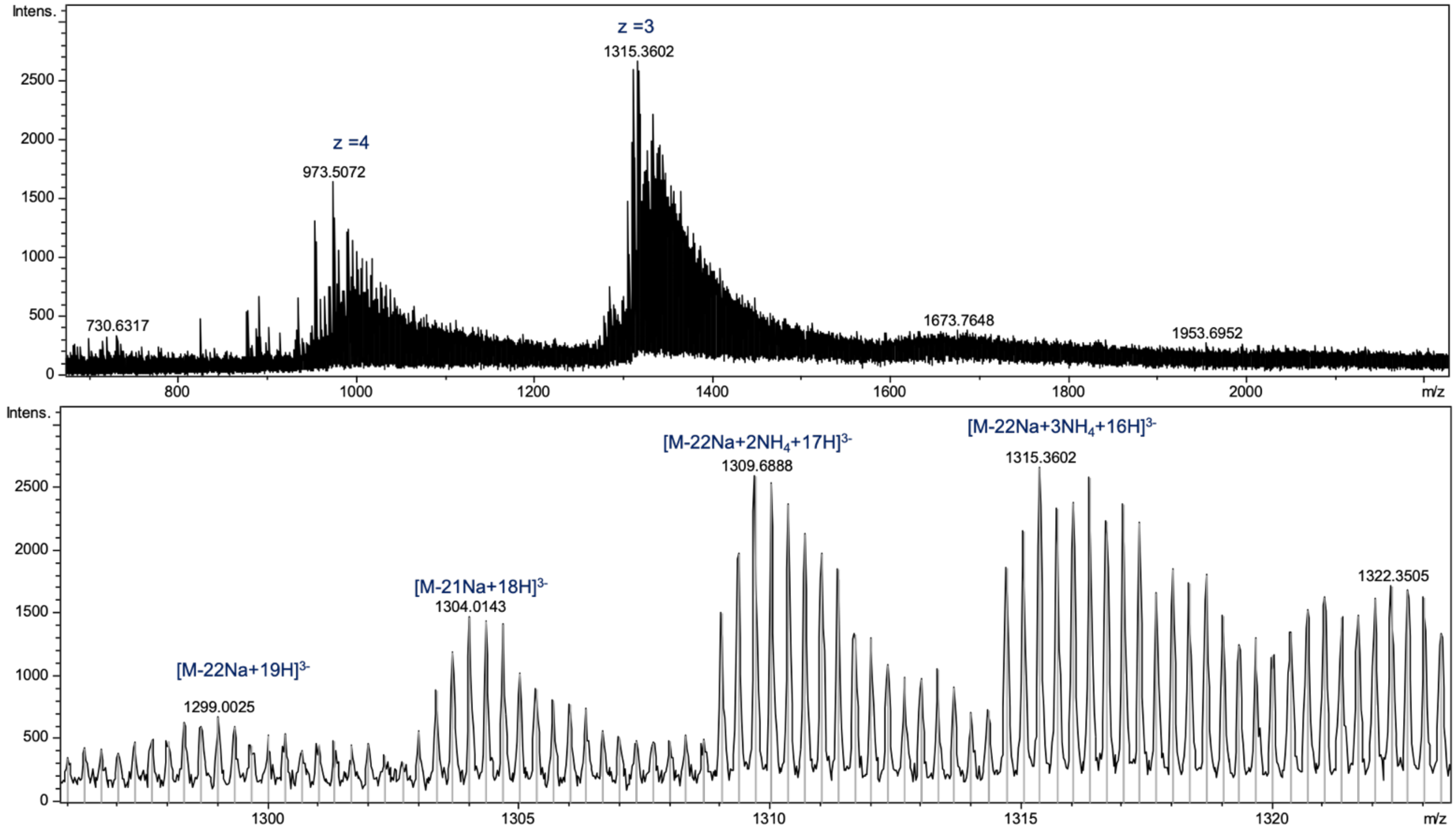

^{1}H NMR spectrum of **13** (600 MHz, D_2O)

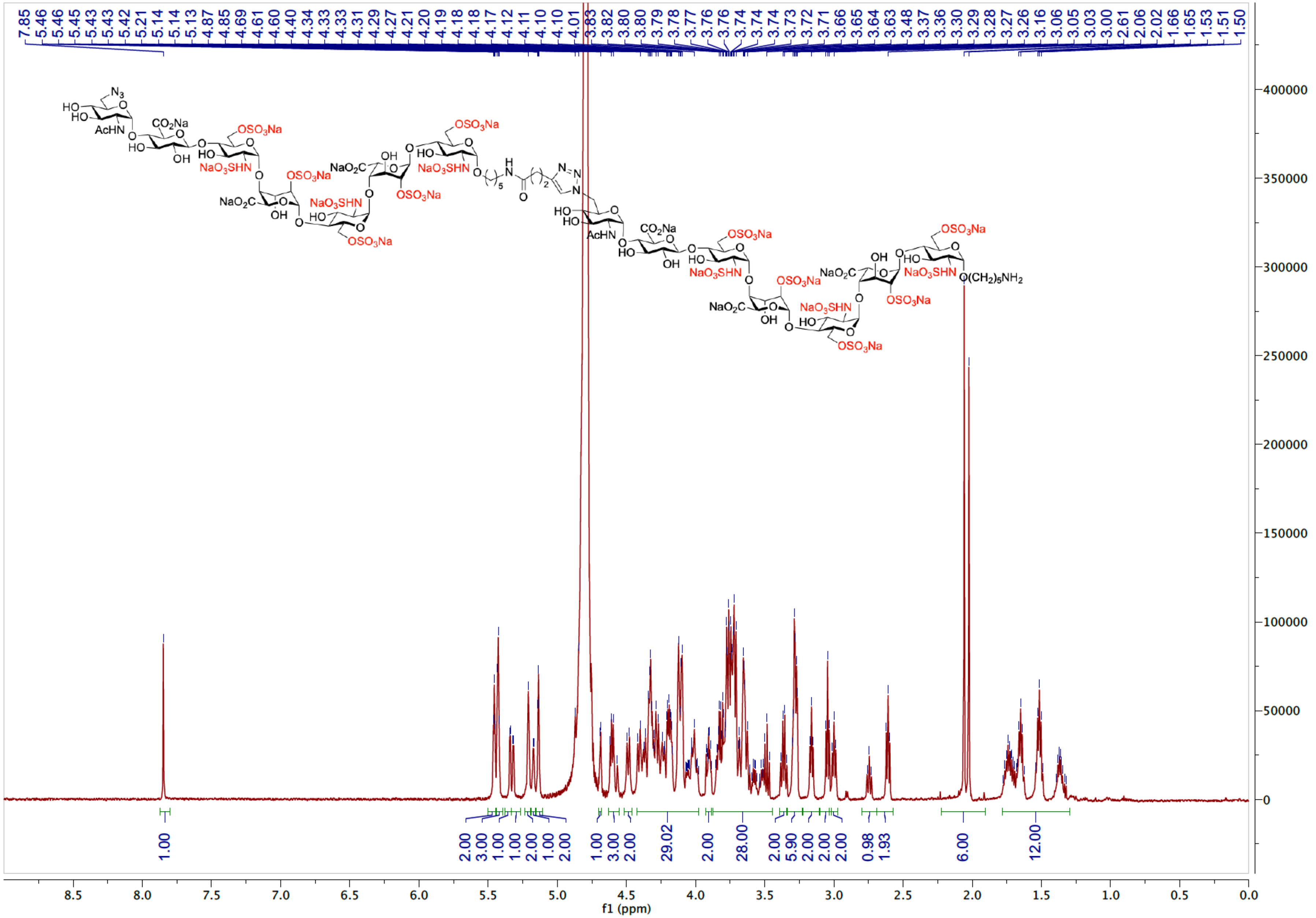

HSQC spectrum of **13** (D_2O)

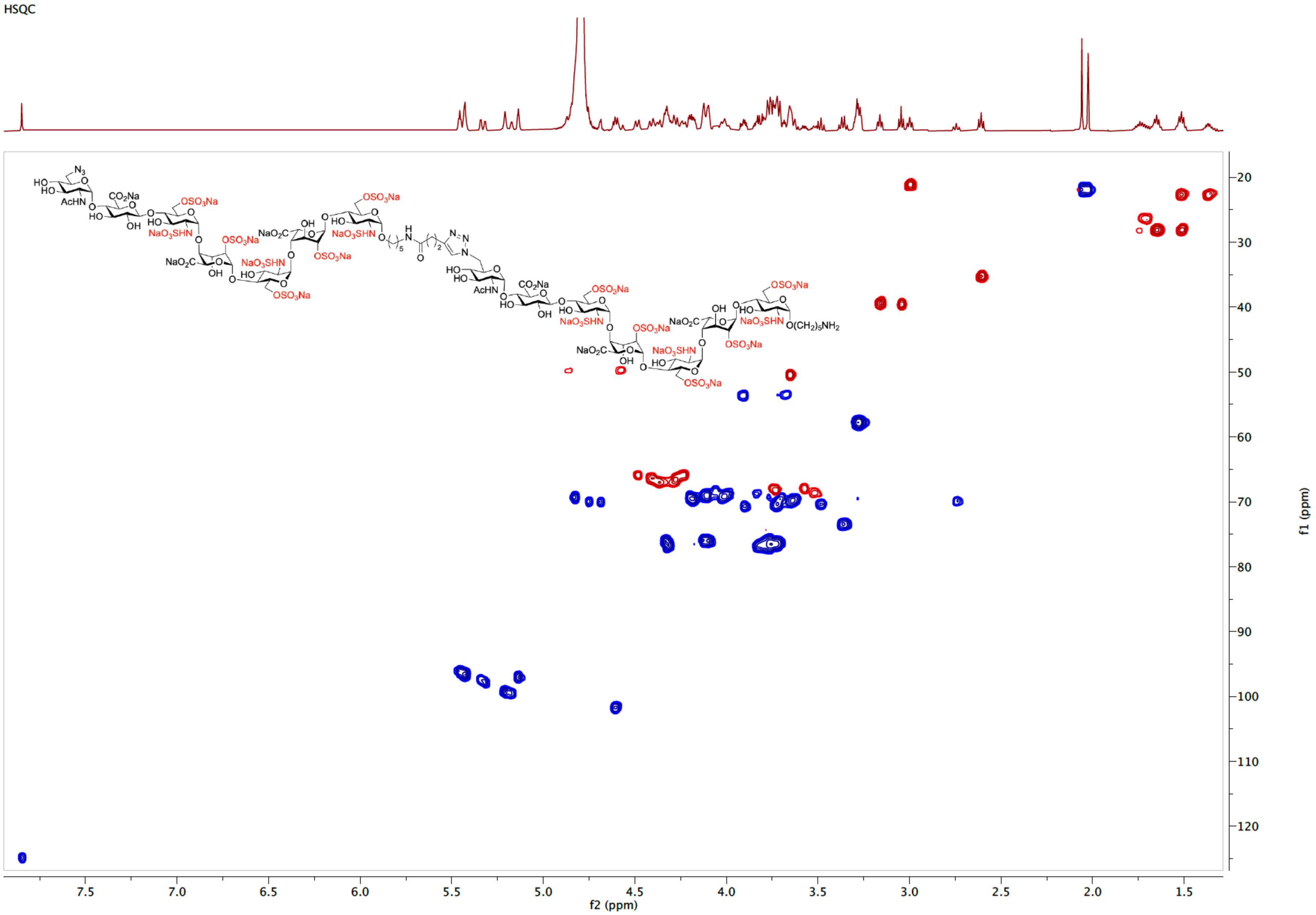

COSY spectrum of **13** (D_2O)

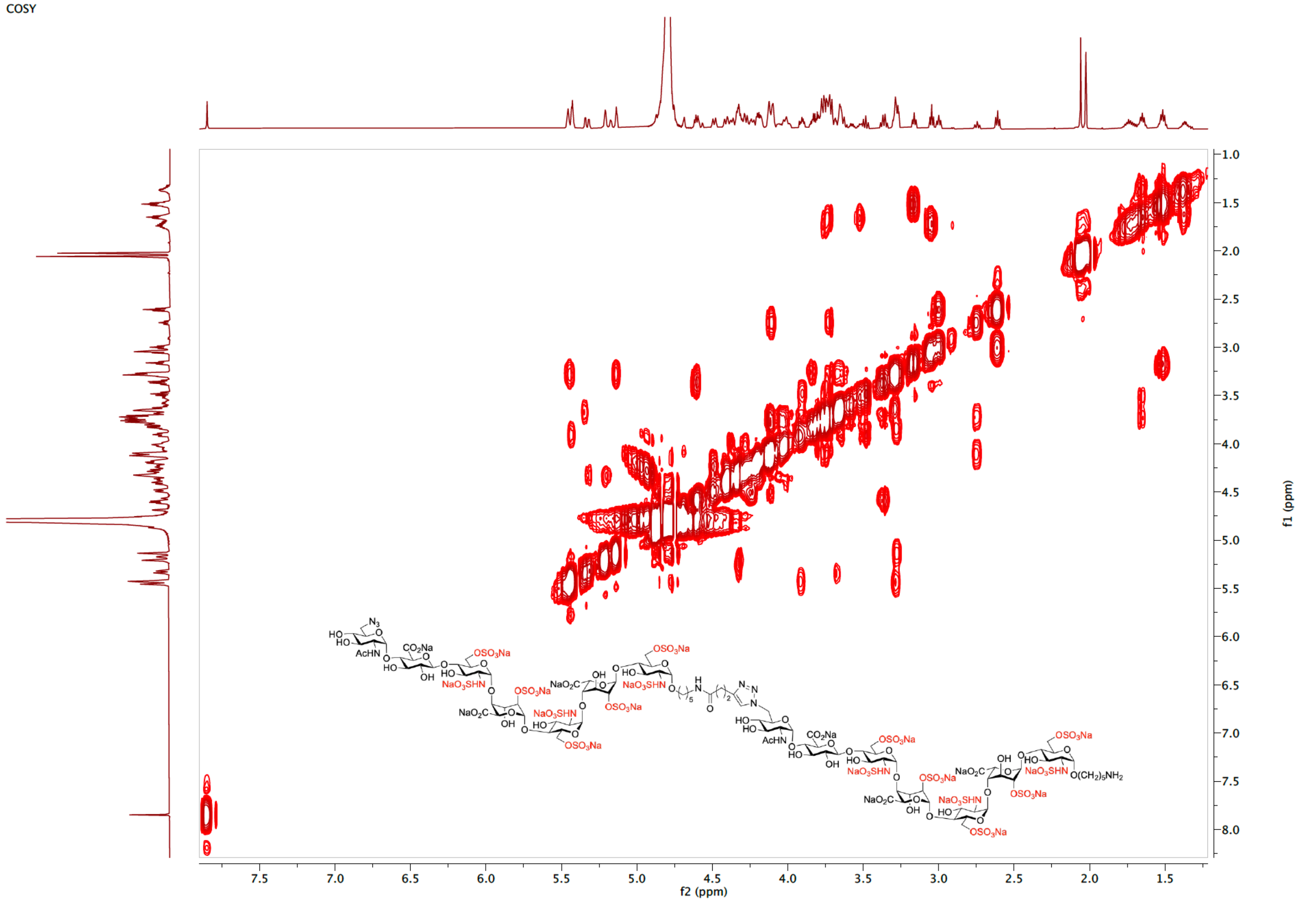

TOCSY spectrum of **13** (D_2O)

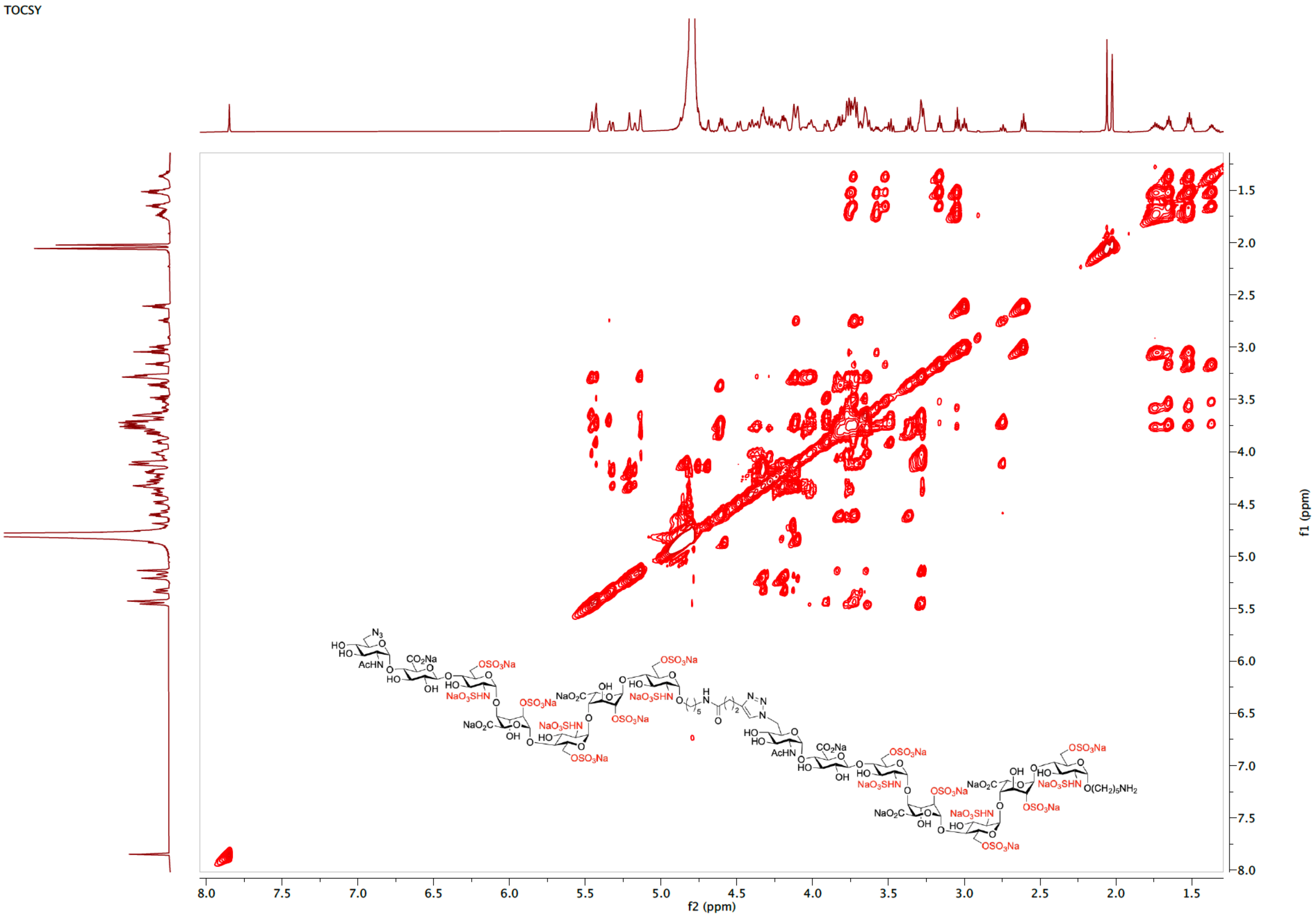

NOESY spectrum of **13** (D_2O)

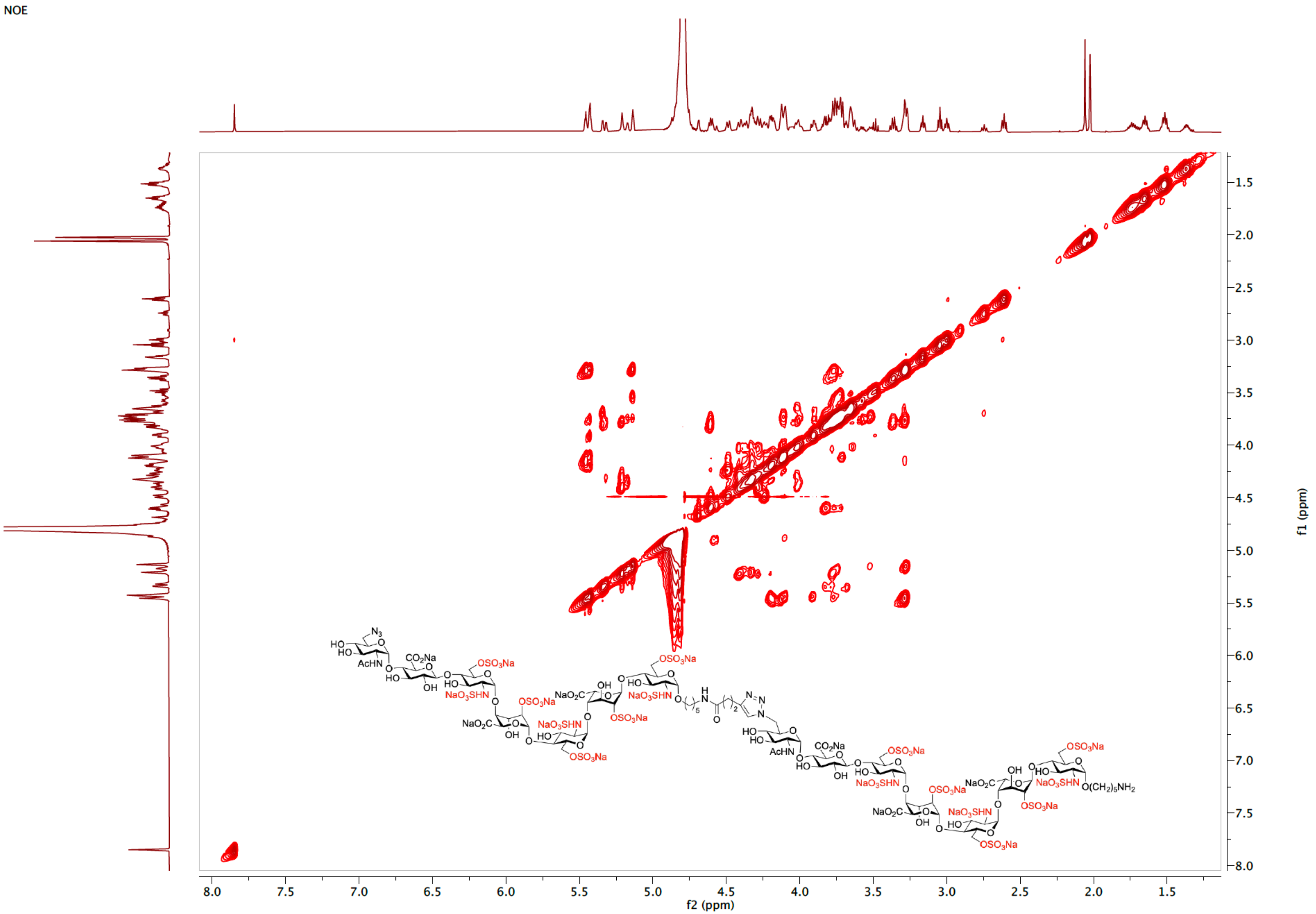

ESI-MS (negative) of **13**

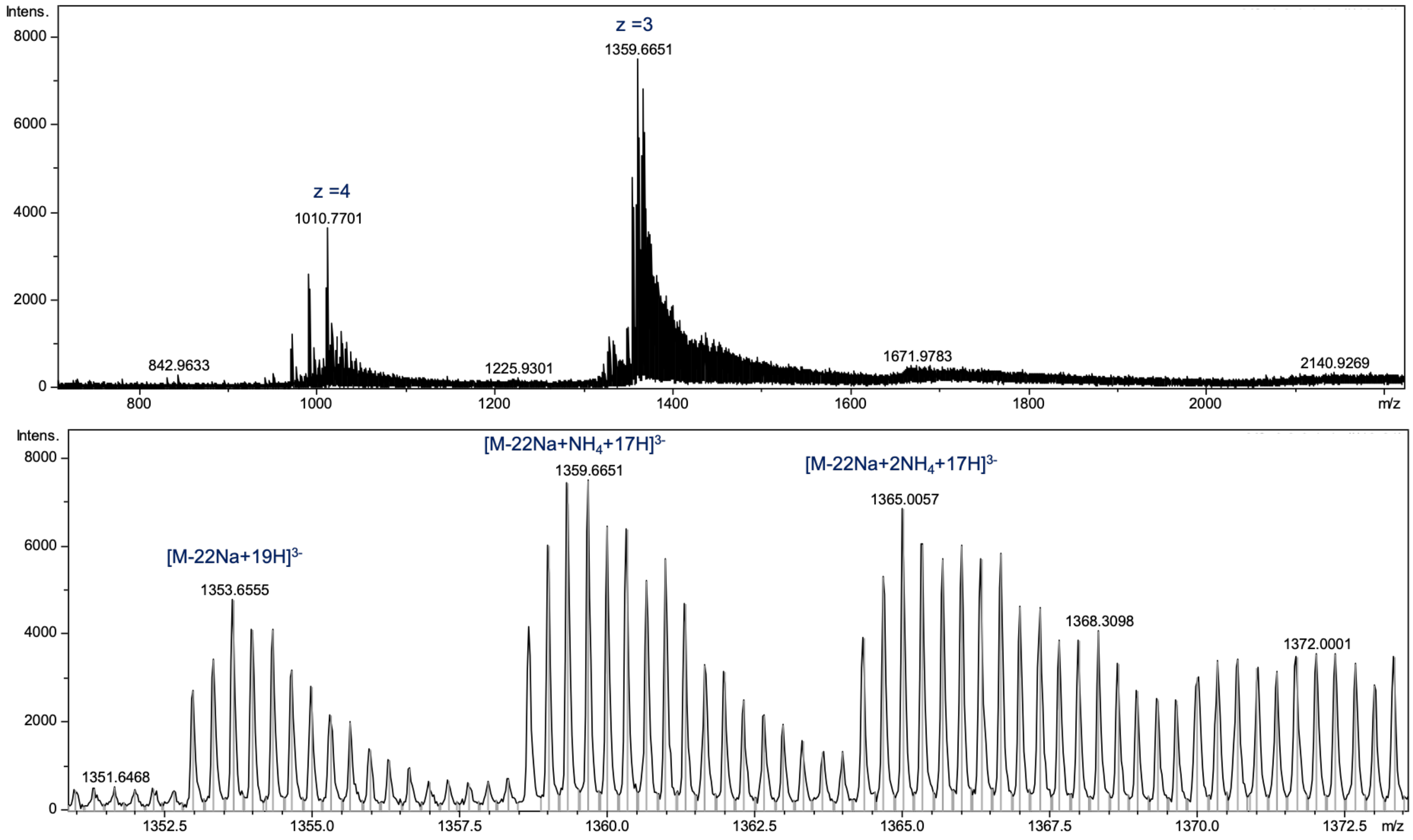

^{1}H NMR spectrum of **5** (600 MHz, D_2O)

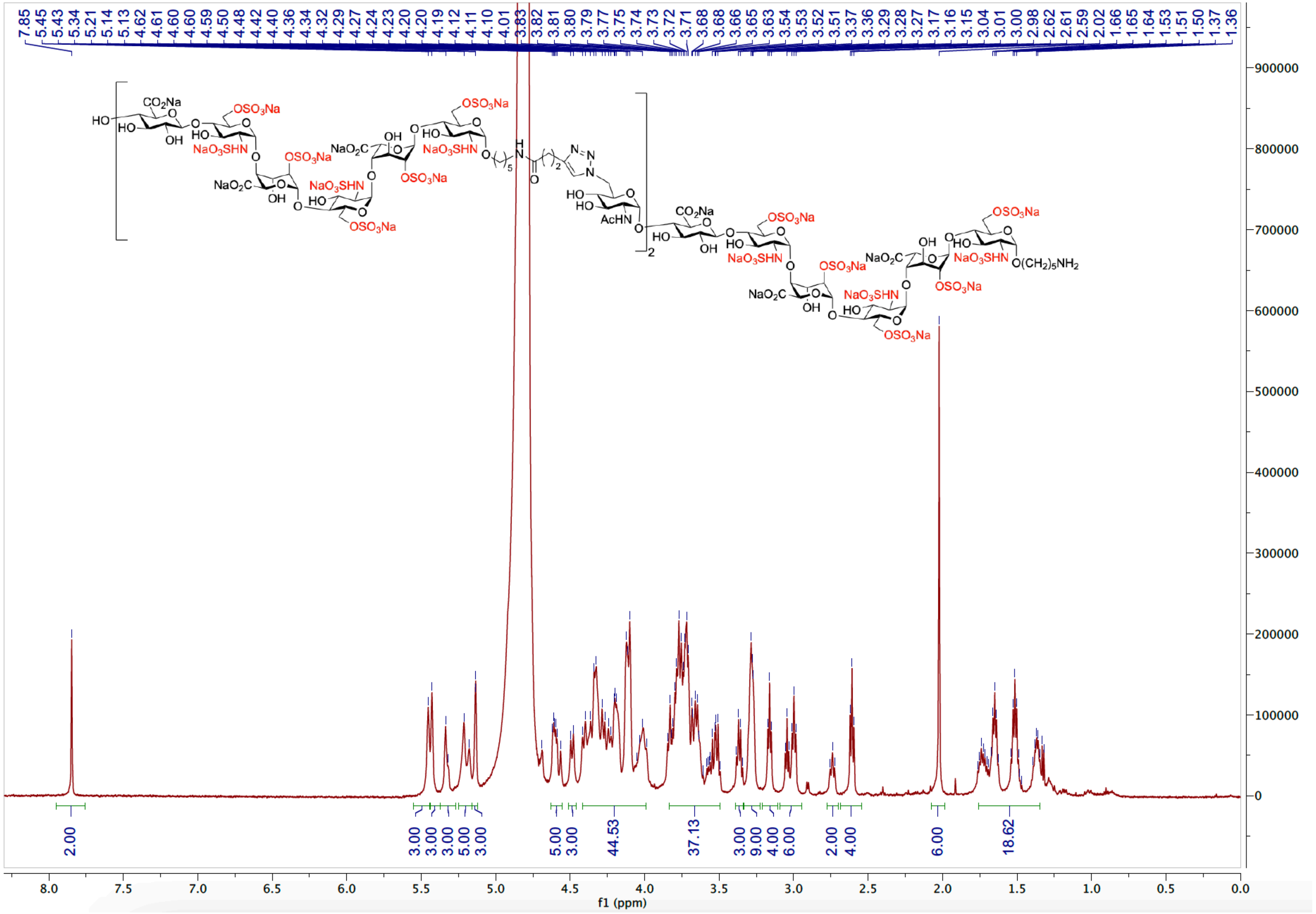

HSQC spectrum of **5** (D_2O)

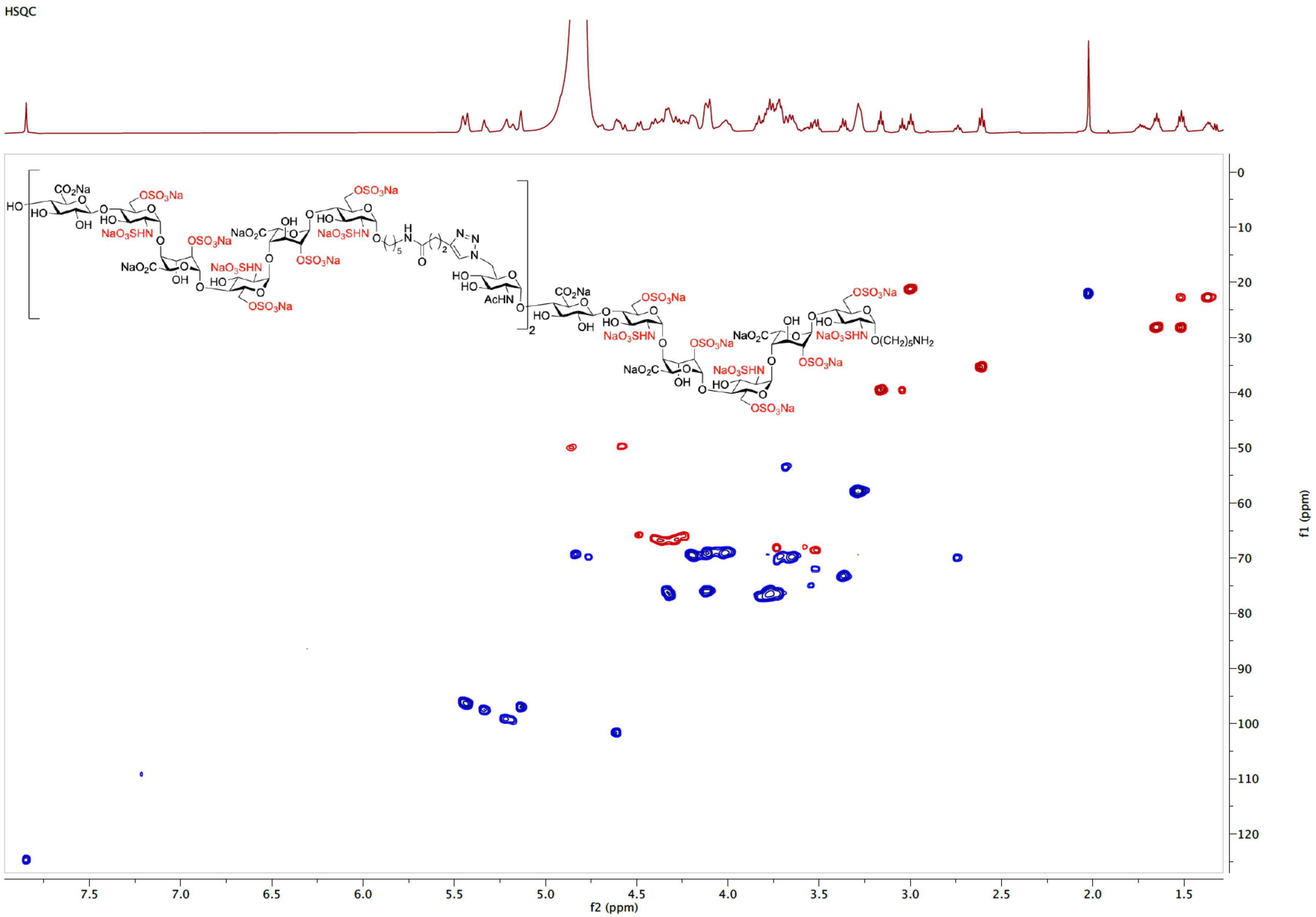

COSY spectrum of **5** (D_2O)

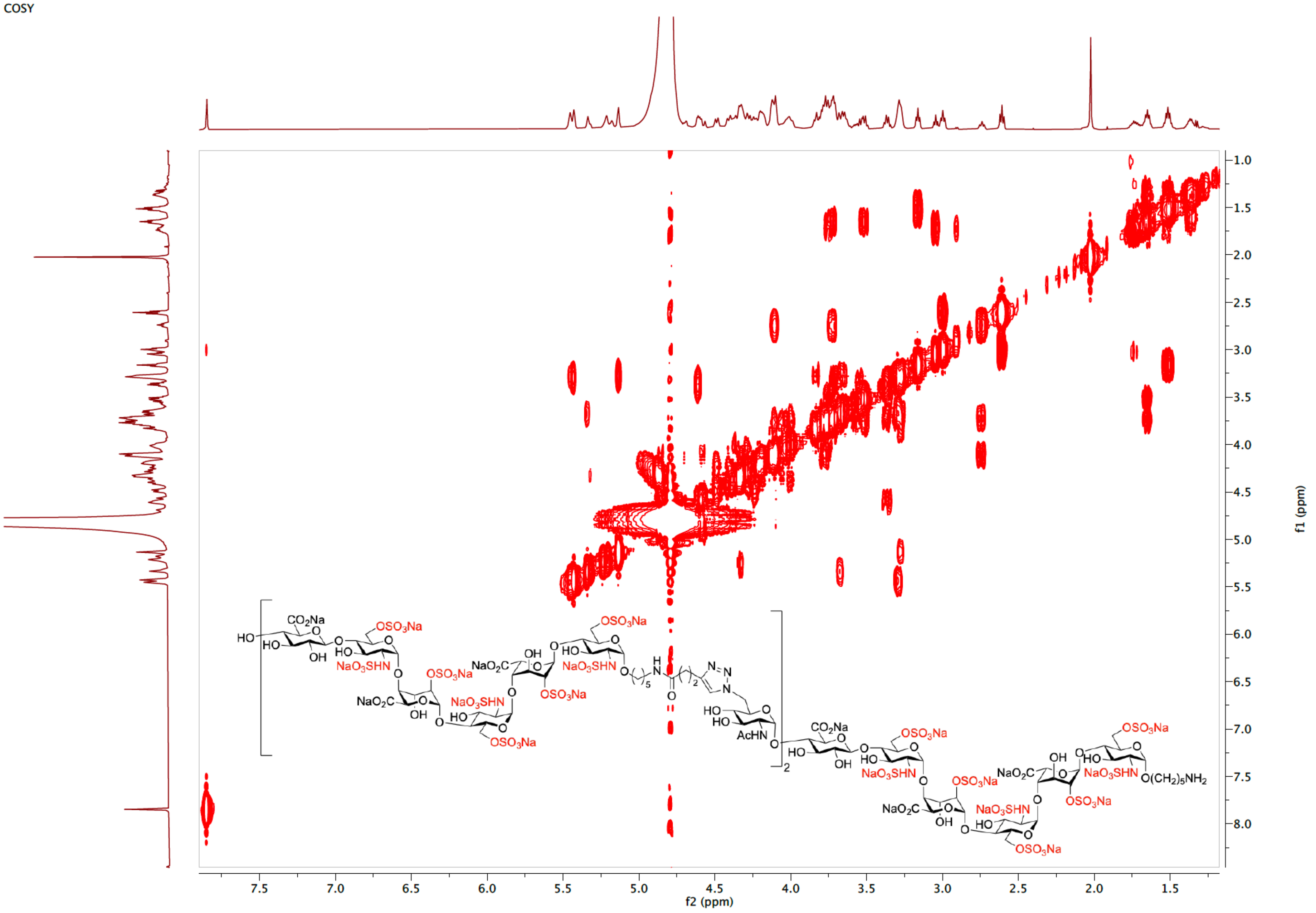

TOCSY spectrum of **5** (D_2O)

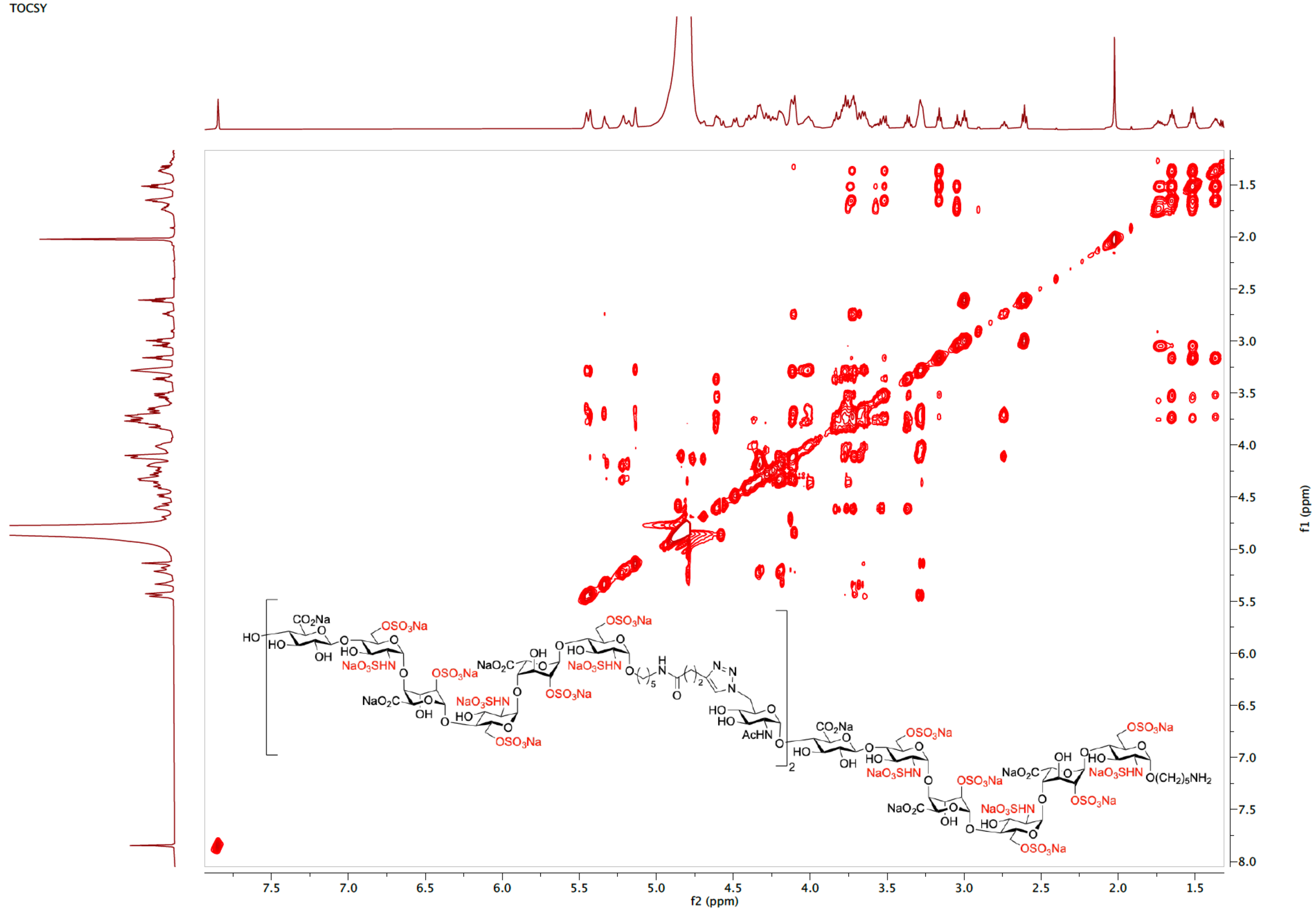

NOESY spectrum of **5** (D_2O)

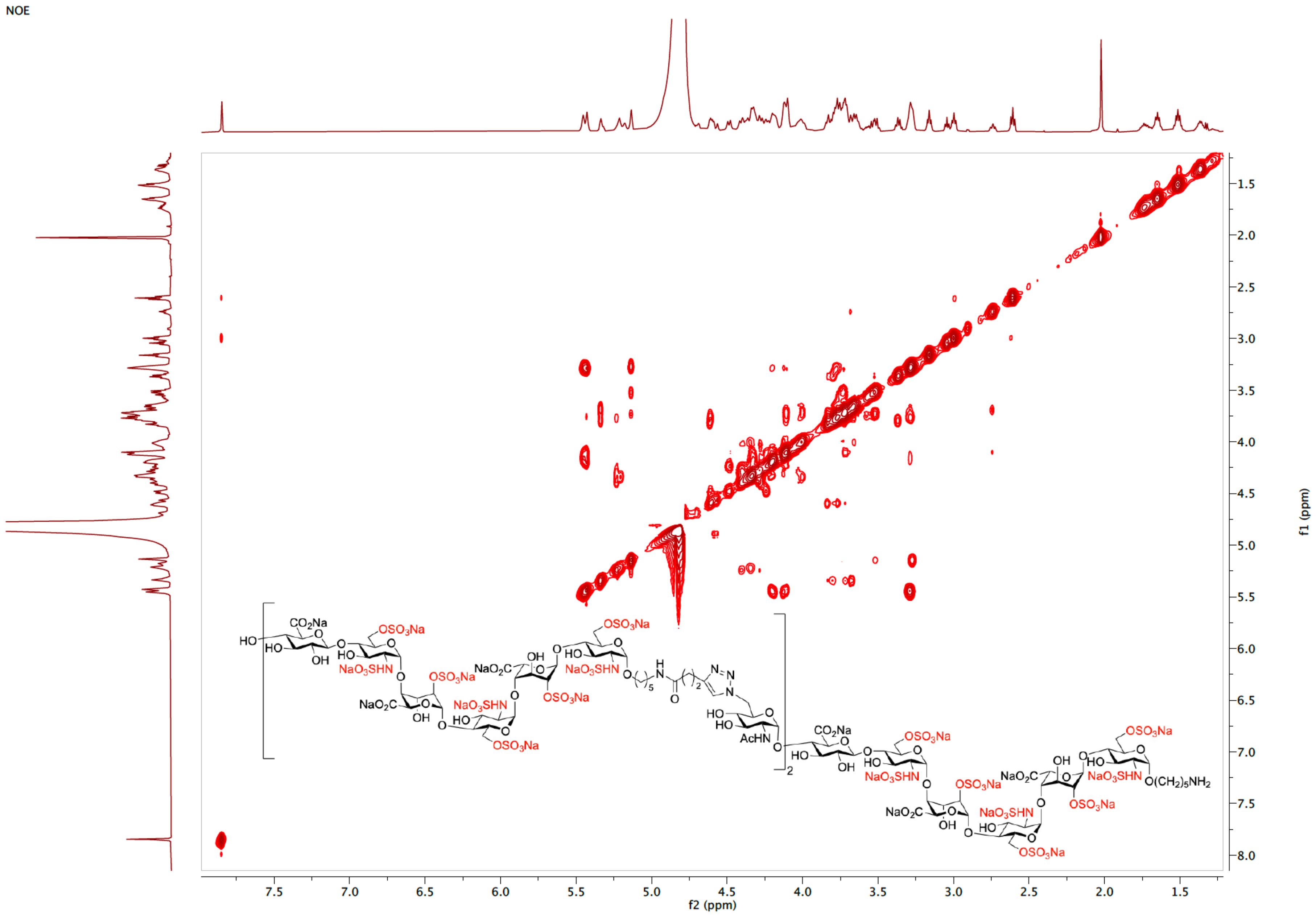

ESI-MS (negative) of **5**

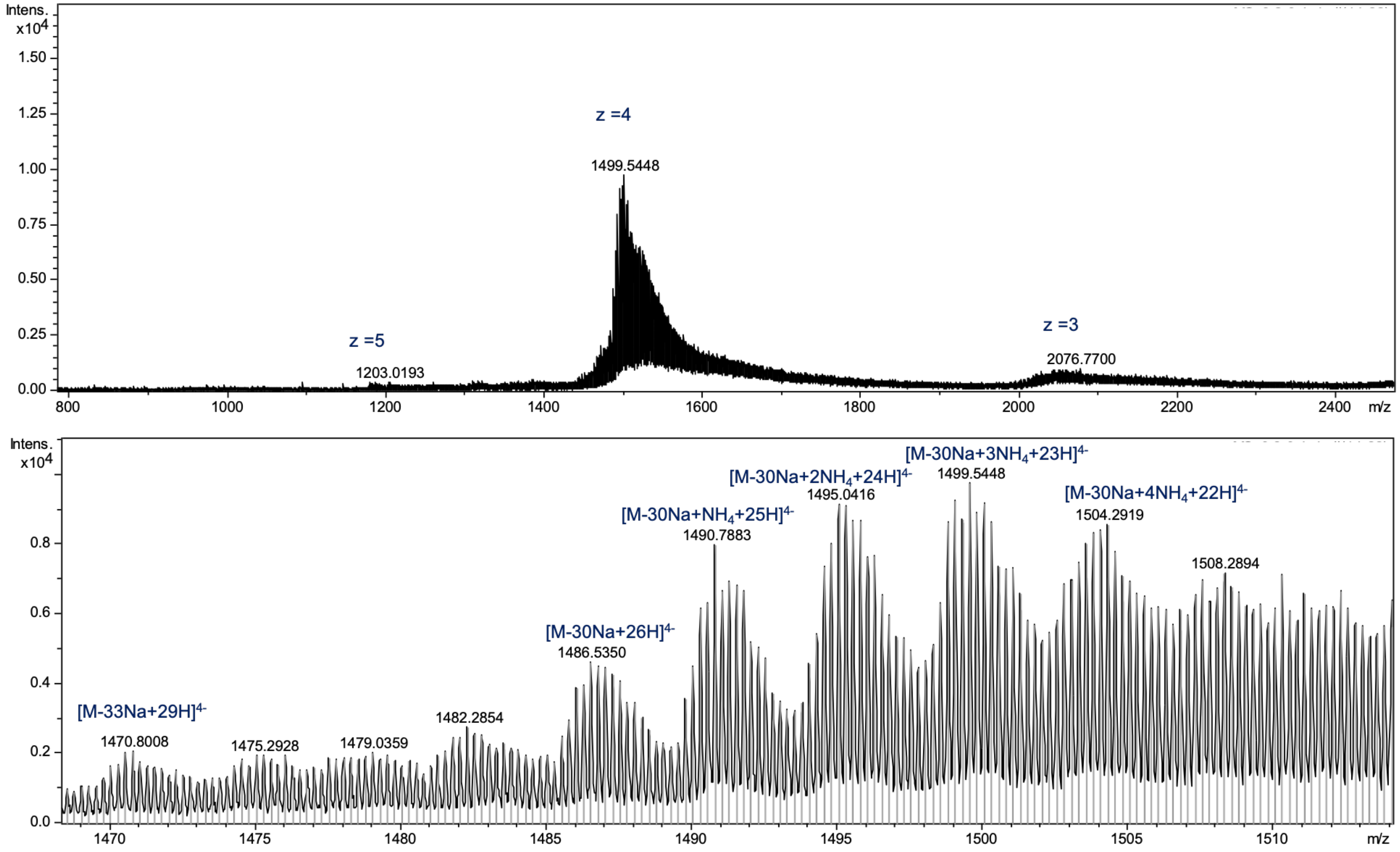

^{1}H NMR spectrum of **6** (600 MHz, D_2O)

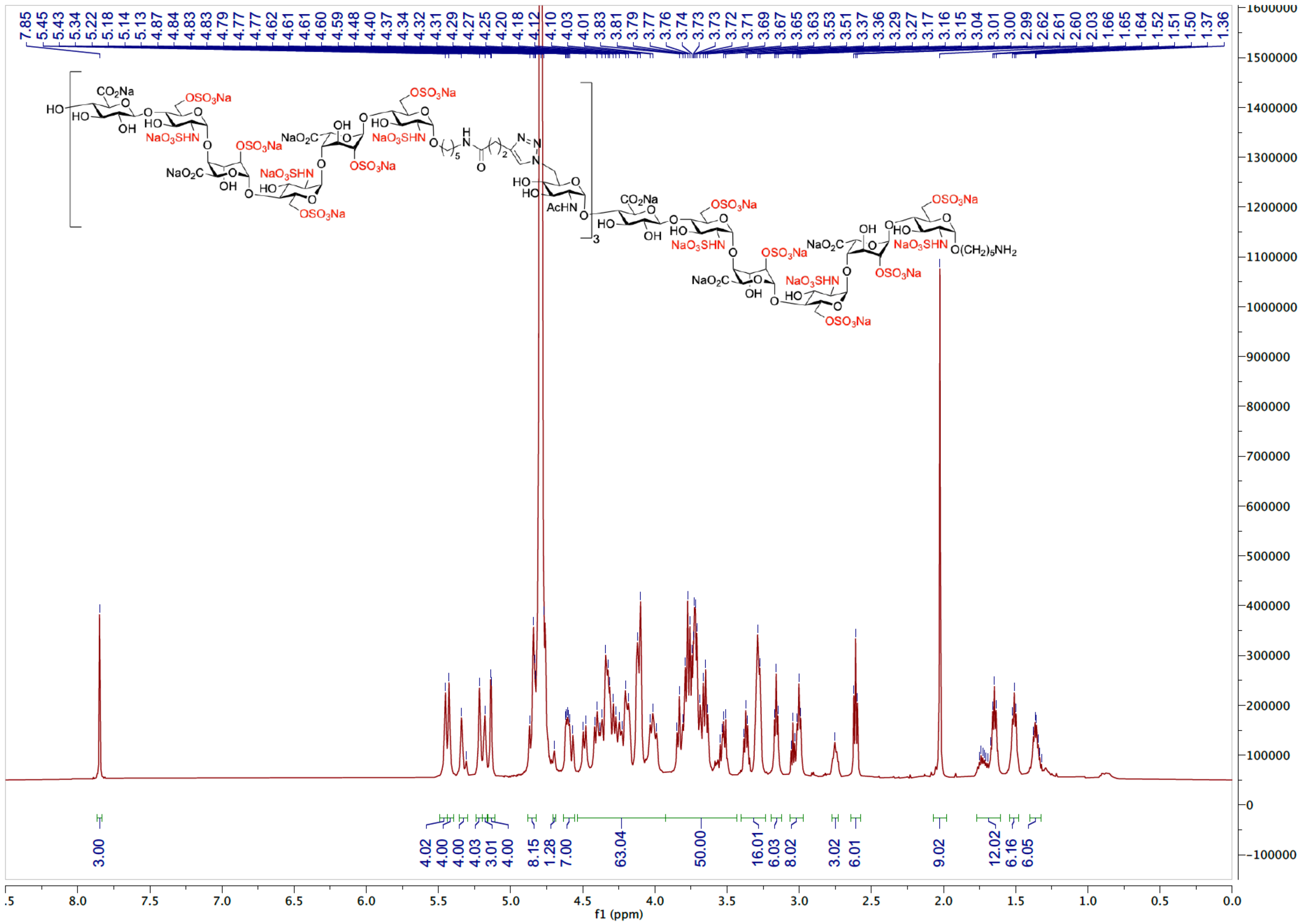

HSQC spectrum of **6** (D_2O)

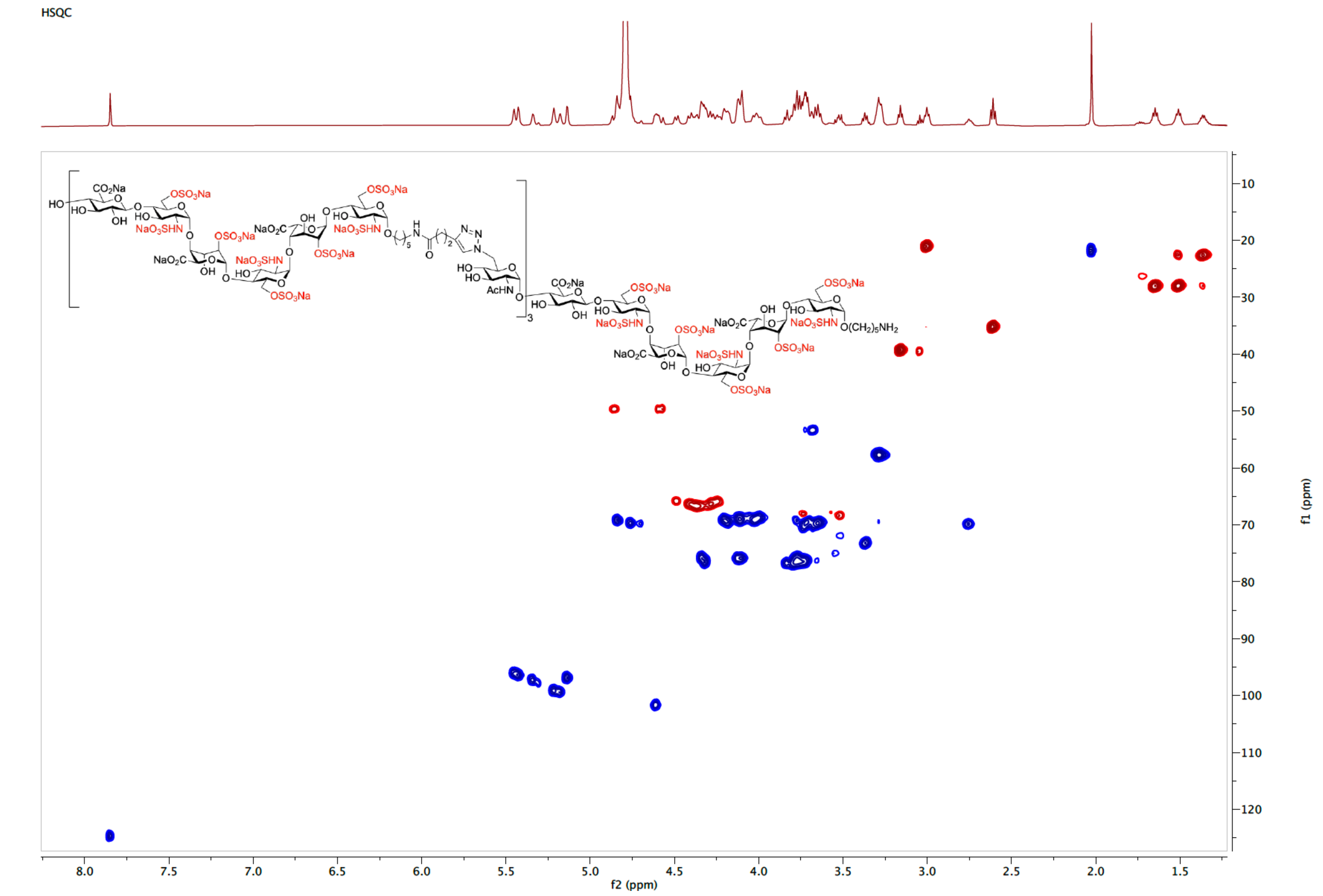

COSY spectrum of **6** (D_2O)

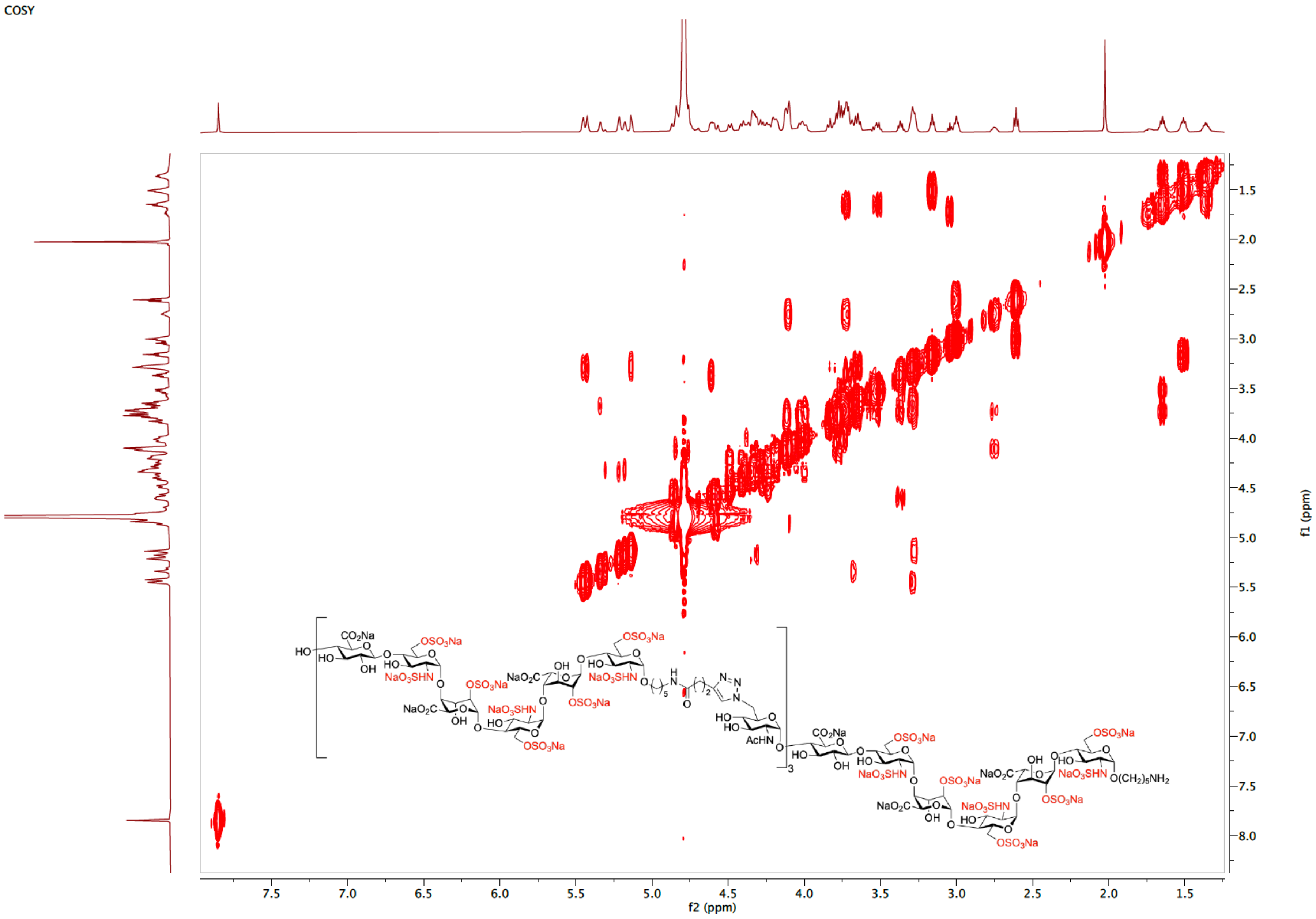

TOCSY spectrum of **6** (D_2O)

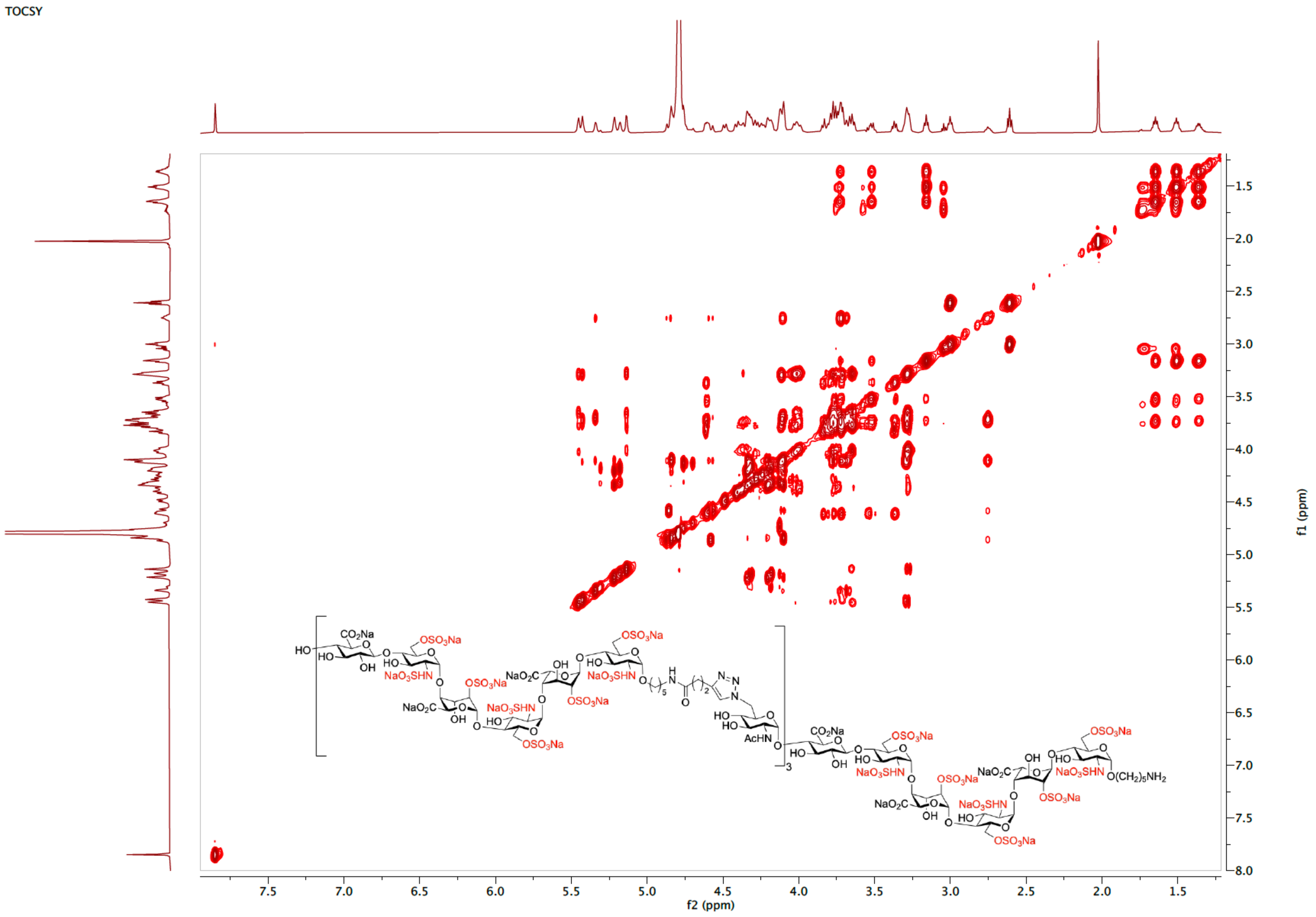

NOESY spectrum of **6** (D_2O)

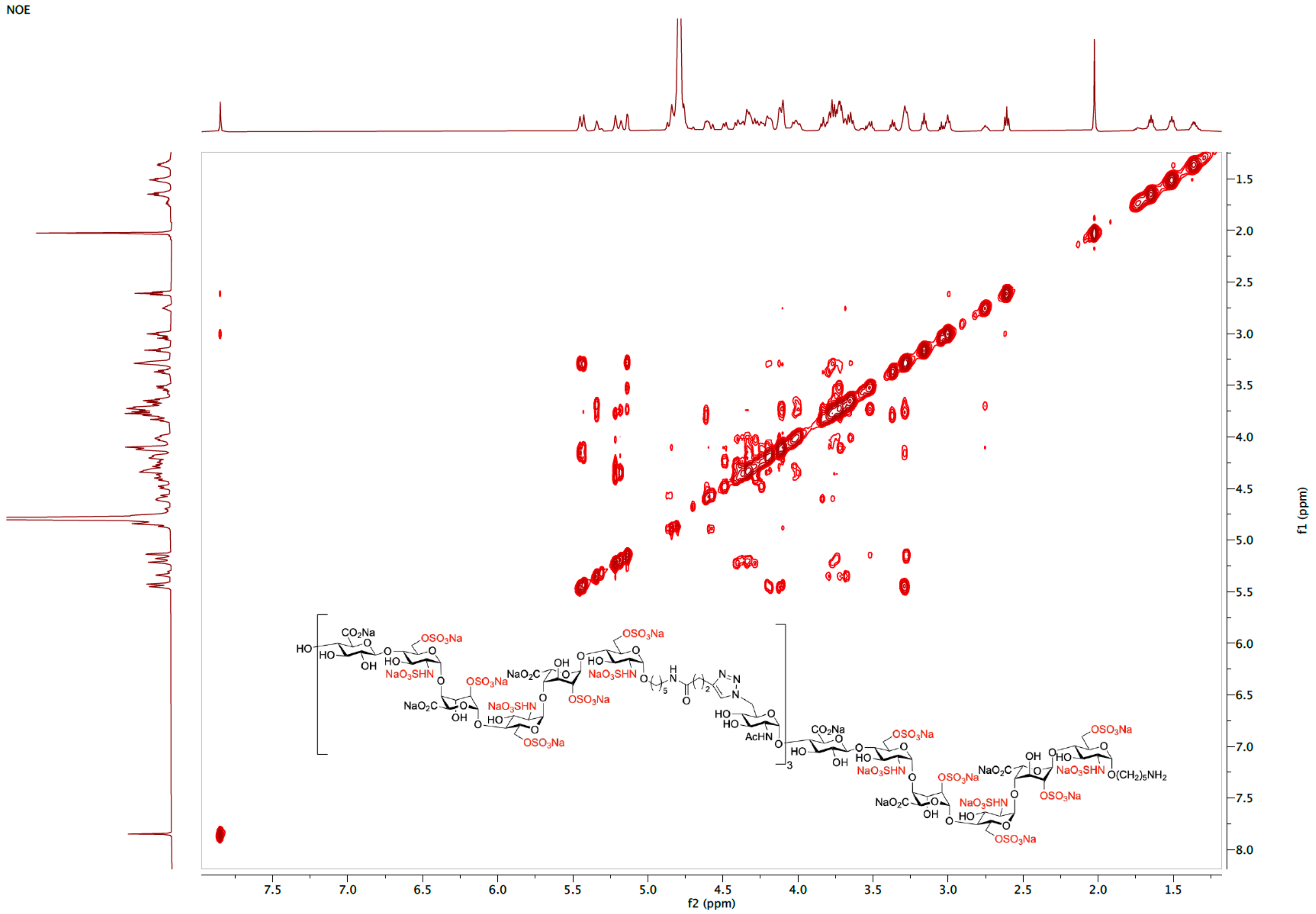

ESI-MS (negative) of **6**

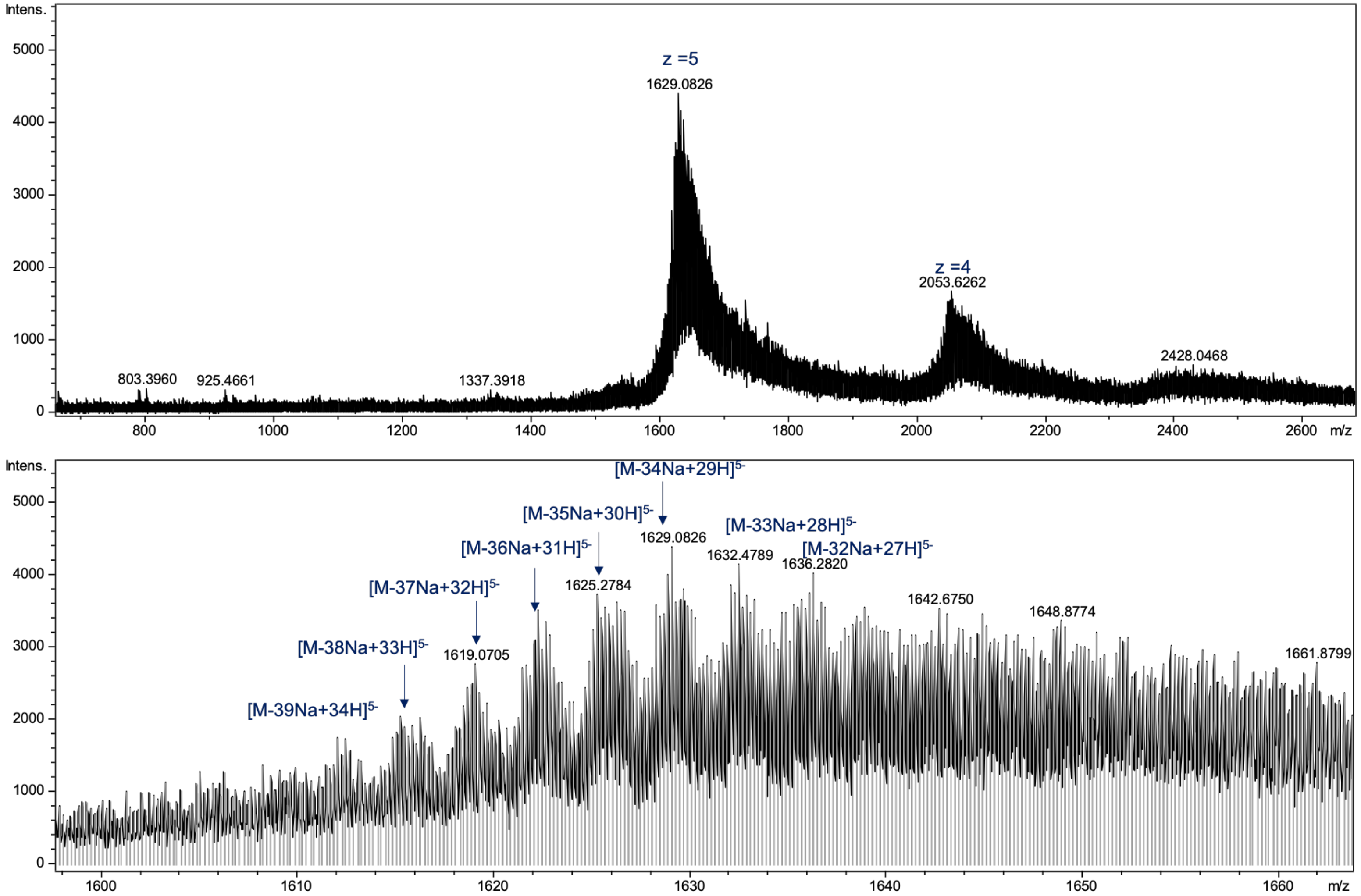

Supplement: Supplementary file 1 — au3c00042_si_001.pdf [file au3c00042_si_001.pdf]
